# Genetic Investigation and Transcriptome Profiling in a Nuclear Family With Peutz–Jeghers Syndrome

**DOI:** 10.1155/humu/5530710

**Published:** 2025-08-15

**Authors:** Tahir N. Khan, Chunyu Liu, Kai Lee Yap, Humayoon Shafique Satti, Ayaz Khan, Muhammad Safeer, Sheraz Khan, Naveed Altaf Malik, Feng Zhang, Muhammad Tariq, Erica E. Davis

**Affiliations:** ^1^Advanced Center for Translational and Genetic Medicine, Stanley Manne Children's Research Institute, Ann & Robert H. Lurie Children's Hospital of Chicago, Chicago, Illinois, USA; ^2^Department of Pediatrics and Department of Cell and Developmental Biology, Feinberg School of Medicine, Northwestern University, Chicago, Illinois, USA; ^3^Department of Biological Sciences, National University of Medical Sciences, Rawalpindi, Pakistan; ^4^International Peace Maternity and Child Health Hospital, Shanghai Key Laboratory of Embryo Original Diseases, School of Medicine, Shanghai Jiao Tong University, Shanghai, China; ^5^Department of Pathology & Laboratory Medicine, Ann & Robert H. Lurie Children's Hospital of Chicago, Chicago, Illinois, USA; ^6^Department of Pathology, Feinberg School of Medicine, Northwestern University, Chicago, Illinois, USA; ^7^National Institute for Biotechnology and Genetic Engineering College, Pakistan Institute of Engineering and Applied Sciences (NIBGE-C, PIEAS), Faisalabad, Pakistan; ^8^Department of Biotechnology and Genetic Engineering, Hazara University, Mansehra, Pakistan

**Keywords:** case report, disease severity, DNA repair, p53 signaling

## Abstract

Peutz–Jeghers syndrome (PJS) is a rare autosomal dominant disorder hallmarked by mucocutaneous melanocytic macules and gastrointestinal hamartomatous polyposis associated with germline/somatic pathogenic variants in the tumor suppressor *STK11*. PJS is clinically heterogeneous; however, the relationship between clinical phenotype and genotype remains elusive. Here, we report a family with PJS that harbors a heterozygous *STK11* whole gene deletion combined with a heterozygous variant in *TP53AIP1* that segregates with mucocutaneous pigmentation in the family. RNA-seq analysis followed by qRT-PCR confirmed that the expression of *STK11*, *TP53*, and *TP53AIP1* and a large fraction of p53 signaling pathway components are significantly reduced, while Wnt signaling pathway effectors are upregulated in cells from an affected individual. Our findings shed light on transcriptome-level pathway dysregulation in PJS with germline deletion of *STK11*. Further evaluation of mutational burden across relevant signaling pathways can likely inform disease prognosis.

## 1. Introduction

Peutz–Jeghers syndrome (PJS, MIM 175200) is a clinically rare autosomal dominant disease characterized by pigmented spots on the lips, mucous membranes, and extremities; scattered gastrointestinal polyps; and susceptibility to tumors [1]. Patients with PJS are at an increased risk (53%–93%) for developing gastrointestinal malignancies [2] with colorectal cancer being the most common (a lifetime risk of 39%) [3]. Most patients die due to complications arising from cancers at a median age of 45 years; however, deaths at a younger age (3–20 years) have been reported due to intussusception [4]. Pathogenic germline and somatic variants, including whole gene deletion of *STK11* (also known as *LKB1*), have been implicated in the etiology of PJS [5–7]. Specifically, impaired *STK11* can result in the disruption of the P53 signaling pathway, which is critical for tumor suppression; the resultant altered P53 activity is suggested to contribute to increased cancer susceptibility observed in individuals with PJS [8].

*STK11* encodes serine/threonine kinase 11, which is involved in processes such as embryonic development, cell polarity, cell cycle arrest, apoptosis, and metabolism. *STK11* is a major tumor suppressor gene [9], and pathogenic *STK11* variants have been detected in a variety of disseminated cancers [10]. For example, in non–small cell lung cancer (NSCLC) tissue and/or circulating tumor DNA, *STK11* and *KRAS* alterations frequently occur together, and *STK11* variants, especially when coupled with pathogenic *KRAS* variants, can lead to resistance against immunotherapy in these cancers [11]. Similarly, STK11 directly interacts with VEGFR2 in tumors, which function to reduce angiogenesis during tumorigenesis; therefore, loss of STK11 function can lead to increased angiogenesis, thereby promoting metastasis [12]. Moreover, STK11 regulates AMPK, which regulates mTOR and HIF-1*α*; thus, loss-of-function variants in *STK11* can lead to the dysregulation of mTOR and HIF-1*α*, promoting oncogenesis [13]. *STK11* variants can also modulate the tumor immune microenvironment. For instance, in lung adenocarcinoma, *STK11* variants alter the tumor immune microenvironment by affecting *CD1E* expression, which regulates the differentiation of macrophages [14]. In breast cancer, STK11 signaling influences the tumor immune microenvironment through interactions with tumor-infiltrating immune cells [15].

Here, we report a Pakistani family with a clinical diagnosis of PJS, in which all five available affected individuals segregate a germline heterozygous deletion spanning the entire *STK11* locus. Further, our findings suggest that a deleterious variant in the tumor suppressor gene *TP53AIP1* may play a role in intensifying the severity of disease symptoms.

## 2. Material and Methods

### 2.1. Ethical Statement and Study Subjects

**Figure 1:**
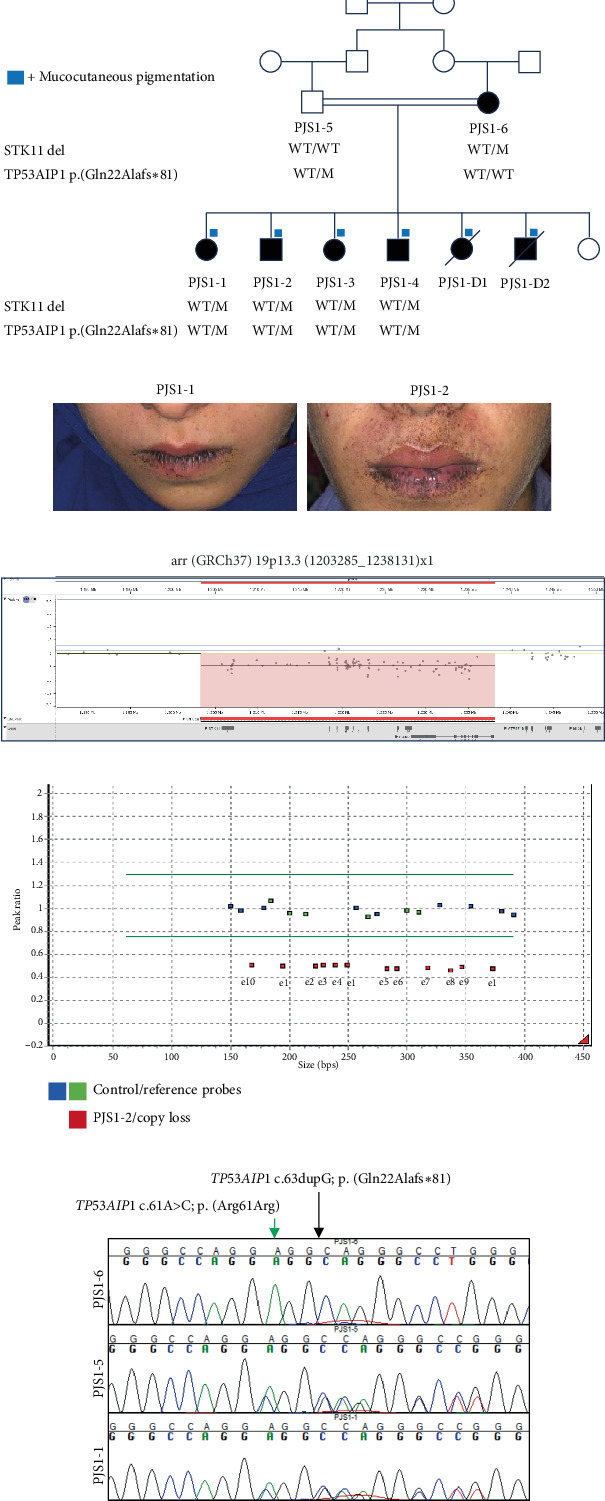
Clinical and genetic findings of individuals with Peutz–Jeghers syndrome in family PJS1. (a) Pedigree and clinical summary. The double horizontal lines in the pedigree indicate consanguinity; squares and circles symbolize males and females, respectively; filled symbols indicate affected individuals diagnosed with PJS; and unfilled symbols indicate healthy individuals. Deceased individuals are marked with a diagonal line. Individuals manifesting mucocutaneous pigmentations are marked with a blue rectangle in the pedigree. The genotypes for identified variants are indicated below each symbol. Del, *STK11* whole gene deletion; WT, wild type; M, mutant. (b) Pictures of individuals PJS1-1 and PJS1-2 showing melanocutaneous hyperpigmentation on and around the lips. (c) Schematic of microarray results on chromosome 19p13.3 showing copy number loss in individual PJS1-2 encompassing the *STK11* locus. Genomic coordinates of the copy loss are given on top of the schematic. (d) Multiplex ligation–dependent probe validation of *STK11* copy loss at exon (e) resolution. (e) DNA sequence chromatograms showing the region encompassing a rare variant identified in *TP53AIP1*. Individual identifiers corresponding to each chromatogram are given on the left side of each chromatogram. The variant position is indicated with a black arrow. The green arrow denotes the position of the synonymous variant *TP53AIP1* c.61A>C; p.(Arg61Arg) in PJS1-6 and PJS1-1.

In this study, we investigated a Pakistani family in which seven individuals were diagnosed with PJS. We obtained DNA from five affected individuals (mother and four affected siblings) and the healthy father (Figure 1a). PJS1-D1 and PJS1-D2 died within 1 year after the clinical diagnosis of PJS but before we enrolled the family for this genetics research study; thus, their DNA samples were not available.

Informed written consent for this study was obtained from all family members, and the study protocol was approved by the Institutional Review Boards of the National Institute for Biotechnology and Genetic Engineering College, Pakistan Institute of Engineering and Applied Sciences (NIBGE-C, PIEAS), Faisalabad, Pakistan, in accordance with the Helsinki Declaration.

### 2.2. Exome Sequencing (ES)

DNA was extracted from peripheral blood obtained from study participants using the GeneJET Genomic DNA Purification Kit (Thermo Scientific). ES was performed on three individuals: PJS1-1, PJS1-2, and PJS1-6. For ES, genomic DNA was target enriched by the Agilent SureSelectXT Human All Exon Kit. Next-generation sequencing was conducted with the Illumina HiSeq X TEN platform at a commercial facility (Cloud Health Genomics). Reads were aligned to the human genome reference assembly (UCSC Genome Browser hg19) with Burrows–Wheeler Aligner (BWA) software. Picard software was employed to remove PCR duplicates and evaluate the quality of variants to attain effective reads, quality bases, average coverage depth, and coverage ratio. Single-nucleotide variants (SNVs) and indels were called and analyzed with GATK using an in-house variant filtration pipeline. We then used ANNOVAR for functional annotation with OMIM, Gene Ontology, KEGG pathway, SIFT, PolyPhen-2, and MutationTaster.

For ES analysis, variants were filtered to retain rare variants with a minor allele frequency (MAF) of < 0.01 in relevant ethnicity-matched populations in control databases (including the Genome Aggregation Database [gnomAD] v2.1.1, the Exome Aggregation Consortium [ExAC], and the 1000 Genomes Project). Additional filters used for variant calling were a minimum coverage of ≥ 10x, allele balance of ≥ 0.3 for heterozygous and ≥ 0.8 for homozygous variants, and genotype quality equal to 99.

### 2.3. Sanger Sequencing

We performed Sanger sequencing of the *STK11* promoter region and secondary validation of the *TP53AIP1* variant using BigDye Terminator v3.1 cycle sequencing chemistry applied to PCR fragments amplified from regions of interest. Bidirectional sequencing was carried out on an ABI3730 Genome Analyzer according to standard protocols. Oligonucleotide sequences are listed in Table S1.

### 2.4. SNP Microarray

We performed SNP microarray using the Infinium Global Diversity Array (Cytogenetics & Enhanced PGx), a customized SNP microarray with 1.8 million probes that is designed to detect genome-wide copy number gains/losses and regions of homozygosity. Data analysis and clinical interpretation were performed on the NxClinical software (Bionano) using the reference UCSC hg19 human genome (NCBI build 37, Feb 2009), as outlined by current practice guidelines [16].

### 2.5. Multiplex Ligation–Dependent Probe Amplification (MLPA)

MLPA was performed on genomic DNA obtained from all six available family members (PJS1-1 to PJS1-6) and an unrelated healthy control using the SALSA MLPA kit and STK11 Probemix P101-B4 (MRC Holland) following the manufacturer's protocol. Capillary electrophoresis of the amplified fragments was performed on an ABI3500 instrument. MLPA data analysis was completed using GeneMarker (Version2.7.4, SoftGenetics).

### 2.6. Primary Dermal Fibroblast Cell Culture

We obtained primary dermal fibroblasts from an affected individual (PJS1-2, age 16 years) and an ethnically matched control individual (24 years of age, used as a control individual for all aspects of this study) and cultured cells in DMEM (Sigma-Aldrich), 10% fetal bovine serum, and 1% penicillin–streptomycin in a sterile T25 flask. We incubated cells in an aseptic, humidified environment at 37°C with 5% CO_2_. Cells from both control and PJS1-2 were in Passage Number 3. We extracted total RNA from nontransformed, growth-synchronized cells at ~80% confluency with the RNeasy Mini Kit (Qiagen).

### 2.7. RNA-Seq and qRT-PCR Validation

RNA-seq was performed on total RNA extracted from nontransformed cultured dermal fibroblasts in technical triplicates (*N* = 3) for each of PJS1-2 and the ethnically matched healthy control individual. The stranded mRNA-seq was conducted in the Northwestern University NUSeq Core Facility, as described previously [17]. Briefly, total RNA examples were checked for quality using RNA integrity numbers (RINs) generated from Agilent Bioanalyzer 2100. RNA quantity was determined with a Qubit fluorometer. The Illumina Stranded mRNA Prep Kit was used to prepare sequencing libraries from 100 ng of high-quality RNA samples (all samples had RIN > 8). The kit procedure was performed without modifications. This procedure includes mRNA purification and fragmentation, cDNA synthesis, 3⁣′ end adenylation, Illumina adapter ligation, and library PCR amplification and validation. The lllumina HiSeq 4000 sequencer was used to sequence the libraries with the production of single-end, 50 bp reads at the depth of 40–50 M reads per sample. The quality of reads, in FASTQ format, was evaluated using FastQC. Reads were trimmed to remove Illumina adapters from the 3⁣′ ends using cutadapt [18]. Trimmed reads were aligned to the human genome (hg38) using STAR [19]. Read counts for each gene were calculated using htseq-count [20] in conjunction with a gene annotation file for hg38 obtained from Ensembl (http://useast.ensembl.org/index.html). Normalization and differential expression were calculated using DESeq2 that employs the Wald test [21]. The cutoff for determining significantly differentially expressed genes was an FDR-adjusted *p* value less than 0.05 using the Benjamini–Hochberg method, protein coding genes only, log2 fold change of ≤ 0.5 or ≥ 0.5X, and minimum of 50x mean coverage in each of control and patient groups. Gene list enrichment analysis and visualization was performed using Enrichr [22], iDEP [23], and Pathview [24] web tools. Gene expression levels for a subset of genes (*STK11*, *TP53*, and *TP53AIP1*) were validated in patient cells by qRT-PCR using SYBR Green chemistry using *GAPDH* as normalization control. For this purpose, cDNA was synthesized from mRNA obtained from cultured, primary dermal fibroblasts of PJS1-2 and the ethnically matched healthy control the using QuantiTect Reverse Transcription Kit (Qiagen). Each qRT-PCR reaction was conducted in technical replicates (*N* = 3). Statistical significance of change in gene expression was calculated using a *t*-test. See Table S1 for oligo sequences used in qRT-PCR.

## 3. Results

### 3.1. Clinical Description

Two affected siblings (now deceased), a female (PJS1-D1; 17 years of age) and a male (PJS1-D2; 16 years of age), complained of severe abdominal pain and presented with massive rectal bleeding (Figure 1a). They had melanotic mucocutaneous pigmentation localized around the mouth, eyes, nostrils, and perianal area, which is suggestive of PJS. Histological examination of a section of the small intestine identified three polyps, with the largest measuring 4.5 × 4 × 3 cm and the smallest measuring 2.5 × 2.5 × 2.5 cm. Computed tomography scan showed that the small intestine loops had a swirling pattern. Intervening mesentery and blood vessels were observed telescoping into each other in the left hemiabdomen. These findings were suggestive of intussusception. External surfaces appeared homogeneous and pale white in color, and extensive areas of hemorrhage were visible. Histopathological examination of a biopsy of the small intestine in PJS1-D2 revealed areas of extensive hemorrhagic infarction. Histological sections prepared from the polyps revealed an arborizing network of connective tissue. Smooth muscle and lamina propria were lined by intestinal glands. Bundles of smooth muscle were evident through the lamina propria. Pools of acellular mucin and glands were also observed in the muscular layer, but no atypia was seen. In follow-up colonoscopy, multiple polyps of variable sizes were observed up to the splenic flexure. These polyps were removed by endoscopic polypectomy. Colonoscopic examination of the other four children (age 9–15 years), who had the mucocutaneous pigmentation (Figure 1a,b), also confirmed the presence of hamartomatous polyps, although the numbers were not determined. The intensity and number of the pigment spots in affected children have increased with age on and around the lip tissue. The mother of these patients (PJS1-6; age 45 years at the time of assessment) does not present with mucocutaneous pigmentation on the external skin or on the buccal mucosa and complained of no abdominal pain. An interview with her parents confirmed the absence of cutaneous pigmentation in her childhood. However, guided by family history, a colonoscopic examination confirmed the presence of polyps. However, she is yet to undergo a polypectomy. The father of the affected children (PJS1-5) was asymptomatic (age 47 years) and never underwent colonoscopic examination.

### 3.2. Genetic Analyses

To identify the underlying genetic cause of PJS, we performed ES in two affected siblings (PJS1-2 and PJS1-3) and their affected mother (PJS1-6) to an average read depth of 124x. After variant filtration for autosomal dominant disease, no variant was identified that was shared by all three affected individuals subjected to ES. Additionally, no homozygous variants were found to be shared by all three affected individuals or by the two affected children for which the mother is heterozygous. Similarly, we performed Sanger sequencing of the *STK11* predicted promoter [25] region, and no variation was found to be shared among affected individuals.

To screen for large deletions, we subjected DNA from PJS1-2 to genome-wide SNP copy number microarray analysis, which revealed a heterozygous deletion spanning the entire *STK11* locus (Figure 1c). Segregation analysis of the *STK11* deletion was performed in all available family members using MLPA; all affected individuals were also found to harbor the heterozygous deletion, whereas the father (PJS-5) had the expected two copies of *STK11* (Figure 1d). We note that MLPA segregation analysis was performed on DNA obtained from peripheral blood samples of all family members, including the mother (PJS1-6), which excludes the possibility of mosaic somatic deletion transmitted from mother to children. Additionally, we observed multiple common variants in *STK11* (all with MAF > 10% in public databases) in PJS1-6 ES data that have a 100% variant allele fraction, consistent with germline copy loss.

**Figure 2:**
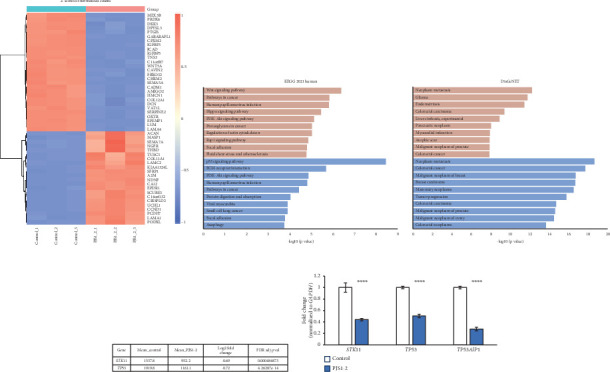
RNA-seq and pathway analyses in a PJS patient. (a) RNA-seq heat map showing the Top 50 differentially expressed genes in PJS1-2 patient-derived fibroblasts compared to matched healthy control fibroblasts. (b) Gene enrichment analysis of upregulated (highlighted in pink) and downregulated (highlighted in blue) pathways from RNA-seq. (c) Gene enrichment analysis of gene-disease association when upregulated (highlighted in pink) and downregulated (highlighted in blue). (d) RNA-seq expression results of *STK11* and *TP53* showing fold reduction in PJS1-2. See Table S2 for more details. (e) qRT-PCR validation of selected differentially expressed genes identified by RNA-seq. Error bars, standard error of mean. *p* values (⁣^∗∗∗∗^; < 0.0001) are indicated on top of each comparison.

RNA-seq and subsequent bioinformatics filtering in cultured fibroblasts obtained from PJS1-2 revealed the downregulation of 945 genes, including *STK11*, and the upregulation of 926 genes (Figure 2a and Table S2). Gene enrichment and pathway analysis showed that p53 signaling and Wnt signaling are the most significantly downregulated and upregulated molecular pathways, respectively, in patients' dermal fibroblasts (Figure 2b,c). We validated the expression of *STK11* and *TP53* using qRT-PCR and found their downregulation to be concordant with the RNA-seq analysis (Figure 2d,e).

To investigate the genetic basis of the observed mucocutaneous pigmentation, we performed reanalysis of the ES data. We asked whether there were rare variants (MAF < 1% in public databases) present in both siblings with pigmentation that were absent from the mother, who did not present with pigmentation. We identified 134 variants that fulfilled these criteria (Table S3). Further examination of this list of variants by relevant biological function revealed a heterozygous frameshift variant in *TP53AIP1* (NM_022112.2:c.63dupG; p.(Gln22Alafs∗81)). Loss of function variants in *TP53AIP1*, including c.63dupG; p.(Gln22Alafs∗81), have been reported previously to result in reduced expression of *TP53AIP1* and predispose to cutaneous melanoma [26]. Segregation analysis of the family using Sanger sequencing confirmed that the children inherited this variant from their father (PJS1-5) who was heterozygous (Figure 1a,e). To test for the presence of additional variants in *cis* to the *STK11* deletion, we searched for *STK11* variants that could be transmitted to affected children on the paternal allele (MAF < 10% in public databases); however, no variants were found. In our RNA-seq data, we sought to correlate *TP53AIP1* genotype with transcriptomic signature; however, there was no coverage/expression of *TP53AIP1* in both the PJS1-2 and healthy control, requiring an additional method of expression analysis. Subsequently, using qRT-PCR, we validated that the expression of *TP53AIP1* is reduced > 2 fold in PJS1-2 compared to healthy control (Figure 2e). Thus, reduced *TP53AIP1* conferred by this heterozygous loss-of-function variant is the only possible explanation for phenotype variability we could identify in this family.

## 4. Discussion

Complete germline deletion of *STK11* in patients with PJS has been reported previously [7]. To investigate further the precise role of STK11, signaling cascades, and molecular pathways underlying PJS in this family, we performed transcriptomic analysis using RNA-seq of primary dermal fibroblasts derived from PJS1-2 (Figures 2a, 2b, 2c, and 2d; Figures S1 and S2, and Table S2). Gene Ontology and enrichment analysis showed that Wnt signaling and p53 signaling pathways are among the top differentially regulated pathways (Figure 2b and Figures S3 and S4) compared to a matched control. These pathways are associated with malignancies including colorectal cancer (Figure 2c). Activation of Wnt signaling, for instance, has been identified as a major protumorigenic pathway in human disease [27]. Similarly, p53 signaling is significantly downregulated in PJS1-2, which is a hallmark of human colorectal carcinoma [28]. Notably, besides *TP53*, there are 26 other genes found to be differentially expressed in the p53 pathway in patient-derived cells (Figure S3) exemplifying the complexity of interactions that are involved in this pathway and associated with PJS.

A remaining unanswered question in this family is why there is mucocutaneous pigmentation in the affected children only and not in their affected mother. PJS cases with or without mucocutaneous pigmentation have been described [1]; however, no plausible molecular causes have been described for this variable manifestation. The only possible genetic explanation we identified in this family is a heterozygous frameshift variant in *TP53AIP1* present in genomic DNA from affected siblings, but not the maternal sample (Figure 1e and Table S3). The father, who carries this heterozygous variant, does not have skin hyperpigmentation, indicating that haploinsufficiency of *TP53AIP1* alone may not cause this phenotype but might increase the likelihood of skin hyperpigmentation.

*TP53AIP1* is a proapoptotic gene exerting its effect downstream of TP53. Recent studies have reported that reduced expression of *TP53AIP1* is associated with an increased risk of cancer. For instance, inhibition of *TP53AIP1* promotes cervical cancer development and metastasis via the activation of the TP53 signaling pathway [29]. Another study reports TP53AIP1 as an inducer of autophagy through the AKT/mTOR signaling pathway in a breast cancer cell line [30]. Previously, it has been shown that *TP53AIP1*:c.63dupG causes diminished *TP53AIP1* mRNA [26]; therefore, its reduction in PJS1-2 could be independent of *STK11* deletion. *TP53AIP1* is a TP53 target that plays a key role in apoptosis in response to UV-induced DNA damage [26]. Although there is growing evidence for the role of TP53AIP1 in various cancers, further functional investigation is required to examine the direct interaction of well-established cancer risk genes in conjunction with pathogenic *TP53AIP1* variants in increasing cancer severity. We are cautious in the interpretation of this result and recognize that confirming such a genetic risk association would require larger cohorts of individuals with variable PJS. We postulate that analysis of *STK11* lesions combined with known predisposing genetic variants is a feasible future prospect to improve individual cancer risk estimates and understand phenotype variability. Further genetic and comparative transcriptome studies are required in PJS cases with or without mucocutaneous pigmentations to establish the precise mechanism.

## 5. Conclusion

Together, the germline deletion of *STK11* dysregulates p53 signaling, DNA repair, and cell cycle pathways (Figures S3, S4, and S5) indicating their critical roles in PJS. Evaluation of *trans*-effects (i.e., mutational burden in tumor suppressor genes such as *TP53* or *TP53AIP1*) holds considerable promise as a means for informing disease prognosis in PJS.

## Data Availability

Supporting data are available from the corresponding authors upon reasonable request. Raw RNA-seq data are deposited to NCBI Gene Expression Omnibus (https://www.ncbi.nlm.nih.gov/geo/) under Accession Number GSE251755 and are publicly available. Filtered exome sequencing and RNA-seq data are included as supporting information in the manuscript.

## Supporting Information

Supporting Information Additional supporting information can be found online in the Supporting Information section. Table S1: List of primer sequences. Figure S1: Principal component analysis of RNA-seq. Figure S2: Volcano plot of differentially expressed protein coding genes obtained from RNA-seq analysis of PJS1-2 versus healthy matched control. Figure S3: Sketch of human p53 signaling pathway obtained from KEGG. Figure S4: Sketch of human Wnt signaling pathway obtained from KEGG. Figure S5: Sketch of the eukaryotic DNA mismatch repair pathway obtained from KEGG. Table S2: Differentially expressed genes in dermal fibroblasts from PJS1-2 versus a healthy control individual; *n* = 3 triplicates per genotype. Table S3: Filtered exome sequencing data showing 134 rare variants (MAF < 1% in public databases) present in both siblings (PJS1-1 and PJS1-2) and with mucocutaneous pigmentation that were absent from the mother (PJS1-6), who did not present with pigmentation.
