# Supplementary material for: Genetic Investigation and Transcriptome Profiling in a Nuclear Family With Peutz–Jeghers Syndrome
Source: Hum Mutat. 2025 Aug 15;2025:5530710. doi: 10.1155/humu/5530710 (PMC12373473; doi:10.1155/humu/5530710)
Supplement: Supporting Information — Additional supporting information can be found online in the Supporting Information section. Table S1: List of primer sequences. Figure S1: Principal component analysis of RNA-seq. Figure S2: Volcano plot of differentially expressed protein coding genes obtained from RNA-seq analysis of PJS1-2 versus healthy matched control. Figure S3: Sketch of human p53 signaling pathway obtained from KEGG. Figure S4: Sketch of human Wnt signaling pathway obtained from KEGG. Figure S5: Sketch of the eukaryotic DNA mismatch repair pathway obtained from KEGG. Table S2: Differentially expressed genes in dermal fibroblasts from PJS1-2 versus a healthy control individual; n = 3 triplicates per genotype. Table S3: Filtered exome sequencing data showing 134 rare variants (MAF < 1% in public databases) present in both siblings (PJS1-1 and PJS1-2) and with mucocutaneous pigmentation that were absent from the mother (PJS1-6), who did not present with pigmentation. [file 5530710.f1.zip › Khan et al, CARE-checklist_07.23.2025_final.pdf]

CARE
case report guidelines

CARE Checklist of information to include when writing a case report

(cc) BY-NC-ND

Topic	Item	Checklist item description	Reported on Line
**Title**	1	The diagnosis or intervention of primary focus followed by the words "case report"	✓ line 2
**Key Words**	2	2 to 5 key words that identify diagnoses or interventions in this case report, including "case report"	✓ line 49
**Abstract (no references)**	3a	Introduction: What is unique about this case and what does it add to the scientific literature?	✓ lines 39-41
	3b	Main symptoms and/or important clinical findings	✓ lines 35-37
	3c	The main diagnoses, therapeutic interventions, and outcomes	✓ line 39
	3d	Conclusion—What is the main "take-away" lesson(s) from this case?	✓ lines 44-47
**Introduction**	4	One or two paragraphs summarizing why this case is unique (**may include references**)	✓ lines 83-86
**Patient Information**	5a	De-identified patient specific information.	✓ line 350
	5b	Primary concerns and symptoms of the patient.	✓ lines 178-205
	5c	Medical, family, and psycho-social history including relevant genetic information	✓ lines 206-225
	5d	Relevant past interventions with outcomes	n/a
**Clinical Findings**	6	Describe significant physical examination (PE) and important clinical findings.	✓ lines 178-205
**Timeline**	7	Historical and current information from this episode of care organized as a timeline	n/a
**Diagnostic Assessment**	8a	Diagnostic testing (such as PE, laboratory testing, imaging, surveys).	✓ lines 178-205
	8b	Diagnostic challenges (such as access to testing, financial, or cultural)	n/a
	8c	Diagnosis (including other diagnoses considered)	✓ lines 178-205
	8d	Prognosis (such as staging in oncology) where applicable	✓ lines 178-205
**Therapeutic Intervention**	9a	Types of therapeutic intervention (such as pharmacologic, surgical, preventive, self-care)	n/a
	9b	Administration of therapeutic intervention (such as dosage, strength, duration)	n/a
	9c	Changes in therapeutic intervention (with rationale)	n/a
**Follow-up and Outcomes**	10a	Clinician and patient-assessed outcomes (if available)	n/a
	10b	Important follow-up diagnostic and other test results	n/a
	10c	Intervention adherence and tolerability (How was this assessed?)	n/a
	10d	Adverse and unanticipated events	n/a
**Discussion**	11a	A scientific discussion of the strengths AND limitations associated with this case report	✓ lines 254-295
	11b	Discussion of the relevant medical literature **with references**.	✓ lines 254-295
	11c	The scientific rationale for any conclusions (including assessment of possible causes)	✓ lines 296-300
	11d	The primary "take-away" lessons of this case report (without references) in a one paragraph conclusion	✓ lines 296-300
**Patient Perspective**	12	The patient should share their perspective in one to two paragraphs on the treatment(s) they received	n/a
**Informed Consent**	13	Did the patient give informed consent? Please provide if requested	Yes ☑ No ☐
