# Supplementary material for: Genetic Investigation and Transcriptome Profiling in a Nuclear Family With Peutz–Jeghers Syndrome
Source: Hum Mutat. 2025 Aug 15;2025:5530710. doi: 10.1155/humu/5530710 (PMC12373473; doi:10.1155/humu/5530710)
Supplement: Supporting Information — Additional supporting information can be found online in the Supporting Information section. Table S1: List of primer sequences. Figure S1: Principal component analysis of RNA-seq. Figure S2: Volcano plot of differentially expressed protein coding genes obtained from RNA-seq analysis of PJS1-2 versus healthy matched control. Figure S3: Sketch of human p53 signaling pathway obtained from KEGG. Figure S4: Sketch of human Wnt signaling pathway obtained from KEGG. Figure S5: Sketch of the eukaryotic DNA mismatch repair pathway obtained from KEGG. Table S2: Differentially expressed genes in dermal fibroblasts from PJS1-2 versus a healthy control individual; n = 3 triplicates per genotype. Table S3: Filtered exome sequencing data showing 134 rare variants (MAF < 1% in public databases) present in both siblings (PJS1-1 and PJS1-2) and with mucocutaneous pigmentation that were absent from the mother (PJS1-6), who did not present with pigmentation. [file 5530710.f1.zip › Khan et al, Supplementary Information_R3_v07.23.2025.docx]

**Supplementary Table 1.** List of primer sequences

| Oligo name | 5'>3' | Usage |
| --- | --- | --- |
| GAPDH_qRT_F | TCAAGGCTGAGAACGGGAAG | qRT PCR |
| GAPDH_qRT_R | CGCCCCACTTGATTTTGGAG |  |
| STK11_qRT_F | GGCTTCAAGGTGGACATCTG | qRT PCR |
| STK11_qRT_R | CCTTCCCGATGTTCTCAAAC |  |
| TP53_qRT_F | CCATCCTCACCATCATCACA | qRT PCR |
| TP53_qRT_R | GTGAGGCTCCCCTTTCTTG |  |
| TP53AIP1_qRT_F | AGACCAGAACCTCTCGGTGATG | qRT PCR |
| TP53AIP1_qRT_R | ACCACGGTGAGAGCAGAGTCTG |  |
| TP53AIP1_F | GTCACTGGGTCCTGGTGAGT | Sanger sequencing |
| TP53AIP1_R | GGACTGGCCCTAACAACAAA |  |
| STK11_Promoter_F1 | GCACAGGAGGGTTCAATATTTTC | STK11 promoter sequencing  (Hearle NCM et al, 2005; PMID: 15774015) |
| STK11_Promoter_R1 | TTGCGGACCTGGAAGGAG |  |
| STK11_Promoter_F2 | ACTGGAATTGGCCACTTTGT |  |
| STK11_Promoter_R2 | GATACAGCGCGCTCATTG |  |
| STK11_Promoter_F3 | GTCTCCCCATGCCTGCTTC |  |
| STK11_Promoter_R3 | GGCCCAGCCCATCCAAGG |  |


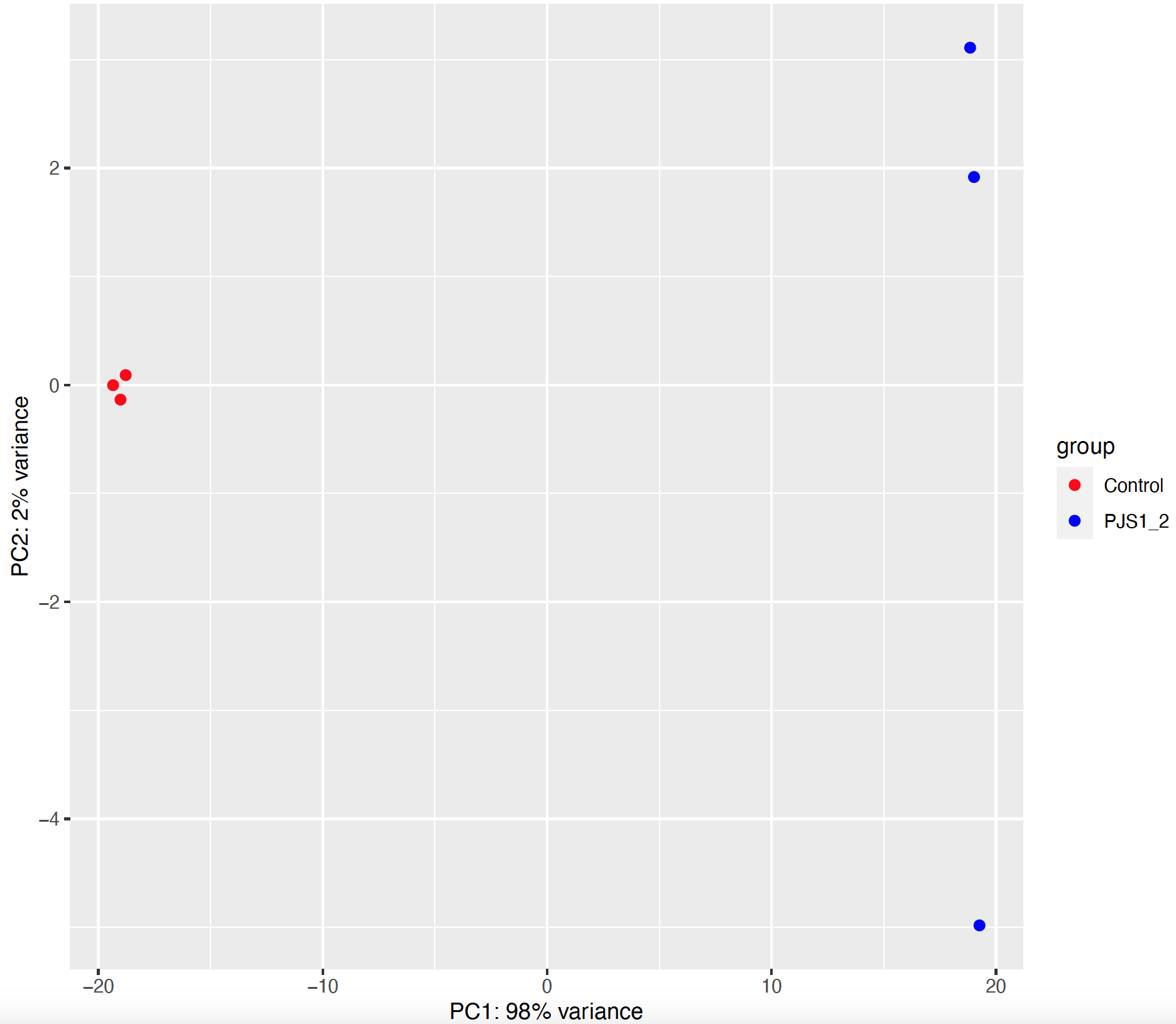


**Supplementary Figure 1:** Principal component analysis of RNA-seq.


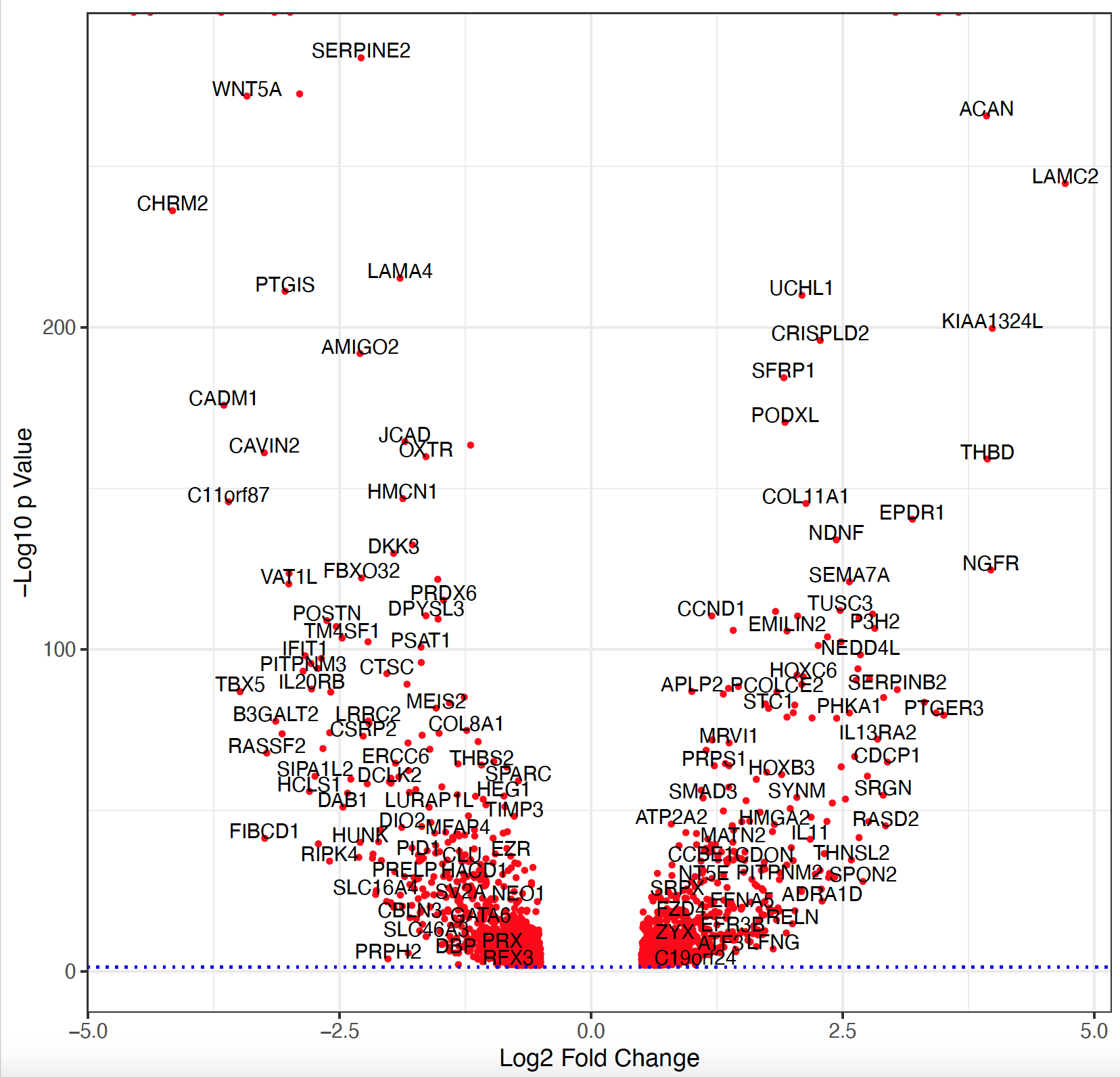


**Supplementary Figure 2**: Volcano plot of differentially expressed protein coding genes obtained from RNA-seq analysis of PJS1-2 versus healthy matched control.


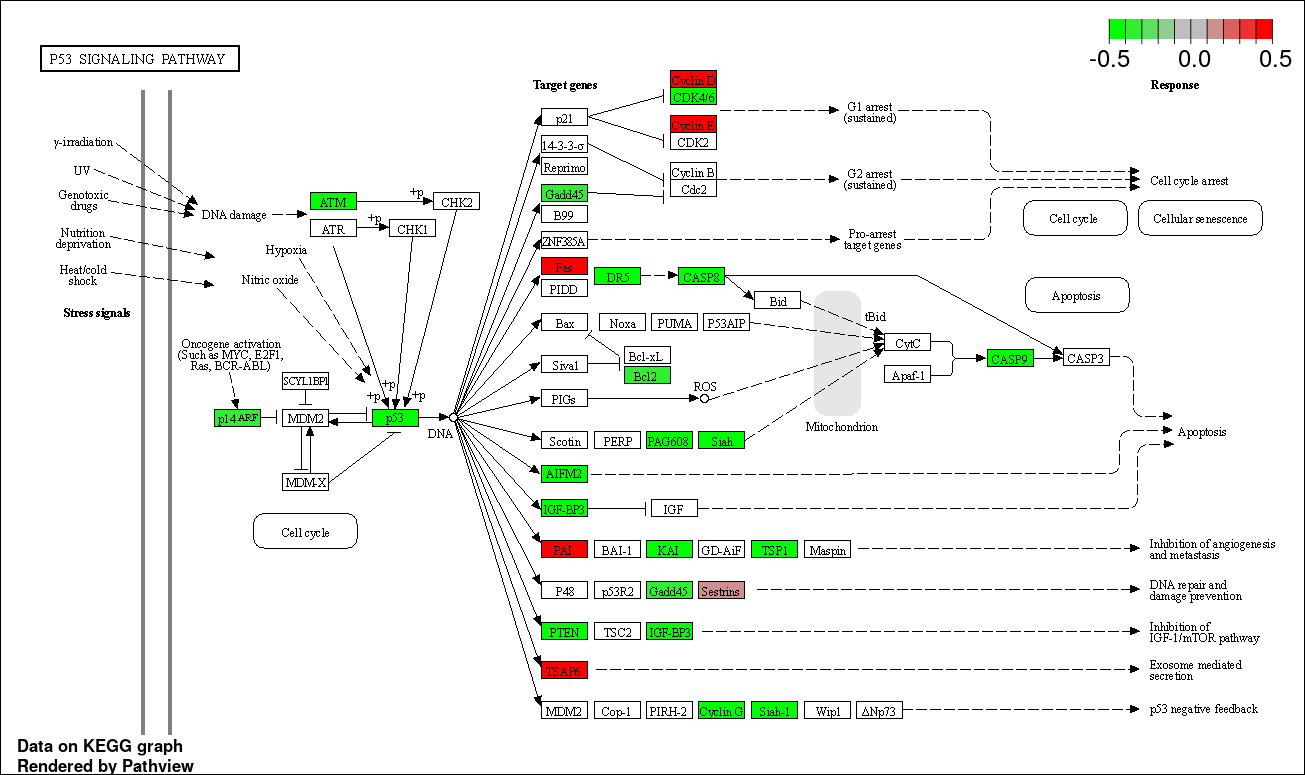


**Supplementary Figure 3:** Sketch of human p53 signaling pathway obtained from KEGG. Differentially expressed genes in primary dermal fibroblasts from PJS1-2 are rendered on a KEGG graph using Pathview. Note that P53AIP (KEGG entry=K13773) highlighted with a light green oval shape is alias to TP53AIP1 in this pathway.


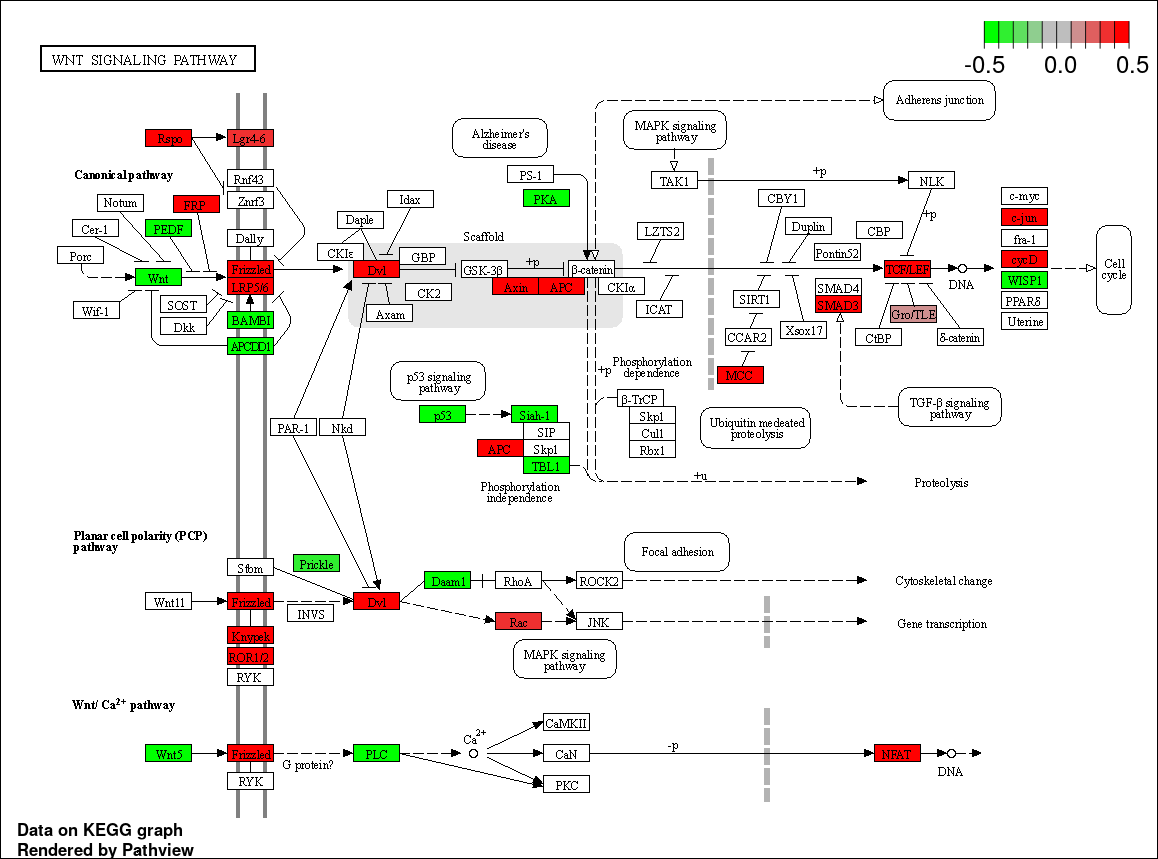


**Supplementary Figure 4:** Sketch of human Wnt signaling pathway obtained from KEGG. Differentially expressed genes in primary dermal fibroblasts from PJS1-2 are rendered on a KEGG graph using Pathview.


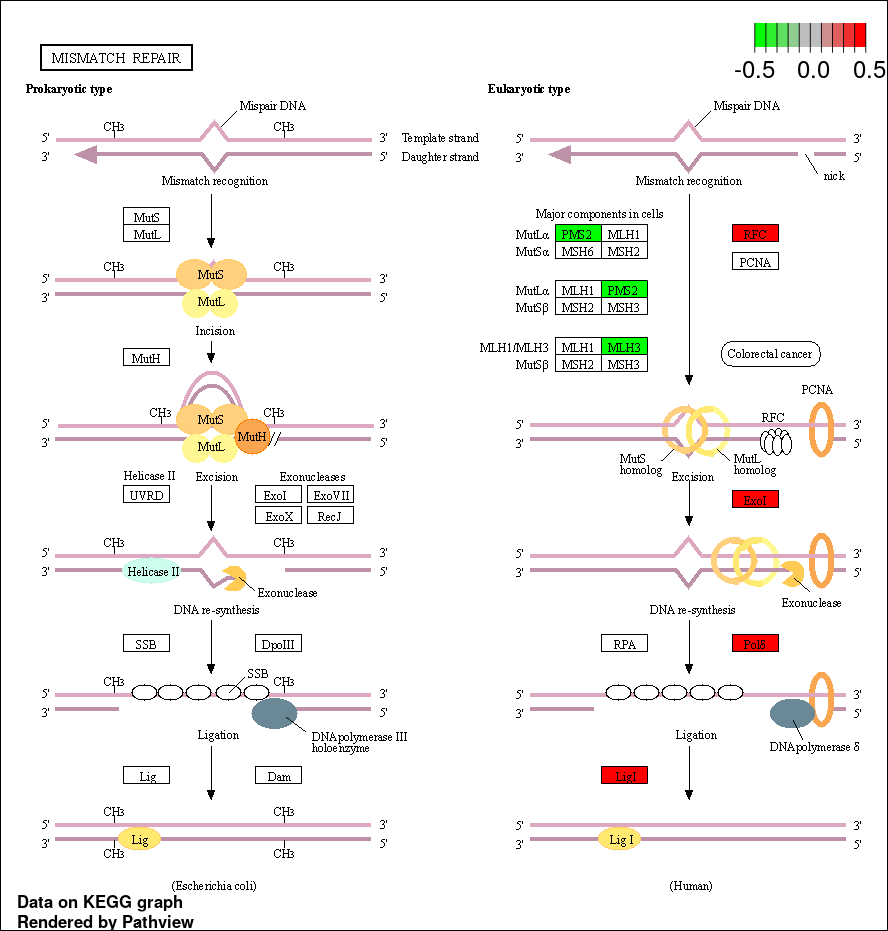


MISMATCH REPAIR

**Supplementary Figure 5:** Sketch of the eukaryotic DNA mismatch repair pathway obtained from KEGG. Differentially expressed genes in primary dermal fibroblasts from PJS1-2 are rendered on a KEGG graph using Pathview.
