# Supplementary material for: Genetic Investigation and Transcriptome Profiling in a Nuclear Family With Peutz–Jeghers Syndrome
Source: Hum Mutat. 2025 Aug 15;2025:5530710. doi: 10.1155/humu/5530710 (PMC12373473; doi:10.1155/humu/5530710)
Supplement: Supporting Information — Additional supporting information can be found online in the Supporting Information section. Table S1: List of primer sequences. Figure S1: Principal component analysis of RNA-seq. Figure S2: Volcano plot of differentially expressed protein coding genes obtained from RNA-seq analysis of PJS1-2 versus healthy matched control. Figure S3: Sketch of human p53 signaling pathway obtained from KEGG. Figure S4: Sketch of human Wnt signaling pathway obtained from KEGG. Figure S5: Sketch of the eukaryotic DNA mismatch repair pathway obtained from KEGG. Table S2: Differentially expressed genes in dermal fibroblasts from PJS1-2 versus a healthy control individual; n = 3 triplicates per genotype. Table S3: Filtered exome sequencing data showing 134 rare variants (MAF < 1% in public databases) present in both siblings (PJS1-1 and PJS1-2) and with mucocutaneous pigmentation that were absent from the mother (PJS1-6), who did not present with pigmentation. [file 5530710.f1.zip › Khan et al, Supplementary Table 2_R3_DEG.pdf]

Ensembl ID	Gene Symbol	Location	Strand	Biotype	Log2 Fold Change	LFC Standard Error	Wald Statistic	p Value	FDR Adj p Value	Significant	Status	Mean	Mean_Control	Mean_PJS1_2	Control_1	Control_2	Control_3	PJS1_2_1	PJS1_2_2	PJS1_2_3
ENSG00000115380	EFEMP1	chr2:55865967-55924139	-	protein_coding	-4.546449864	0.077078242	-58.98486727	0	0	Yes	OK	8711.559301	16711.59297	711.5256357	16751.7984	16876.5491	16506.43139	756.743191	649.302069	728.5316472
ENSG00000121898	CPXM2	chr10:123706207-123940267	-	protein_coding	-4.381309723	0.099192182	-44.16990983	0	0	Yes	OK	2154.573956	4111.599786	197.5481266	4040.386737	4110.902971	4183.509649	200.6934497	199.5416115	192.4093186
ENSG00000181072	CHRM2	chr7:136868669-137020255	+	protein_coding	-4.160252195	0.126567849	-32.86973927	5.9508E-237	6.9964E-234	Yes	OK	1394.330171	2643.425232	145.2351092	2721.248255	2600.02409	2609.003351	174.9163093	115.6074416	145.1815768
ENSG00000139329	LUM	chr12:91102629-91111831	-	protein_coding	-3.67585017	0.086858636	-42.31991587	0	0	Yes	OK	16855.59954	31268.31907	2442.880014	31496.5318	31377.94539	30930.48003	2771.042585	2199.708717	2357.888741
ENSG00000182985	CADM1	chr11:115169218-115504957	-	protein_coding	-3.649815693	0.128844631	-28.32726252	1.5955E-176	1.1419E-173	Yes	OK	988.7192067	1833.814365	143.6240484	1871.580235	1882.196571	1747.666288	165.7101878	115.6074416	149.5545158
ENSG00000185742	C11orf87	chr11:109422120-109429114	+	protein_coding	-3.602798986	0.139749811	-25.78034966	1.473E-146	7.3472E-144	Yes	OK	926.3107071	1713.366936	139.2544785	1761.301939	1644.521239	1734.277629	123.3620287	120.3584323	174.0429745
ENSG00000089225	TBX5	chr12:114353931-114408442	-	protein_coding	-3.488251101	0.175849425	-19.83657947	1.43924E-87	2.5473E-85	Yes	OK	333.4410352	612.7814276	54.10064275	602.8546886	586.5859212	648.9036729	46.03060772	50.67723465	65.59408589
ENSG00000114251	WNT5A	chr3:55465715-55490539	-	protein_coding	-3.420534551	0.096976015	-35.27196434	1.5805E-272	2.365E-269	Yes	OK	5968.31286	10921.20759	1015.418131	11059.33775	10758.60999	10945.67502	1028.323777	867.8476435	1150.082973
ENSG00000168497	CAVIN2	chr2:191834302-191847255	-	protein_coding	-3.24842474	0.119857417	-27.1024091	9.2232E-162	5.6228E-159	Yes	OK	1212.760293	2192.790203	232.7303841	2249.677252	2153.482557	2175.210799	247.6446696	258.137164	192.4093186
ENSG00000130720	FIBCD1	chr9:130902438-130939286	-	protein_coding	-3.243486239	0.238714747	-13.58728894	4.76402E-42	3.3227E-40	Yes	OK	260.8524297	469.818535	51.88632452	504.1293563	446.5415335	458.7847151	28.53897679	80.76684273	46.35315403
ENSG00000101265	RASSF2	chr20:4780023-4823645	-	protein_coding	-3.223782108	0.18427093	-17.49479478	1.56977E-68	1.87235E-66	Yes	OK	732.1978711	1325.627792	138.7679499	1404.210311	1282.006338	1290.666728	154.6628419	82.35050631	179.2905014
ENSG00000115461	IGFBP5	chr2:216672105-216695525	-	protein_coding	-3.146695019	0.078984426	-39.83943659	0	0	Yes	OK	23977.22549	43083.89687	4870.554104	43435.99539	42771.15652	43044.53869	4646.329544	5436.71708	4528.61569
ENSG00000162630	B3GALT2	chr1:193179045-193186654	-	protein_coding	-3.134258533	0.167204534	-18.74505711	2.12444E-78	2.98874E-76	Yes	OK	346.8511598	624.1843431	69.51797644	649.0665462	620.9968279	602.4896551	85.61693037	53.84456182	69.09243713
ENSG00000168952	STXBP6	chr14:24809656-25050297	-	protein_coding	-3.071156224	0.168168563	-18.26236823	1.64978E-74	2.20775E-72	Yes	OK	393.9950641	705.9974246	81.99270371	754.0934955	665.0107784	698.8879999	81.01386959	61.76287974	103.2013618
ENSG00000124212	PTGIS	chr20:49503874-49568146	-	protein_coding	-3.041937807	0.097911009	-31.06839416	6.4396E-212	6.6247E-209	Yes	OK	4141.446451	7381.967488	900.9254146	7239.50761	7246.296749	7660.098103	834.0746119	1040.466974	828.2346578
ENSG00000171724	VAT1L	chr16:77788530-77980107	+	protein_coding	-3.004920745	0.128449765	-23.39374265	4.9487E-121	1.697E-118	Yes	OK	573.1397427	1019.935239	126.3442465	1005.107904	1052.333542	1002.364271	132.5681502	118.7747687	127.6898205
ENSG00000183496	MEX3B	chr15:82041778-82046141	-	protein_coding	-3.001546422	0.126569833	-23.71454828	2.5523E-124	9.548E-122	Yes	OK	609.4452689	1083.842859	135.0476786	1031.364641	1103.549775	1116.614161	139.0124353	131.4440774	134.686523
ENSG00000112902	SEMA5A	chr5:9035026-9546075	-	protein_coding	-2.990095564	0.06928135	-43.15873718	0	0	Yes	OK	7448.862087	13233.84442	1663.879757	13773.23412	12813.66135	13114.63778	1676.434733	1618.504182	1696.700355
ENSG00000136205	TNS3	chr7:47275154-47582558	-	protein_coding	-2.896951408	0.08202859	-35.31636226	3.2939E-273	5.4218E-270	Yes	OK	6215.205883	10955.0211	1475.390666	10929.10434	11025.09446	10910.86451	1370.791498	1635.924481	1419.456019
ENSG00000091622	PITPNM3	chr17:6451264-6556494	-	protein_coding	-2.862059142	0.139200127	-20.56075096	6.16712E-94	1.30142E-91	Yes	OK	459.9936848	808.6217081	111.3656614	820.2604735	805.8554198	799.749231	98.50550053	114.023778	121.5677058
ENSG00000185745	IFIT1	chr10:89392546-89406486	+	protein_coding	-2.84171726	0.134748088	-21.08911012	1.00127E-98	2.28902E-96	Yes	OK	739.0326564	1299.459783	178.6055294	1382.154652	1245.19467	1271.030028	204.3758983	142.5297225	188.9109674
ENSG00000180353	HCLS1	chr3:121631399-121660927	-	protein_coding	-2.799434084	0.176571338	-15.85440832	1.31056E-56	1.25418E-54	Yes	OK	244.9420041	427.4785602	62.40544809	436.9121088	446.5415335	398.9820382	62.6016265	71.26486123	53.34985652
ENSG00000144218	AFF3	chr2:99545419-100142739	-	protein_coding	-2.784232841	0.133680096	-20.82757967	2.43457E-96	5.34307E-94	Yes	OK	645.4693415	1127.144931	163.7937523	1147.944555	1049.932781	1183.557456	184.1224309	164.7010126	142.5578133
ENSG00000174564	IL20RB	chr3:136946230-137011085	+	protein_coding	-2.778672102	0.139393152	-19.93406471	2.06122E-88	3.76974E-86	Yes	OK	549.0826851	958.5880943	139.577276	941.0414651	1032.327201	902.3956167	158.3452906	136.1950681	124.1914693
ENSG00000116991	SIPA1L2	chr1:232397965-232561558	-	protein_coding	-2.744748211	0.166132762	-16.5214144	2.57293E-61	2.6804E-59	Yes	OK	298.2551203	519.8538686	76.65637203	477.872619	565.7793265	515.9096602	85.61693037	66.51387048	77.83831525
ENSG00000075643	MOCOS	chr18:36187519-36272157	+	protein_coding	-2.71775836	0.131573498	-20.655819	8.65412E-95	1.8743E-92	Yes	OK	844.9237963	1464.917945	224.9296476	1349.596298	1503.676598	1541.48094	231.0736508	256.5535004	187.1617917
ENSG00000206262	FOXL2NB	chr3:138947234-138953451	+	protein_coding	-2.710525826	0.203870935	-13.29530284	2.46473E-40	1.63587E-38	Yes	OK	183.971552	317.7083801	50.23472384	306.6786918	338.5072915	307.939157	39.58632264	66.51387048	44.6039784
ENSG00000150051	MKX	chr10:27672875-27746060	-	protein_coding	-2.680111521	0.12762229	-21.00034037	6.51174E-98	1.46826E-95	Yes	OK	1576.783017	2731.2442	422.3218349	2812.6217	2648.039309	2733.071591	483.3213811	326.2346981	457.4094256
ENSG00000151012	SLC7A11	chr4:138164097-138242349	-	protein_coding	-2.665145755	0.150788248	-17.67475771	6.56217E-70	8.00099E-68	Yes	OK	4100.936196	7088.225509	1113.646883	7188.044405	6949.402647	7127.229475	1397.48925	785.4971371	1157.954263
ENSG00000133110	POSTN	chr13:37562583-37598844	-	protein_coding	-2.626025788	0.118034954	-22.24786557	1.1829E-109	3.357E-107	Yes	OK	36663.8035	63109.61183	10217.99517	63900.49645	63582.5528	61845.78624	11831.70741	7880.309989	10941.96811
ENSG00000183421	RIPK4	chr21:41739369-41767106	-	protein_coding	-2.599429717	0.210728843	-12.33542443	5.83805E-35	3.14034E-33	Yes	OK	172.422782	294.6631013	50.18246262	287.7738409	309.6981603	286.5173026	50.6336685	64.9302069	34.98351247
ENSG00000145390	USP53	chr4:119212587-119295517	+	protein_coding	-2.598445704	0.141969242	-18.30287795	7.8491E-75	1.06774E-72	Yes	OK	1935.590089	3325.913301	545.2668769	3402.873155	3364.26632	3210.600429	641.6666717	395.9158957	598.2180633
ENSG00000080823	MOK	chr14:102224500-102305200	-	protein_coding	-2.587515898	0.13053506	-19.82238258	1.90855E-87	3.27237E-85	Yes	OK	590.0398839	1011.231398	168.8483699	916.8852668	1055.534557	1061.27437	174.9163093	174.2029941	157.4258061
ENSG00000205221	VIT	chr2:36696690-36814792	+	protein_coding	-2.533094623	0.114851347	-22.05541929	8.4743E-108	2.3642E-105	Yes	OK	891.2264305	1520.975459	261.4774025	1493.483218	1594.905513	1474.537645	291.834053	240.7168646	251.8812898
ENSG00000169908	TM4SF1	chr3:149369022-149377865	-	protein_coding	-2.474791654	0.11415655	-21.67892814	3.2435E-104	8.3418E-102	Yes	OK	1220.111565	2067.295867	372.9272622	2071.131439	2120.672157	2010.084005	412.4342452	387.9975778	318.3499635
ENSG00000173406	DAB1	chr1:56994778-58546734	-	protein_coding	-2.466689404	0.162981312	-15.13479906	9.54719E-52	8.18472E-50	Yes	OK	362.1404705	612.6296945	111.6512465	638.5638513	613.7945451	585.530687	128.8857016	120.3584323	85.70960556
ENSG00000066468	FGFR2	chr10:121478334-121598458	-	protein_coding	-2.420098552	0.153418928	-15.77444573	4.66475E-56	4.41275E-54	Yes	OK	347.2396421	585.3450381	109.1342461	524.0844767	624.9980962	606.9525414	119.6795801	104.5217965	103.2013618
ENSG00000153993	SEMA3D	chr7:84995553-85186855	-	protein_coding	-2.388725449	0.145687617	-16.3962147	2.03531E-60	2.08082E-58	Yes	OK	704.7010199	1186.136483	223.2655564	1231.966114	1180.374125	1146.069211	254.0889546	164.7010126	251.006702
ENSG00000116675	DNAJC6	chr1:65248219-65415869	+	protein_coding	-2.308850278	0.183957661	-12.55098733	3.92584E-36	2.23596E-34	Yes	OK	520.6776026	868.6306162	172.7245889	871.7236786	913.8896617	820.2785082	229.2324265	114.023778	174.9175624
ENSG00000139211	AMIGO2	chr12:47075707-47079951	-	protein_coding	-2.296971903	0.077593475	-29.60264251	1.3817E-192	1.083E-189	Yes	OK	3558.014329	5914.748001	1201.280657	6098.914942	5950.686099	5694.642962	1229.017226	1143.405107	1231.419639
ENSG00000142149	HUNK	chr21:31873315-32044633	+	protein_coding	-2.295374199	0.171634576	-13.37361188	8.62488E-41	5.77096E-39	Yes	OK	389.1437571	644.3702704	133.9172439	704.7308293	601.7907405	626.5892413	111.3940707	175.7866577	114.5710033
ENSG00000135919	SERPINE2	chr2:223975112-224039319	-	protein_coding	-2.286519321	0.063445477	-36.0391227	2.0418E-284	3.7342E-281	Yes	OK	112471.8985	186685.4403	38258.35661	187174.8278	186377.4732	186504.0199	39747.42977	35119.32362	39908.31644
ENSG00000156804	FBXO32	chr8:123497889-123541206	-	protein_coding	-2.281673013	0.096781002	-23.57562905	6.8553E-123	2.5075E-120	Yes	OK	1469.650173	2436.013233	503.2871132	2521.697051	2379.954338	2406.388311	498.0511756	544.7802725	467.0298915
ENSG00000175183	CSRP2	chr12:76858715-76879060	-	protein_coding	-2.264976698	0.124660552	-18.16915348	9.05851E-74	1.19282E-71	Yes	OK	669.451304	1110.505058	228.3975501	1097.531619	1107.551044	1126.432511	247.6446696	190.03963	247.5083507
ENSG00000021826	CPS1	chr2:210477682-210679107	+	protein_coding	-2.225052209	0.137388992	-16.19527278	5.44557E-59	5.39964E-57	Yes	OK	374.0679343	616.7801195	131.3557492	617.5584615	641.003169	591.7787279	131.6475381	125.1094231	137.3102865
ENSG00000171345	KRT19	chr17:41523617-41528308	-	protein_coding	-2.217388183	0.102864743	-21.55634784	4.6163E-103	1.1497E-100	Yes	OK	1833.492053	3016.671333	650.3127738	2842.029246	3233.024723	2974.96003	580.9062695	690.4773222	679.5547298
ENSG00000163827	LRRC2	chr3:46515423-46580099	-	protein_coding	-2.215865928	0.118158956	-18.75326262	1.82071E-78	2.58352E-76	Yes	OK	664.7749936	1093.644133	235.9058543	1119.587279	1033.127455	1128.217665	247.6446696	242.3005282	217.7723651
ENSG00000155792	DEPTOR	chr8:119873717-120050913	+	protein_coding	-2.207718412	0.118291031	-18.66344724	9.81878E-78	1.36964E-75	Yes	OK	673.8524504	1106.587353	241.1175482	1158.44725	1064.337347	1096.977461	221.8675292	264.4718184	237.013297
ENSG00000111981	ULBP1	chr6:149964007-149973710	+	protein_coding	-2.173231723	0.173834766	-12.50170935	7.30632E-36	4.11856E-34	Yes	OK	300.4733128	493.1450537	107.801572	500.9785478	485.7539621	492.7026513	139.9330475	85.51783348	97.95383492
ENSG00000183770	FOXL2	chr3:138944224-138947140	-	protein_coding	-2.167905317	0.170240349	-12.73438017	3.80859E-37	2.23891E-35	Yes	OK	250.4574281	409.6379024	91.27695371	386.4991732	419.3329096	423.0816245	74.56958451	93.43615139	105.8251252
ENSG00000168679	SLC16A4	chr1:110362848-110391082	-	protein_coding	-2.141482707	0.209178235	-10.23759811	1.34535E-24	4.95401E-23	Yes	OK	177.1697035	290.4714552	63.86795186	317.1813867	271.2859854	282.9469936	69.96652374	41.17525316	80.46207869
ENSG00000100344	PNPLA3	chr22:43923739-43964488	+	protein_coding	-2.135315396	0.203307086	-10.50290689	8.37603E-26	3.19883E-24	Yes	OK	149.5283116	244.572737	54.48388629	242.6122527	253.6804052	237.425553	52.4748928	42.75891674	68.21784932
ENSG00000138735	PDE5A	chr4:119494395-119628991	-	protein_coding	-2.112283143	0.157601179	-13.40271156	5.82938E-41	3.93244E-39	Yes	OK	1127.863589	1834.796682	420.9304953	1724.542506	1943.816102	1836.031438	459.3854651	296.14509	507.2609308
ENSG00000198846	TOX	chr8:58805418-59119208	-	protein_coding	-2.091213491	0.149245252	-14.01192647	1.31781E-44	9.90464E-43	Yes	OK	313.1590014	506.0582823	120.2597204	489.4255834	521.7653761	506.9838875	118.7589679	136.1950681	105.8251252
ENSG00000133216	EPHB2	chr1:22710839-22921500	+	protein_coding	-2.084798609	0.168540889	-12.36969036	3.81288E-35	2.06448E-33	Yes	OK	592.2664312	957.0491276	227.4837349	924.2371532	973.1084314	973.8017981	158.3452906	273.9737999	250.1321142
ENSG00000111341	MGP	chr12:14881181-14885926	-	protein_coding	-2.032977791	0.208651473	-9.743414521	1.96827E-22	6.46662E-21	Yes	OK	176.5101547	284.4565792	68.56373016	323.4830036	242.4768542	287.4098799	87.45815467	57.01188899	61.22114683
ENSG00000109861	CTSC	chr11:88293592-88337787	-	protein_coding	-2.030514205	0.099157709	-20.47762327	3.40891E-93	7.10261E-91	Yes	OK	1011.299477	1625.932431	396.6665232	1622.666366	1656.525044	1598.605885	396.7838386	376.9119327	416.3037984
ENSG00000112619	PRPH2	chr6:42696600-42722574	-	protein_coding	-2.019283083	0.529282705	-3.815131431	0.00013611	0.000827013	Yes	OK	141.6686042	226.6046007	56.73260783	222.6571324	215.2682303	241.8884393	44.18938341	99.77080573	26.23763435
ENSG00000170390	DCLK2	chr4:150078274-150257457	+	protein_coding	-2.005979845	0.123212628	-16.2806352	1.35456E-59	1.35951E-57	Yes	OK	848.2929576	1360.548936	336.0369792	1308.635787	1317.217498	1455.793522	345.2295579	285.0594449	377.8219347
ENSG00000120875	DUSP4	chr8:29333064-29350668	-	protein_coding	-1.991696246	0.206230103	-9.657640766	4.5625E-22	1.46964E-20	Yes	OK	339.1615616	539.6052441	138.7178791	521.9839377	550.5745072	546.2572873	105.8703978	202.7089386	107.5743009
ENSG00000013588	GPRC5A	chr12:12890782-12917937	+	protein_coding	-1.989699235	0.12105561	-16.43624139	1.05247E-60	1.08273E-58	Yes	OK	496.1871326	792.2634066	200.1108586	790.8529277	813.8579562	772.0793358	196.0903889	207.4599294	196.7822577
ENSG00000143196	DPT	chr1:168695459-168729264	-	protein_coding	-1.98886134	0.122628664	-16.21856812	3.72784E-59	3.71881E-57	Yes	OK	581.7735709	930.9600811	232.5870606	942.0917346	947.5003148	903.288194	234.7560994	205.8762658	257.1288167
ENSG00000050165	DKK3	chr11:11963106-12009769	-	protein_coding	-1.9636675	0.080774652	-24.3104422	1.5203E-130	6.2559E-128	Yes	OK	19456.12399	30967.67645	7944.571522	30537.63576	31185.88452	31179.50908	7215.758067	8821.006157	7796.950342
ENSG00000108448	TRIM16L	chr17:18697998-18736118	+	protein_coding	-1.963468919	0.210838975	-9.312646885	1.24686E-20	3.80765E-19	Yes	OK	144.4048754	228.4537778	60.35597304	233.1598273	227.272035	224.9294712	46.95121988	80.76684273	53.34985652
ENSG00000187123	LYPD6	chr2:149329985-149474148	+	protein_coding	-1.961311743	0.210181565	-9.33151175	1.04371E-20	3.20514E-19	Yes	OK	499.5352612	796.8490486	202.2214738	799.2550836	767.4432448	823.8488172	287.2309922	123.5257595	195.9076698
ENSG00000111907	TPD52L1	chr6:125119049-125264407	+	protein_coding	-1.952232718	0.166728936	-11.70902163	1.14594E-31	5.5971E-30	Yes	OK	372.4144975	593.1458553	151.6831397	620.7092699	609.7932769	548.9350191	133.4887624	128.2767502	193.2839064
ENSG00000225830	ERCC6	chr10:49455368-49539538	-	protein_coding	-1.944563176	0.113873708	-17.07648949	2.22085E-65	2.52105E-63	Yes	OK	1355.559216	2154.994653	556.1237797	2207.666473	2122.272665	2135.044822	606.6834098	460.8461026	600.8418267
ENSG00000174348	PODN	chr1:53062052-53085502	+	protein_coding	-1.91165428	0.115927119	-16.49013883	4.31956E-61	4.47169E-59	Yes	OK	5871.895446	9274.024682	2469.76621	8914.687451	9222.92325	9684.463345	2103.598773	2991.540508	2314.15935
ENSG00000112769	LAMA4	chr6:112108760-112254939	-	protein_coding	-1.899033202	0.060541944	-31.36723179	5.6644E-216	6.2157E-213	Yes	OK	12371.4648	19513.6126	5229.31699	19670.49732	19632.62265	19237.71783	5332.185599	5056.63782	5299.127552
ENSG00000137965	IFI44	chr1:78649796-78664078	+	protein_coding	-1.896115761	0.185916778	-10.19873398	2.00874E-24	7.33123E-23	Yes	OK	168.2859884	265.417741	71.15423566	240.5117137	296.894102	258.8474074	73.64897236	68.09753407	71.71620057
ENSG00000211448	DIO2	chr14:80197527-80387757	-	protein_coding	-1.881746305	0.133017769	-14.1465785	1.96067E-45	1.49411E-43	Yes	OK	364.2353028	572.5235722	155.9470333	569.2460648	576.1826239	572.142028	147.2979447	163.117349	157.4258061
ENSG00000064205	WISP2	chr20:44714844-44728509	+	protein_coding	-1.876074407	0.199972316	-9.381670665	6.49355E-21	2.02432E-19	Yes	OK	283.5491263	444.262992	122.8352607	377.0467477	496.9575131	458.7847151	85.61693037	155.1990311	127.6898205
ENSG00000143341	HMCN1	chr1:185734551-186190949	+	protein_coding	-1.871052677	0.072334566	-25.86664696	1.581E-147	8.1323E-145	Yes	OK	7402.589148	11628.78626	3176.39204	12068.64674	11683.70321	11134.00883	3341.822121	3032.715761	3154.638237
ENSG00000188783	PRELP	chr1:203475828-203491352	+	protein_coding	-1.854006922	0.162417791	-11.41504824	3.51709E-30	1.60364E-28	Yes	OK	289.4442924	452.5217149	126.3668699	453.7164207	436.1382361	467.7104878	99.42611268	144.113386	135.5611108
ENSG00000165757	JCAD	chr10:30012800-30115494	-	protein_coding	-1.84994521	0.067522952	-27.39727963	2.955E-165	1.9456E-162	Yes	OK	4144.317298	6488.52332	1800.111276	6481.213037	6490.857309	6493.499616	1792.431865	1805.376485	1802.52548
ENSG00000106829	TLE4	chr9:79571773-79726882	+	protein_coding	-1.829679207	0.091010365	-20.10407494	6.79761E-90	1.30103E-87	Yes	OK	1562.622095	2437.003082	688.2411083	2397.765251	2479.18579	2434.058206	671.1262606	741.1545568	652.4425076
ENSG00000213626	LBH	chr2:30231531-30323730	+	protein_coding	-1.820144286	0.101683848	-17.90003357	1.17851E-71	1.46956E-69	Yes	OK	12989.59564	20241.40465	5737.786635	19839.59071	20493.69558	20390.92766	5111.238682	6790.749444	5311.371781
ENSG00000136869	TLR4	chr9:117704175-117724730	+	protein_coding	-1.817370484	0.383498617	-4.738923179	2.14857E-06	1.85159E-05	Yes	OK	413.4794469	644.9859881	181.9729056	696.3286734	605.7920087	632.8372821	216.3438563	93.43615139	236.1387092
ENSG00000170153	RNF150	chr4:140859807-141212877	-	protein_coding	-1.815807071	0.108326485	-16.76235565	4.6003E-63	4.94909E-61	Yes	OK	751.2275189	1171.152036	331.303002	1184.703987	1206.782496	1121.969624	341.5471093	310.3980623	341.9638344

ENSG00000182463	TSHZ2	chr20:52972407-53495330	+	protein_coding	-1.80726149	0.114323555	-15.80830381	2.72711E-56	2.59469E-54	Yes	OK	601.0733859	933.9007403	268.2460315	930.5387702	917.89093	953.2725209	255.0095668	288.2267721	261.5017557
ENSG00000105894	PTN	chr7:137227341-137343865	-	protein_coding	-1.800716143	0.165105383	-10.90646535	1.0735E-27	4.3737E-26	Yes	OK	995.1190426	1548.900415	441.3376698	1484.030793	1537.287251	1625.383203	545.9230076	304.0634079	474.026594
ENSG00000139899	CBLN3	chr14:24426532-24430954	-	protein_coding	-1.800531177	0.212138988	-8.487507142	2.11117E-17	5.34613E-16	Yes	OK	127.2265191	196.9699535	57.48308461	218.4560544	191.260621	181.1931852	57.99856573	68.09753407	46.35315403
ENSG00000170801	HTRA3	chr4:8269765-8307111	+	protein_coding	-1.78224569	0.188500291	-9.454869674	3.23425E-21	1.01984E-19	Yes	OK	170.2464237	264.4041924	76.08865494	251.0144087	244.0773615	298.1208071	86.53754252	66.51387048	75.21455182
ENSG00000163682	RPL9	chr4:39452521-39458949	-	protein_coding	-1.779752841	0.136183435	-13.06879094	4.96484E-39	3.17981E-37	Yes	OK	359.7687668	558.1073254	161.4302081	576.5979153	527.3671516	570.3568735	166.6308	148.8643768	168.7954477
ENSG00000139112	GABARAPL1	chr12:10212458-10223130	+	protein_coding	-1.775227501	0.072291508	-24.55651514	3.6847E-133	1.596E-130	Yes	OK	3298.927407	5107.067147	1490.787667	5046.54491	5120.022816	5154.633716	1529.136789	1453.803169	1489.423044
ENSG00000204642	HLA-F	chr6:29722775-29738528	+	protein_coding	-1.759366118	0.154942311	-11.35497532	7.00597E-30	3.12516E-28	Yes	OK	241.652396	372.6694501	110.6353418	349.7397409	388.9232711	379.3453384	110.4734585	115.6074416	105.8251252
ENSG00000110852	CLEC2B	chr12:9852984-9870136	-	protein_coding	-1.758728368	0.165509949	-10.6261187	2.25295E-26	8.8294E-25	Yes	OK	433.424062	671.3003463	195.5477777	712.0827158	653.8072274	648.0110957	234.7560994	139.3623953	212.5248383
ENSG00000171160	MORN4	chr10:97614553-97633500	-	protein_coding	-1.749092371	0.156110292	-11.2042092	3.8881E-29	1.69307E-27	Yes	OK	258.2346526	399.0988179	117.3704873	384.3986342	412.1306268	400.7671928	131.6475381	99.77080573	120.693118
ENSG00000198795	ZNF521	chr18:25061926-25352190	-	protein_coding	-1.742175832	0.109322082	-15.93617507	3.55455E-57	3.44165E-55	Yes	OK	983.8936975	1516.754274	451.0331207	1600.610706	1526.883953	1422.768163	482.4007689	405.4178772	465.2807159
ENSG00000172197	MBOAT1	chr6:20102145-20212399	-	protein_coding	-1.737757762	0.207305883	-8.382578131	5.17808E-17	1.27401E-15	Yes	OK	160.0986886	247.1533179	73.04405935	230.0090188	278.4882682	232.9626666	93.90243976	57.01188899	68.21784932
ENSG00000088899	LZTS3	chr20:3162617-3173592	-	protein_coding	-1.71815792	0.172862916	-9.939424593	2.80442E-23	9.61684E-22	Yes	OK	210.4230159	321.7797286	99.06630317	322.4327341	307.2973994	335.6090523	81.01386959	115.6074416	100.5775984
ENSG00000153823	PID1	chr2:228850526-229271285	-	protein_coding	-1.717326918	0.135436631	-12.67992943	7.64006E-37	4.44365E-35	Yes	OK	690.5775129	1061.512584	319.6424414	1043.967875	1050.733035	1089.836843	341.5471093	251.8025097	365.5777053
ENSG00000119922	IFIT2	chr10:89283694-89309276	+	protein_coding	-1.715838815	0.151988731	-11.28925019	1.48296E-29	6.52662E-28	Yes	OK	363.3924491	559.0400005	167.7448977	558.7433699	541.7717172	576.6049143	176.7575337	131.4440774	195.033082
ENSG00000143367	TUFT1	chr1:151540305-151583583	+	protein_coding	-1.712336847	0.138902048	-12.32765733	6.42887E-35	3.43569E-33	Yes	OK	318.9987456	488.15428	149.8432113	471.571002	492.1559912	500.7358467	141.7742718	159.9500219	147.8053402
ENSG00000197457	STMN3	chr20:63639705-63657682	-	protein_coding	-1.704765297	0.23297114	-7.317495634	2.52641E-13	4.89233E-12	Yes	OK	139.6232325	212.1079426	67.13852235	222.6571324	220.8700058	192.7966896	49.71305634	96.60347856	55.09903214
ENSG00000185339	TCN2	chr22:30606838-30627278	+	protein_coding	-1.699921624	0.19119423	-8.891071764	6.05223E-19	1.65481E-17	Yes	OK	195.8026314	297.8690544	93.73620837	291.9749189	293.6930874	307.939157	71.80774805	121.9420959	87.45878118
ENSG00000196139	AKR1C3	chr10:5035354-5107686	+	protein_coding	-1.693296303	0.136697984	-12.38713446	3.06812E-35	1.67223E-33	Yes	OK	338.3721232	516.6482424	160.096004	508.3304343	495.3570058	546.2572873	154.6628419	164.7010126	160.9241574
ENSG00000135069	PSAT1	chr9:78297143-78330093	+	protein_coding	-1.690714612	0.079075314	-21.38106725	2.0049E-101	4.7827E-99	Yes	OK	5914.681258	9034.921327	2794.44119	9038.619251	9010.055781	9056.088949	2988.307053	2538.612723	2856.403793
ENSG00000134363	FST	chr5:53480409-53487134	+	protein_coding	-1.688470061	0.080939272	-20.86094954	1.2124E-96	2.69678E-94	Yes	OK	4066.185995	6206.70878	1925.663211	6340.476925	6332.407087	5947.242329	2056.647553	1917.816599	1802.52548
ENSG00000139174	PRICKLE1	chr12:42456757-42590355	-	protein_coding	-1.687937955	0.186955185	-9.028569917	1.7393E-19	4.95309E-18	Yes	OK	155.2911065	237.2645409	73.31767214	214.2549764	252.0798979	245.4587484	81.01386959	68.09753407	70.84161276
ENSG00000143631	FLG	chr1:152302175-152325203	-	protein_coding	-1.685039954	0.185385684	-9.089374758	9.96102E-20	2.88659E-18	Yes	OK	152.1520376	231.3908487	72.91322647	218.4560544	240.0760933	235.6403984	69.04591158	82.35050631	67.34326151
ENSG00000112414	ADGRG6	chr6:142301854-142446266	+	protein_coding	-1.684353106	0.118676233	-14.19284269	1.01461E-45	7.76766E-44	Yes	OK	667.6074252	1019.203834	316.0110167	951.54416	1063.537093	1042.530248	303.802011	296.14509	348.0859491
ENSG00000130707	ASS1	chr9:130444929-130501274	+	protein_coding	-1.67968544	0.092259941	-18.20601041	4.62442E-74	6.13855E-72	Yes	OK	1580.549495	2409.921946	751.1770443	2384.111748	2461.58021	2384.07388	712.5538076	728.4852482	812.4920772
ENSG00000154556	SORBS2	chr4:185585444-185956652	-	protein_coding	-1.675950916	0.211790997	-7.913230217	2.50795E-15	5.52622E-14	Yes	OK	222.1110939	336.2856028	107.936585	321.3824646	346.509828	340.9645159	83.77570606	155.1990311	84.83501775
ENSG00000136231	IGF2BP3	chr7:23310209-23470467	-	protein_coding	-1.673685543	0.157623654	-10.61823846	2.45143E-26	9.56175E-25	Yes	OK	295.5359715	450.2232831	140.4486199	487.3250444	477.7514256	385.5933792	148.2185569	131.4440774	141.6832255
ENSG00000122420	PTGFR	chr1:78303884-78539749	+	protein_coding	-1.667092127	0.133289066	-12.50734348	6.80626E-36	3.84986E-34	Yes	OK	879.8278693	1340.601799	419.0539398	1323.33956	1344.426122	1354.039714	496.2099513	334.153016	426.7988522
ENSG00000012171	SEMA3B	chr3:50267558-50277546	+	protein_coding	-1.656980225	0.163112927	-10.15848503	3.0376E-24	1.09888E-22	Yes	OK	518.0311686	784.3366767	251.7256604	768.7972684	751.4381719	832.7745899	197.0110011	324.6510345	233.5149458
ENSG00000113657	DPYSL3	chr5:147390811-147510056	-	protein_coding	-1.641502706	0.073274301	-22.40216124	3.7494E-111	1.1644E-108	Yes	OK	6531.115952	9888.388577	3173.843328	9757.003583	10010.37284	9897.789311	3062.876638	3415.962348	3042.690997
ENSG00000180914	OXTR	chr3:8750408-8769628	-	protein_coding	-1.641460575	0.06079625	-26.99937187	1.5032E-160	8.8367E-158	Yes	OK	14004.25883	21212.59698	6795.920686	21194.43835	21326.75962	21116.59298	6942.336257	6515.19198	6930.233821
ENSG00000139508	SLC46A3	chr13:28700064-28718970	-	protein_coding	-1.639705918	0.243183096	-6.742680492	1.55491E-11	2.50674E-10	Yes	OK	153.6400504	234.1446199	73.13548096	273.070068	224.0710204	205.2927714	83.77570606	41.17525316	94.45548368
ENSG00000104415	WISP1	chr8:133191039-133231690	+	protein_coding	-1.636026827	0.15966202	-10.24681277	1.2231E-24	4.51396E-23	Yes	OK	489.2777874	742.5391867	236.016388	768.7972684	701.0221924	757.7980995	283.5485436	172.6193305	251.8812898
ENSG00000138061	CYP1B1	chr2:38066973-38109902	-	protein_coding	-1.629584571	0.126121659	-12.92073533	3.4384E-38	2.15194E-36	Yes	OK	6358.255242	9613.844357	3102.666127	9852.578107	9575.835107	9413.119855	3705.463922	2402.417655	3200.116803
ENSG00000153714	LURAP1L	chr9:12775021-12822131	+	protein_coding	-1.609587795	0.106402193	-15.12739307	1.06845E-51	9.11226E-50	Yes	OK	789.9814291	1189.816742	390.1461162	1161.598058	1155.566262	1252.285905	405.9899601	392.7485686	371.69982
ENSG00000153885	KCTD15	chr19:33795933-33815763	+	protein_coding	-1.602772284	0.090827833	-17.64626799	1.08707E-69	1.31567E-67	Yes	OK	1383.313539	2080.615436	686.011641	2031.221198	2031.844003	2178.781108	688.6178915	704.7302944	664.686737
ENSG00000176595	KBTBD11	chr8:1973878-2006936	+	protein_coding	-1.600155014	0.17143255	-9.334020933	1.01929E-20	3.13598E-19	Yes	OK	197.6789131	297.6826117	97.67521445	324.5332731	294.4933411	274.0212209	91.14060329	93.43615139	108.4488887
ENSG00000135269	TES	chr7:116210493-116258783	+	protein_coding	-1.592419891	0.110679013	-14.38773127	6.17898E-47	4.86631E-45	Yes	OK	1633.629677	2456.207355	811.0519985	2464.982498	2376.753323	2526.886242	919.6915423	690.4773222	822.9871309
ENSG00000128165	ADM2	chr22:50481556-50486440	+	protein_coding	-1.585448067	0.143565582	-11.04337156	2.36008E-28	9.80982E-27	Yes	OK	1164.56765	1743.956728	585.1785717	1723.492237	1743.752691	1764.625256	503.5748485	749.0728547	502.8879918
ENSG00000103449	SALL1	chr16:51135975-51151367	-	protein_coding	-1.563963202	0.153097371	-10.21548045	1.6904E-24	6.2107E-23	Yes	OK	357.6784708	534.303461	181.0534806	592.3519937	470.5491428	540.0092464	156.5040663	191.6232935	195.033082
ENSG00000168685	IL7R	chr5:35852695-35879603	+	protein_coding	-1.563585811	0.13706961	-11.40723905	3.84748E-30	1.74462E-28	Yes	OK	1144.587606	1713.292805	575.8824081	1742.397088	1637.318956	1760.16237	629.6987137	440.2584761	657.6900345
ENSG00000101871	MID1	chrX:10445310-10833654	-	protein_coding	-1.554430395	0.112138535	-13.86169705	1.08088E-43	7.87226E-42	Yes	OK	906.6470287	1354.782747	458.5113105	1359.048723	1401.244131	1304.055387	498.0511756	399.0832229	478.3995331
ENSG00000108846	ABCC3	chr17:50634777-50692252	+	protein_coding	-1.544933797	0.12081479	-12.7876214	1.92269E-37	1.15082E-35	Yes	OK	770.9317967	1145.936018	395.9275765	1135.341321	1171.571335	1130.895397	356.2769038	462.4297662	369.0760566
ENSG00000127863	TNFRSF19	chr13:23570370-23676104	+	protein_coding	-1.544479549	0.116898338	-13.21216002	7.46511E-40	4.87602E-38	Yes	OK	804.3132799	1200.039106	408.5874536	1258.222852	1201.18072	1140.713747	432.6877126	353.156979	439.9176693
ENSG00000134138	MEIS2	chr15:36889204-37101299	-	protein_coding	-1.540778117	0.080087644	-19.23864945	1.75728E-82	2.70325E-80	Yes	OK	2425.23955	3610.321011	1240.158088	3490.045523	3633.151545	3707.765966	1293.460077	1213.086305	1213.927883
ENSG00000106484	MEST	chr7:130486171-130506465	+	protein_coding	-1.530083622	0.175263456	-8.730192016	2.54241E-18	6.77152E-17	Yes	OK	201.9307577	298.6910976	105.1704178	293.0251884	296.894102	306.1540025	103.1085613	126.6930866	85.70960556
ENSG00000111799	COL12A1	chr6:75084326-75206051	-	protein_coding	-1.524498576	0.064777051	-23.53454729	1.8074E-122	6.4672E-120	Yes	OK	127824.9623	189712.3427	65937.58185	191587.01	190993.3363	186556.682	69073.52995	60269.48498	68469.73061
ENSG00000011465	DCN	chr12:91140484-91183123	-	protein_coding	-1.521377421	0.068232854	-22.2968458	3.9648E-110	1.1449E-107	Yes	OK	32356.56871	47998.03022	16715.10721	48469.93707	48133.65619	47390.4974	17704.29234	15423.29963	17017.72964
ENSG00000149131	SERPING1	chr11:57597387-57614853	+	protein_coding	-1.511121257	0.082656857	-18.28186205	1.15418E-74	1.55719E-72	Yes	OK	2183.019123	3233.733422	1132.304823	3183.366831	3317.051355	3200.782079	1182.066006	1067.389255	1147.459209
ENSG00000164292	RHOBTB3	chr5:95713522-95824383	+	protein_coding	-1.506931107	0.117153826	-12.8628416	7.28485E-38	4.45757E-36	Yes	OK	3177.686089	4704.193843	1651.178336	4705.207325	4784.716539	4622.657665	1926.841239	1341.363055	1685.330713
ENSG00000150394	CDH8	chr16:61647242-62037035	-	protein_coding	-1.502305098	0.207346707	-7.24537716	4.31237E-13	8.21547E-12	Yes	OK	125.1741146	184.8189032	65.52932595	206.90309	167.2530116	180.3006079	71.80774805	69.68119765	55.09903214
ENSG00000185950	IRS2	chr13:109752698-109786568	-	protein_coding	-1.497561858	0.144826796	-10.34036447	4.62774E-25	1.71947E-23	Yes	OK	3945.699778	5824.261814	2067.137742	5830.045952	5671.397577	5971.341915	1646.975144	2698.562745	1855.875337
ENSG00000125266	EFNB2	chr13:106489731-106535662	-	protein_coding	-1.483885296	0.092413557	-16.05700886	5.10587E-58	5.00253E-56	Yes	OK	2397.299402	3534.291788	1260.307016	3569.866004	3648.356364	3384.652996	1360.664764	1135.486789	1284.769496
ENSG00000150556	LYPD6B	chr2:149038107-149215262	+	protein_coding	-1.483724027	0.253491145	-5.853159202	4.82322E-09	5.96023E-08	Yes	OK	170.0211579	248.8217701	91.22054568	218.4560544	243.2771079	284.7321481	58.91917789	137.7787317	76.96372744
ENSG00000147852	VLDLR	chr9:2621834-2660053	+	protein_coding	-1.476641395	0.121334352	-12.17001923	4.48977E-34	2.33128E-32	Yes	OK	947.0323381	1394.965017	499.0996594	1321.239021	1467.665184	1395.990845	578.144433	435.5074853	483.6470599
ENSG00000117592	PRDX6	chr1:173477266-173488807	+	protein_coding	-1.467503947	0.064101338	-22.89349949	5.3934E-116	1.8117E-113	Yes	OK	8545.366777	12552.97352	4537.760031	12226.18176	12768.84715	12663.88626	4690.518927	4424.756051	4498.005116
ENSG00000136040	PLXNC1	chr12:94148723-94307675	+	protein_coding	-1.467262466	0.164779179	-8.904416645	5.36675E-19	1.4772E-17	Yes	OK	196.9140576	289.0327979	104.7953174	306.6786918	287.2910583	273.1286436	96.66427622	109.2727872	108.4488887
ENSG00000107731	UNC5B	chr10:71212570-71302864	+	protein_coding	-1.466403818	0.128395848	-11.42095979	3.28585E-30	1.50236E-28	Yes	OK	1784.594246	2617.669414	951.519078	2649.829929	2519.198472	2683.979841	827.6303269	1175.078379	851.8485287
ENSG00000099204	ABLIM1	chr10:114431113-114685003	-	protein_coding	-1.45665152	0.173611849	-8.390277094	4.84998E-17	1.19507E-15	Yes	OK	287.9204703	423.5386168	152.3023238	377.0467477	463.34686	430.2222426	175.8369215	114.023778	167.0462721
ENSG00000085117	CD82	chr11:44564427-44620363	+	protein_coding	-1.450105896	0.240403822	-6.031958577	1.61984E-09	2.1313E-08	Yes	OK	93.19755922	135.6971582	50.69796026	148.0879984	145.6461633	113.3573129	43.26877126	63.34654332	45.47856621
ENSG00000119938	PPP1R3C	chr10:91628442-91633054	-	protein_coding	-1.446350758	0.104046619	-13.90098761	6.24733E-44	4.59068E-42	Yes	OK	4368.761104	6395.389226	2342.132982	6388.789323	6556.478107	6240.900249	2559.301789	1952.657198	2514.439959
ENSG00000138347	MYPN	chr10:68106117-68212017	+	protein_coding	-1.442570384	0.143664359	-10.04125445	1.00389E-23	3.53078E-22	Yes	OK	328.2500006	479.0614919	177.4385094	486.2747749	476.951172	473.9585287	187.8048795	193.2069571	151.3036914
ENSG00000198768	APCDD1L	chr20:58459101-58515131	-	protein_coding	-1.42957682	0.122373575	-11.6820712	1.57413E-31	7.66571E-30	Yes	OK	1124.978773	1638.035741	611.921805	1637.370138	1631.717181	1645.019903	535.7962739	733.2362389	566.7329021
ENSG00000117069	ST6GALNAC5	chr1:76867441-77065711	+	protein_coding	-1.429473445	0.115297403	-12.39814089	2.67457E-35	1.47236E-33	Yes	OK	599.7591101	874.3640194	325.1542007	810.8080481	909.8883935	902.3956167	313.0081325	330.9856888	331.4687807
ENSG00000154856	APCDD1	chr18:10454628-10489948	+	protein_coding	-1.424458638	0.156197994	-9.119570644	7.54225E-20	2.22085E-18	Yes	OK	225.0063419	327.0893708	122.9233131	309.8295002	338.5072915	332.9313205	117.8383558	134.6114046	116.320179
ENSG00000100889	PCK2	chr14:24094053-24110598	+	protein_coding	-1.421303876	0.106314921	-13.36880908	9.20021E-41	6.13099E-39	Yes	OK	2793.293785	4064.608236	1521.979334	3841.885803	4192.528842	4159.410063	1352.379255	1762.617568	1450.94118
ENSG00000164920	OSR2	chr8:98944403-98952104	+	protein_coding	-1.41576853	0.157422222	-8.993447775	2.39594E-19	6.72989E-18	Yes	OK	375.0657961	544.1916781	205.939914	496.7774699	560.9778046	574.8197598	163.8689635	242.3005282	211.6502505
ENSG00000196562	SULF2	chr20:47656348-47786616	-	protein_coding	-1.414068575	0.132520915	-10.67053135	1.39824E-26	5.51921E-25	Yes	OK	1008.210556	1463.439411	552.9817011	1485.081062	1424.451487	1480.785686	493.4481148	680.9753407	484.5216477
ENSG00000171867	PRNP	chr20:4686236-4701590	+	protein_coding	-1.409205484	0.072518943	-19.432396	4.11988E-84	6.58381E-82	Yes	OK	7411.602426	10771.48138	4051.723467	10795.72011	10943.46859	10575.25546	4275.322845	3767.535664	4112.311891
ENSG00000153944	MSI2	chr17:57255851-57684685	+	protein_coding	-1.408250642	0.124311306	-11.32841961	9.49016E-30	4.19914E-28	Yes	OK	785.3864804	1142.434102	428.3388587	1243.519079	1070.739376	1113.043852	487.9244419	381.6629235	415.4292106
ENSG00000185924	RTN4RL1	chr17:1934677-2025345	-	protein_coding	-1.405861912	0.278604671	-5.046081627	4.50963E-07	4.35102E-06	Yes	OK	156.0418754	224.8339391	87.24981167	209.003629	251.2796443	214.218544	57.99856573	142.5297225	61.22114683
ENSG00000139292	LGR5	chr12:71439770-71586310	+	protein_coding	-1.405618719	0.255015441	-5.511896519	3.54988E-08	3.96411E-07	Yes	OK	129.919179	189.9034621	69.93489586	228.9587493	172.0545335	168.6971034	69.96652374	42.75891674	97.07924711
ENSG00000105855	ITGB8	chr7:20330702-20415754	+	protein_coding	-1.402973357	0.207853177	-6.749828768	1.4802E-11	2.39804E-10	Yes	OK	134.2787766	196.0599671	72.49758616	186.9479696	204.8649329	196.3669987	82.8550939	50.67723465	83.96042993
ENSG00000184985	SORCS2	chr4:7192538-7742836	+	protein_coding	-1.397901857	0.178319573	-7.839306869	4.5304E-15	9.85078E-14	Yes	OK	712.9764668	1031.200438	394.752496	971.4992804	1001.917563	1120.18447	304.7226231	538.4456182	341.0892466
ENSG00000167981	ZNF597	chr16:3432422-3443542	-	protein_coding	-1.383171662	0.217362812	-6.363423674	1.97305E-10	2.84881E-09	Yes	OK	149.7338534	217.9634379	81.50426889	228.9587493	216.0684839	208.8630804	98.50550053	50.67723465	95.33007149
ENSG00000112297	CRYBG1	chr6:106360808-106571978	+	protein_coding	-1.380926715	0.132466437	-10.42472902	1.91204E-25	7.21837E-24	Yes	OK	645.7440555	931.1042908	360.3838202	900.0809549	935.4965101	957.7354073	359.0387402	421.2545131	300.8582073
ENSG00000106785	TRIM14	chr9:98069275-98119212	-	protein_coding	-1.376937267	0.134832701	-10.21219077	1.74871E-24	6.41065E-23	Yes	OK	464.8768243	671.8421583	257.9114903	599.7038801	694.6201632	721.2024316	284.4691557	239.133201	250.1321142
ENSG00000162849	KIF26B	chr1:245154985-245709431	+	protein_coding	-1.376033467	0.100883361	-13.6398456	2.32051E-42	1.6393E-40	Yes	OK	1640.138684	2365.872057	914.4053117	2464.982498	2311.932778	2320.700894	820.2654296	1015.128357	907.8221487
ENSG00000152953	STK32B	chr4:5051442-5500998	+	protein_coding	-1.373963963	0.101555948	-13.52913331	1.05257E-41	7.21889E-40	Yes	OK	777.9683243	1122.046452	433.8901962	1131.140243	1098.748254	1136.250861	424.4022032	451.3441211	425.9242644
ENSG00000087510	TFAP2C	chr20:56629302-56639283	+	protein_coding	-1.37354634	0.132692839	-10.3513223	4.12745E-25	1.53706E-23	Yes	OK	392.2471988	566.8859637	217.6084338	556.6428309	536.1699416	607.8451187	218.1850806	201.125275	233.5149458
ENSG00000179630	LACC1	chr13:43879284-43893932	+	protein_coding	-1.352825987	0.15995221	-8.45768862	2.72729E-17	6.84317E-16	Yes	OK	442.5091225	637.9939642	247.0242808	632.2622344	634.6011399	647.1185184	299.1989502	182.121312	259.7525801

ENSG00000171992	SYNPO	chr5:150601080-150659220	+	protein_coding	-1.349228858	0.128161877	-10.52753664	6.45003E-26	2.48055E-24	Yes	OK	627.8225171	899.5112961	356.133738	896.9301464	901.8858571	899.7178849	313.9287447	421.2545131	333.2179563
ENSG00000105516	DBP	chr19:48630030-48637438	-	protein_coding	-1.348028976	0.283559719	-4.753950812	1.9948E-06	1.73177E-05	Yes	OK	168.6248137	240.412315	96.83731233	246.8133307	228.0722886	246.3513256	60.76040219	161.5336855	68.21784932
ENSG00000169169	CPT1C	chr19:49690898-49713731	+	protein_coding	-1.335517309	0.202542683	-6.593757355	4.28832E-11	6.62778E-10	Yes	OK	343.9580212	491.6333887	196.2826536	481.0234275	491.3557376	502.5210012	123.3620287	240.7168646	224.7690676
ENSG00000178882	RFLNA	chr12:123973241-124316024	+	protein_coding	-1.335048643	0.169573758	-7.872967267	3.46327E-15	7.59061E-14	Yes	OK	307.3650687	438.2597946	176.4703429	444.2639952	423.3341778	447.1812107	150.0597812	228.0475559	151.3036914
ENSG00000169946	ZFPM2	chr8:104590733-105804532	+	protein_coding	-1.332838305	0.120138613	-11.09417087	1.33896E-28	5.63664E-27	Yes	OK	431.3738417	617.9611219	244.7865516	618.608731	619.3963206	615.8783141	245.8034452	237.5495374	251.006702
ENSG00000110031	LPXN	chr11:58526871-58578220	-	protein_coding	-1.329239798	0.084634059	-15.70573142	1.38175E-55	1.29963E-53	Yes	OK	1891.398346	2707.058697	1075.737995	2749.605531	2715.260615	2656.309946	1048.577244	1037.299647	1141.337094
ENSG00000111452	ADGRD1	chr12:130953907-131141469	+	protein_coding	-1.32918657	0.131044916	-10.14298642	3.56052E-24	1.28522E-22	Yes	OK	408.9167453	583.8908817	233.942609	594.4525326	607.392516	549.8275963	215.4232441	258.137164	228.2674189
ENSG00000077420	APBB1IP	chr10:26438203-26567803	+	protein_coding	-1.325872329	0.135349181	-9.795939063	1.17203E-22	3.90519E-21	Yes	OK	1181.599241	1686.657563	676.5409192	1749.748974	1604.508557	1705.715157	633.3811623	833.0070446	563.2345508
ENSG00000166482	MFAP4	chr17:19383442-19387240	-	protein_coding	-1.324743891	0.09592834	-13.80972387	2.22684E-43	1.6006E-41	Yes	OK	3014.826112	4306.523562	1723.128662	4152.765573	4356.580839	4410.224275	1535.581074	1925.734917	1708.069996
ENSG00000165124	SVEP1	chr9:110365251-110579880	-	protein_coding	-1.322375841	0.077576044	-17.04618822	3.73084E-65	4.17753E-63	Yes	OK	2962.525493	4231.97038	1693.080606	4342.864351	4182.925798	4170.12099	1602.785761	1732.52796	1743.928097
ENSG00000105464	GRIN2D	chr19:48394875-48444931	+	protein_coding	-1.320453061	0.145227602	-9.09230095	9.69649E-20	2.81986E-18	Yes	OK	444.1278348	632.2067564	256.0489133	606.0054971	642.6036763	648.0110957	220.0263049	311.9817258	236.1387092
ENSG00000243678	NME2	chr17:51165435-51171747	+	protein_coding	-1.319169516	0.495835409	-2.660498809	0.0078025	0.02899078	Yes	OK	159.7986848	227.5289645	92.06840509	240.5117137	214.4679767	227.607203	47.87183203	158.3663583	69.96702494
ENSG00000108379	WNT3	chr17:46762506-46833154	-	protein_coding	-1.317563471	0.276734043	-4.761118142	1.92523E-06	1.6758E-05	Yes	OK	128.7476816	182.1776765	75.31768664	177.4955442	197.6626501	171.3748352	47.87183203	120.3584323	57.72279558
ENSG00000167642	SPINT2	chr19:38244035-38292614	+	protein_coding	-1.315299517	0.2310776	-5.692025186	1.25541E-08	1.49092E-07	Yes	OK	127.4414652	180.5603225	74.32260786	189.0485086	192.8611283	159.7713308	53.39550496	101.3544693	68.21784932
ENSG00000101255	TRIB3	chr20:362835-397559	+	protein_coding	-1.311354493	0.095656132	-13.70904785	8.96263E-43	6.38636E-41	Yes	OK	2035.561608	2899.36909	1171.754126	2830.476282	2910.522504	2957.108485	1058.703978	1298.604138	1157.954263
ENSG00000165029	ABCA1	chr9:104781002-104928237	-	protein_coding	-1.308947336	0.101610187	-12.88204831	5.68076E-38	3.48901E-36	Yes	OK	1037.109178	1479.031501	595.1868544	1568.052352	1459.662647	1409.379504	591.0330032	562.200572	632.3269879
ENSG00000196878	LAMB3	chr1:209614870-209652466	-	protein_coding	-1.30888792	0.161563646	-8.101376461	5.43408E-16	1.23714E-14	Yes	OK	256.7908572	365.5091735	148.0725409	367.5943223	358.5136326	370.4195657	119.6795801	158.3663583	166.1716842
ENSG00000149596	JPH2	chr20:44111695-44187578	-	protein_coding	-1.301749128	0.136136826	-9.562064646	1.15431E-21	3.67502E-20	Yes	OK	1334.841518	1896.36418	773.3188555	1852.675384	1855.788201	1980.628955	669.2850363	967.6184492	683.053081
ENSG00000159164	SV2A	chr1:149903318-149917882	-	protein_coding	-1.29878935	0.130636476	-9.942011493	2.73253E-23	9.44904E-22	Yes	OK	479.2268483	681.5362953	276.9174013	607.0557665	753.8389329	683.7141864	280.7867071	269.2228091	280.7426876
ENSG00000072163	LIMS2	chr2:127638381-127681786	-	protein_coding	-1.289493444	0.166747194	-7.733224242	1.04856E-14	2.24439E-13	Yes	OK	1467.309573	2079.860346	854.7587989	2187.711352	2046.248568	2005.621118	680.3323821	1162.40907	721.5349447
ENSG00000120885	CLU	chr8:27596917-27615031	-	protein_coding	-1.283126492	0.104295434	-12.30280602	8.74813E-35	4.64498E-33	Yes	OK	2190.480886	3102.219237	1278.742535	2939.704309	3069.772979	3297.180423	1200.478249	1458.55416	1177.195195
ENSG00000176485	PLA2G16	chr11:63573195-63616883	-	protein_coding	-1.277861275	0.229625834	-5.56497174	2.62195E-08	2.98048E-07	Yes	OK	95.60373744	135.7474713	55.46000355	121.8312611	125.6398221	159.7713308	54.31611711	49.09357107	62.97032245
ENSG00000116117	PARD3B	chr2:204545793-205620162	+	protein_coding	-1.275867478	0.101124962	-12.61674125	1.70742E-36	9.75836E-35	Yes	OK	906.9595276	1284.828909	529.0901457	1287.630398	1309.214962	1257.641369	519.2252551	500.4376922	567.6074899
ENSG00000197077	KIAA1671	chr22:24952730-25197448	+	protein_coding	-1.271912492	0.149339521	-8.516918241	1.63855E-17	4.18148E-16	Yes	OK	249.3603102	352.5357461	146.1848743	380.1975562	340.9080525	336.5016296	147.2979447	150.4480404	140.8086377
ENSG00000114270	COL7A1	chr3:48564073-48595267	-	protein_coding	-1.271657355	0.11352871	-11.20119624	4.02265E-29	1.74704E-27	Yes	OK	2437.445183	3444.227021	1430.663345	3420.727736	3374.669618	3537.283708	1224.414165	1686.601716	1380.974155
ENSG00000204525	HLA-C	chr6:31268749-31272130	-	protein_coding	-1.270120205	0.109558295	-11.5930994	4.46671E-31	2.12492E-29	Yes	OK	8944.654294	12642.28057	5247.028016	12224.08662	12880.88266	12821.87244	4523.888127	6287.144424	4930.051495
ENSG00000182326	C1S	chr12:6988259-7071032	+	protein_coding	-1.261599182	0.064230623	-19.64170872	6.80689E-86	1.12041E-83	Yes	OK	7303.191561	10309.18914	4297.193981	10194.96596	10392.09382	10340.50764	4417.097117	4136.529279	4337.955547
ENSG00000130513	GDF15	chr19:18374731-18389176	+	protein_coding	-1.260111898	0.135502304	-9.299560666	1.41027E-20	4.29872E-19	Yes	OK	596.3165389	839.3010054	353.3320723	848.6177498	795.4521224	873.8331442	306.5638474	422.8381766	330.5941929
ENSG00000115841	RMDN2	chr2:37923187-38067142	+	protein_coding	-1.255929129	0.174878631	-7.181718654	6.88405E-13	1.28763E-11	Yes	OK	229.3329158	324.6596324	134.0061993	353.9408189	312.0989213	307.939157	160.1865149	104.5217965	137.3102865
ENSG00000188042	ARL4C	chr2:234493041-234497053	-	protein_coding	-1.255737028	0.091242617	-13.76261515	4.27769E-43	3.06134E-41	Yes	OK	1568.520154	2212.871341	924.1689674	2141.499495	2225.505385	2271.609144	960.1984771	852.0110076	960.2974174
ENSG00000100979	PLTP	chr20:45898621-45912155	-	protein_coding	-1.255097963	0.182308938	-6.88445655	5.80086E-12	9.783E-11	Yes	OK	581.030585	816.5643171	345.4967989	779.2999633	785.8490787	884.5440714	278.0248706	475.0990749	283.366451
ENSG00000197635	DPP4	chr2:161992241-162074542	-	protein_coding	-1.253173126	0.117209281	-10.69175676	1.11244E-26	4.41222E-25	Yes	OK	2050.686528	2892.24364	1209.129416	2928.151345	2905.720982	2842.858595	1317.395993	977.1204307	1332.871825
ENSG00000107796	ACTA2	chr10:88935074-88991339	-	protein_coding	-1.252381325	0.098664304	-12.69335789	6.43658E-37	3.75696E-35	Yes	OK	22552.6606	31765.72528	13339.59592	31693.98247	31891.70823	31711.48513	12574.64142	15686.18779	11757.95854
ENSG00000148803	FUOM	chr10:133355154-133358035	-	protein_coding	-1.251775502	0.215590803	-5.806256508	6.38851E-09	7.78925E-08	Yes	OK	116.2003391	163.1055833	69.29509487	143.8869204	162.4514898	182.9783397	55.23672927	79.18317915	73.46537619
ENSG00000254122	PCDHGB7	chr5:141417645-141512979	+	protein_coding	-1.246269003	0.138720626	-8.984020924	2.61048E-19	7.29516E-18	Yes	OK	390.76754	548.5898739	232.9452061	536.6877106	568.1800874	540.9018237	243.0416088	258.137164	197.6568455
ENSG00000122176	FMOD	chr1:203340628-203351489	-	protein_coding	-1.245538108	0.115362532	-10.79672999	3.56681E-27	1.43194E-25	Yes	OK	830.404237	1166.039274	494.7691997	1139.542399	1133.959414	1224.61601	463.9885258	563.7842355	456.5348378
ENSG00000082196	C1QTNF3	chr5:34019448-34043832	-	protein_coding	-1.244629827	0.181010643	-6.876003562	6.1555E-12	1.03493E-10	Yes	OK	298.4884569	421.4378509	175.5390628	379.1472867	443.3405189	441.8257471	208.0583469	123.5257595	195.033082
ENSG00000123500	COL10A1	chr6:116118923-116158747	-	protein_coding	-1.243054664	0.229901898	-5.406891701	6.41279E-08	6.93525E-07	Yes	OK	107.4457231	151.9284307	62.96301558	182.7468917	140.0443877	132.9940127	67.20468728	49.09357107	72.59078838
ENSG00000110076	NRXN2	chr11:64606174-64723188	-	protein_coding	-1.241462324	0.204170861	-6.080506886	1.19803E-09	1.59802E-08	Yes	OK	133.6337357	186.9396615	80.32780989	161.7415018	208.0659475	191.0115351	68.12529943	95.01981498	77.83831525
ENSG00000144810	COL8A1	chr3:99638475-99799226	+	protein_coding	-1.236911631	0.067250876	-18.39249851	1.50865E-75	2.06936E-73	Yes	OK	26371.53453	37035.01183	15708.05724	36736.3263	37236.60232	37132.10688	16258.01065	14525.36238	16340.79868
ENSG00000221926	TRIM16	chr17:15627960-15684311	-	protein_coding	-1.231824488	0.212486055	-5.797201557	6.74306E-09	8.1912E-08	Yes	OK	161.5715922	225.5217611	97.62142322	252.0646782	212.0672157	212.4333895	69.96652374	126.6930866	96.2046593
ENSG00000158290	CUL4B	chrX:120524609-120575794	-	protein_coding	-1.229880115	0.107657136	-11.42404637	3.17118E-30	1.45397E-28	Yes	OK	4714.984092	6614.373958	2815.594226	6741.679871	6729.332894	6372.109108	3189.921115	2337.487448	2919.374116
ENSG00000171385	KCND3	chr1:111770662-111989155	-	protein_coding	-1.221788561	0.245515802	-4.976415173	6.47726E-07	6.0993E-06	Yes	OK	104.3766832	147.2147392	61.53862716	144.9371899	136.0431195	160.663908	81.01386959	38.00792599	65.59408589
ENSG00000169583	CLIC3	chr9:136994635-136996803	-	protein_coding	-1.221464143	0.198637984	-6.149197237	7.78761E-10	1.06113E-08	Yes	OK	418.3286621	583.3068333	253.3504909	544.039597	603.3912478	602.4896551	192.4079403	359.4916333	208.1518992
ENSG00000138759	FRAS1	chr4:78057570-78544269	+	protein_coding	-1.219437425	0.082858986	-14.71702093	5.01261E-49	4.10485E-47	Yes	OK	3138.312032	4388.377763	1888.246302	4496.203697	4369.384898	4299.544694	1807.161659	2052.428004	1805.149244
ENSG00000111371	SLC38A1	chr12:46183063-46270017	-	protein_coding	-1.216936854	0.098840876	-12.31208076	7.79863E-35	4.15422E-33	Yes	OK	6589.562414	9218.219142	3960.905687	9421.967615	9175.708285	9056.981526	4446.556706	3368.452441	4067.707913
ENSG00000140961	OSGIN1	chr16:83948282-83966332	+	protein_coding	-1.215958661	0.143004356	-8.502948433	1.84835E-17	4.69504E-16	Yes	OK	265.0110657	370.1285175	159.8936139	369.6948613	364.9156618	375.7750293	149.139169	167.8683398	162.673333
ENSG00000006534	ALDH3B1	chr11:68008578-68029282	+	protein_coding	-1.213109139	0.115075579	-10.54184694	5.54E-26	2.13556E-24	Yes	OK	1190.784092	1662.18328	719.3849036	1523.941033	1645.321493	1817.287315	640.7460595	794.9991186	722.4095326
ENSG00000121989	ACVR2A	chr2:147844571-147930826	+	protein_coding	-1.208348202	0.1614277	-7.485383251	7.13387E-14	1.43725E-12	Yes	OK	464.9155003	650.371668	279.4593326	661.6697801	645.0044373	644.4407866	358.1181281	224.8802288	255.379641
ENSG00000143028	SYPL2	chr1:109466628-109482137	+	protein_coding	-1.207770907	0.136759263	-8.831364565	1.03406E-18	2.80869E-17	Yes	OK	542.2863452	754.8893773	329.6833131	766.6967294	756.2396938	741.7317087	279.866095	394.3322322	314.8516123
ENSG00000147642	SYBU	chr8:109573978-109691791	-	protein_coding	-1.207092687	0.213836111	-5.644943124	1.65236E-08	1.93029E-07	Yes	OK	103.0892186	144.0589921	62.11944508	131.2836865	161.6512361	139.2420536	68.12529943	57.01188899	61.22114683
ENSG00000162946	DISC1	chr1:231626815-232041272	+	protein_coding	-1.201443464	0.236249022	-5.085496029	3.66667E-07	3.57332E-06	Yes	OK	91.16252475	127.4912194	54.83383012	123.9318001	149.6474315	108.8944265	62.6016265	45.92624391	55.97361996
ENSG00000153531	ADPRHL1	chr13:113399610-113453524	-	protein_coding	-1.20046304	0.219640292	-5.465586602	4.61378E-08	5.0866E-07	Yes	OK	100.4485933	140.5425931	60.35459359	124.9820696	152.0481924	144.5975172	68.12529943	49.09357107	63.84491026
ENSG00000161681	SHANK1	chr19:50661827-50719450	-	protein_coding	-1.200385128	0.185099405	-6.485083664	8.86826E-11	1.32358E-09	Yes	OK	155.3422827	215.6017627	95.08280275	193.2495866	223.2707667	230.2849348	91.14060329	109.2727872	84.83501775
ENSG00000146674	IGFBP3	chr7:45912245-45921874	-	protein_coding	-1.198400087	0.043891508	-27.30368881	3.8347E-164	2.4277E-161	Yes	OK	167557.1977	233406.1513	101708.2441	231717.8073	233618.0464	234882.6003	101087.8176	101424.1505	102612.7642
ENSG00000145632	PLK2	chr5:58453982-58460260	-	protein_coding	-1.196046805	0.096294095	-12.42076994	2.01612E-35	1.12113E-33	Yes	OK	2043.257783	2847.353744	1239.161822	2873.537331	2856.90551	2811.61839	1356.061704	1095.895199	1265.528564
ENSG00000248905	FMN1	chr15:32765545-33194733	-	protein_coding	-1.195416304	0.110488482	-10.81937483	2.78672E-27	1.12701E-25	Yes	OK	1022.406664	1425.199881	619.6134458	1542.845884	1387.639819	1345.113941	658.2376904	554.282254	646.3203929
ENSG00000150594	ADRA2A	chr10:111077163-111080907	+	protein_coding	-1.194109045	0.117040436	-10.20253413	1.93165E-24	7.06556E-23	Yes	OK	1619.44226	2251.124474	987.7600457	2137.298417	2323.936583	2292.138421	852.486855	1159.241743	951.5515392
ENSG00000178038	ALS2CL	chr3:46668997-46693704	-	protein_coding	-1.191289171	0.212425827	-5.608024152	2.04649E-08	2.36057E-07	Yes	OK	120.1706479	166.3445619	73.99673386	175.3950052	165.6525044	157.9861762	77.33142097	88.68516065	55.97361996
ENSG00000132386	SERPINF1	chr17:1761959-1777574	+	protein_coding	-1.179946667	0.111107586	-10.61985689	2.4093E-26	9.41974E-25	Yes	OK	1713.386172	2375.064687	1051.707656	2226.571438	2381.554845	2517.067892	920.6121545	1197.249669	1037.261145
ENSG00000138162	TACC2	chr10:121989174-122254545	+	protein_coding	-1.179298968	0.11864264	-9.939925203	2.79037E-23	9.58861E-22	Yes	OK	468.7164604	649.9120723	287.5208484	620.7092699	681.0158513	648.0110957	284.4691557	292.9777628	285.1156266
ENSG00000185633	NDUFA4L2	chr12:57234903-57240715	-	protein_coding	-1.177351794	0.204869215	-5.746845841	9.09236E-09	1.08923E-07	Yes	OK	133.5691809	184.3523422	82.78601951	194.2998561	178.4565627	180.3006079	85.61693037	99.77080573	62.97032245
ENSG00000128655	PDE11A	chr2:177623252-178072755	-	protein_coding	-1.176806237	0.191145531	-6.156598222	7.43241E-10	1.01525E-08	Yes	OK	229.3694386	319.3379735	139.4009037	322.4327341	308.8979067	326.6832796	183.2018187	102.9381329	132.0627596
ENSG00000091409	ITGA6	chr2:172427354-172506282	+	protein_coding	-1.176705315	0.11675199	-10.07867461	6.86458E-24	2.44569E-22	Yes	OK	2025.380422	2811.043586	1239.717258	2906.095685	2860.906778	2666.128296	1418.66333	1026.214002	1274.274442
ENSG00000196517	SLC6A9	chr1:43991500-44031467	-	protein_coding	-1.169783982	0.115801169	-10.10165957	5.43151E-24	1.94777E-22	Yes	OK	1407.099899	1945.588944	868.6108537	1936.696944	2039.046286	1861.023601	769.6317611	1016.71202	819.4887797
ENSG00000175315	CST6	chr11:66011841-66013505	+	protein_coding	-1.169208061	0.279667149	-4.180712906	2.90597E-05	0.000205288	Yes	OK	92.22494326	126.573367	57.87651951	94.52425431	135.2428659	149.9529808	41.42754695	82.35050631	49.85150527
ENSG00000178033	CALHM5	chr6:116511646-116524792	+	protein_coding	-1.16771808	0.101172359	-11.54186868	8.1143E-31	3.79436E-29	Yes	OK	1010.314192	1399.593125	621.0352592	1415.763276	1454.861125	1328.154973	632.4605501	576.4535442	654.1916832
ENSG00000087842	PIR	chrX:15384799-15493564	-	protein_coding	-1.166164534	0.179677001	-6.490338374	8.56438E-11	1.28388E-09	Yes	OK	144.6498789	200.5519438	88.747814	211.104168	196.8623965	193.6892669	92.06121545	82.35050631	91.83172024
ENSG00000065911	MTHFD2	chr2:74198562-74217565	+	protein_coding	-1.163088754	0.089289489	-13.02604337	8.70036E-39	5.46595E-37	Yes	OK	4123.192294	5703.815107	2542.569481	5701.913074	5781.83258	5627.699667	2713.044019	2239.300306	2675.364116
ENSG00000187824	TMEM220	chr17:10699015-10730316	-	protein_coding	-1.162053629	0.163558836	-7.104804955	1.20493E-12	2.21105E-11	Yes	OK	208.6619128	289.5715771	127.7522484	294.0754578	280.0887755	294.550498	138.0918232	106.1054601	139.0594621
ENSG00000155966	AFF2	chrX:148500619-149000663	+	protein_coding	-1.161424959	0.111609706	-10.40612861	2.32485E-25	8.73675E-24	Yes	OK	673.3074733	930.1987993	416.4161474	983.0522488	912.2891544	895.2549986	385.7364927	432.3401581	431.1717912
ENSG00000165996	HACD1	chr10:17589032-17617377	-	protein_coding	-1.161047179	0.102329538	-11.34615871	7.74929E-30	3.44739E-28	Yes	OK	1207.27308	1669.566408	744.9797524	1554.398849	1763.759032	1690.541343	740.1721722	693.6446493	801.1224356
ENSG00000154736	ADAMTS5	chr21:26917512-26966513	-	protein_coding	-1.159852515	0.08270271	-14.02435931	1.10607E-44	8.3513E-43	Yes	OK	2217.216679	3062.353818	1372.079539	3206.47276	2944.933411	3035.655284	1339.490685	1431.631879	1345.116055
ENSG00000134853	PDGFRA	chr4:54229097-54298247	+	protein_coding	-1.157097738	0.084411092	-13.70788724	9.10713E-43	6.46135E-41	Yes	OK	8816.129433	12176.91291	5455.345955	12417.33621	12143.0488	11970.35372	5913.091868	4838.092246	5614.853752
ENSG00000158825	CDA	chr1:20588948-20618908	+	protein_coding	-1.155086065	0.172218794	-6.707084839	1.98551E-11	3.18533E-10	Yes	OK	197.8466488	271.8855814	123.8077161	278.3214155	255.2809125	282.0544163	113.235295	148.8643768	109.3234765
ENSG00000204682	CASC10	chr10:21492658-21497262	-	protein_coding	-1.153398953	0.168003853	-6.865312506	6.63459E-12	1.11207E-10	Yes	OK	176.642606	243.9299487	109.3552633	254.1652171	231.2733032	246.3513256	118.7589679	106.1054601	103.2013618
ENSG00000115902	SLC1A4	chr2:64988477-65023865	+	protein_coding	-1.149191928	0.073495356	-15.63625235	4.12289E-55	3.81251E-53	Yes	OK	2827.272206	3896.487398	1758.057013	3903.851703	3956.454017	3829.156474	1764.8135	1794.290839	1715.066699
ENSG00000165568	AKR1E2	chr10:4786629-4848062	+	protein_coding	-1.135414425	0.274788873	-4.131951965	3.59696E-05	0.000249498	Yes	OK	108.3319263	147.7944055	68.86944706	149.1382679	149.6474315	144.5975172	37.74509833	99.77080573	69.09243713
ENSG00000116157	GPX7	chr1:52602372-52609051	+	protein_coding	-1.132567967	0.119823366	-9.451979265	3.32483E-21	1.0464E-19	Yes	OK	750.4433286	1029.733251	471.1534058	1124.838626	937.8972711	1026.463857	434.5289369	516.274328	462.6569524
ENSG00000173421	CCDC36	chr3:49198428-49258104	+	protein_coding	-1.128949538	0.225268795	-5.011566461	5.39887E-07	5.16359E-06	Yes	OK	84.35544662	115.8531166	52.85777667	112.3788357	120.0380466	115.1424674	58.91917789	50.67723465	48.97691746

ENSG00000123096	SSPN	chr12:26121991-26299290	+	protein_coding	-1.127639063	0.140488066	-8.026582557	1.00225E-15	2.25988E-14	Yes	OK	891.0661441	1224.994318	557.1379706	1244.569348	1207.582749	1222.830855	647.1903446	426.0055038	598.2180633
ENSG00000134343	ANO3	chr11:26309599-26663288	+	protein_coding	-1.126332717	0.297881224	-3.781147071	0.000156107	0.000936758	Yes	OK	85.9559255	119.0459313	52.86591974	139.6858425	111.2352566	106.2166947	58.91917789	25.33861733	74.339964
ENSG00000204941	PSG5	chr19:43166256-43186536	-	protein_coding	-1.123503331	0.062582411	-17.95238163	4.59758E-72	5.86637E-70	Yes	OK	8383.36486	11490.11504	5276.614676	11419.58019	11543.65882	11507.10612	5150.825004	5469.974016	5209.045007
ENSG00000105472	CLEC11A	chr19:50723329-50725718	+	protein_coding	-1.116569527	0.161394331	-6.918269821	4.57193E-12	7.81453E-11	Yes	OK	2028.533397	2773.857216	1283.209579	2740.153105	2714.460361	2866.958181	969.4045986	1713.523997	1166.700141
ENSG00000175356	SCUBE2	chr11:9020391-9138114	-	protein_coding	-1.115595895	0.195243021	-5.713883591	1.10427E-08	1.31712E-07	Yes	OK	141.7417507	194.2322455	89.25125584	202.702012	203.2644256	176.7302988	76.41080882	85.51783348	105.8251252
ENSG00000197620	CXorf40A	chrX:149540355-149550510	+	protein_coding	-1.115268911	0.173589277	-6.424756917	1.3208E-10	1.9342E-09	Yes	OK	177.1565185	242.1955611	112.1174759	211.104168	251.2796443	264.202871	108.6322342	114.023778	113.6964155
ENSG00000167081	PBX3	chr9:125747345-125967377	+	protein_coding	-1.114363799	0.098121237	-11.35700924	6.8448E-30	3.06156E-28	Yes	OK	1577.952645	2161.111396	994.7938939	2234.97348	2107.067845	2141.292863	1072.51316	886.8516065	1025.016915
ENSG00000214944	ARHGEF28	chr5:73626158-73941993	+	protein_coding	-1.11272517	0.133409938	-8.340646806	7.38853E-17	1.79904E-15	Yes	OK	322.4160815	440.4400795	204.3920834	440.0629173	438.538997	442.7183243	189.6461038	215.3782473	208.1518992
ENSG00000113721	PDGFRB	chr5:150113837-150155872	-	protein_coding	-1.112530216	0.094419083	-11.78289585	4.78214E-32	2.34967E-30	Yes	OK	11611.66819	15875.76974	7347.566644	15501.97771	16365.98728	15759.34422	6528.981399	8440.926897	7072.791634
ENSG00000170006	TMEM154	chr4:152618632-152680165	-	protein_coding	-1.104036907	0.149868277	-7.366715146	1.74884E-13	3.41469E-12	Yes	OK	283.805013	386.0985425	181.5114834	383.3483647	369.7171836	405.2300791	171.2338607	210.6272565	162.673333
ENSG00000179981	TSHZ1	chr18:75210755-75289950	+	protein_coding	-1.102252743	0.098416381	-11.19989099	4.08236E-29	1.7683E-27	Yes	OK	1175.560344	1601.965035	749.1556521	1604.811784	1622.114137	1578.969185	719.9187048	823.5050631	704.0431885
ENSG00000131386	GALNT15	chr3:16174649-16231992	+	protein_coding	-1.099732297	0.124983771	-8.799000769	1.3804E-18	3.72481E-17	Yes	OK	391.7151627	534.2858318	249.1444936	509.3807038	540.9714635	552.5053281	243.9622209	245.4678554	258.0034045
ENSG00000141448	GATA6	chr18:22169443-22202528	+	protein_coding	-1.099468225	0.135300596	-8.126115188	4.43268E-16	1.02045E-14	Yes	OK	301.838837	411.1713569	192.5063172	407.504563	414.5313877	411.47812	181.3605944	201.125275	195.033082
ENSG00000198542	ITGBL1	chr13:101452593-101720856	+	protein_coding	-1.097670804	0.09039374	-12.14321698	6.23265E-34	3.22608E-32	Yes	OK	7058.20153	9617.511606	4498.891454	9685.585258	9735.085582	9431.863978	4416.176505	5066.139802	4014.358056
ENSG00000002745	WNT16	chr7:121325367-121341104	+	protein_coding	-1.091491536	0.199293753	-5.476797534	4.33092E-08	4.78757E-07	Yes	OK	129.1244289	176.6273418	81.62151597	169.0933883	184.0583382	176.7302988	82.8550939	64.9302069	97.07924711
ENSG00000186340	THBS2	chr6:169215780-169254044	-	protein_coding	-1.088655386	0.064046756	-16.99782256	8.52273E-65	9.47866E-63	Yes	OK	18049.12595	24550.16777	11548.08413	24362.05114	24514.97014	24773.48204	11160.58115	12241.7195	11241.95173
ENSG00000155755	TMEM237	chr2:201620184-201643570	-	protein_coding	-1.086231258	0.119237162	-9.109838231	8.25049E-20	2.42073E-18	Yes	OK	1375.986156	1873.321272	878.6510401	1869.479696	1896.601137	1853.882983	997.9435754	720.5669302	917.4426146
ENSG00000107201	DDX58	chr9:32455705-32526324	-	protein_coding	-1.082295671	0.139995575	-7.730927691	1.06766E-14	2.28229E-13	Yes	OK	371.2947286	505.9458507	236.6436064	532.4866326	480.1521865	505.198733	255.0095668	199.5416115	255.379641
ENSG00000075223	SEMA3C	chr7:80742538-80922359	-	protein_coding	-1.0802185	0.108342344	-9.970418402	2.05362E-23	7.14642E-22	Yes	OK	7675.790228	10425.44795	4926.13251	10507.94627	10437.70828	10330.68929	5672.812096	4047.844118	5057.741316
ENSG00000234745	HLA-B	chr6:31269491-31357188	-	protein_coding	-1.080189072	0.091409195	-11.81707241	3.18594E-32	1.58911E-30	Yes	OK	5611.960679	7617.379701	3606.541656	7244.758958	7537.589075	8069.791069	3228.586826	3970.244602	3620.793541
ENSG00000197766	CFD	chr19:859643-863630	+	protein_coding	-1.077934989	0.218767918	-4.927299197	8.3374E-07	7.7011E-06	Yes	OK	162.2569109	218.7165199	105.7973019	215.3052459	232.8738105	207.9705032	75.49019667	145.6970496	96.2046593
ENSG00000188157	AGRN	chr1:1020123-1056118	+	protein_coding	-1.077577668	0.327363871	-3.291681713	0.000995903	0.004959926	Yes	OK	2842.673626	3856.663625	1828.683628	3720.054542	3870.826877	3979.109455	1280.571507	2639.967193	1565.512183
ENSG00000175906	ARL4D	chr17:43398959-43401137	+	protein_coding	-1.077227172	0.155168321	-6.942313795	3.85729E-12	6.65524E-11	Yes	OK	713.5853114	965.990394	461.1802287	979.9014363	905.8871253	1012.182621	376.5303712	590.7065164	416.3037984
ENSG00000102466	FGF14	chr13:101710804-102402457	-	protein_coding	-1.074981256	0.186587109	-5.761283611	8.34766E-09	1.00367E-07	Yes	OK	206.2036721	281.0496887	131.3576554	288.8241104	247.2783761	307.0465798	145.4567204	98.18714214	150.4291036
ENSG00000181019	NQO1	chr16:69706996-69726951	-	protein_coding	-1.072899106	0.069229407	-15.49773642	3.5933E-54	3.23201E-52	Yes	OK	7068.378108	9584.542946	4552.21327	9554.301571	9647.857935	9551.469332	4705.248721	4267.973356	4683.417732
ENSG00000152689	RASGRP3	chr2:33436324-33564750	+	protein_coding	-1.072762336	0.258883895	-4.143797116	3.41602E-05	0.000238354	Yes	OK	78.79523287	107.1635432	50.42692252	95.5745238	115.2365248	110.6795811	68.12529943	41.17525316	41.98021497
ENSG00000213949	ITGA1	chr5:52787896-52959210	+	protein_coding	-1.071369405	0.129148204	-8.295658579	1.07985E-16	2.57974E-15	Yes	OK	2588.801143	3510.703784	1666.898502	3611.876784	3496.308172	3423.926395	2011.537558	1323.942755	1665.215194
ENSG00000172201	ID4	chr6:19837386-19840684	+	protein_coding	-1.063755381	0.098453184	-10.80468237	3.27087E-27	1.31957E-25	Yes	OK	1378.230685	1865.241112	891.2202573	1882.08293	1774.962583	1938.677823	973.0870473	858.345662	842.2280628
ENSG00000176209	SMIM19	chr8:42541155-42555193	+	protein_coding	-1.062785614	0.228821483	-4.64460591	3.40726E-06	2.84544E-05	Yes	OK	343.6884413	466.5099256	220.8669569	431.6607613	500.1585277	467.7104878	279.866095	120.3584323	262.3763435
ENSG00000103257	SLC7A5	chr16:87830023-87869488	-	protein_coding	-1.062527475	0.119375335	-8.90072871	5.54812E-19	1.52203E-17	Yes	OK	6511.790754	8803.668276	4219.913232	8832.76643	8666.746967	8911.491431	3510.294145	5159.575953	3989.869597
ENSG00000135362	PRR5L	chr11:36296288-36465204	+	protein_coding	-1.061917	0.124390826	-8.536939868	1.37823E-17	3.5336E-16	Yes	OK	460.6877931	622.5582251	298.817361	568.1957953	635.4013935	664.0774865	289.0722165	305.6470715	301.7327951
ENSG00000265972	TXNIP	chr1:145992435-145996600	-	protein_coding	-1.061075824	0.092548423	-11.4650881	1.97558E-30	9.08327E-29	Yes	OK	1501.859207	2030.770154	972.9482602	1993.411496	2025.441974	2073.456991	906.8029721	966.0347856	1046.007023
ENSG00000187688	TRPV2	chr17:16415542-16437003	+	protein_coding	-1.060190626	0.173934943	-6.095328578	1.09213E-09	1.46269E-08	Yes	OK	805.1223286	1086.0128	524.2318567	1010.359252	1050.733035	1196.946115	403.2281237	701.5629673	467.9044793
ENSG00000198113	TOR4A	chr9:137277749-137282641	+	protein_coding	-1.059665015	0.196540388	-5.391589111	6.98373E-08	7.51813E-07	Yes	OK	499.9018966	673.4378357	326.3659575	655.3681632	671.4128075	693.5325363	243.9622209	464.0134298	271.1222217
ENSG00000173801	JUP	chr17:41754604-41786931	-	protein_coding	-1.059432658	0.220854794	-4.796964738	1.61088E-06	1.42325E-05	Yes	OK	100.7050539	136.2402065	65.16990135	113.4291052	156.0494606	139.2420536	65.36346297	60.17921615	69.96702494
ENSG00000064012	CASP8	chr2:201233443-201287711	+	protein_coding	-1.05267615	0.180998723	-5.815931366	6.02972E-09	7.3627E-08	Yes	OK	338.6871378	458.8812883	218.4929873	461.0683071	450.5428017	465.032756	266.9775248	148.8643768	239.6370604
ENSG00000100342	APOL1	chr22:36253010-36267530	+	protein_coding	-1.05145875	0.155676584	-6.754122696	1.43702E-11	2.33268E-10	Yes	OK	251.5964441	338.9873666	164.2055216	332.9354291	314.4996822	369.5269884	144.5361083	175.7866577	172.2937989
ENSG00000179456	ZBTB18	chr1:244048939-244057476	+	protein_coding	-1.04984601	0.096726498	-10.85375809	1.91391E-27	7.75934E-26	Yes	OK	870.4716798	1173.742939	567.2004206	1146.894286	1167.570067	1206.764465	572.6207601	570.1188899	558.8616117
ENSG00000115844	DLX2	chr2:172099439-172102900	-	protein_coding	-1.046956813	0.244566715	-4.280863871	1.86169E-05	0.000136133	Yes	OK	149.1975209	199.7449475	98.65009436	222.6571324	163.2517434	213.3259668	77.33142097	142.5297225	76.08913963
ENSG00000152778	IFIT5	chr10:89414586-89421001	+	protein_coding	-1.045954	0.10995956	-9.512169711	1.86727E-21	5.89927E-20	Yes	OK	787.1644317	1062.078324	512.2505389	1113.285662	1041.129991	1031.81932	558.8115778	467.180757	510.7592821
ENSG00000119280	C1orf198	chr1:230837119-230869589	-	protein_coding	-1.045662309	0.06861319	-15.23996048	1.91986E-52	1.6809E-50	Yes	OK	6286.640666	8468.514262	4104.767071	8488.278037	8345.845256	8571.419493	3896.030638	4272.724347	4145.546228
ENSG00000137809	ITGA11	chr15:68296533-68432162	-	protein_coding	-1.045449027	0.083848034	-12.46837846	1.11063E-35	6.19694E-34	Yes	OK	27157.87698	36584.91659	17730.83736	36406.54168	36682.02655	36666.18154	16061.92026	19992.16907	17138.42276
ENSG00000104611	SH2D4A	chr8:19313617-19396218	+	protein_coding	-1.045422475	0.093674584	-11.16015072	6.38832E-29	2.73833E-27	Yes	OK	4531.511456	6108.003302	2955.01961	6104.166289	6123.540886	6096.302732	3281.061719	2579.787977	3004.209134
ENSG00000114698	PLSCR4	chr3:146192339-146251179	-	protein_coding	-1.044545714	0.157007962	-6.65282004	2.8753E-11	4.51597E-10	Yes	OK	805.83791	1087.69104	523.9847801	1057.621379	1099.548507	1105.903234	556.0497413	380.0792599	635.8253392
ENSG00000158555	GDPD5	chr11:75434640-75525903	-	protein_coding	-1.04295257	0.137238311	-7.599573076	2.97109E-14	6.15583E-13	Yes	OK	621.2513509	834.8949522	407.6077496	827.6123599	813.0577026	864.0147942	331.4203756	475.0990749	416.3037984
ENSG00000085276	MECOM	chr3:169083499-169663618	-	protein_coding	-1.042825704	0.187141337	-5.572396365	2.51259E-08	2.86407E-07	Yes	OK	240.4576518	325.1759526	155.7393511	314.0305782	347.3100816	314.1871979	154.6628419	114.023778	198.5314333
ENSG00000102409	BEX4	chrX:103215092-103217246	+	protein_coding	-1.042523596	0.141575252	-7.363741748	1.78825E-13	3.48752E-12	Yes	OK	276.5545382	372.4826649	180.6264116	390.7002511	346.509828	380.2379156	177.6781458	180.5376485	183.6634405
ENSG00000092621	PHGDH	chr1:119648411-119744226	+	protein_coding	-1.040634712	0.094666959	-10.99258624	4.14865E-28	1.70717E-26	Yes	OK	4577.005043	6157.004635	2997.00545	5841.598916	6225.173099	6404.241889	2659.648514	3336.779169	2994.588668
ENSG00000132846	ZBED3	chr5:77072072-77087323	-	protein_coding	-1.039942209	0.18286985	-5.68678877	1.2945E-08	1.53623E-07	Yes	OK	216.3340277	289.6550313	143.0130241	295.1257273	302.4958775	271.3434891	139.0124353	178.9539849	111.0726521
ENSG00000074935	TUBE1	chr6:112070777-112087529	-	protein_coding	-1.039621326	0.165942323	-6.264955854	3.72931E-10	5.20648E-09	Yes	OK	397.5725965	536.4098461	258.7353469	569.2460648	524.9663906	515.0170829	327.737927	202.7089386	245.7591751
ENSG00000111335	OAS2	chr12:112978395-113011723	+	protein_coding	-1.032503013	0.227558663	-4.537304799	5.69777E-06	4.58832E-05	Yes	OK	83.83180866	112.65446	55.00915734	122.8815306	100.8319592	114.2498901	57.99856573	55.4282254	51.6006809
ENSG00000125354	SEPT6	chrX:119615724-119693370	-	protein_coding	-1.03145049	0.106023002	-9.728553923	2.27808E-22	7.43993E-21	Yes	OK	1130.946127	1520.540783	741.3514712	1465.125942	1522.882685	1573.613721	731.8866628	668.306032	823.8617187
ENSG00000132530	XAF1	chr17:6755447-6775647	+	protein_coding	-1.03034919	0.198909578	-5.179987821	2.219E-07	2.23804E-06	Yes	OK	152.7951923	205.2574381	100.3329464	207.9533595	232.8738105	174.9451443	85.61693037	98.18714214	117.1947668
ENSG00000152582	SPEF2	chr5:35617844-35814611	+	protein_coding	-1.022543755	0.241818169	-4.228564631	2.35187E-05	0.000168239	Yes	OK	100.4249618	135.5948375	65.25508609	131.2836865	149.6474315	125.8533946	69.04591158	42.75891674	83.96042993
ENSG00000196730	DAPK1	chr9:87497228-87708633	+	protein_coding	-1.019807715	0.235687993	-4.326939622	1.51195E-05	0.000112661	Yes	OK	104.9704141	141.1747079	68.76612021	130.233417	142.4451487	150.8455581	92.9818276	53.84456182	59.4719712
ENSG00000126785	RHOJ	chr14:63204114-63293219	+	protein_coding	-1.014771947	0.160157637	-6.336082154	2.35681E-10	3.37038E-09	Yes	OK	258.9161504	347.0977273	170.7345735	328.7343511	345.7095743	366.8492566	203.4552861	150.4480404	158.3003939
ENSG00000056998	GYG2	chrX:2828788-2882820	+	protein_coding	-1.012646455	0.263826545	-3.8383039	0.000123887	0.000758906	Yes	OK	90.39830101	119.6208774	61.1757246	114.4793747	117.6372857	126.7459719	46.03060772	90.26882423	47.22774184
ENSG00000075651	PLD1	chr3:171600405-171810950	-	protein_coding	-1.012508554	0.201574085	-5.023009551	5.0868E-07	4.87362E-06	Yes	OK	160.0963442	214.3850445	105.8076438	256.2657561	220.8700058	166.0193716	105.8703978	101.3544693	110.1980643
ENSG00000095015	MAP3K1	chr5:56815574-56896152	+	protein_coding	-1.012359098	0.158366638	-6.39250232	1.63193E-10	2.37293E-09	Yes	OK	245.7932722	329.4691315	162.1174129	343.438124	351.3113498	293.6579208	162.9483513	144.113386	179.2905014
ENSG00000110318	CEP126	chr11:101915015-102001058	+	protein_coding	-1.010694948	0.16940978	-5.965977574	2.43174E-09	3.14673E-08	Yes	OK	272.7117378	365.8699368	179.5535388	351.8402799	390.5237784	355.2457522	216.3438563	137.7787317	184.5380283
ENSG00000179954	SSC5D	chr19:55488404-55519098	+	protein_coding	-1.010209793	0.112345552	-8.991987417	2.428E-19	6.80831E-18	Yes	OK	4186.292708	5591.926956	2780.65846	5415.189502	5579.368408	5781.222957	2349.402218	3281.350944	2711.222217
ENSG00000143382	ADAMTSL4	chr1:150549369-150560937	+	protein_coding	-1.007487307	0.160546952	-6.275343722	3.48863E-10	4.89121E-09	Yes	OK	853.7137528	1137.874853	569.5526528	1188.905065	1125.956877	1098.762615	454.7824043	747.4892112	506.386343
ENSG00000091592	NLRP1	chr17:5499427-5619424	-	protein_coding	-1.004763892	0.107265972	-9.367032913	7.46001E-21	2.31246E-19	Yes	OK	1281.609929	1708.862938	854.3569199	1684.632266	1654.124283	1787.832265	754.9019667	950.1981498	857.9706434
ENSG00000189298	ZKSCAN3	chr6:28349914-28369177	+	protein_coding	-1.003259429	0.248566604	-4.036179488	5.43287E-05	0.000360585	Yes	OK	100.1019153	134.3686789	65.83515163	135.4847645	132.8421049	134.7791673	92.9818276	45.92624391	58.59738339
ENSG00000172954	LCLAT1	chr2:30447226-30644225	+	protein_coding	-1.003253073	0.160326163	-6.257575529	3.91008E-10	5.45423E-09	Yes	OK	869.8425001	1162.55445	577.1305498	1116.43647	1233.190866	1138.036015	727.283602	426.0055038	578.1025436
ENSG00000145687	SSBP2	chr5:81413021-81751797	-	protein_coding	-1.000874702	0.101567103	-9.854319677	6.56598E-23	2.21922E-21	Yes	OK	751.5525143	1002.830925	500.2741034	1012.459791	988.3132506	1007.719734	523.8283159	484.6010564	492.392938
ENSG00000052795	FNIP2	chr4:158769138-158908049	+	protein_coding	-0.99998926	0.113253783	-8.829632255	1.0502E-18	2.84784E-17	Yes	OK	1548.257825	2066.204369	1030.311281	1914.641284	2131.075455	2152.896367	1163.653763	890.0189336	1037.261145
ENSG00000160886	LY6K	chr8:142700111-142705127	+	protein_coding	-0.99855339	0.134378633	-7.430894072	1.07866E-13	2.14689E-12	Yes	OK	378.0410514	502.7628131	253.3192898	470.5207325	544.1724781	493.5952285	245.8034452	277.141127	237.013297
ENSG00000120899	PTK2B	chr8:27311482-27459391	+	protein_coding	-0.997218108	0.195952913	-5.08907009	3.59824E-07	3.51079E-06	Yes	OK	169.9323931	225.3095921	114.555194	203.7522815	208.8662011	263.3102937	101.267337	140.9460589	101.4521862
ENSG00000079691	CARMIL1	chr6:25279078-25620530	+	protein_coding	-0.995646244	0.120594634	-8.256140491	1.50459E-16	3.57883E-15	Yes	OK	420.1800001	560.0915089	280.2684914	571.3466038	561.7780583	547.1498645	292.7546651	270.8064727	277.2443363
ENSG00000152409	JMY	chr5:79236189-79327215	+	protein_coding	-0.993283152	0.09992	-9.940784143	2.76641E-23	9.52617E-22	Yes	OK	848.659148	1130.659199	566.6590974	1135.341321	1089.14521	1167.491065	585.5093302	541.6129454	572.8550167
ENSG00000128510	CPA4	chr7:130293134-130324180	+	protein_coding	-0.99234551	0.200441028	-4.950810324	7.39051E-07	6.90005E-06	Yes	OK	164.4777812	217.7883365	111.1672259	194.2998561	229.6727959	229.3923576	83.77570606	137.7787317	111.9472399
ENSG00000101935	AMMECR1	chrX:110194186-110440233	-	protein_coding	-0.98681545	0.179448605	-5.499153652	3.81618E-08	4.24422E-07	Yes	OK	635.5186805	846.6704235	424.3669375	842.3161328	881.879516	815.8156218	544.0817833	288.2267721	440.7922572
ENSG00000065060	UHRF1BP1	chr6:34792015-34883138	+	protein_coding	-0.986353773	0.121019808	-8.15034987	3.62873E-16	8.43628E-15	Yes	OK	749.7661094	998.5302201	501.0019986	1045.018145	949.1008221	1001.471693	553.2879048	427.5891674	522.1289236
ENSG00000138131	LOXL4	chr10:98247690-98268250	-	protein_coding	-0.982335895	0.117864743	-8.334433811	7.78699E-17	1.89047E-15	Yes	OK	850.5035504	1127.733692	573.2734091	1066.023535	1082.743181	1234.43436	522.9077037	639.8000875	557.1124361
ENSG00000179388	EGR3	chr8:22687659-22693302	-	protein_coding	-0.980312842	0.24400755	-4.017551268	5.8806E-05	0.000387644	Yes	OK	74.66332819	99.16639916	50.16025722	82.97128989	105.633481	108.8944265	47.87183203	47.50990749	55.09903214
ENSG00000148677	ANKRD1	chr10:90912096-90921276	-	protein_coding	-0.979936719	0.19572192	-5.006780649	5.53479E-07	5.28132E-06	Yes	OK	422.1004381	561.9539401	282.246936	558.7433699	589.7869358	537.3315146	362.7211889	180.5376485	303.4819707
ENSG00000197208	SLC22A4	chr5:132294443-132344206	+	protein_coding	-0.978602499	0.136358214	-7.176703702	7.14122E-13	1.3327E-11	Yes	OK	292.5378892	387.9788324	197.096946	377.0467477	397.7260612	389.1636883	205.2965104	197.9579479	188.0363795
ENSG00000159023	EPB41	chr1:28887091-29120046	+	protein_coding	-0.976865758	0.146443	-6.670621039	2.54723E-11	4.02375E-10	Yes	OK	255.8487794	339.6949626	172.0025962	358.1418969	328.9042478	332.0387432	186.8842674	164.7010126	164.4225086
ENSG00000110455	ACCS	chr11:44065925-44084222	+	protein_coding	-0.975740964	0.189367267	-5.152637932	2.56848E-07	2.5638E-06	Yes	OK	250.5806365	331.1424137	170.0188594	276.2208765	344.109067	373.0972975	129.8063138	202.7089386	177.5413258

ENSG00000134986	NREP	chr5:111662621-111997464	-	protein_coding	-0.974982954	0.07967174	-12.2375005	1.95975E-34	1.03389E-32	Yes	OK	4109.861141	5448.706333	2771.015948	5680.907684	5525.751414	5139.459902	2933.070324	2715.983045	2663.994475
ENSG00000136235	GPNMB	chr7:23235967-23275108	+	protein_coding	-0.974035627	0.07166352	-13.59179156	4.47979E-42	3.13776E-40	Yes	OK	11821.18472	15669.36603	7973.003403	15713.08187	16166.72412	15128.29209	8387.697339	7427.382204	8103.930664
ENSG00000110628	SLC22A18	chr11:2899721-2925246	+	protein_coding	-0.971657493	0.239747417	-4.052838218	5.06E-05	0.000339257	Yes	OK	150.0165674	197.5810623	102.4520724	172.2441967	184.8585918	235.6403984	68.12529943	137.7787317	101.4521862
ENSG00000179348	GATA2	chr3:128479427-128493185	-	protein_coding	-0.970907106	0.146493704	-6.627637106	3.41103E-11	5.31178E-10	Yes	OK	832.7863399	1100.50498	565.0677003	1074.425691	1113.152819	1113.936429	459.3854651	711.0649487	524.7526871
ENSG00000163071	SPATA18	chr4:52051331-52097292	+	protein_coding	-0.970811267	0.130277992	-7.451843959	9.20446E-14	1.84089E-12	Yes	OK	767.7822532	1018.970432	516.5940742	1081.777577	945.0995539	1030.034166	555.1291291	424.4218402	570.2312533
ENSG00000091136	LAMB1	chr7:107923799-108003255	-	protein_coding	-0.967496438	0.056434282	-17.14377166	6.99617E-66	8.10964E-64	Yes	OK	14014.70163	18546.13411	9483.269145	18673.79157	18581.08937	18383.52139	9468.496009	9387.95772	9593.353708
ENSG00000157404	KIT	chr4:54657918-54740715	+	protein_coding	-0.965365887	0.126860276	-7.60967829	2.74779E-14	5.7179E-13	Yes	OK	460.2005184	609.8377382	310.5632986	645.9157378	618.596067	565.0014099	328.6585391	280.3084542	322.7229026
ENSG00000129474	AJUBA	chr14:22971174-22982642	-	protein_coding	-0.964819798	0.097799753	-9.865258055	5.88828E-23	1.99426E-21	Yes	OK	2007.650659	2652.966045	1362.335272	2524.847859	2668.845904	2765.204372	1385.521292	1482.309114	1219.17541
ENSG00000099365	STX1B	chr16:30989256-31010661	-	protein_coding	-0.963254829	0.235336921	-4.093088432	4.25665E-05	0.000290122	Yes	OK	86.97362688	114.3434714	59.60378239	119.7307221	99.23145189	124.0682401	55.23672927	72.84852482	50.72609308
ENSG00000136732	GYPC	chr2:126655933-126696670	+	protein_coding	-0.96319734	0.112539631	-8.558739117	1.14109E-17	2.93015E-16	Yes	OK	905.6874497	1195.370113	616.0047865	1223.563959	1169.170574	1193.375806	550.5260684	698.3956401	599.0926511
ENSG00000161714	PLCD3	chr17:45108967-45133354	-	protein_coding	-0.961291683	0.1417679	-6.780742912	1.19559E-11	1.95427E-10	Yes	OK	2697.972201	3562.23525	1833.709151	3474.291481	3439.490163	3772.924107	1464.693938	2332.736458	1703.697057
ENSG00000139192	TAPBPL	chr12:6451690-6466517	+	protein_coding	-0.960462637	0.194213788	-4.945388522	7.59921E-07	7.07482E-06	Yes	OK	268.7037049	353.6729468	183.734463	395.9515986	324.1027259	340.9645159	133.4887624	237.5495374	180.1650892
ENSG00000179715	PCED1B	chr12:47079603-47236662	+	protein_coding	-0.95743277	0.246384739	-3.885925619	0.000101941	0.000636549	Yes	OK	192.7316249	252.8309	132.6323497	207.9533595	256.8814198	293.6579208	94.82305191	196.3742843	106.699713
ENSG00000052126	PLEKHA5	chr12:19129752-19376400	+	protein_coding	-0.954747618	0.111452192	-8.56643198	1.06741E-17	2.75385E-16	Yes	OK	840.5498673	1109.570033	571.5297012	1121.687818	1155.566262	1051.45602	530.272601	551.1149269	633.2015757
ENSG00000105928	GSDME	chr7:24698351-24758113	-	protein_coding	-0.954613257	0.117767884	-8.105887841	5.23617E-16	1.19539E-14	Yes	OK	869.2924653	1145.809994	592.7749368	1218.312611	1097.147746	1121.969624	626.016265	639.8000875	512.5084577
ENSG00000136010	ALDH1L2	chr12:105019784-105084577	-	protein_coding	-0.952844099	0.095846258	-9.941380274	2.7499E-23	9.48917E-22	Yes	OK	7190.149481	9484.886749	4895.412213	9345.297942	9692.672139	9416.690164	5459.230076	4199.875822	5027.130742
ENSG00000005020	SKAP2	chr7:26667062-26995239	-	protein_coding	-0.9524171	0.136050424	-7.000471392	2.55103E-12	4.51019E-11	Yes	OK	864.0192278	1141.168521	586.8699349	1155.296442	1097.147746	1171.061374	707.0301346	479.8500656	573.7296045
ENSG00000145934	TENM2	chr5:167284799-168264157	+	protein_coding	-0.952387725	0.074747784	-12.74135067	3.48312E-37	2.06231E-35	Yes	OK	7203.275093	9495.282502	4911.267684	9518.592409	9508.613801	9458.641296	4592.934039	5305.273003	4835.596011
ENSG00000151388	ADAMTS12	chr5:33523535-33892192	-	protein_coding	-0.943843763	0.10124442	-9.322427474	1.13708E-20	3.48537E-19	Yes	OK	981.2160139	1290.619718	671.8123094	1342.244411	1338.024093	1191.590651	656.3964661	695.2283129	663.8121492
ENSG00000170962	PDGFD	chr11:103907186-104164379	-	protein_coding	-0.941747758	0.167683994	-5.616205448	1.95197E-08	2.25627E-07	Yes	OK	571.7166903	754.065757	389.3676236	772.9983463	716.2270116	772.971913	457.5442408	272.3901363	438.1684937
ENSG00000142065	ZFP14	chr19:36334453-36379199	-	protein_coding	-0.941596181	0.320323098	-2.939520088	0.00328721	0.013927277	Yes	OK	95.42579619	126.6007732	64.25081921	142.836651	120.0380466	116.9276219	94.82305191	25.33861733	72.59078838
ENSG00000133106	EPSTI1	chr13:42886388-42992271	-	protein_coding	-0.941472549	0.220681784	-4.266199643	1.98831E-05	0.000144429	Yes	OK	98.96109056	129.9776769	67.94450423	135.4847645	146.4464169	108.0018493	62.6016265	71.26486123	69.96702494
ENSG00000164463	CREBRF	chr5:173056352-173139284	+	protein_coding	-0.941056892	0.159974983	-5.882525352	4.04053E-09	5.03461E-08	Yes	OK	623.6222871	822.0356379	425.2089362	841.2658933	793.8516151	830.9894354	528.4313767	315.149053	432.046379
ENSG00000151917	BEND6	chr6:56955126-57027342	+	protein_coding	-0.940673428	0.125336563	-7.505179687	6.13444E-14	1.24351E-12	Yes	OK	840.6512837	1107.047632	574.2549358	1105.933775	1138.760936	1076.448184	672.9674849	490.9357107	558.8616117
ENSG00000184898	RBM43	chr2:151247940-151261879	-	protein_coding	-0.940323948	0.19709752	-4.770856323	1.83444E-06	1.60185E-05	Yes	OK	371.2502449	489.9228147	252.5776751	474.7218105	512.1623323	482.8843013	302.8813988	158.3663583	296.4852682
ENSG00000160469	BRSK1	chr19:55282072-55312533	+	protein_coding	-0.940303779	0.215119298	-4.371080554	1.23633E-05	9.34774E-05	Yes	OK	102.5055035	135.2624908	69.74851611	136.535034	126.4400758	142.8123627	63.52223866	61.76287974	83.96042993
ENSG00000103175	WFDC1	chr16:84294646-84329851	+	protein_coding	-0.937125953	0.148779371	-6.298762711	3.00031E-10	4.24635E-09	Yes	OK	293.2917473	384.1609768	202.4225177	375.9964782	387.3227638	389.1636883	206.2171226	229.6312195	171.4192111
ENSG00000188906	LRRK2	chr12:40196744-40369285	+	protein_coding	-0.9369218	0.160867971	-5.824166185	5.73984E-09	7.0296E-08	Yes	OK	236.7917343	311.9910342	161.5924345	321.3824646	336.1065306	278.4841072	173.9956972	139.3623953	171.4192111
ENSG00000177409	SAMD9L	chr7:93130055-93148369	-	protein_coding	-0.928694744	0.155918833	-5.956270489	2.58059E-09	3.31848E-08	Yes	OK	1115.685954	1465.199858	766.1720495	1507.136721	1422.850979	1465.611872	923.3739909	549.5312633	825.6108943
ENSG00000089335	ZNF302	chr19:34677639-34686397	+	protein_coding	-0.928091481	0.372825127	-2.489347991	0.012797763	0.04341572	Yes	OK	567.1758808	744.256619	390.0951426	763.5459209	734.6328454	734.5910906	505.4160728	207.4599294	457.4094256
ENSG00000174899	PQLC2L	chr3:157543246-157677749	+	protein_coding	-0.924996557	0.20836759	-4.439253511	9.02714E-06	7.0022E-05	Yes	OK	105.321903	138.5261778	72.11762828	145.9874594	131.2415977	138.3494764	69.04591158	63.34654332	83.96042993
ENSG00000155893	PXYLP1	chr3:141228726-141367753	+	protein_coding	-0.923224093	0.196856811	-4.689825503	2.73438E-06	2.31284E-05	Yes	OK	162.1704056	213.6209535	110.7198577	235.2603663	205.6651866	199.9373078	115.9971315	82.35050631	133.8119352
ENSG00000152661	GJA1	chr6:121435692-121449727	+	protein_coding	-0.920510921	0.083326663	-11.04701526	2.26625E-28	9.44365E-27	Yes	OK	4480.919104	5865.890866	3095.947342	5927.721015	5930.679758	5739.271825	3394.297014	2864.847422	3028.697592
ENSG00000170190	SLC16A5	chr17:75087727-75106162	+	protein_coding	-0.919994203	0.127441656	-7.218944195	5.23927E-13	9.88973E-12	Yes	OK	417.1606386	545.0319155	289.2893617	555.5925614	530.5681661	548.9350191	258.6920154	313.5653894	295.6106804
ENSG00000116678	LEPR	chr1:65420652-65641559	+	protein_coding	-0.918578653	0.186051412	-4.937230207	7.92399E-07	7.35155E-06	Yes	OK	464.2830853	609.2232509	319.3429197	646.9660073	581.7843994	598.919346	394.9426143	210.6272565	352.4588882
ENSG00000147394	ZNF185	chrX:152914442-152973480	+	protein_coding	-0.916965798	0.166155682	-5.518714674	3.41488E-08	3.81854E-07	Yes	OK	185.5413758	241.9295992	129.1531523	245.7630612	256.8814198	223.1443167	123.3620287	142.5297225	121.5677058
ENSG00000196151	WDSUB1	chr2:159235793-159286799	-	protein_coding	-0.916202473	0.204528837	-4.479576008	7.47915E-06	5.9053E-05	Yes	OK	156.0309478	205.1749916	106.886904	216.3555154	184.0583382	215.1111213	106.7910099	79.18317915	134.686523
ENSG00000181481	RNF135	chr17:30968785-30999911	+	protein_coding	-0.915243556	0.14373325	-6.367653662	1.91942E-10	2.7738E-09	Yes	OK	342.4291185	446.9635211	237.8947159	387.5494427	471.3493965	481.9917241	222.7881414	248.6351825	242.2608239
ENSG00000158023	WDR66	chr12:121917862-122003927	+	protein_coding	-0.908489874	0.20959074	-4.334589754	1.46032E-05	0.000109159	Yes	OK	106.1245802	137.9119216	74.3372387	147.0377289	140.8446414	125.8533946	73.64897236	85.51783348	63.84491026
ENSG00000105755	ETHE1	chr19:43506719-43527244	-	protein_coding	-0.907181188	0.134277986	-6.75599342	1.4186E-11	2.30505E-10	Yes	OK	697.3027441	907.4795725	487.1259158	868.5728701	913.0894081	940.7764392	412.4342452	582.7881985	466.1553037
ENSG00000231924	PSG1	chr19:42866464-42879822	-	protein_coding	-0.905202561	0.172228103	-5.255835413	1.47354E-07	1.52161E-06	Yes	OK	154.0378212	200.5367813	107.5388612	198.500934	204.0646793	199.0447305	110.4734585	112.4401144	99.70301055
ENSG00000114423	CBLB	chr3:105655461-105869552	-	protein_coding	-0.904843855	0.108677748	-8.325934892	8.36658E-17	2.02223E-15	Yes	OK	1770.407027	2310.719711	1230.094342	2356.804741	2282.323393	2293.030998	1354.220479	1037.299647	1298.762901
ENSG00000111912	NCOA7	chr6:125781161-125932030	+	protein_coding	-0.899956139	0.125411164	-7.176044848	7.1757E-13	1.33762E-11	Yes	OK	722.3419799	942.353325	502.3306348	929.4885007	921.0919445	976.4795299	577.2238208	426.0055038	503.7625796
ENSG00000104870	FCGRT	chr19:49506816-49526333	+	protein_coding	-0.89645621	0.160292009	-5.592644436	2.23637E-08	2.56699E-07	Yes	OK	1393.028066	1810.047325	976.0088074	1730.844123	1790.967656	1908.330196	735.5691114	1273.265521	919.1917902
ENSG00000187608	ISG15	chr1:1001138-1014541	+	protein_coding	-0.894454468	0.205322964	-4.356329425	1.32262E-05	9.95568E-05	Yes	OK	408.5228925	529.6402024	287.4055826	536.6877106	496.1572594	556.0756372	188.7254917	392.7485686	280.7426876
ENSG00000196083	IL1RAP	chr3:190514051-190659750	+	protein_coding	-0.890726132	0.193220356	-4.609897991	4.02867E-06	3.31891E-05	Yes	OK	552.3547228	719.4735868	385.2358588	681.6249005	761.8414693	714.9543907	482.4007689	243.8841918	429.4226156
ENSG00000176928	GCNT4	chr5:75025346-75030899	-	protein_coding	-0.886909748	0.177301825	-5.002259547	5.66622E-07	5.39377E-06	Yes	OK	284.8751988	371.326822	198.4235755	419.0575274	359.3138863	335.6090523	234.7560994	148.8643768	211.6502505
ENSG00000169330	KIAA1024	chr15:79432516-79472290	+	protein_coding	-0.886011879	0.206918055	-4.281945722	1.85266E-05	0.000135533	Yes	OK	111.6593005	145.2320215	78.08657937	151.2388069	141.644895	142.8123627	93.90243976	71.26486123	69.09243713
ENSG00000164989	CCDC171	chr9:15553086-16061663	+	protein_coding	-0.885765047	0.219253655	-4.039910059	5.34717E-05	0.000356189	Yes	OK	100.1493823	130.848349	69.45041554	134.434495	122.4388076	135.6717445	78.25203313	52.26089824	77.83831525
ENSG00000105227	PRX	chr19:40393768-40413366	-	protein_coding	-0.885387602	0.161631585	-5.477813022	4.30615E-08	4.76339E-07	Yes	OK	190.5362749	246.9298075	134.1427423	248.9138697	262.4831953	229.3923576	136.2505989	139.3623953	126.8152327
ENSG00000205593	DENND6B	chr22:50309030-50327060	-	protein_coding	-0.885037487	0.259291436	-3.413292392	0.00064183	0.003344263	Yes	OK	106.7350629	137.3888615	76.08126425	130.233417	124.8395685	157.093599	49.71305634	107.6891236	70.84161276
ENSG00000151414	NEK7	chr1:198156963-198322420	+	protein_coding	-0.883100521	0.217366649	-4.062723165	4.85035E-05	0.000326264	Yes	OK	18734.97421	24297.61068	13172.33774	25021.62038	23677.90483	24193.30682	16316.00921	9532.071106	13668.93291
ENSG00000099889	ARVCF	chr22:19969896-20016808	-	protein_coding	-0.882133697	0.232182543	-3.799311028	0.000145099	0.000878062	Yes	OK	105.2557433	135.6613252	74.8501613	135.4847645	122.4388076	149.0604036	54.31611711	95.01981498	75.21455182
ENSG00000253846	PCDHGA10	chr5:141412987-141512979	+	protein_coding	-0.881682711	0.136058534	-6.480172073	9.16181E-11	1.3635E-09	Yes	OK	329.0492407	425.9331976	232.1652838	431.6607613	425.7349387	420.4038927	243.0416088	247.0515189	206.4027236
ENSG00000128602	SMO	chr7:129188872-129213545	+	protein_coding	-0.880661182	0.209068392	-4.212311463	2.52771E-05	0.000179957	Yes	OK	223.6677675	288.590941	158.7445939	263.6176426	273.6867463	328.4684342	110.4734585	207.4599294	158.3003939
ENSG00000139874	SSTR1	chr14:38207999-38213067	+	protein_coding	-0.87931919	0.076166133	-11.54475292	7.84666E-31	3.69017E-29	Yes	OK	3245.492114	4203.556952	2287.427276	4108.654254	4299.762831	4202.253772	2168.041624	2381.830029	2312.410174
ENSG00000128849	CGNL1	chr15:57375967-57550727	+	protein_coding	-0.878018288	0.200915916	-4.370078308	1.24202E-05	9.38212E-05	Yes	OK	107.356041	139.3843879	75.32769414	134.434495	148.0469242	135.6717445	79.17264528	68.09753407	78.71290306
ENSG00000172465	TCEAL1	chrX:103628704-103630953	+	protein_coding	-0.87743158	0.154514721	-5.678627751	1.3578E-08	1.60555E-07	Yes	OK	482.5807913	625.9852167	339.1763658	619.6590005	597.7894723	660.5071774	424.4022032	291.3940993	301.7327951
ENSG00000116016	EPAS1	chr2:46293667-46386703	+	protein_coding	-0.874299028	0.063104244	-13.85483724	1.18926E-43	8.62345E-42	Yes	OK	13809.0843	17867.38108	9750.787524	17882.93865	17600.77865	18118.42594	9473.099069	10238.38506	9540.878439
ENSG00000164125	FAM198B	chr4:158124474-158173318	-	protein_coding	-0.874104608	0.110282411	-7.926056411	2.26215E-15	5.01144E-14	Yes	OK	1035.71596	1342.14828	729.2836393	1329.641177	1335.623332	1361.180332	812.9005324	635.0490968	739.9012888
ENSG00000071282	LMCD1	chr3:8501707-8574673	+	protein_coding	-0.874072125	0.111747495	-7.821849842	5.20527E-15	1.13033E-13	Yes	OK	1973.31752	2550.911775	1395.723265	2514.345165	2562.412169	2575.977992	1199.557637	1623.255173	1364.356986
ENSG00000135473	PAN2	chr12:56316223-56334053	-	protein_coding	-0.87234579	0.123044068	-7.089702115	1.34401E-12	2.46078E-11	Yes	OK	393.9617358	509.1425989	278.7808727	486.2747749	522.5656297	518.587392	271.5805856	286.6431085	278.1189242
ENSG00000182010	RTKN2	chr10:62183035-62268707	-	protein_coding	-0.871951521	0.211383574	-4.124972934	3.70778E-05	0.000256537	Yes	OK	142.93974	185.3383664	100.5411136	203.7522815	172.8547872	179.4080306	130.7269259	88.68516065	82.21125431
ENSG00000117226	GBP3	chr1:89006666-89022894	-	protein_coding	-0.870756026	0.118105775	-7.372679531	1.67232E-13	3.27695E-12	Yes	OK	820.7957404	1062.726495	578.8649858	995.6554787	1049.132528	1143.391479	636.1429987	508.3560101	592.0959486
ENSG00000185187	SIGIRR	chr11:405716-417455	-	protein_coding	-0.870587567	0.196524413	-4.429920717	9.42677E-06	7.27448E-05	Yes	OK	206.9572593	266.7316144	147.1829043	198.500934	300.8953702	300.7985389	135.3299867	166.2846762	139.9340499
ENSG00000152580	IGSF10	chr3:151425384-151458709	-	protein_coding	-0.869995612	0.271329061	-3.206422517	0.001343965	0.006430716	Yes	OK	83.65714816	108.9641985	58.35009785	91.37344583	124.8395685	110.6795811	75.49019667	34.84059883	64.71949807
ENSG00000173706	HEG1	chr3:124965710-125055958	-	protein_coding	-0.866649732	0.05539264	-15.64557553	3.56135E-55	3.31185E-53	Yes	OK	16175.2483	20892.8333	11457.6633	21090.46167	20663.34935	20924.68887	11491.08091	11438.80206	11443.10693
ENSG00000124788	ATXN1	chr6:16299112-16761491	-	protein_coding	-0.866357642	0.080425925	-10.77211914	4.66147E-27	1.86685E-25	Yes	OK	2211.844593	2855.841588	1567.847598	2893.492451	2793.685472	2880.34684	1486.788629	1623.255173	1593.498993
ENSG00000213654	GPSM3	chr6:32190766-32195523	-	protein_coding	-0.865355123	0.167888092	-5.154356777	2.54503E-07	2.54194E-06	Yes	OK	172.0491754	222.0009844	122.0973664	207.9533595	238.475586	219.5740076	115.0765193	123.5257595	127.6898205
ENSG00000169239	CA5B	chrX:15688830-15788409	+	protein_coding	-0.862402614	0.170615718	-5.054649256	4.31182E-07	4.17486E-06	Yes	OK	296.5376545	384.150192	208.9251171	401.2029461	358.5136326	392.7339974	255.0095668	158.3663583	213.3994261
ENSG00000165338	HECTD2	chr10:91409280-91514829	+	protein_coding	-0.862297451	0.194996858	-4.422109462	9.77419E-06	7.51791E-05	Yes	OK	526.5909553	681.1171302	372.0647804	667.9713971	720.2282798	655.1517138	493.4481148	242.3005282	380.4456981
ENSG00000185697	MYBL1	chr8:66562175-66614247	-	protein_coding	-0.860258756	0.140370884	-6.128470047	8.87281E-10	1.20203E-08	Yes	OK	2245.229536	2897.976146	1592.482926	2994.318323	2850.503481	2849.106636	1920.396954	1198.833332	1658.218491
ENSG00000111199	TRPV4	chr12:109783085-109833401	-	protein_coding	-0.85926884	0.219400933	-3.916432024	8.98691E-05	0.000568503	Yes	OK	132.213798	171.1288989	93.29869709	203.7522815	163.2517434	146.3826718	81.93448175	80.76684273	117.1947668
ENSG00000056558	TRAF1	chr9:120902393-120929173	-	protein_coding	-0.858528193	0.240396539	-3.571300144	0.000355214	0.001970615	Yes	OK	74.08081268	94.95352867	53.20809669	86.12209837	103.2327201	95.50576755	50.6336685	61.76287974	47.22774184
ENSG00000137801	THBS1	chr15:39581079-39599466	+	protein_coding	-0.85792295	0.056638197	-15.14742704	7.87913E-52	6.79008E-50	Yes	OK	231837.5665	298804.4315	164870.7015	303353.5385	294251.6645	298808.0916	159725.2882	176117.6434	158769.173
ENSG00000187735	TCEA1	chr8:53966552-54022529	-	protein_coding	-0.857457292	0.177332851	-4.835298645	1.32946E-06	1.188E-05	Yes	OK	1027.254211	1326.050888	728.4575337	1338.043933	1316.417245	1323.692087	831.3127755	475.0990749	878.9607509
ENSG00000187605	TET3	chr2:73986404-74108176	+	protein_coding	-0.857349455	0.14846382	-5.774803974	7.70427E-09	9.29028E-08	Yes	OK	294.7768355	380.2983075	209.2553635	371.7954003	381.7209883	387.3785338	185.9636552	199.5416115	242.2608239
ENSG00000151702	FLI1	chr11:128686535-128813267	+	protein_coding	-0.854458919	0.123994364	-6.891110946	5.53583E-12	9.35522E-11	Yes	OK	442.1379826	568.7605231	315.5154421	579.7487597	532.9689271	593.5638824	318.5318054	334.153016	293.8615048
ENSG00000154319	FAM167A	chr8:11421463-11474715	-	protein_coding	-0.85324012	0.147034236	-5.803003045	6.51376E-09	7.93608E-08	Yes	OK	581.3532958	746.3850094	416.3215822	784.5513107	706.6239679	747.9797496	366.4036375	517.8579916	364.7031175
ENSG00000115295	CLIP4	chr2:29097705-29189643	+	protein_coding	-0.849328952	0.094880588	-8.951556565	3.50504E-19	9.71263E-18	Yes	OK	1380.534794	1776.305171	984.7644176	1740.296549	1837.382367	1751.236597	1069.751323	939.1125047	945.4294246

ENSG00000188677	PARVB	chr22:43999211-44172949	+	protein_coding	-0.84844433	0.123921259	-6.84664063	7.56043E-12	1.25702E-10	Yes	OK	1595.729136	2049.166334	1142.291937	2044.874702	2101.46607	2001.158232	991.4992904	1384.121972	1051.25455
ENSG00000176046	NUPR1	chr16:28532708-28539174	-	protein_coding	-0.847146153	0.101946268	-8.309731859	9.59186E-17	2.30485E-15	Yes	OK	5093.826452	6544.66437	3642.988534	6558.932979	6416.43372	6658.62641	3244.237232	4253.720384	3431.007986
ENSG00000108823	SGCA	chr17:50164214-50175931	+	protein_coding	-0.845305599	0.21774195	-3.882143977	0.00010354	0.000645553	Yes	OK	98.37618216	125.7508743	71.00149003	118.6804526	118.4375393	140.1346309	62.6016265	83.9341699	66.4686737
ENSG00000186470	BTN3A2	chr6:26365159-26378320	+	protein_coding	-0.844615748	0.126200321	-6.692659242	2.19151E-11	3.50216E-10	Yes	OK	469.8406544	604.9482688	334.73304	607.0557665	594.5884577	613.2005823	371.0066982	292.9777628	340.2146588
ENSG00000164342	TLR3	chr4:186069152-186088069	+	protein_coding	-0.84203456	0.22648962	-3.717762253	0.000200995	0.001179039	Yes	OK	87.1700624	112.6045921	61.7355327	115.5296442	108.034242	114.2498901	63.52223866	49.09357107	72.59078838
ENSG00000204271	SPIN3	chrX:56818298-56995827	-	protein_coding	-0.841935237	0.154614007	-5.445400781	5.16889E-08	5.67199E-07	Yes	OK	250.5320196	322.8786313	178.1854078	331.8851596	306.4971457	330.2535887	192.4079403	148.8643768	193.2839064
ENSG00000029534	ANK1	chr8:41653220-41896762	-	protein_coding	-0.840306911	0.187225996	-4.488195715	7.18289E-06	5.7006E-05	Yes	OK	143.3993327	183.0829427	103.7157227	179.5960832	176.8560554	192.7966896	96.66427622	123.5257595	90.95713243
ENSG00000166147	FBN1	chr15:48408306-48645849	-	protein_coding	-0.838106922	0.0495485	-16.91487968	3.4953E-64	3.78504E-62	Yes	OK	81052.51007	103955.8456	58149.17451	104941.8774	104721.9921	102203.6674	59406.18172	57517.07767	57524.26415
ENSG00000140105	WARS	chr14:100333788-100376805	-	protein_coding	-0.837967719	0.064249296	-13.04244206	7.01728E-39	4.44248E-37	Yes	OK	6554.14857	8405.309412	4702.987728	8184.750153	8480.287868	8550.890216	4592.934039	4739.905104	4776.12404
ENSG00000112246	SIM1	chr6:100385015-100464929	-	protein_coding	-0.837019109	0.105777135	-7.913043861	2.51171E-15	5.5271E-14	Yes	OK	721.198946	925.0880704	517.3098216	990.4041312	892.2828133	892.5772668	521.0664794	513.1070009	517.7559846
ENSG00000198093	ZNF649	chr19:51889224-51905040	-	protein_coding	-0.836619915	0.278938158	-2.999302498	0.002705985	0.011823869	Yes	OK	130.4816027	168.5591028	92.40410272	191.1490476	167.2530116	147.275249	109.5528464	44.34258032	123.3168815
ENSG00000140545	MFGE8	chr15:88898683-88913411	-	protein_coding	-0.83605888	0.110042142	-7.597624555	3.01616E-14	6.23693E-13	Yes	OK	18998.8714	24351.34872	13646.39408	23699.33109	24296.50089	25058.21419	11723.07517	16489.10523	12727.00184
ENSG00000169604	ANTXR1	chr2:69013178-69249327	+	protein_coding	-0.83163264	0.059710842	-13.9276656	4.30187E-44	3.17528E-42	Yes	OK	14242.43635	18239.25894	10245.61377	18503.64792	17976.09761	18238.03129	10527.19999	9927.987002	10281.65432
ENSG00000239713	APOBEC3G	chr22:39077005-39087743	+	protein_coding	-0.830415279	0.33351154	-2.489914681	0.012777376	0.043379593	Yes	OK	72.10713482	93.19285987	51.02140978	80.87075091	111.2352566	87.47257214	60.76040219	20.58762658	71.71620057
ENSG00000166123	GPT2	chr16:46884378-46931297	+	protein_coding	-0.83003315	0.112479716	-7.379402996	1.59001E-13	3.11937E-12	Yes	OK	1125.090063	1438.191162	811.9889634	1451.472438	1402.844638	1460.256408	707.0301346	921.6922053	807.2445503
ENSG00000185305	ARL15	chr5:53883945-54310582	-	protein_coding	-0.830009678	0.209212135	-3.967311353	7.2688E-05	0.000467909	Yes	OK	239.1623427	307.7024598	170.6222256	321.3824646	316.1001895	285.6247254	208.9789591	106.1054601	196.7822577
ENSG00000206527	HACD2	chr3:123490820-123585185	-	protein_coding	-0.829526649	0.100339417	-8.267206201	1.37135E-16	3.26662E-15	Yes	OK	2978.742847	3814.62005	2142.865644	3933.259249	3725.180714	3785.420188	2428.574863	1886.143327	2113.878741
ENSG00000054598	FOXC1	chr6:1609972-1613897	+	protein_coding	-0.826094946	0.140205386	-5.892034338	3.8147E-09	4.78217E-08	Yes	OK	581.6354087	741.7202936	421.5505238	744.64107	745.0361428	735.4836678	355.3562916	513.1070009	396.1882787
ENSG00000080298	RFX3	chr9:3218297-3526004	-	protein_coding	-0.825161459	0.308762753	-2.672477338	0.007529346	0.028153802	Yes	OK	104.360878	134.4234854	74.29827063	147.0377289	100.0317055	156.2010217	108.6322342	36.42426241	77.83831525
ENSG00000006025	OSBPL7	chr17:47807372-47821834	-	protein_coding	-0.824290773	0.207396757	-3.97446318	7.05382E-05	0.000455139	Yes	OK	139.499337	178.4509056	100.5477685	166.9928493	164.8522507	203.5076168	81.01386959	98.18714214	122.4422937
ENSG00000051108	HERPUD1	chr16:56932048-56944863	+	protein_coding	-0.823809811	0.06992869	-11.78071269	4.90764E-32	2.40416E-30	Yes	OK	3899.250747	4982.088344	2816.41315	4870.099636	5092.013938	4984.151458	2743.42422	2887.018712	2818.796517
ENSG00000138411	HECW2	chr2:196189099-196593692	-	protein_coding	-0.821222946	0.129985984	-6.317780727	2.65346E-10	3.78147E-09	Yes	OK	730.929161	935.4130748	526.4452472	976.7506278	869.0754577	960.4131391	602.080349	440.2584761	536.9969165
ENSG00000090339	ICAM1	chr19:10270835-10286615	+	protein_coding	-0.820954698	0.189036699	-4.342832384	1.40657E-05	0.000105525	Yes	OK	186.2824526	237.4335719	135.1313333	218.4560544	214.4679767	279.3766845	110.4734585	148.8643768	146.0561646
ENSG00000143847	PPFIA4	chr1:203026498-203078740	+	protein_coding	-0.818242361	0.199517839	-4.10109876	4.11193E-05	0.00028119	Yes	OK	142.7060362	181.6659594	103.7461129	170.1436578	170.4540262	204.4001941	81.93448175	115.6074416	113.6964155
ENSG00000122042	UBL3	chr13:29764371-29850684	-	protein_coding	-0.817350938	0.100602091	-8.124591921	4.4887E-16	1.0319E-14	Yes	OK	3514.420592	4485.741014	2543.10017	4462.595073	4370.185151	4624.442819	2931.2291	2320.067149	2378.00426
ENSG00000184271	POU6F1	chr12:51186936-51217708	-	protein_coding	-0.817200227	0.145834304	-5.603621411	2.09919E-08	2.41796E-07	Yes	OK	237.3306215	303.0513294	171.6099137	301.4273443	310.498414	297.2282298	166.6308	166.2846762	181.9142649
ENSG00000154359	LONRF1	chr8:12721894-12756073	-	protein_coding	-0.814188875	0.157394177	-5.172928834	2.30453E-07	2.3172E-06	Yes	OK	366.8220979	468.9796435	264.6645523	437.9623783	467.3481282	501.6284239	318.5318054	212.2109201	263.2509314
ENSG00000167555	ZNF528	chr19:52397849-52418412	+	protein_coding	-0.810389084	0.157542365	-5.143943884	2.6903E-07	2.67567E-06	Yes	OK	450.5735604	575.6529337	325.4941872	569.2460648	618.596067	539.1166691	364.5624132	245.4678554	366.4522931
ENSG00000131370	SH3BP5	chr3:15254853-15341368	-	protein_coding	-0.809624009	0.162640272	-4.978004508	6.42432E-07	6.0529E-06	Yes	OK	204.3662011	260.7588576	147.9735447	290.9246494	236.0748251	255.2770983	147.2979447	140.9460589	155.6766305
ENSG00000101463	SYNDIG1	chr20:24469199-24666616	+	protein_coding	-0.808705033	0.232193955	-3.482885817	0.00049604	0.002657818	Yes	OK	201.9459736	255.9557655	147.9361817	238.4111748	261.6829417	267.77318	89.29937898	197.9579479	156.5512183
ENSG00000260916	CCPG1	chr15:55340032-55408510	-	protein_coding	-0.807321279	0.165724698	-4.871460256	1.10776E-06	1.00351E-05	Yes	OK	771.7181569	984.2714498	559.164864	1012.459791	983.5117288	956.84283	678.4911578	391.164905	607.8385292
ENSG00000108582	CPD	chr17:30378905-30469989	+	protein_coding	-0.80605511	0.093867434	-8.587164672	8.91418E-18	2.31067E-16	Yes	OK	6396.561191	8141.598558	4651.523824	8347.541925	8114.571953	7962.681797	5056.001952	4003.501538	4895.067983
ENSG00000138642	HERC6	chr4:88378739-88443111	+	protein_coding	-0.803864268	0.277921146	-2.892418519	0.003822883	0.015806244	Yes	OK	88.14214186	113.1009198	63.18336387	118.6804526	122.4388076	98.18349935	68.12529943	34.84059883	86.58419337
ENSG00000214021	TTLL3	chr3:9808086-9855138	+	protein_coding	-0.803287702	0.138807215	-5.787074553	7.16228E-09	8.68123E-08	Yes	OK	409.3019645	521.9278994	296.6760296	530.3860936	533.7691807	501.6284239	303.802011	248.6351825	337.5908954
ENSG00000082512	TRAF5	chr1:211326615-211374946	+	protein_coding	-0.802861227	0.098429124	-8.156744603	3.44175E-16	8.0129E-15	Yes	OK	865.1160919	1100.763193	629.4689912	1118.537009	1078.741912	1105.010656	660.0789147	598.6248344	629.7032245
ENSG00000106546	AHR	chr7:16916359-17346152	+	protein_coding	-0.801691031	0.116281504	-6.894398544	5.40932E-12	9.16022E-11	Yes	OK	1193.767872	1519.425365	868.11038	1510.28753	1463.663915	1584.324649	959.277865	728.4852482	916.5680268
ENSG00000135931	ARMC9	chr2:231198546-231374837	+	protein_coding	-0.799628753	0.080700545	-9.908591748	3.81992E-23	1.30178E-21	Yes	OK	1840.778353	2337.730659	1343.826047	2294.838841	2396.759665	2321.593471	1303.586811	1363.534345	1364.356986
ENSG00000092820	EZR	chr6:158765741-158819412	-	protein_coding	-0.799367199	0.06332832	-12.62258645	1.58528E-36	9.09187E-35	Yes	OK	5438.75171	6908.141412	3969.362008	6908.67272	6827.764093	6987.987422	3941.140633	3971.828266	3995.117124
ENSG00000188177	ZC3H6	chr2:112275594-112340063	+	protein_coding	-0.798870675	0.192856617	-4.14230369	3.43835E-05	0.000239607	Yes	OK	271.3195649	346.1030537	196.536076	371.7954003	319.3012041	347.2125568	192.4079403	140.9460589	256.2542289
ENSG00000157693	TMEM268	chr9:114611206-114646422	+	protein_coding	-0.79646003	0.126650023	-6.288668675	3.202E-10	4.51581E-09	Yes	OK	417.8592618	530.81672	304.9018036	516.7325902	540.1712099	535.5463601	337.8646607	288.2267721	288.6139779
ENSG00000059728	MXD1	chr2:69897688-69942945	+	protein_coding	-0.796319902	0.154979227	-5.138236368	2.77329E-07	2.75322E-06	Yes	OK	231.3240523	292.4700118	170.1780928	289.8743799	292.0925802	295.4430753	161.107127	196.3742843	153.0528671
ENSG00000172661	WASHC2C	chr10:45727200-45792961	+	protein_coding	-0.795352626	0.141008036	-5.640477313	1.69579E-08	1.97403E-07	Yes	OK	500.5008101	636.4798316	364.5217886	686.876248	628.9993644	593.5638824	428.0846518	310.3980623	355.0826516
ENSG00000108423	TUBD1	chr17:59859482-59892945	-	protein_coding	-0.795178657	0.229594032	-3.463411699	0.000533372	0.002839359	Yes	OK	107.4626499	137.1707478	77.75455203	144.9371899	149.6474315	116.9276219	96.66427622	57.01188899	79.58749087
ENSG00000121310	ECHDC2	chr1:52895910-52927212	-	protein_coding	-0.794494637	0.133241841	-5.962801411	2.4795E-09	3.20098E-08	Yes	OK	318.7741049	404.7423727	232.8058371	410.6553715	400.1268221	403.4449246	220.9469171	226.4638924	251.006702
ENSG00000111058	ACSS3	chr12:80936414-81261205	+	protein_coding	-0.794320682	0.124239111	-6.393483312	1.62149E-10	2.35983E-09	Yes	OK	379.9645843	482.0049671	277.9242014	476.8223495	504.1597959	465.032756	284.4691557	275.5574634	273.7459851
ENSG00000159216	RUNX1	chr21:34787801-36004667	-	protein_coding	-0.794229786	0.066490332	-11.9450416	6.89159E-33	3.5011E-31	Yes	OK	4982.786832	6319.720141	3645.853522	6360.432045	6197.164221	6401.564157	3645.624132	3735.862392	3556.074043
ENSG00000198431	TXNRD1	chr12:104215779-104350305	+	protein_coding	-0.793536766	0.080077865	-9.909564502	3.78291E-23	1.29184E-21	Yes	OK	15046.44391	19086.30169	11006.58613	19101.25126	18970.81289	19186.84093	12019.51229	9894.730066	11105.51603
ENSG00000241399	CD302	chr2:159768630-159798255	-	protein_coding	-0.793385022	0.247030281	-3.211691367	0.001319561	0.006332352	Yes	OK	113.59554	144.5138719	82.67720816	151.2388069	144.8459096	137.4568991	117.8383558	68.09753407	62.09573464
ENSG00000173559	NABP1	chr2:191678068-191696659	+	protein_coding	-0.790879032	0.104361931	-7.578233018	3.50293E-14	7.20422E-13	Yes	OK	2820.27997	3577.164365	2063.395575	3610.826515	3646.755857	3473.910722	2294.165489	1751.531923	2144.489315
ENSG00000113578	FGF1	chr5:142592178-142698070	-	protein_coding	-0.789432232	0.164605822	-4.795894952	1.6195E-06	1.4301E-05	Yes	OK	443.9387821	564.2245178	323.6530464	578.6984903	525.7666443	588.2084188	324.0554784	245.4678554	401.4358056
ENSG00000176531	PHLDB3	chr19:43474954-43504935	-	protein_coding	-0.788991033	0.199633165	-3.952204195	7.74346E-05	0.00049575	Yes	OK	113.0518338	143.6624001	82.44126752	142.836651	132.8421049	155.3084444	81.93448175	74.4321884	90.95713243
ENSG00000148154	UGCG	chr9:111896766-111935369	+	protein_coding	-0.78807023	0.099075847	-7.954211365	1.80276E-15	4.02079E-14	Yes	OK	2050.226289	2598.99494	1501.457639	2579.461873	2642.437533	2575.085415	1650.657593	1314.440774	1539.274549
ENSG00000166839	ANKDD1A	chr15:64911902-64958700	+	protein_coding	-0.787234845	0.30183951	-2.608123921	0.009103999	0.032956196	Yes	OK	88.00234848	112.2598946	63.74480232	101.8761408	122.4388076	112.4647356	98.50550053	33.25693524	59.4719712
ENSG00000117280	RAB29	chr1:205767986-205775460	-	protein_coding	-0.784976494	0.098305354	-7.985083853	1.40427E-15	3.1448E-14	Yes	OK	932.1260135	1180.039833	684.2121939	1204.659108	1109.951805	1225.508587	678.4911578	676.2243499	697.9210738
ENSG00000109083	IFT20	chr17:28328325-28335489	-	protein_coding	-0.782529742	0.149981042	-5.21752437	1.8133E-07	1.84926E-06	Yes	OK	348.5302969	442.0471068	255.013487	445.3142647	480.9524402	399.8746155	281.7073193	212.2109201	271.1222217
ENSG00000206560	ANKRD28	chr3:15667236-15859771	-	protein_coding	-0.78162136	0.111205538	-7.028618997	2.08588E-12	3.73191E-11	Yes	OK	2556.988574	3235.851586	1878.125563	3264.237582	3220.220664	3223.09651	2122.931628	1556.741302	1954.703759
ENSG00000137831	UACA	chr15:70654554-70763593	-	protein_coding	-0.781025083	0.120851258	-6.462697181	1.02853E-10	1.52108E-09	Yes	OK	3321.828745	4202.297542	2441.359949	4315.557344	4145.313877	4146.021404	2862.183188	1960.575516	2501.321142
ENSG00000221955	SLC12A8	chr3:125082636-125212864	-	protein_coding	-0.78082478	0.165955961	-4.70501195	2.53851E-06	2.15714E-05	Yes	OK	203.4342977	256.6202278	150.2483676	227.9084798	261.6829417	280.2692618	134.4093746	161.5336855	154.8020427
ENSG00000109220	CHIC2	chr4:54009789-54064690	-	protein_coding	-0.779386702	0.122667619	-6.353646613	2.1027E-10	3.01747E-09	Yes	OK	504.5133598	638.9252933	370.1014263	621.7595394	658.6087492	636.4075912	390.3395535	324.6510345	395.3136909
ENSG00000162639	HENMT1	chr1:108648290-108661526	-	protein_coding	-0.778831936	0.169287809	-4.600638065	4.21199E-06	3.463E-05	Yes	OK	188.0651514	238.5669616	137.5633413	247.8636002	246.4781224	221.3591622	151.9010055	115.6074416	145.1815768
ENSG00000176472	ZNF575	chr19:43525497-43536130	+	protein_coding	-0.77822428	0.211759531	-3.675037798	0.000237814	0.001371556	Yes	OK	167.4321579	210.0922038	124.772112	210.0538985	233.6740641	186.5486488	105.8703978	167.8683398	100.5775984
ENSG00000163520	FBLN2	chr3:13549131-13638422	+	protein_coding	-0.774550191	0.127240524	-6.087291746	1.14837E-09	1.53551E-08	Yes	OK	18010.0795	22728.67031	13291.48868	22248.90892	22485.5269	23451.57511	10700.27507	16706.06714	12468.12385
ENSG00000256043	CTSO	chr4:155924118-155953917	-	protein_coding	-0.774298214	0.111332963	-6.954797539	3.53069E-12	6.13029E-11	Yes	OK	690.9495074	872.7501484	509.1488665	918.9858058	868.275204	830.9894354	552.3672927	486.18472	488.8945868
ENSG00000184785	SMIM10	chrX:134990938-134992473	+	protein_coding	-0.772516207	0.142349588	-5.426894584	5.7343E-08	6.25491E-07	Yes	OK	291.7723303	367.0546475	216.4900132	364.4435138	376.1192128	360.6012158	216.3438563	240.7168646	192.4093186
ENSG00000148842	CNNM2	chr10:102918293-103090221	+	protein_coding	-0.77232648	0.170632644	-4.526252776	6.00388E-06	4.82774E-05	Yes	OK	316.9276886	398.0113738	235.8440035	384.3986342	424.9346851	384.700802	191.4873281	300.8960808	215.1486017
ENSG00000105976	MET	chr7:116672390-116798386	+	protein_coding	-0.770441997	0.088301842	-8.725095445	2.65956E-18	7.07212E-17	Yes	OK	5708.571514	7200.6038	4216.539227	7370.791297	7143.064029	7087.956075	4591.092814	3723.193084	4335.331783
ENSG00000019485	PRDM11	chr11:45095806-45235110	+	protein_coding	-0.769915365	0.203766401	-3.778421578	0.000157826	0.000945689	Yes	OK	141.1481774	176.8555681	105.4407867	171.1939272	180.8573236	178.5154534	80.09325744	133.027741	103.2013618
ENSG00000137198	GMPR	chr6:16238580-16295549	+	protein_coding	-0.768171158	0.178414872	-4.305533209	1.66584E-05	0.00012329	Yes	OK	147.5315912	185.7813407	109.2818417	171.1939272	180.8573236	205.2927714	106.7910099	110.8564508	110.1980643
ENSG00000177425	PAWR	chr12:79574979-79691097	-	protein_coding	-0.767522645	0.122191975	-6.281285215	3.35785E-10	4.71589E-09	Yes	OK	2597.825684	3275.509674	1920.141695	3276.840816	3249.830049	3299.858155	2252.737942	1542.48833	1965.198813
ENSG00000136193	SCRN1	chr7:29920103-29990289	-	protein_coding	-0.765827461	0.073367201	-10.43828102	1.65785E-25	6.30215E-24	Yes	OK	6081.634492	7661.097069	4502.171915	7576.644117	7838.484445	7568.162645	4760.485451	4207.79414	4538.236155
ENSG00000075826	SEC31B	chr10:100486642-100519864	-	protein_coding	-0.765797267	0.268183797	-2.855494159	0.004296991	0.01747245	Yes	OK	67.99576692	85.14011732	50.85141651	90.32317634	77.62460349	87.47257214	34.98326187	63.34654332	54.22444433
ENSG00000100234	TIMP3	chr22:32801701-32863043	+	protein_coding	-0.764584091	0.052033286	-14.69413414	7.02912E-49	5.72769E-47	Yes	OK	63627.57274	80101.72121	47153.42427	79751.16363	80479.10824	80074.89176	45914.61059	48640.64329	46905.01894
ENSG00000166046	TCP11L2	chr12:106301929-106347015	+	protein_coding	-0.764191113	0.222633266	-3.432510904	0.00059802	0.003139843	Yes	OK	251.5740521	318.0197181	185.1283861	318.2316562	314.4996822	321.327816	259.6126276	115.6074416	180.1650892
ENSG00000105655	ISYNA1	chr19:18434388-18438301	-	protein_coding	-0.763981119	0.224386878	-3.404749533	0.000662248	0.00343434	Yes	OK	205.0507708	256.6593109	153.4422308	260.4668341	225.6715277	283.8395708	109.5528464	216.9619109	133.8119352
ENSG00000100330	MTMR3	chr22:29883155-30030866	+	protein_coding	-0.763856254	0.196533091	-3.886654657	0.000101635	0.000634882	Yes	OK	115.4873012	145.9925156	84.98208685	152.2890764	146.4464169	139.2420536	91.14060329	72.84852482	90.95713243
ENSG00000137876	RSL24D1	chr15:55180806-55197067	-	protein_coding	-0.763844216	0.188837357	-4.044984669	5.23265E-05	0.00034941	Yes	OK	1124.277032	1417.200827	831.3532375	1417.863815	1425.25174	1408.486927	1018.197043	514.6906645	961.1720052
ENSG00000185532	PRKG1	chr10:50990891-52298350	+	protein_coding	-0.76249155	0.135572925	-5.624217004	1.86351E-08	2.1601E-07	Yes	OK	1141.906439	1439.055331	844.7575477	1478.779445	1446.858589	1391.527959	993.3405147	661.9713777	878.9607509
ENSG00000132640	BTBD3	chr20:11890723-11926609	+	protein_coding	-0.762289461	0.106273753	-7.172885487	7.34333E-13	1.36578E-11	Yes	OK	848.4624815	1069.133022	627.7919412	1041.867336	1081.942927	1083.588802	658.2376904	562.200572	662.9375614
ENSG00000186468	RPS23	chr5:82273358-82278577	-	protein_coding	-0.761497632	0.082513418	-9.228773291	2.73749E-20	8.19256E-19	Yes	OK	2987.724018	3759.563942	2215.884095	3786.22152	3588.337341	3904.132965	2365.973237	2128.443855	2153.235193
ENSG00000164692	COL1A2	chr7:94394561-94431232	+	protein_coding	-0.758500128	0.061986776	-12.23648292	1.98447E-34	1.04359E-32	Yes	OK	528845.4253	664745.5641	392945.2865	656985.5782	665999.0916	671252.0226	362721.1889	427326.2792	388788.3913

ENSG00000205413	SAMD9	chr7:93099513-93118023	-	protein_coding	-0.758339345	0.112212846	-6.758043968	1.39867E-11	2.27716E-10	Yes	OK	1167.700623	1469.53595	865.8652953	1520.790225	1498.074822	1389.742804	974.9282716	758.5748562	864.0927581
ENSG00000111275	ALDH2	chr12:111766887-111817529	+	protein_coding	-0.75806544	0.115968153	-6.536841548	6.28316E-11	9.54947E-10	Yes	OK	665.6959807	838.3069012	493.0850602	874.8744871	832.26379	807.7824264	532.1138253	435.5074853	511.6338699
ENSG00000115828	QPCT	chr2:37344574-37373322	+	protein_coding	-0.757842213	0.11324987	-6.691771123	2.20486E-11	3.52007E-10	Yes	OK	650.6614378	818.4560125	482.8668631	799.2550836	855.4711457	800.6418083	529.3519888	451.3441211	467.9044793
ENSG00000152217	SETBP1	chr18:44680173-45068510	+	protein_coding	-0.757653926	0.120741384	-6.275014415	3.49602E-10	4.8974E-09	Yes	OK	500.0187204	629.4044648	370.632976	613.3573835	632.200379	642.6556321	364.5624132	338.9040068	408.4325081
ENSG00000168016	TRANK1	chr3:36826820-36945098	-	protein_coding	-0.753742171	0.110005381	-6.851866352	7.28926E-12	1.21685E-10	Yes	OK	714.7919453	896.0743049	533.5095857	896.9301464	890.682306	900.6104622	484.2419932	582.7881985	533.4985652
ENSG00000186073	C15orf41	chr15:36579611-36810248	+	protein_coding	-0.753264801	0.111210301	-6.773336579	1.25846E-11	2.05498E-10	Yes	OK	600.4715457	754.6504775	446.292614	781.4005023	727.4305626	755.1203677	475.9564839	421.2545131	441.666845
ENSG00000117533	VAMP4	chr1:171700160-171742247	-	protein_coding	-0.752770829	0.22135991	-3.400664689	0.000672222	0.00348168	Yes	OK	564.6351027	710.1986165	419.0715889	690.0270564	712.2257434	728.3430497	598.3979004	248.6351825	410.1816837
ENSG00000123119	NECAB1	chr8:90791550-90959408	+	protein_coding	-0.752121166	0.216874771	-3.467997517	0.000524352	0.00279496	Yes	OK	117.4823895	147.754646	87.21013299	150.1885374	152.0481924	141.0272082	112.3146828	77.59951556	71.71620057
ENSG00000187189	TSPYL4	chr6:116249961-116254140	-	protein_coding	-0.751218009	0.089681552	-8.376505452	5.45224E-17	1.33746E-15	Yes	OK	1534.883886	1926.278057	1143.489716	2045.924971	1870.99302	1861.916178	1188.510291	1125.984807	1115.974048
ENSG00000013563	DNASE1L1	chrX:154401238-154412112	-	protein_coding	-0.749387573	0.161137872	-4.650598672	3.30973E-06	2.76679E-05	Yes	OK	293.8127303	366.8992019	220.7262587	380.1975562	369.7171836	350.7828658	186.8842674	272.3901363	202.9043723
ENSG00000083123	BCKDHB	chr6:80106647-80346270	+	protein_coding	-0.749002045	0.144075407	-5.198680761	2.00708E-07	2.03427E-06	Yes	OK	503.6111925	633.0559772	374.1664079	587.1006462	634.6011399	677.4661455	427.1640397	300.8960808	394.4391031
ENSG00000170624	SGCD	chr5:155870344-156767788	+	protein_coding	-0.748976811	0.099906291	-7.496793289	6.5398E-14	1.3208E-12	Yes	OK	6950.42513	8718.201353	5182.648906	8947.245805	8740.370302	8466.987953	5764.873311	4377.246143	5405.827265
ENSG00000136048	DRAM1	chr12:101877351-102012130	+	protein_coding	-0.748246556	0.106474389	-7.02747924	2.10298E-12	3.75842E-11	Yes	OK	1929.577594	2421.407187	1437.748	2558.456483	2420.767274	2284.997803	1579.770457	1243.175913	1490.297631
ENSG00000163701	IL17RE	chr3:9902612-9916402	+	protein_coding	-0.746732143	0.188728432	-3.956648908	7.60085E-05	0.000487436	Yes	OK	148.0399537	184.6090964	111.470811	177.4955442	180.8573236	195.4744214	92.06121545	133.027741	109.3234765
ENSG00000154734	ADAMTS1	chr21:26835747-26845409	-	protein_coding	-0.745824896	0.065753732	-11.34270069	8.06175E-30	3.57672E-28	Yes	OK	7260.187545	9094.222527	5426.152563	9073.278144	9106.886472	9102.502967	5200.538061	5661.597309	5416.322319
ENSG00000105889	STEAP1B	chr7:22419444-22632925	-	protein_coding	-0.745780035	0.24459162	-3.049082525	0.002295414	0.010244717	Yes	OK	70.65404322	88.09998974	53.20809669	94.52425431	91.22891544	78.54679948	50.6336685	61.76287974	47.22774184
ENSG00000114796	KLHL24	chr3:183635568-183684477	+	protein_coding	-0.745543288	0.165283182	-4.510702651	6.46132E-06	5.1678E-05	Yes	OK	702.5959899	882.2845537	522.9074261	930.5387702	797.8528833	918.4620075	568.9383115	375.3282692	624.4556976
ENSG00000106823	ECM2	chr9:92493554-92536655	-	protein_coding	-0.745086532	0.155970056	-4.77711267	1.7783E-06	1.55862E-05	Yes	OK	341.2704015	429.1544288	253.3863741	448.4650732	425.7349387	413.2632745	290.9134408	196.3742843	272.8713973
ENSG00000219438	FAM19A5	chr22:48489460-48850912	+	protein_coding	-0.745077803	0.117329077	-6.350325249	2.1486E-10	3.08066E-09	Yes	OK	1687.791731	2112.045806	1263.537657	2112.091949	2093.463533	2130.581936	1056.862753	1466.472478	1267.277739
ENSG00000185880	TRIM69	chr15:44728988-44767829	+	protein_coding	-0.744145964	0.147775773	-5.035642504	4.76249E-07	4.58156E-06	Yes	OK	377.1479411	473.2791129	281.0167693	549.2909445	438.538997	432.0073971	276.1836463	258.137164	308.7294976
ENSG00000132906	CASP9	chr1:15490832-15526534	-	protein_coding	-0.742832843	0.173690283	-4.276766853	1.89627E-05	0.000138477	Yes	OK	197.8167008	248.5383991	147.0950024	221.6068629	262.4831953	261.5251392	156.5040663	120.3584323	164.4225086
ENSG00000187720	THSD4	chr15:71096952-71783383	+	protein_coding	-0.742365277	0.108097344	-6.867562614	6.53081E-12	1.09579E-10	Yes	OK	956.7887076	1195.677304	717.9001109	1197.307221	1227.58909	1162.135601	665.6025877	807.6684273	680.4293176
ENSG00000018869	ZNF582	chr19:56375846-56393545	-	protein_coding	-0.74145036	0.288598651	-2.569140078	0.010195124	0.036220966	Yes	OK	68.38022126	86.38312264	50.37731987	74.56913395	94.42993002	90.15030394	56.15734142	28.50594449	66.4686737
ENSG00000174080	CTSF	chr11:66563463-66568841	-	protein_coding	-0.741153594	0.118519907	-6.253410193	4.01586E-10	5.5923E-09	Yes	OK	1035.257937	1293.290034	777.225841	1319.138482	1323.619528	1237.112092	690.4591158	907.439233	733.7791741
ENSG00000135842	FAM129A	chr1:184790724-184974550	-	protein_coding	-0.741077162	0.076987804	-9.625903422	6.21583E-22	1.9944E-20	Yes	OK	3638.316909	4554.797128	2721.83669	4648.492773	4549.441968	4466.456643	2722.250141	2562.367677	2880.892252
ENSG00000128709	HOXD9	chr2:176122720-176124937	+	protein_coding	-0.741057945	0.180540319	-4.104667307	4.04897E-05	0.000277345	Yes	OK	216.6516601	269.6458853	163.6574349	241.5619832	288.8915656	278.4841072	136.2505989	204.2926022	150.4291036
ENSG00000180787	ZFP3	chr17:5078248-5096374	+	protein_coding	-0.74073873	0.186252686	-3.977063341	6.97716E-05	0.000451254	Yes	OK	151.9383831	190.0839542	113.792812	174.3447357	196.8623965	199.0447305	132.5681502	110.8564508	97.95383492
ENSG00000232593	KANTR	chrX:53494165-53170914	+	protein_coding	-0.740115223	0.282972647	-2.615500935	0.008909668	0.032359473	Yes	OK	81.39233243	102.7529941	60.03167081	105.0269492	95.23018367	108.0018493	85.61693037	33.25693524	61.2214683
ENSG00000130150	MOSPD2	chrX:14873441-14922327	+	protein_coding	-0.739723996	0.138330043	-5.34752958	8.91628E-08	9.46241E-07	Yes	OK	1251.980506	1568.009245	935.9517665	1709.838733	1550.891563	1443.29744	1128.670501	761.7421834	917.4426146
ENSG00000162600	OMA1	chr1:58415384-58546802	-	protein_coding	-0.739216851	0.165970674	-4.453900393	8.43242E-06	6.58433E-05	Yes	OK	189.4546693	237.5064724	141.4028662	244.7127917	254.4806589	213.3259668	146.3773326	128.2767502	149.5545158
ENSG00000119684	MLH3	chr14:75013764-75051532	-	protein_coding	-0.738804119	0.161657888	-4.570170542	4.87327E-06	3.96119E-05	Yes	OK	483.4269536	606.4275907	360.4263164	651.1670852	605.7920087	562.3236781	425.3228154	267.6391455	388.3169884
ENSG00000111276	CDKN1B	chr12:12715058-12722371	+	protein_coding	-0.73872251	0.096498729	-7.655256399	1.92927E-14	4.07648E-13	Yes	OK	843.3533578	1054.926024	631.7806921	1043.967875	1059.535825	1061.27437	652.7140175	620.7961245	621.8319342
ENSG00000118707	TGIF2	chr20:36573488-36593950	+	protein_coding	-0.738187703	0.16634117	-4.43779314	9.08859E-06	7.0333E-05	Yes	OK	262.4979304	326.681774	198.3140869	295.1257273	337.7070379	347.2125568	178.598758	242.3005282	174.0429745
ENSG00000005249	PRKAR2B	chr7:107044649-107161811	+	protein_coding	-0.736778212	0.195830718	-3.762321967	0.000168343	0.001001782	Yes	OK	213.2466875	267.9830412	158.5103339	283.5727629	255.2809125	265.0954482	160.1865149	112.4401144	202.9043723
ENSG00000196110	ZNF699	chr19:9294275-9309838	-	protein_coding	-0.736045385	0.191552558	-3.84252443	0.000121775	0.000747361	Yes	OK	130.0626422	163.079529	97.04575538	173.2944662	170.4540262	145.4900945	106.7910099	85.51783348	98.82842273
ENSG00000138764	CCNG2	chr4:77157151-77433388	+	protein_coding	-0.734289412	0.153253014	-4.791353801	1.6566E-06	1.4605E-05	Yes	OK	1216.836982	1522.026952	911.6470121	1549.147501	1485.270764	1531.66259	1142.479684	679.3916771	913.0696755
ENSG00000179454	KLHL28	chr14:44924319-45042322	-	protein_coding	-0.733959433	0.205709881	-3.567934745	0.000359806	0.001994077	Yes	OK	432.5819914	541.9888201	323.1751627	550.341214	540.9714635	534.6537828	423.4815911	197.9579479	348.0859491
ENSG00000128965	CHAC1	chr15:40952962-40956519	+	protein_coding	-0.731956932	0.153570705	-4.766253654	1.87683E-06	1.6354E-05	Yes	OK	1101.581311	1372.979197	830.1834248	1398.958964	1250.796446	1469.182181	683.0942186	1065.805591	741.6504644
ENSG00000132326	PER2	chr2:238244038-238290102	-	protein_coding	-0.731623788	0.127029586	-5.759475503	8.43757E-09	1.01374E-07	Yes	OK	382.1169667	476.2889671	287.9449662	484.1742359	492.1559912	452.5366743	269.7393613	307.2307351	286.8648023
ENSG00000082996	RNF13	chr3:149812708-149962139	+	protein_coding	-0.731134792	0.183808085	-3.977707467	6.95829E-05	0.00045021	Yes	OK	708.4823163	886.1718286	530.792804	937.8906566	861.0729212	859.5519079	677.5705457	351.5733154	563.2345508
ENSG00000158008	EXTL1	chr1:26019884-26036464	+	protein_coding	-0.731076764	0.235351164	-3.106323128	0.001894296	0.00869482	Yes	OK	101.9869242	126.1649868	77.80886155	130.233417	130.441344	117.8201992	62.6016265	106.1054601	64.71949807
ENSG00000182621	PLCB1	chr20:8077251-8968360	+	protein_coding	-0.730921089	0.131091116	-5.575672173	2.46576E-08	2.81459E-07	Yes	OK	478.6756002	599.0898504	358.26135	615.4579225	572.1813556	609.6302732	385.7364927	302.4797443	386.5678128
ENSG00000156172	C8orf37	chr8:95244919-95269201	-	protein_coding	-0.730446811	0.299325911	-2.440305984	0.014674826	0.048546241	Yes	OK	68.25266537	85.87635259	50.62897814	88.22263735	94.42993002	74.97649041	74.56958451	30.08960808	47.22774184
ENSG00000119917	IFIT3	chr10:89327894-89340971	+	protein_coding	-0.730369344	0.1237483	-5.902055598	3.59E-09	4.52461E-08	Yes	OK	681.4620665	851.2161963	511.7079367	936.8403871	817.0589708	799.749231	573.5413722	481.4337292	480.1487087
ENSG00000198205	ZXDA	chrX:57906708-57910820	-	protein_coding	-0.730086034	0.263036348	-2.77560892	0.005509844	0.021593341	Yes	OK	76.79779314	96.42037566	57.17521062	94.52425431	76.0240962	118.7127765	59.83979004	44.34258032	67.34326151
ENSG00000141510	TP53	chr17:7661779-7687550	-	protein_coding	-0.728225787	0.091637596	-7.946801489	1.91389E-15	4.26287E-14	Yes	OK	1540.494035	1919.804927	1161.183143	1995.512035	1842.183889	1921.718855	1123.146828	1247.926903	1112.475697
ENSG00000156103	MMP16	chr8:88032009-88328025	-	protein_coding	-0.727654213	0.137953084	-5.274649827	1.3301E-07	1.38654E-06	Yes	OK	963.9191199	1204.096161	723.7418243	1270.826086	1173.972096	1167.491065	855.2486915	571.7025534	744.2742278
ENSG00000147853	AK3	chr9:4709559-4742043	-	protein_coding	-0.727569974	0.146912189	-4.95241395	7.32985E-07	6.84729E-06	Yes	OK	1663.912427	2077.126339	1250.698514	2021.768773	2087.061504	2122.54874	1478.50312	915.3575509	1358.234872
ENSG00000277443	MARCKS	chr6:113857362-113863471	+	protein_coding	-0.72624745	0.059614393	-12.18241796	3.85676E-34	2.00893E-32	Yes	OK	15931.88523	19859.38258	12004.38787	20500.21022	19357.3354	19720.60213	12179.6988	12058.01452	11775.4503
ENSG00000188785	ZNF548	chr19:57389850-57402992	+	protein_coding	-0.725922774	0.187270899	-3.876324508	0.000106046	0.000660116	Yes	OK	273.8760824	342.9613458	204.790819	361.2927054	340.9080525	326.6832796	250.406506	140.9460589	223.019892
ENSG00000177565	TBL1XR1	chr3:177019340-177228000	-	protein_coding	-0.725198894	0.173599324	-4.177429264	2.94822E-05	0.000207917	Yes	OK	2458.033504	3065.270688	1850.796321	3131.903626	3096.981603	2966.926835	2400.956499	1227.339277	1924.093186
ENSG00000149311	ATM	chr11:108222484-108369102	+	protein_coding	-0.72479048	0.108761017	-6.664064955	2.66356E-11	4.19542E-10	Yes	OK	2150.349715	2681.993175	1618.706255	2731.750949	2608.82688	2705.401696	1809.002884	1363.534345	1683.581538
ENSG00000274180	NATD1	chr17:21238870-21253410	-	protein_coding	-0.723565198	0.144886342	-4.994019383	5.91355E-07	5.60374E-06	Yes	OK	643.4436698	799.982441	486.9048987	837.0647854	704.2232069	858.6593306	413.3548574	581.2045349	466.1553037
ENSG00000130766	SESN2	chr1:28259527-28282491	+	protein_coding	-0.723545631	0.122560291	-5.903589357	3.55677E-09	4.48616E-08	Yes	OK	1560.371384	1941.15861	1179.584158	1870.529966	1936.613819	2016.332046	1008.070309	1409.460589	1121.221575
ENSG00000160050	CCDC28B	chr1:32200386-32205387	+	protein_coding	-0.723495389	0.296694341	-2.43852103	0.014747501	0.048725786	Yes	OK	76.36224851	94.34162621	58.38287082	89.27290685	76.82434985	116.9276219	36.82448618	82.35050631	55.97361996
ENSG00000113140	SPARC	chr5:151661096-151687165	-	protein_coding	-0.72347104	0.044327893	-16.32089844	7.00963E-60	7.07844E-58	Yes	OK	153580.5219	191300.5583	115860.4855	191804.4158	192436.1936	189661.0657	115416.2252	116915.5477	115249.6835
ENSG00000151881	TMEM267	chr5:43444252-43483893	-	protein_coding	-0.723423292	0.17982982	-4.02282165	5.7505E-05	0.000379524	Yes	OK	449.3047657	561.1504209	337.4591105	581.8492987	516.9638542	584.6381097	441.8938341	248.6351825	321.8483147
ENSG00000186310	NAP1L3	chrX:93670930-93673568	-	protein_coding	-0.72306709	0.143119432	-5.05219369	4.36764E-07	4.21898E-06	Yes	OK	299.5664162	374.3194871	224.8133453	373.8959393	369.7171836	379.3453384	245.8034452	191.6232935	237.013297
ENSG00000168209	DDIT4	chr10:72273920-72276036	+	protein_coding	-0.722441169	0.218206734	-3.310810603	0.000930261	0.004656966	Yes	OK	593.5189053	737.0885904	449.9492202	706.8313683	723.4292944	781.0051084	312.0875204	673.0570228	364.7031175
ENSG00000198270	TMEM116	chr12:111931282-112013185	-	protein_coding	-0.722285692	0.216762938	-3.332145705	0.000861791	0.004357937	Yes	OK	104.0047546	128.8631579	79.14635186	130.233417	114.4362711	141.9197854	67.20468728	95.01981498	75.21455182
ENSG00000102452	NALCN	chr13:101053776-101416492	-	protein_coding	-0.722009448	0.210543731	-3.4292612	0.000605227	0.003173633	Yes	OK	122.7364641	153.5278147	91.94511345	173.2944662	148.0469242	139.2420536	113.235295	76.01585198	86.58419337
ENSG00000111554	MDM1	chr12:68272443-68332381	-	protein_coding	-0.72170616	0.169146465	-4.266752835	1.98339E-05	0.000144135	Yes	OK	291.4229681	364.2232699	218.6226664	356.0413579	400.1268221	336.5016296	241.2003845	166.2846762	248.3829386
ENSG00000138190	EXOC6	chr10:92834713-93059493	+	protein_coding	-0.721571728	0.203840778	-3.539879186	0.00040031	0.002189799	Yes	OK	114.9996936	143.654654	86.34473329	153.3393459	154.4489533	123.1756628	92.06121545	76.01585198	90.95713243
ENSG00000185418	TARSL2	chr15:101653598-101724604	-	protein_coding	-0.721409963	0.149811891	-4.815438592	1.46877E-06	1.3054E-05	Yes	OK	277.0247628	346.0687979	207.9807277	351.8402799	332.905516	353.4605976	234.7560994	175.7866577	213.3994261
ENSG00000151338	MIPOL1	chr14:37197913-37552361	+	protein_coding	-0.720235143	0.231168789	-3.115624501	0.001835558	0.008458368	Yes	OK	119.0247697	149.2136313	88.83590819	169.0933883	123.2390612	155.3084444	106.7910099	61.76287974	97.95383492
ENSG00000067141	NEO1	chr15:73051710-73305206	+	protein_coding	-0.719954292	0.073398051	-9.808902058	1.03084E-22	3.44871E-21	Yes	OK	2655.403258	3304.788517	2006.018	3343.007794	3348.261248	3223.09651	2030.870413	1992.248787	1994.934799
ENSG00000116679	IVNS1ABP	chr1:185296388-185317329	-	protein_coding	-0.71808034	0.104069061	-6.900036726	5.19891E-12	8.81298E-11	Yes	OK	2999.763415	3733.783914	2265.742916	3880.745774	3785.999991	3534.605976	2528.000976	1946.322543	2322.905228
ENSG00000164663	USP49	chr6:41789896-41895361	-	protein_coding	-0.717871531	0.162708425	-4.412012038	1.02414E-05	7.84066E-05	Yes	OK	187.0348814	232.310047	141.7597158	240.5117137	244.8776151	211.5408122	140.8536596	148.8643768	135.5611108
ENSG00000164967	RPP25L	chr9:34610486-34612104	-	protein_coding	-0.717849109	0.174403734	-4.116019138	3.85472E-05	0.000265921	Yes	OK	146.4319368	182.182842	110.6810317	173.2944662	192.0608746	181.1931852	114.1559072	107.6891236	110.1980643
ENSG00000106948	AKNA	chr9:114334156-114394405	-	protein_coding	-0.717682509	0.104681464	-6.855870015	7.08798E-12	1.18565E-10	Yes	OK	838.4862944	1042.256179	634.7164093	1061.822457	986.7127434	1078.233338	589.1917789	665.1387048	649.8187442
ENSG00000165322	ARHGAP12	chr10:31805404-31928876	-	protein_coding	-0.717265997	0.121338758	-5.911268659	3.39483E-09	4.30832E-08	Yes	OK	1442.155543	1795.92154	1088.389546	1800.16191	1799.770446	1787.832265	1179.30417	878.9332885	1206.93118
ENSG00000170456	DENND5B	chr12:31382223-31591097	-	protein_coding	-0.717114279	0.114091368	-6.285438508	3.2693E-10	4.59544E-09	Yes	OK	891.5391771	1110.448567	672.629787	1110.134853	1133.15916	1088.051688	750.2989059	582.7881985	684.8022566
ENSG00000166821	PEX11A	chr15:89677764-89690783	-	protein_coding	-0.71324176	0.228075623	-3.1272161	0.001764702	0.008170742	Yes	OK	102.8372604	128.4876859	77.18683493	116.5799136	129.6410904	139.2420536	97.58488837	57.01188899	76.96372744
ENSG00000197121	PGAP1	chr2:196833004-196927796	-	protein_coding	-0.71260622	0.144335868	-4.937138825	7.9277E-07	7.35155E-06	Yes	OK	633.9534891	789.213005	478.6939731	899.0306854	732.2320845	736.3762451	541.3199468	392.7485686	502.013404
ENSG00000138380	CARF	chr2:202912214-202987063	+	protein_coding	-0.710683867	0.192951216	-3.683230833	0.000230296	0.001331103	Yes	OK	183.4018332	228.8110572	137.9926091	265.7181816	221.6702595	199.0447305	161.107127	107.6891236	145.1815768
ENSG00000122512	PMS2	chr7:5973239-6009125	-	protein_coding	-0.710566055	0.147050977	-4.832107	1.35096E-06	1.20524E-05	Yes	OK	240.0453837	297.8160408	182.2747266	316.1311172	280.0887755	297.2282298	173.9956972	190.03963	182.7888527
ENSG00000164107	HAND2	chr4:173524969-173530229	-	protein_coding	-0.710459525	0.173984726	-4.083459181	4.43702E-05	0.000301169	Yes	OK	282.28039	348.928592	215.632188	349.7397409	317.7006968	379.3453384	178.598758	272.3901363	195.9076698
ENSG00000003393	ALS2	chr2:201700554-201781189	-	protein_coding	-0.707877409	0.101432928	-6.978773268	2.97769E-12	5.21967E-11	Yes	OK	1238.279166	1538.194321	938.36401	1581.705855	1517.280909	1515.596199	1002.546636	833.0070446	979.5383492
ENSG00000166348	USP54	chr10:73497538-73625953	-	protein_coding	-0.707230434	0.150411132	-4.701981979	2.57648E-06	2.18715E-05	Yes	OK	319.9651163	395.8507403	244.0794923	430.6104918	363.3151545	393.6265746	240.2797723	280.3084542	211.6502505
ENSG00000136738	STAM	chr10:17644125-17715914	+	protein_coding	-0.705892106	0.12478343	-5.656937816	1.54098E-08	1.80402E-07	Yes	OK	1579.482281	1960.757017	1198.207544	2094.237368	1880.596064	1907.437619	1372.632722	973.9531035	1248.036807

ENSG00000156113	KCNMA1	chr10:76869601-77638369	-	protein_coding	-0.70432503	0.078336508	-8.991018993	2.44949E-19	6.8569E-18	Yes	OK	2145.239544	2659.528011	1630.951078	2615.171036	2631.233982	2732.179014	1628.562901	1586.83091	1677.459423
ENSG00000166289	PLEKHF1	chr19:29665056-29675457	+	protein_coding	-0.704186213	0.204092112	-3.450335278	0.000559891	0.002962927	Yes	OK	374.4133967	462.092865	286.7339284	452.6661512	495.3570058	438.255438	244.8828331	405.4178772	209.9010748
ENSG00000178814	OPLAH	chr8:144051266-144063965	-	protein_coding	-0.703688878	0.186611983	-3.770866508	0.000162682	0.000972354	Yes	OK	458.9473592	566.7204141	351.1743042	572.3968733	560.9778046	566.7865644	267.8981369	483.0173928	302.6073829
ENSG00000111911	HINT3	chr6:125956781-125980244	+	protein_coding	-0.703406088	0.158808242	-4.429279493	9.45484E-06	7.28589E-05	Yes	OK	598.8001534	743.6240529	453.9762538	706.8313683	754.6391865	769.401604	561.5734142	342.0713339	458.2840134
ENSG00000168502	MTCL1	chr18:8705661-8832778	+	protein_coding	-0.702811273	0.08263177	-8.50533967	1.81065E-17	4.60638E-16	Yes	OK	3029.72937	3751.226716	2308.232023	3737.909123	3680.36651	3835.404515	2174.485909	2499.021134	2251.189028
ENSG00000177854	TMEM187	chrX:153972327-153983195	+	protein_coding	-0.702629691	0.2606639	-2.695538932	0.007027486	0.026609714	Yes	OK	105.1536183	129.8997264	80.40751012	113.4291052	135.2428659	141.0272082	48.79244419	91.85248781	100.5775984
ENSG00000140853	NLRC5	chr16:56989485-57083531	+	protein_coding	-0.701229847	0.106819145	-6.564645745	5.21567E-11	7.97121E-10	Yes	OK	1150.940245	1423.011736	878.8687547	1412.612467	1445.258082	1411.164659	823.9478782	999.2917208	813.366665
ENSG00000164930	FZD6	chr8:103298433-103332866	+	protein_coding	-0.701073533	0.105735516	-6.630445104	3.34676E-11	5.21664E-10	Yes	OK	2045.012324	2534.88816	1555.136488	2601.517532	2403.961947	2599.185001	1689.323303	1331.861073	1644.225086
ENSG00000128284	APOL3	chr22:36140330-36166177	-	protein_coding	-0.700662836	0.212466048	-3.297763772	0.000974581	0.004861091	Yes	OK	108.9866986	134.5892704	83.38412685	139.6858425	148.0469242	116.0350447	69.96652374	91.85248781	88.33336899
ENSG00000185909	KLHDC8B	chr3:49171611-49176486	+	protein_coding	-0.700634195	0.114793968	-6.103406037	1.03832E-09	1.39516E-08	Yes	OK	508.1924165	629.5444097	386.8404234	601.8044191	628.9993644	657.8294456	381.1334319	375.3282692	404.0595691
ENSG00000182492	BGN	chrX:153494939-153509554	+	protein_coding	-0.700492426	0.126764468	-5.525936711	3.27732E-08	3.66971E-07	Yes	OK	30537.98685	37806.14878	23269.82491	37647.96022	37217.39623	38553.08988	19296.95137	29658.85158	20853.67178
ENSG00000181458	TMEM45A	chr3:100492619-100577444	+	protein_coding	-0.699457021	0.138782516	-5.039950572	4.65652E-07	4.49012E-06	Yes	OK	937.7018624	1162.918459	712.4852662	1257.172582	1105.150283	1126.432511	797.2501258	557.4495812	782.7560916
ENSG00000013392	RWDD2A	chr6:83193379-83198932	+	protein_coding	-0.697548269	0.161152932	-4.328486354	1.50138E-05	0.000112076	Yes	OK	189.6280184	235.3430342	143.9130026	237.3609053	239.2758396	229.3923576	153.7422298	126.6930866	151.3036914
ENSG00000118046	STK11	chr19:1177558-1228435	+	protein_coding	-0.697274513	0.176161276	-3.95815999	7.55294E-05	0.000484873	Yes	OK	1245.065456	1537.882191	952.2487204	1382.154652	1582.901708	1648.590212	727.283602	1298.604138	830.8584212
ENSG00000072422	RHOBTB1	chr10:60869438-61001440	-	protein_coding	-0.696237397	0.092266861	-7.545909657	4.49141E-14	9.18368E-13	Yes	OK	1071.242969	1325.820527	816.6654124	1308.635787	1322.819274	1346.006518	840.518897	775.9951556	833.4821847
ENSG00000117155	SSX2IP	chr1:84643707-84690803	-	protein_coding	-0.695311367	0.189033356	-3.678246968	0.000234843	0.001354892	Yes	OK	1355.305366	1677.70439	1032.906343	1786.508406	1622.114137	1624.490626	1354.220479	655.6367233	1088.861826
ENSG00000155324	GRAMD2B	chr5:126360132-126496494	+	protein_coding	-0.695243281	0.105689293	-6.578180852	4.76239E-11	7.3124E-10	Yes	OK	2304.504356	2851.953207	1757.055505	2910.296763	2839.29993	2806.262927	1913.032057	1483.892777	1874.241681
ENSG00000240344	PPIL3	chr2:200870907-200889303	-	protein_coding	-0.693997092	0.185500674	-3.741210617	0.000183136	0.001083554	Yes	OK	460.9443122	571.569219	350.3194054	577.6482208	557.77679	579.2826461	421.6403667	229.6312195	399.68663
ENSG00000113739	STC2	chr5:173314713-173329503	-	protein_coding	-0.691686553	0.076044731	-9.095785413	9.39053E-20	2.73572E-18	Yes	OK	13988.9084	17276.1705	10701.64629	17103.63868	17554.36394	17170.50888	10080.70309	11753.95111	10270.28467
ENSG00000198756	COLGALT2	chr1:183929854-184037729	-	protein_coding	-0.69142238	0.131074464	-5.275034957	1.32731E-07	1.3845E-06	Yes	OK	359.1379949	444.4740307	273.8019591	475.77208	426.5351924	431.1148199	288.1516043	253.3861733	279.8680998
ENSG00000172331	BPGM	chr7:134646808-134679813	+	protein_coding	-0.689479457	0.119651831	-5.762381155	8.29354E-09	9.97893E-08	Yes	OK	577.1680938	712.1879628	442.1482248	748.842148	690.618895	697.1028454	475.0358717	456.0951119	395.3136909
ENSG00000197128	ZNF772	chr19:57466663-57477570	-	protein_coding	-0.689165027	0.17488307	-3.94071895	8.12378E-05	0.000518284	Yes	OK	273.1475078	338.3820997	207.9129159	341.337585	348.9105889	324.8981251	193.3285524	166.2846762	264.1255192
ENSG00000101752	MIB1	chr18:21704957-21870957	+	protein_coding	-0.688894441	0.123868056	-5.561518158	2.67438E-08	3.02961E-07	Yes	OK	2079.74606	2569.703745	1589.788374	2639.327234	2507.194667	2562.589333	1779.543295	1259.012548	1730.80928
ENSG00000182199	SHMT2	chr12:57229327-57234935	+	protein_coding	-0.68875646	0.098115472	-7.019855748	2.22097E-12	3.9607E-11	Yes	OK	5025.466026	6200.118588	3850.813464	6031.697694	6217.970816	6350.687253	3354.710691	4366.160498	3831.569204
ENSG00000051620	HEBP2	chr6:138403531-138422197	+	protein_coding	-0.688146516	0.085174746	-8.079231787	6.5176E-16	1.48176E-14	Yes	OK	1509.355721	1863.942186	1154.769256	1893.635895	1846.985411	1851.205251	1179.30417	1099.062527	1185.941073
ENSG00000204947	ZNF425	chr7:149102784-149126346	-	protein_coding	-0.68758863	0.224771652	-3.059054035	0.002220371	0.009958394	Yes	OK	104.8085089	130.0548337	79.56218398	114.4793747	124.8395685	150.8455581	89.29937898	60.17921615	89.2079568
ENSG00000110057	UNC93B1	chr11:67991104-68004982	-	protein_coding	-0.687044482	0.189518796	-3.625204981	0.000288732	0.001633173	Yes	OK	323.9272621	397.9361194	249.9184047	356.0413579	420.9334169	416.8335836	185.9636552	329.4020253	234.3895336
ENSG00000136114	THSD1	chr13:52377167-52406494	-	protein_coding	-0.686801683	0.18705456	-3.67166501	0.000240975	0.001388329	Yes	OK	353.622773	434.5004579	272.7450882	474.7218105	413.7311341	415.0484291	235.6767115	367.4099512	215.1486017
ENSG00000003400	CASP10	chr2:201182881-201229406	+	protein_coding	-0.686298021	0.205357644	-3.341964809	0.000831876	0.004222226	Yes	OK	100.6716854	124.1156616	77.2277092	123.9318001	113.6360175	134.7791673	76.41080882	79.18317915	76.08913963
ENSG00000197971	MBP	chr18:76978827-77133683	-	protein_coding	-0.685379939	0.280618019	-2.442394618	0.014590189	0.048311105	Yes	OK	110.8911346	135.7083898	86.07387941	165.9425798	137.6436268	103.5389629	62.6016265	128.2767502	67.34326151
ENSG00000151718	WWC2	chr4:183099293-183320777	+	protein_coding	-0.685146269	0.082219924	-8.333092933	7.87573E-17	1.9092E-15	Yes	OK	3540.040911	4367.439422	2712.642399	4258.842791	4427.00316	4416.472316	2774.725034	2483.184498	2880.017664
ENSG00000196458	ZNF605	chr12:132918308-132956306	-	protein_coding	-0.68486694	0.149118398	-4.592772922	4.37395E-06	3.59078E-05	Yes	OK	584.0009739	721.9122005	446.0897474	721.5351412	765.8427375	678.3587227	508.1779093	342.0713339	488.019999
ENSG00000139517	LNX2	chr13:27545911-27620404	-	protein_coding	-0.68426783	0.147444844	-4.640839333	3.46997E-06	2.89486E-05	Yes	OK	252.0211056	311.5372735	192.5049377	302.4776138	323.3024723	308.8317343	202.534674	169.4520034	205.5281358
ENSG00000187164	SHTN1	chr10:116881482-117126586	-	protein_coding	-0.682562769	0.177446202	-3.846589912	0.000119773	0.000737151	Yes	OK	344.8008142	426.2357818	263.3658467	444.2639952	449.7425481	384.700802	333.2615999	197.9579479	258.8779923
ENSG00000115738	ID2	chr2:8678845-8684453	+	protein_coding	-0.682110062	0.066194875	-10.30457508	6.71879E-25	2.4852E-23	Yes	OK	4714.653181	5809.064334	3620.242028	5707.164421	5953.08686	5766.941721	3606.037809	3590.165343	3664.522931
ENSG00000170915	PAQR8	chr6:52361421-52407777	+	protein_coding	-0.681657944	0.155182749	-4.392614182	1.11996E-05	8.51872E-05	Yes	OK	237.0345862	291.8529438	182.2162285	297.2262663	271.2859854	307.0465798	198.8522254	186.8723028	160.9241574
ENSG00000203995	ZYG11A	chr1:52842511-52894998	+	protein_coding	-0.680798873	0.214977946	-3.166831225	0.001541097	0.007251704	Yes	OK	92.02907588	113.0047767	71.05337506	115.5296442	120.8383003	102.6463857	69.96652374	77.59951556	65.59408589
ENSG00000125730	C3	chr19:6677704-6730562	-	protein_coding	-0.680614755	0.256986071	-2.648449979	0.008086181	0.029909786	Yes	OK	71.94443695	88.80005809	55.08881581	105.0269492	94.42993002	66.94329501	56.15734142	52.26089824	56.84820777
ENSG00000079150	FKBP7	chr2:178463664-178478600	-	protein_coding	-0.680131718	0.163701437	-4.154708283	3.25703E-05	0.000228228	Yes	OK	749.8517368	925.3229136	574.38056	887.477721	868.275204	1020.215816	675.7293214	413.3361952	634.0761636
ENSG00000111581	NUP107	chr12:68686734-68745809	+	protein_coding	-0.67929591	0.158877156	-4.275604671	1.90619E-05	0.000139016	Yes	OK	966.3193866	1191.839794	740.7989787	1222.513689	1213.184525	1139.82117	876.422771	527.3599731	818.6141919
ENSG00000170271	FAXDC2	chr5:154818491-154859252	-	protein_coding	-0.679088348	0.136211117	-4.985557457	6.17834E-07	5.84121E-06	Yes	OK	368.7819214	452.7574689	284.806374	470.5207325	444.1407726	443.6109016	275.2630342	324.6510345	254.5050532
ENSG00000146373	RNF217	chr6:124962545-125092633	+	protein_coding	-0.678087175	0.137176746	-4.943164151	7.68647E-07	7.14395E-06	Yes	OK	564.9918532	697.1443197	432.8393867	703.6805598	682.6163585	705.1360408	502.6542363	351.5733154	444.2906084
ENSG00000205133	TRIQK	chr8:92883530-93017673	-	protein_coding	-0.677349748	0.210742012	-3.21411826	0.001308458	0.006286402	Yes	OK	565.9455301	698.2180603	433.6729999	751.9929565	640.2029154	702.458309	543.1611711	254.9698369	502.8879918
ENSG00000187187	ZNF546	chr19:39984134-40021041	+	protein_coding	-0.677149707	0.219367952	-3.086821479	0.002023091	0.009176101	Yes	OK	84.09286647	103.7892244	64.39650857	105.0269492	99.23145189	107.109272	68.12529943	58.59555257	66.4686737
ENSG00000159403	C1R	chr12:7080209-7092607	-	protein_coding	-0.676844307	0.113005073	-5.989503722	2.10482E-09	2.7366E-08	Yes	OK	5223.74747	6424.182485	4023.312454	6197.640274	6479.653757	6595.253424	3413.629869	4798.500656	3857.806838
ENSG00000071246	VASH1	chr14:76762189-76783015	+	protein_coding	-0.676834667	0.192324175	-3.519238631	0.000432787	0.002352602	Yes	OK	124.381344	152.7227295	96.03995849	152.2890764	141.644895	164.2342171	100.3467248	102.9381329	84.83501775
ENSG00000187391	MAGI2	chr7:78017057-79453574	-	protein_coding	-0.676378334	0.143765809	-4.704723176	2.54211E-06	2.15909E-05	Yes	OK	289.2386317	355.8877172	222.5895463	335.035968	396.1255539	336.5016296	231.9942629	215.3782473	220.3961286
ENSG00000154240	CEP112	chr17:65635538-66192084	-	protein_coding	-0.676108914	0.152986631	-4.41939867	9.89759E-06	7.60926E-05	Yes	OK	309.9693338	382.755452	237.1832157	375.9964782	392.9245393	379.3453384	264.2156883	188.4559664	258.8779923
ENSG00000181852	RNF41	chr12:56202175-56221933	-	protein_coding	-0.675974573	0.094291415	-7.168993847	7.5551E-13	1.40358E-11	Yes	OK	2021.4659	2484.907083	1558.024717	2621.472653	2344.743178	2488.50542	1514.406994	1700.854688	1458.81247
ENSG00000152380	FAM151B	chr5:80487969-80542563	+	protein_coding	-0.675197292	0.256798691	-2.629286345	0.008556428	0.031290559	Yes	OK	75.64107111	93.79420019	57.48794203	86.12209837	102.4324665	92.82803574	65.36346297	38.00792599	69.09243713
ENSG00000147883	CDKN2B	chr9:22002903-22009363	-	protein_coding	-0.674653539	0.13535245	-4.984420583	6.21478E-07	5.87229E-06	Yes	OK	443.1128714	543.525309	342.7004338	548.240675	560.177551	522.1577011	347.9913944	387.9975778	292.1123291
ENSG00000125457	MIF4GD	chr17:75266228-75271227	-	protein_coding	-0.674548786	0.196450182	-3.433688778	0.000595427	0.003129226	Yes	OK	125.9299222	155.3635135	96.49633096	147.0377289	174.4552944	144.5975172	102.1879491	82.35050631	104.9505374
ENSG00000197063	MAFG	chr17:81918270-81927714	-	protein_coding	-0.673622347	0.101324962	-6.64813817	2.96824E-11	4.6575E-10	Yes	OK	1532.701222	1882.290397	1183.112046	1879.982391	1878.195303	1888.693496	1049.497856	1311.273447	1188.564836
ENSG00000206503	HLA-A	chr6:29941260-29945884	+	protein_coding	-0.672778575	0.110914662	-6.065731665	1.31354E-09	1.73941E-08	Yes	OK	9003.34953	11062.04841	6944.650651	10757.91041	11299.58146	11128.65336	6110.102869	8391.833326	6332.015757
ENSG00000178878	APOLD1	chr12:12725917-12829975	+	protein_coding	-0.671154021	0.14664217	-4.576814571	4.7211E-06	3.8489E-05	Yes	OK	295.87683	363.8245383	227.9291218	331.8851596	348.1103352	411.47812	237.5179359	215.3782473	230.8911823
ENSG00000137502	RAB30	chr11:82973133-83071923	-	protein_coding	-0.670448432	0.135461154	-4.949377815	7.44511E-07	6.94314E-06	Yes	OK	884.8377504	1088.930674	680.7448266	1158.44725	1051.533289	1056.811484	787.1233921	543.196609	711.9144788
ENSG00000175087	PDIK1L	chr1:26111165-26125543	+	protein_coding	-0.670144558	0.234629497	-2.85618205	0.004287692	0.017447568	Yes	OK	137.548041	169.7745906	105.3214914	135.4847645	202.464172	171.3748352	118.7589679	71.26486123	125.9406449
ENSG00000143162	CREG1	chr1:167529677-167553767	-	protein_coding	-0.669541615	0.092511464	-7.237390753	4.57399E-13	8.68372E-12	Yes	OK	1464.169794	1799.571039	1128.768549	1831.669995	1830.980338	1736.062784	1196.795801	1042.050638	1147.459209
ENSG00000187049	TMEM216	chr11:61391687-61398863	+	protein_coding	-0.668660195	0.184010294	-3.633819503	0.000279256	0.001583928	Yes	OK	147.8864073	181.528822	114.2439926	166.9928493	177.656309	199.9373078	127.9650895	112.4401144	102.326774
ENSG00000215252	GOLGA8B	chr15:34525207-34588503	-	protein_coding	-0.668654691	0.236849782	-2.823117196	0.004755918	0.019093272	Yes	OK	88.96394928	109.2929457	68.6349529	134.434495	87.22764722	106.2166947	69.04591158	71.26486123	65.59408589
ENSG00000188227	ZNF793	chr19:37506939-37548762	+	protein_coding	-0.66773079	0.230546767	-2.89629215	0.003776007	0.015639928	Yes	OK	118.3145878	146.0039699	90.6252057	153.3393459	132.0418513	152.6307126	122.4414165	68.09753407	81.3366665
ENSG00000038002	AGA	chr4:177430770-177442503	-	protein_coding	-0.666565492	0.133807686	-4.981518702	6.30872E-07	5.9508E-06	Yes	OK	494.8265701	608.6457267	381.0074135	610.206575	641.8034227	573.9271825	422.5609789	319.9000438	400.5612178
ENSG00000189227	C15orf61	chr15:67521068-67530143	+	protein_coding	-0.665665764	0.188812577	-3.525537207	0.000422625	0.002304972	Yes	OK	157.7026446	192.6686902	122.7365989	216.3555154	192.0608746	169.5896807	112.3146828	145.6970496	110.1980643
ENSG00000011638	TMEM159	chr16:21158377-21180616	+	protein_coding	-0.665631702	0.133680957	-4.979255942	6.38292E-07	6.01734E-06	Yes	OK	441.2147773	542.313866	340.1156886	521.9839377	572.9816093	531.976051	385.7364927	296.14509	338.4654832
ENSG00000107798	LIPA	chr10:89213569-89414557	-	protein_coding	-0.663961273	0.135903825	-4.885523086	1.03155E-06	9.412E-06	Yes	OK	3033.508161	3722.001145	2345.015176	3629.731365	3850.820536	3685.451535	2689.108103	1756.282914	2589.654511
ENSG00000104689	TNFRSF10A	chr8:23190452-23225126	-	protein_coding	-0.662924741	0.236566573	-2.802275626	0.00507435	0.020155357	Yes	OK	86.71155359	106.8079497	66.61515749	115.5296442	104.0329738	100.8612311	57.99856573	57.01188899	84.83501775
ENSG00000084764	MAPRE3	chr2:26970612-27027196	+	protein_coding	-0.662779737	0.122000838	-5.432583478	5.5544E-08	6.07477E-07	Yes	OK	427.7363109	523.5939937	331.8786281	508.3304343	508.1610641	554.2904827	322.2142541	351.5733154	321.8483147
ENSG00000072415	MPP5	chr14:67241109-67335819	+	protein_coding	-0.662757447	0.127750218	-5.187916356	2.1266E-07	2.15011E-06	Yes	OK	1446.867385	1775.503796	1118.230974	1807.513796	1814.175012	1704.82258	1328.443339	913.7738874	1112.475697
ENSG00000091986	CCDC80	chr3:112596794-112649530	-	protein_coding	-0.660708514	0.056021704	-11.79379539	4.2017E-32	2.08942E-30	Yes	OK	59873.00662	73351.20067	46394.81256	73682.7065	72897.50522	73473.39029	47976.78182	44214.30357	46993.3523
ENSG00000112078	KCTD20	chr6:36442767-36491143	+	protein_coding	-0.66046583	0.102791034	-6.425325276	1.31588E-10	1.9287E-09	Yes	OK	4252.10273	5211.469305	3292.736155	5498.160792	5228.857312	4907.389813	3641.941683	2815.75385	3420.512932
ENSG00000112425	EPM2A	chr6:145382535-145736023	-	protein_coding	-0.660366958	0.185466786	-3.560567208	0.000370055	0.002042622	Yes	OK	170.9688759	210.6290719	131.3086798	230.0090188	199.2631574	202.6150396	149.139169	101.3544693	143.4324011
ENSG00000205795	CYS1	chr2:10056780-10080944	-	protein_coding	-0.660166078	0.205268242	-3.216114057	0.001299392	0.006250143	Yes	OK	107.4979668	131.8024754	83.19345818	134.434495	120.8383003	140.1346309	73.64897236	82.35050631	93.58089586
ENSG00000164932	CTHRC1	chr8:103371515-103382997	+	protein_coding	-0.658448062	0.102707166	-6.41092621	1.44638E-10	2.11059E-09	Yes	OK	5552.233352	6800.578611	4303.888094	6744.83068	6935.798335	6721.106819	4784.421367	3601.250988	4525.991926
ENSG00000115232	ITGA4	chr2:181457202-181536187	+	protein_coding	-0.657772455	0.117252999	-5.609856139	2.02495E-08	2.33899E-07	Yes	OK	4424.128759	5418.374197	3429.883321	5583.232621	5506.545326	5165.344643	3847.238194	2742.905326	3699.506444
ENSG00000140403	DNAJA4	chr15:78264086-78282196	+	protein_coding	-0.6576059	0.203524586	-3.23108825	0.001233199	0.005977164	Yes	OK	186.7133338	227.2736718	146.1529958	249.9641392	225.6715277	206.1853486	112.3146828	193.2069571	132.9373474
ENSG00000206530	CFAP44	chr3:113286947-113441610	-	protein_coding	-0.656041376	0.254060393	-2.582226092	0.009816525	0.035156659	Yes	OK	118.2883652	145.7004936	90.8762369	131.2836865	157.6499679	148.1678263	125.203253	53.84456182	93.58089586
ENSG00000183605	SFXN4	chr10:119140767-119165667	-	protein_coding	-0.654434186	0.224605657	-2.913703038	0.003571695	0.014913774	Yes	OK	181.5061433	223.2959929	139.7162937	226.8582103	244.8776151	198.1521532	165.7101878	85.51783348	167.9208599
ENSG00000175197	DDIT3	chr12:57516588-57521737	-	protein_coding	-0.654092321	0.139436399	-4.690972547	2.71909E-06	2.30228E-05	Yes	OK	728.0702531	888.321119	567.8193872	882.2263735	933.8960028	848.8409807	473.1946474	688.8936586	541.3698555
ENSG00000196683	TOMM7	chr7:22812628-22822852	-	protein_coding	-0.653782717	0.09419418	-6.94079735	3.89893E-12	6.72004E-11	Yes	OK	1287.395562	1572.728536	1002.062587	1539.695076	1531.685475	1646.805057	972.1664351	1073.723909	960.2974174
ENSG00000165516	KLHDC2	chr14:49767608-49786385	+	protein_coding	-0.653520589	0.140335517	-4.65684385	3.21094E-06	2.68967E-05	Yes	OK	890.6705303	1091.172507	690.1685541	1077.576499	1089.14521	1106.795811	734.6484993	532.1109639	803.7461991

ENSG00000026559	KCNG1	chr20:51003656-51023129	-	protein_coding	-0.653214809	0.230818858	-2.829988917	0.004654962	0.018747411	Yes	OK	293.1324661	356.7345731	229.530359	365.4937833	320.9017113	383.8082247	161.107127	342.0713339	185.4126161
ENSG00000167740	CYB5D2	chr17:4143168-4187310	+	protein_coding	-0.65270202	0.136360039	-4.786607746	1.69624E-06	1.49305E-05	Yes	OK	466.6633058	571.2523815	362.07423	595.5028021	596.9892186	521.2651238	334.1822121	335.7366796	416.3037984
ENSG00000144681	STAC	chr3:36380344-36548007	+	protein_coding	-0.652313358	0.104248712	-6.257279757	3.9175E-10	5.45996E-09	Yes	OK	708.7614166	865.8288855	551.6939477	845.4669413	913.8896617	838.1300535	538.5581104	563.7842355	552.7394971
ENSG00000121039	RDH10	chr8:73294612-73325281	+	protein_coding	-0.652132462	0.150087079	-4.345027352	1.39258E-05	0.000104523	Yes	OK	615.2751335	750.0528171	480.4974498	652.2173547	825.8617609	772.0793358	417.037306	582.7881985	441.666845
ENSG00000183671	GPR1	chr2:206175316-206218047	-	protein_coding	-0.651830215	0.152186372	-4.283105035	1.84303E-05	0.000134945	Yes	OK	631.7033707	772.7833853	490.6233561	699.4794819	837.8655655	781.0051084	609.4452463	426.0055038	436.4193181
ENSG00000196911	KPNA5	chr6:116681187-116741866	+	protein_coding	-0.651572795	0.222917925	-2.922926884	0.003467579	0.01456401	Yes	OK	171.400841	210.8218078	131.9798743	211.104168	216.0684839	205.2927714	146.3773326	80.76684273	168.7954477
ENSG00000221963	APOL6	chr22:35648395-35668409	+	protein_coding	-0.650666636	0.117516242	-5.536823028	3.08007E-08	3.46535E-07	Yes	OK	1629.190089	1992.961233	1265.418945	2119.443835	1880.596064	1978.8438	1383.680068	1049.968956	1362.607811
ENSG00000124313	IQSEC2	chrX:53225828-53321350	-	protein_coding	-0.649247452	0.154051818	-4.214474464	2.5036E-05	0.000178473	Yes	OK	484.4285743	589.5675674	379.2895812	554.5422919	608.9930233	605.1673869	332.3409878	476.6827385	328.8450172
ENSG00000126709	IFI6	chr1:27666061-27672218	-	protein_coding	-0.648844215	0.213835586	-3.034313552	0.002410837	0.010678789	Yes	OK	531.50649	647.0584471	415.9545328	619.6590005	637.8021545	683.7141864	304.7226231	616.0451338	327.0958416
ENSG00000162645	GBP2	chr1:89106132-89150456	-	protein_coding	-0.648483736	0.12743974	-5.088551925	3.60808E-07	3.51831E-06	Yes	OK	571.8499651	699.6487558	444.0511743	729.9372971	729.0310699	639.9779003	450.1793435	389.5812414	492.392938
ENSG00000147162	OGT	chrX:71533083-71575897	+	protein_coding	-0.648476735	0.084561667	-7.668684384	1.73769E-14	3.67641E-13	Yes	OK	3642.558201	4450.111518	2835.004884	4488.85181	4549.441968	4312.040776	3001.195624	2568.702332	2935.116696
ENSG00000146733	PSPH	chr7:56011051-56051604	-	protein_coding	-0.647814978	0.107822746	-6.00814766	1.87655E-09	2.45338E-08	Yes	OK	683.5850347	833.482916	533.6871535	839.1653243	833.8642973	827.4191263	492.5275026	573.286217	535.2477408
ENSG00000116183	PAPPA2	chr1:176463171-176845605	+	protein_coding	-0.647566644	0.240409697	-2.693596189	0.007068575	0.026734545	Yes	OK	82.00836113	99.84052023	64.17620203	106.0772187	87.22764722	106.2166947	49.71305634	72.84852482	69.96702494
ENSG00000125398	SOX9	chr17:72121020-72126420	+	protein_coding	-0.647014797	0.16657806	-3.884153744	0.000102687	0.000640723	Yes	OK	549.5484124	668.647995	430.4488299	648.0162767	662.6100174	695.3176908	382.9746563	563.7842355	344.5875979
ENSG00000172007	RAB33B	chr4:139453232-139476609	+	protein_coding	-0.646949915	0.192356948	-3.363278118	0.000770227	0.003947055	Yes	OK	316.9157036	387.9485048	245.8829024	420.1077969	361.7146472	382.0230702	331.4203756	188.4559664	217.7723651
ENSG00000072840	EVC	chr4:5711197-5814305	+	protein_coding	-0.646320598	0.079614077	-8.118169851	4.73266E-16	1.08495E-14	Yes	OK	2505.495292	3056.426656	1954.563928	3098.295002	2927.327831	3143.657134	1897.38165	2041.342358	1924.967774
ENSG00000122435	TRMT13	chr1:100133150-100150497	+	protein_coding	-0.645750435	0.260277848	-2.481004198	0.013101283	0.044244382	Yes	OK	238.1466655	291.9454944	184.3478367	288.8241104	288.8915656	298.1208071	243.9622209	88.68516065	220.3961286
ENSG00000079102	RUNX1T1	chr8:91954967-92103286	-	protein_coding	-0.645410028	0.098315602	-6.564675515	5.21463E-11	7.97121E-10	Yes	OK	881.3852728	1076.106964	686.6635813	1116.43647	1059.535825	1052.348598	676.6499335	663.5550413	719.7857691
ENSG00000133112	TPT1	chr13:45333471-45341370	-	protein_coding	-0.645316083	0.117104357	-5.510606949	3.57598E-08	3.99056E-07	Yes	OK	17605.95384	21482.21052	13729.69716	21230.14752	21704.47934	21512.00471	15500.34684	10691.31285	14997.4318
ENSG00000101194	SLC17A9	chr20:62952647-62969585	+	protein_coding	-0.64511062	0.155581041	-4.146460361	3.37655E-05	0.0002359	Yes	OK	1052.994407	1282.301895	823.6869203	1225.664498	1255.597968	1365.643218	657.3170783	1064.221928	749.5217547
ENSG00000175274	TP53I11	chr11:44885903-44951306	-	protein_coding	-0.644248491	0.139650837	-4.613280565	3.96363E-06	3.27517E-05	Yes	OK	1725.658363	2102.229023	1349.087703	2011.266078	2101.46607	2193.954922	1090.925403	1688.185379	1268.152327
ENSG00000163412	EIF4E3	chr3:71675416-71754773	-	protein_coding	-0.642401989	0.244749994	-2.624727293	0.008671839	0.031670396	Yes	OK	166.3834493	204.0863128	128.6805857	215.3052459	203.2644256	193.6892669	176.7575337	72.84852488	136.4356986
ENSG00000122642	FKBP9	chr7:32957404-33006931	+	protein_coding	-0.642219408	0.071510527	-8.980767351	2.68885E-19	7.50143E-18	Yes	OK	11254.14091	13715.45382	8792.828007	13648.25205	13689.93909	13808.17032	8381.253054	9506.732488	8490.498477
ENSG00000026950	BTN3A1	chr6:26402237-26415216	+	protein_coding	-0.641753022	0.127737607	-5.023994405	5.06077E-07	4.8515E-06	Yes	OK	366.1395457	445.555413	286.7236784	462.1185766	442.5402653	432.0073971	269.7393613	308.8143987	281.6172754
ENSG00000134882	UBAC2	chr13:99200774-99386434	+	protein_coding	-0.64051872	0.088543959	-7.23390649	4.69295E-13	8.8993E-12	Yes	OK	1389.177227	1692.539228	1085.815226	1726.643045	1614.911854	1736.062784	1047.656632	1099.062527	1110.726521
ENSG00000123095	BHLHE41	chr12:26120026-26125127	-	protein_coding	-0.639772142	0.131239625	-4.874839771	1.08897E-06	9.88115E-06	Yes	OK	434.225692	530.4994245	337.9519595	542.9893275	512.962586	535.5463601	361.8005767	288.2267721	363.8285297
ENSG00000188452	CERKL	chr2:181536676-181680665	-	protein_coding	-0.638909497	0.145411157	-4.393813435	1.11379E-05	8.47967E-05	Yes	OK	801.1534523	977.6208578	624.6860469	985.1527838	927.4939737	1020.215816	750.2989059	486.18472	637.5745148
ENSG00000171298	GAA	chr17:80101556-80119879	+	protein_coding	-0.637535812	0.117812621	-5.411439003	6.25203E-08	6.77475E-07	Yes	OK	1821.458071	2214.955926	1427.960216	2168.806502	2175.889659	2300.171616	1242.826409	1697.687361	1343.366879
ENSG00000031698	SARS	chr1:109213918-109238169	+	protein_coding	-0.637513138	0.070180989	-9.083843754	1.04807E-19	3.02652E-18	Yes	OK	6931.03299	8435.498261	5426.567718	8392.703513	8646.740626	8267.050645	5273.266421	5759.784451	5246.652283
ENSG00000169760	NLGN1	chr3:173396284-174286644	+	protein_coding	-0.636552926	0.133995518	-4.75055387	2.0286E-06	1.75464E-05	Yes	OK	794.1326049	968.5745718	619.690638	987.2533228	941.0982856	977.3721071	715.315644	495.6867015	648.0695686
ENSG00000151914	DST	chr6:56547987-56954628	-	protein_coding	-0.636309099	0.099862755	-6.371835996	1.86779E-10	2.70156E-09	Yes	OK	18910.34301	23017.50903	14803.177	23993.40655	22939.27071	22119.84983	16491.84614	12393.7512	15523.93366
ENSG00000160352	ZNF714	chr19:21082159-21125270	+	protein_coding	-0.635913071	0.190986056	-3.329630892	0.000869612	0.004392087	Yes	OK	232.7099283	284.6760475	180.7438092	318.2316562	260.882688	274.9137982	215.4232441	128.2767502	198.5314333
ENSG00000182957	SPATA13	chr13:23979805-24307074	+	protein_coding	-0.635803376	0.159721478	-3.980700547	6.87125E-05	0.000445454	Yes	OK	229.5059012	280.4058969	178.6059056	299.3268053	277.6880146	264.202871	185.9636552	150.4480404	199.4060211
ENSG00000177694	NAALADL2	chr3:174438573-175810552	+	protein_coding	-0.63541025	0.141489011	-4.490880566	7.09293E-06	5.63736E-05	Yes	OK	398.4030455	486.1514544	310.6546367	520.9336682	453.7438163	483.7768786	353.5150673	264.4718184	313.9770244
ENSG00000166710	B2M	chr15:44711477-44718877	+	protein_coding	-0.635096112	0.107912464	-5.885289707	3.97357E-09	4.96244E-08	Yes	OK	25957.2538	31583.24833	20331.25927	31871.47801	31908.51356	30969.75343	23285.04322	16435.26066	21273.47393
ENSG00000110619	CARS	chr11:3000922-3057613	-	protein_coding	-0.633898431	0.0815503	-7.773097522	7.65897E-15	1.64793E-13	Yes	OK	3193.684605	3881.787997	2505.581213	3812.478257	3918.041842	3914.843892	2431.3367	2714.399381	2371.007558
ENSG00000122644	ARL4A	chr7:12686856-12690934	+	protein_coding	-0.632369245	0.132399013	-4.776238354	1.78605E-06	1.56457E-05	Yes	OK	317.9304597	386.7778333	249.083086	410.6553715	372.1179446	377.5601838	257.7714032	245.4678554	244.0099995
ENSG00000115944	COX7A2L	chr2:42333546-42425088	-	protein_coding	-0.63225643	0.091759166	-6.890389905	5.56397E-12	9.39312E-11	Yes	OK	1681.34393	2044.139065	1318.548796	1916.741823	2204.69879	2010.976582	1307.269259	1287.518493	1360.858635
ENSG00000100592	DAAM1	chr14:59188646-59371405	+	protein_coding	-0.630817204	0.142298156	-4.433066621	9.29021E-06	7.17246E-05	Yes	OK	574.7382202	700.1240732	449.3523671	673.2227466	720.2282798	706.9211953	523.8283159	356.3243062	467.9044793
ENSG00000253250	C8orf88	chr8:90958637-90985257	-	protein_coding	-0.630548855	0.207778421	-3.034717713	0.002407609	0.010670233	Yes	OK	167.9025756	202.6182269	133.1869242	197.4506646	210.4667084	199.9373078	123.3620287	177.3703213	98.82842273
ENSG00000100802	C14orf93	chr14:22985908-23010166	-	protein_coding	-0.63035604	0.187763479	-3.357181304	0.000787415	0.004023857	Yes	OK	130.9141528	158.6594323	103.1688733	140.736112	165.6525044	169.5896807	104.0291735	109.2727872	96.2046593
ENSG00000196507	TCEAL3	chrX:103607451-103629690	+	protein_coding	-0.629987149	0.145631192	-4.325908801	1.51905E-05	0.000113138	Yes	OK	545.5518299	664.4879171	426.6157426	720.4848717	656.2079883	616.7708913	500.813012	345.2386611	433.7955547
ENSG00000151892	GFRA1	chr10:116056925-116273467	-	protein_coding	-0.629801388	0.082909568	-7.59624499	3.04847E-14	6.28795E-13	Yes	OK	2008.645795	2441.852334	1575.439257	2446.077648	2439.173108	2440.306247	1655.260654	1477.558123	1593.498993
ENSG00000169660	HEXDC	chr17:82418318-82442645	+	protein_coding	-0.62827893	0.212956284	-2.950271856	0.003174944	0.013528238	Yes	OK	114.806995	138.6892624	90.92472749	136.535034	145.6461633	133.88659	69.04591158	109.2727872	94.45548368
ENSG00000198597	ZNF536	chr19:30228290-30713538	+	protein_coding	-0.627419286	0.149766098	-4.18932786	2.79782E-05	0.000197903	Yes	OK	274.8998113	332.658572	217.1410506	333.9856986	325.7032332	338.2867841	187.8048795	250.2188461	213.3994261
ENSG00000180346	TIGD2	chr4:89111500-89114899	+	protein_coding	-0.626934403	0.1821657	-3.441561182	0.000578368	0.003050283	Yes	OK	266.5187955	324.9322255	208.1053655	316.1311172	340.9080525	317.757507	255.9301789	148.8643768	219.5215408
ENSG00000251322	SHANK3	chr22:50674415-50733298	+	protein_coding	-0.626419355	0.162467633	-3.855656305	0.00011542	0.000712873	Yes	OK	227.1620907	274.5556784	179.7685029	280.4219544	273.6867463	269.5583346	174.9163093	212.2109201	152.1782793
ENSG00000251192	ZNF674	chrX:46497727-46545457	-	protein_coding	-0.626359292	0.209016501	-2.996697824	0.002729211	0.011912705	Yes	OK	106.8478229	130.0236097	83.67203605	139.6858425	112.0355102	138.3494764	78.25203313	79.18317915	93.58089586
ENSG00000172123	SLFN12	chr17:35411060-35433283	-	protein_coding	-0.62632983	0.13393034	-4.676534325	2.91764E-06	2.45523E-05	Yes	OK	343.8931615	418.1395715	269.6467516	443.2137257	411.3303731	399.8746155	294.5958894	250.2188461	264.1255192
ENSG00000110881	ASIC1	chr12:50057548-50083611	+	protein_coding	-0.62485296	0.167081136	-3.739817524	0.000184154	0.001088844	Yes	OK	187.2323641	227.1046324	147.3600959	228.9587493	210.4667084	241.8884393	162.0277392	148.8643768	131.1881718
ENSG00000153879	CEBPG	chr19:33373330-33382686	+	protein_coding	-0.624629696	0.145716838	-4.286599291	1.81429E-05	0.000133031	Yes	OK	1303.803724	1583.800806	1023.806641	1600.610706	1655.72479	1495.066922	1228.096614	772.8278285	1070.495482
ENSG00000113456	RAD1	chr5:34905264-34918989	-	protein_coding	-0.623890922	0.105556358	-5.910500633	3.4107E-09	4.32512E-08	Yes	OK	1006.459937	1222.535269	790.3846046	1213.061264	1245.994924	1208.549619	850.6456307	698.3956401	822.1125431
ENSG00000213047	DENND1B	chr1:197504748-197775696	-	protein_coding	-0.622811949	0.150657306	-4.133964476	3.56559E-05	0.000247531	Yes	OK	523.038303	635.7721226	410.3044834	593.4022631	641.8034227	672.1106819	487.0038297	321.4837073	422.4259131
ENSG00000011258	MBTD1	chr17:51177425-51260163	-	protein_coding	-0.622396862	0.160888669	-3.868494064	0.00010951	0.000679943	Yes	OK	257.1448956	312.9655955	201.3241957	312.9803087	292.0925802	333.8238978	231.9942629	164.7010126	207.2773114
ENSG00000166780	C16orf45	chr16:15434295-15625028	+	protein_coding	-0.622360769	0.083079115	-7.49118195	6.82561E-14	1.37683E-12	Yes	OK	3100.169387	3756.405426	2443.933147	3582.469238	3807.606839	3879.140801	2366.893849	2628.881548	2336.024045
ENSG00000121988	ZRANB3	chr2:135136916-135531236	-	protein_coding	-0.621858101	0.174439021	-3.564902496	0.000363991	0.002014559	Yes	OK	158.2683476	192.6817174	123.8549778	201.6517425	188.0596064	188.3338033	131.6475381	106.1054601	133.8119352
ENSG00000162836	ACP6	chr1:147629652-147670496	-	protein_coding	-0.621530485	0.206127061	-3.015278459	0.002567434	0.011296436	Yes	OK	156.3854112	189.2611233	123.5096991	215.3052459	163.2517434	189.2263806	92.9818276	139.3623953	138.1848743
ENSG00000213625	LEPROT	chr1:65420587-65436007	+	protein_coding	-0.621463063	0.107308264	-5.791381227	6.98099E-09	8.47398E-08	Yes	OK	3890.350632	4718.391952	3062.309312	4738.815949	4675.882043	4740.477864	3371.28171	2530.694406	3284.951821
ENSG00000138303	ASCC1	chr10:72096032-72217134	-	protein_coding	-0.619044593	0.11478071	-5.393280747	6.91827E-08	7.4623E-07	Yes	OK	563.4164769	683.2166572	443.6162967	656.4184327	720.2282798	673.0032591	470.4328109	411.7525316	448.6635475
ENSG00000112290	WASF1	chr6:110099819-110180004	-	protein_coding	-0.618693024	0.098241032	-6.297704863	3.02085E-10	4.27175E-09	Yes	OK	1183.978903	1434.748673	933.2091337	1557.549655	1382.838297	1363.858064	917.850318	915.3575509	966.419532
ENSG00000148572	NRBF2	chr10:63133247-63155031	+	protein_coding	-0.618659042	0.158551116	-3.901953244	9.54196E-05	0.000599468	Yes	OK	451.6009946	548.7749723	354.4270169	588.1509157	552.9752682	505.198733	397.7044507	267.6391455	397.9374544
ENSG00000144741	SLC25A26	chr3:66133610-66388116	+	protein_coding	-0.618576809	0.176934327	-3.496081397	0.000472145	0.00254303	Yes	OK	214.3363383	260.3841733	168.2885032	235.2603663	281.6892828	264.202871	159.2659027	140.9460589	204.653548
ENSG00000198919	DZIP3	chr3:108589682-108694846	+	protein_coding	-0.617435717	0.14708437	-4.197833648	2.6948E-05	0.000191274	Yes	OK	491.989934	597.5907674	386.3891007	614.407653	586.5859212	591.7787279	422.5609789	299.3124172	437.2939059
ENSG00000125454	SLC25A19	chr17:75272981-75289510	-	protein_coding	-0.617407062	0.222024264	-2.780808954	0.005422364	0.021314008	Yes	OK	89.00654222	108.1111539	69.90193058	98.72533228	102.4324665	123.1756628	72.7283602	61.76287974	75.21455182
ENSG00000198682	PAPSS2	chr10:87659613-87747705	+	protein_coding	-0.614572155	0.081150076	-7.57327881	3.6392E-14	7.46898E-13	Yes	OK	3761.224748	4552.871808	2969.577688	4608.582532	4609.460991	4440.571902	3092.336227	2715.983045	3100.413793
ENSG00000115504	EHBP1	chr2:62673851-63046487	+	protein_coding	-0.614340754	0.123809231	-4.961994741	6.97729E-07	6.53649E-06	Yes	OK	3207.791578	3883.092236	2532.49092	3932.208979	4024.475577	3692.592153	2946.879506	2012.836414	2637.75684
ENSG00000010318	PHF7	chr3:52410657-52423641	+	protein_coding	-0.614248593	0.188224342	-3.263385519	0.001100897	0.005423752	Yes	OK	125.4075017	151.9430307	98.87197266	152.2890764	146.4464169	157.093599	110.4734585	93.43615139	92.70630805
ENSG00000137331	IER3	chr6:30743199-30744554	-	protein_coding	-0.614174524	0.119912795	-5.121843139	3.02564E-07	2.98037E-06	Yes	OK	630.6657054	761.1857158	500.145695	721.5351412	789.0500932	772.971913	457.5442408	565.3678991	477.5249452
ENSG00000128340	RAC2	chr22:37225261-37244448	-	protein_coding	-0.614013518	0.156652741	-3.919583624	8.87021E-05	0.000561552	Yes	OK	479.2556554	577.8835291	380.6277816	607.0557665	563.3785656	563.2162553	317.6111933	481.4337292	342.8384222
ENSG00000175938	ORAI3	chr16:30949066-30956461	+	protein_coding	-0.61306691	0.205652593	-2.98108038	0.002872334	0.012425392	Yes	OK	187.1905064	225.1932366	149.1877762	192.1993171	243.2771079	240.1032848	109.5528464	188.4559664	149.5545158
ENSG00000132613	MTSS1L	chr16:70661204-70686066	-	protein_coding	-0.612665494	0.096764579	-6.331505814	2.4278E-10	3.46588E-09	Yes	OK	2334.241922	2820.317074	1848.166771	2863.034636	2764.876341	2833.040245	1663.546163	2071.431967	1809.522183
ENSG00000118785	SPP1	chr4:87975650-87983426	+	protein_coding	-0.611569709	0.185888779	-3.289976477	0.001001958	0.004987064	Yes	OK	151.677763	183.2404081	120.1151179	191.1490476	156.8497143	201.7224623	107.7116221	126.6930866	125.9406449
ENSG00000050426	LETMD1	chr12:51047962-51060424	+	protein_coding	-0.609767094	0.124507153	-4.89744627	9.70902E-07	8.87836E-06	Yes	OK	604.7819424	729.2862147	480.27767	727.8367582	783.4483177	676.5735682	433.6083248	538.4456182	468.7790671
ENSG00000167969	ECI1	chr16:2239395-2252300	-	protein_coding	-0.609372517	0.125148349	-4.869201401	1.1205E-06	1.01393E-05	Yes	OK	605.2938958	731.3134019	479.2743896	656.4184327	753.8389329	783.6828402	440.973222	470.3480841	526.5018627
ENSG00000110721	CHKA	chr11:68052859-68121444	-	protein_coding	-0.60915355	0.215471219	-2.827076177	0.004697515	0.018909538	Yes	OK	155.2877724	188.4028088	122.172736	175.3950052	226.4717813	163.3416398	123.3620287	91.85248781	151.3036914
ENSG00000184575	XPOT	chr12:64404350-64451127	+	protein_coding	-0.609108885	0.10445589	-5.831254535	5.50122E-09	6.75747E-08	Yes	OK	5666.953008	6848.758909	4485.147108	6933.879188	6810.958766	6801.438773	4975.908695	3720.025756	4759.506872
ENSG00000117228	GBP1	chr1:89052319-89065360	-	protein_coding	-0.60904554	0.136016577	-4.477730256	7.54408E-06	5.94996E-05	Yes	OK	1433.828997	1734.336731	1133.321263	1751.849513	1744.552944	1706.607734	1325.681502	872.5986342	1201.683653
ENSG00000111110	PPM1H	chr12:62643982-62935037	-	protein_coding	-0.609036065	0.234753245	-2.594366973	0.009476528	0.034169474	Yes	OK	80.40917026	97.65260047	63.16574004	112.3788357	90.4286618	90.15030394	71.80774805	53.84456182	63.84491026
ENSG00000134717	BTF3L4	chr1:52056125-52090716	+	protein_coding	-0.60891142	0.151895692	-4.008747138	6.10417E-05	0.000399979	Yes	OK	618.65497	749.1989778	488.1109622	761.4453819	696.2206705	789.9308811	573.5413722	372.160942	518.6305724
ENSG00000223865	HLA-DPB1	chr6:33075926-33087201	+	protein_coding	-0.608264793	0.24318723	-2.501220117	0.012376623	0.042282941	Yes	OK	97.54462663	117.3549001	77.73435316	115.5296442	139.2441341	97.29092208	88.37876683	87.10149706	57.72279558

ENSG00000110768	GTF2H1	chr11:18322295-18367044	+	protein_coding	-0.60797009	0.089331216	-6.805796607	1.00492E-11	1.65409E-10	Yes	OK	1893.714212	2288.465481	1498.962944	2287.486954	2314.333539	2263.575949	1624.880453	1390.456626	1481.551753
ENSG00000115365	LANCL1	chr2:210431249-210477652	-	protein_coding	-0.607081827	0.0995407	-6.098830201	1.06847E-09	1.43347E-08	Yes	OK	1642.317805	1984.722516	1299.913095	2024.919581	2041.447046	1887.800919	1417.742718	1163.992733	1318.003832
ENSG00000069966	GNB5	chr15:52115105-52191369	-	protein_coding	-0.606129492	0.152495552	-3.974735543	7.04575E-05	0.000454796	Yes	OK	584.0934219	706.5926971	461.5941468	660.6195107	682.6163585	776.5422221	459.3854651	367.4099512	557.9870239
ENSG00000105810	CDK6	chr7:92604921-92836594	-	protein_coding	-0.605455446	0.111995795	-5.406055164	6.4428E-08	6.96313E-07	Yes	OK	2753.405225	3325.522085	2181.288366	3368.214262	3244.228274	3364.123718	2422.130578	1792.707176	2329.027343
ENSG00000113638	TTC33	chr5:40512333-40755975	-	protein_coding	-0.604930942	0.223263803	-2.709489553	0.006738683	0.025705381	Yes	OK	209.2138527	253.7744728	164.6532327	233.1598273	272.8864927	255.2770983	201.6140618	98.18714214	194.1584942
ENSG00000109738	GLRB	chr4:157076057-157172090	+	protein_coding	-0.604578086	0.171662285	-3.521903987	0.000428459	0.002331385	Yes	OK	231.6094173	280.0738927	183.144942	296.1759968	297.6943557	246.3513256	219.1056928	161.5336855	168.7954477
ENSG00000185630	PBX1	chr1:164555584-164899296	+	protein_coding	-0.604522695	0.083223067	-7.263883843	3.76131E-13	7.19897E-12	Yes	OK	3612.886873	4358.853739	2866.920007	4456.293456	4303.764099	4316.503662	3088.653778	2874.349403	2637.75684
ENSG00000158092	NCK1	chr3:136862208-136949823	+	protein_coding	-0.603806335	0.166154633	-3.634002399	0.000279058	0.00158335	Yes	OK	666.102895	805.2846821	526.9211079	758.2945734	850.6696238	806.8898492	631.539938	373.7446056	575.4787802
ENSG00000078098	FAP	chr2:162170684-162245151	-	protein_coding	-0.602657422	0.12066677	-4.994394258	5.90207E-07	5.59609E-06	Yes	OK	3474.583674	4192.507842	2756.659507	4236.787132	4250.947358	4089.789036	3189.921115	2196.54139	2883.516016
ENSG00000204852	TCTN1	chr12:110614027-110649430	+	protein_coding	-0.602471258	0.121411911	-4.962208824	6.9696E-07	6.53337E-06	Yes	OK	670.325828	806.4225524	534.2291037	800.3053531	790.6506005	828.3117036	495.2893391	617.6287974	489.7691746
ENSG00000111670	GNPTAB	chr12:101745497-101830938	-	protein_coding	-0.602256096	0.122280299	-4.925209529	8.427E-07	7.77514E-06	Yes	OK	1656.458916	1999.010721	1313.90711	2004.964461	2092.66328	1899.404424	1556.755153	1113.315499	1271.650678
ENSG00000159733	ZFYVE28	chr4:2269582-2418663	-	protein_coding	-0.602115853	0.154515727	-3.896793328	9.74747E-05	0.000610328	Yes	OK	312.1946243	374.9723795	249.416869	362.3429748	360.9143936	401.65977	215.4232441	299.3124172	233.5149458
ENSG00000164040	PGRMC2	chr4:128269237-128288829	-	protein_coding	-0.601020734	0.123611816	-4.862162473	1.1611E-06	1.04837E-05	Yes	OK	4298.040007	5183.2624	3412.817614	5206.185873	5180.041839	5163.559488	3952.187979	2663.722147	3622.542717
ENSG00000196470	SIAH1	chr16:48356364-48448402	-	protein_coding	-0.600995753	0.195224871	-3.078479445	0.002080599	0.009406899	Yes	OK	180.8167014	218.9855135	142.6478894	225.8079408	200.8636647	230.2849348	181.3605944	109.2727872	137.3102865
ENSG00000120053	GOT1	chr10:99396870-99430624	-	protein_coding	-0.6005239	0.092609906	-6.484445634	8.90586E-11	1.32781E-09	Yes	OK	1517.437633	1826.86075	1208.014515	1805.413257	1891.799615	1783.369379	1161.812539	1311.273447	1150.95756
ENSG00000110446	SLC15A3	chr11:60937084-60952530	-	protein_coding	-0.600385542	0.246906462	-2.431631551	0.015030989	0.049491914	Yes	OK	108.1616771	129.5289704	86.7943839	120.7809916	131.2415977	136.5643218	55.23672927	112.4401144	92.70630805
ENSG00000162636	FAM102B	chr1:108560089-108644900	+	protein_coding	-0.599692886	0.129945583	-4.61495398	3.93182E-06	3.25215E-05	Yes	OK	887.5392692	1070.809674	704.2688645	1003.007365	1065.1376	1144.284056	827.6303269	598.6248344	686.5514323
ENSG00000132950	ZMYM5	chr13:19823482-19863636	-	protein_coding	-0.599656483	0.153525814	-3.905900048	9.38753E-05	0.000590894	Yes	OK	297.0275165	359.0320099	235.0230231	347.639202	384.9220029	344.534825	266.9775248	193.2069571	244.8845873
ENSG00000121931	LRIF1	chr1:110947185-110964079	-	protein_coding	-0.599055608	0.153575845	-3.90071503	9.5909E-05	0.000601624	Yes	OK	339.2165195	409.2806247	269.1524143	430.6104918	400.9270758	396.3043064	324.9760905	245.4678554	237.013297
ENSG00000163947	ARHGEF3	chr3:56727418-57079329	-	protein_coding	-0.598154309	0.149833719	-3.992120818	6.5485E-05	0.000426546	Yes	OK	329.7127388	397.67218	261.7532976	349.7397409	450.5428017	392.7339974	274.342422	232.7985467	278.1189242
ENSG00000164751	PEX2	chr8:76980258-77001044	-	protein_coding	-0.597731423	0.168770803	-3.541675521	0.000397594	0.002177113	Yes	OK	671.5885346	810.6065637	532.5705054	772.9983463	847.4686093	811.3527355	631.539938	370.5772784	595.5942998
ENSG00000174963	ZIC4	chr3:147386046-147406860	-	protein_coding	-0.596923782	0.147914643	-4.035596282	5.44638E-05	0.000361336	Yes	OK	321.1608869	385.9797176	256.3420561	369.6948613	416.9321487	371.312143	217.2644685	277.141127	274.6205729
ENSG00000073060	SCARB1	chr12:124776856-124882668	-	protein_coding	-0.596701533	0.164006974	-3.63826926	0.000274476	0.001560581	Yes	OK	234.2016952	281.4572898	186.9461007	311.9300392	274.487	257.9548301	158.3452906	207.4599294	195.033082
ENSG00000151422	FER	chr5:108747822-109196841	+	protein_coding	-0.595779694	0.153411151	-3.883548812	0.000102943	0.000642076	Yes	OK	1489.609496	1795.160708	1184.058284	1815.915952	1778.163598	1791.402574	1389.203741	844.0926897	1318.87842
ENSG00000187134	AKR1C1	chr10:4963253-4983283	+	protein_coding	-0.595029683	0.144744434	-4.110898552	3.94122E-05	0.000271093	Yes	OK	366.1814617	439.0398315	293.3230919	427.4596834	435.3379825	454.3218288	276.1836463	348.4059883	255.379641
ENSG00000163617	CCDC191	chr3:113964137-114056613	-	protein_coding	-0.594942379	0.227677565	-2.613091801	0.00897272	0.032559737	Yes	OK	93.05288099	112.3492994	73.7564264	124.9820696	119.237793	92.82803574	69.04591158	66.51387048	85.70960556
ENSG00000073331	ALPK1	chr4:112285509-112442620	+	protein_coding	-0.594311906	0.120073259	-4.949577531	7.43747E-07	6.93996E-06	Yes	OK	427.1924957	513.6348025	340.750189	502.0288173	534.5694344	504.3061557	323.1348662	348.4059883	350.7097125
ENSG00000178467	P4HTM	chr3:48989886-49007154	+	protein_coding	-0.593596724	0.153136589	-3.876256664	0.000106076	0.000660116	Yes	OK	546.3089245	655.1024315	437.5154174	602.8546886	693.0196559	669.4329501	368.2448618	546.3639361	397.9374544
ENSG00000204084	INPP5B	chr1:37860697-37947057	-	protein_coding	-0.593400342	0.113410331	-5.232330593	1.67386E-07	1.71662E-06	Yes	OK	556.4168256	669.3296438	443.5040073	685.8259785	655.4077346	666.7552183	474.1152595	441.8421396	414.5546228
ENSG00000118523	CTGF	chr6:131948176-131951373	-	protein_coding	-0.593220459	0.054444568	-10.89586136	1.2062E-27	4.90223E-26	Yes	OK	45151.06435	54302.97185	35999.15684	54347.24515	54983.82739	53577.84302	35360.71285	37250.9348	35385.82287
ENSG00000153046	CDYL	chr6:4706159-4955551	+	protein_coding	-0.593052795	0.10050649	-5.900641766	3.6209E-09	4.55658E-08	Yes	OK	855.1890057	1029.810192	680.5678188	1060.772187	999.5168017	1029.141589	706.1095225	635.0490968	700.5448373
ENSG00000165997	ARL5B	chr10:18659405-18681639	+	protein_coding	-0.593048323	0.159824537	-3.710621238	0.000206751	0.001210646	Yes	OK	585.2499137	705.2698414	465.229986	635.4130428	730.6315772	749.7649041	573.5413722	359.4916333	462.6569524
ENSG00000164938	TP53INP1	chr8:94925972-94949411	-	protein_coding	-0.592936693	0.124168476	-4.775259498	1.79476E-06	1.5697E-05	Yes	OK	861.2082269	1037.226211	685.1902426	1008.258713	1032.327201	1071.09272	800.0119622	589.1228529	666.4359126
ENSG00000204262	COL5A2	chr2:189031896-189179879	-	protein_coding	-0.592767115	0.053749947	-11.02823626	2.79289E-28	1.15796E-26	Yes	OK	30540.4449	36728.80399	24352.09582	36206.99048	37175.78304	36803.63844	24518.66351	23902.23446	24635.38948
ENSG00000173848	NET1	chr10:5412551-5458463	+	protein_coding	-0.592669554	0.10539566	-5.623282325	1.87363E-08	2.16877E-07	Yes	OK	1085.250939	1306.615128	863.8867503	1300.233631	1378.036775	1241.574978	924.2946031	774.4114921	892.9541559
ENSG00000118762	PKD2	chr4:88007668-88077777	+	protein_coding	-0.592322938	0.077926094	-7.601085991	2.93656E-14	6.0953E-13	Yes	OK	4761.143587	5727.304265	3794.982909	5722.918464	5848.253632	5610.740699	4015.710218	3498.312855	3870.925655
ENSG00000112186	CAP2	chr6:17393216-17557792	+	protein_coding	-0.590659148	0.117227929	-5.038553129	4.69064E-07	4.52037E-06	Yes	OK	1547.001678	1861.592874	1232.410483	1902.038051	1895.800883	1786.939688	1390.124353	1021.463011	1285.644083
ENSG00000122786	CALD1	chr7:134744252-134970728	+	protein_coding	-0.58874117	0.112112366	-5.251349065	1.50989E-07	1.5533E-06	Yes	OK	74807.4746	89866.16979	59748.7794	89325.42032	90449.46839	89823.62066	69795.28988	46897.02968	62554.01865
ENSG00000140044	JDP2	chr14:75427716-75474111	+	protein_coding	-0.588316005	0.093835415	-6.269658464	3.61841E-10	5.06024E-09	Yes	OK	2945.275364	3535.121779	2355.428949	3501.598487	3491.50665	3612.260199	2134.899586	2639.967193	2291.420067
ENSG00000198736	MSRB1	chr16:1938210-1943326	-	protein_coding	-0.58828874	0.154276631	-3.813207067	0.000137175	0.000832253	Yes	OK	732.7431005	877.9475799	587.538621	855.9696362	875.4774868	902.3956167	476.877096	752.2402019	533.4985652
ENSG00000198453	ZNF568	chr19:36916329-36998700	+	protein_coding	-0.588003572	0.165445279	-3.554066793	0.000379323	0.002090276	Yes	OK	248.7561312	299.6366901	197.8755724	345.538663	286.4908046	266.8806028	191.4873281	177.3703213	224.7690676
ENSG00000003436	TFPI	chr2:187464230-187565760	-	protein_coding	-0.587981328	0.133929577	-4.390227628	1.13232E-05	8.5929E-05	Yes	OK	620.0671984	746.1510591	493.9833416	743.5908005	729.0310699	765.8312949	584.5887181	418.0871859	479.2741209
ENSG00000124588	NQO2	chr6:2987987-3028869	+	protein_coding	-0.587350375	0.100892689	-5.821535515	5.83094E-09	7.13586E-08	Yes	OK	806.5576439	968.4511338	644.6641539	927.3879617	951.501583	1026.463857	649.9521811	642.9674147	641.0728661
ENSG00000132000	PODNL1	chr19:13931187-13953392	-	protein_coding	-0.58662899	0.20153518	-2.910801929	0.003605025	0.015045311	Yes	OK	590.2136527	706.6155072	473.8117982	696.3286734	736.2333527	687.2844954	336.9440485	677.8080135	406.6833325
ENSG00000154134	ROBO3	chr11:124865386-124881470	+	protein_coding	-0.58559915	0.186725247	-3.136154104	0.001711792	0.007959351	Yes	OK	141.59171	169.4636952	113.7197248	158.5906933	163.2517434	186.5486488	119.6795801	123.5257595	97.95383492
ENSG00000178607	ERN1	chr17:64039142-64130819	-	protein_coding	-0.585418813	0.131658636	-4.446490021	8.72847E-06	6.78652E-05	Yes	OK	409.725752	492.5492145	326.9022895	542.9893275	455.3443236	479.3139923	350.7532309	307.2307351	322.7229026
ENSG00000172667	ZMAT3	chr3:179017223-179072279	-	protein_coding	-0.585177928	0.123110928	-4.753257381	2.00165E-06	1.73671E-05	Yes	OK	3253.825435	3907.425076	2600.225794	4033.03485	3785.999991	3903.240388	3020.528479	2061.929985	2718.218919
ENSG00000276600	RAB7B	chr1:205976740-206003461	-	protein_coding	-0.584913632	0.138448934	-4.224760818	2.39195E-05	0.000170883	Yes	OK	294.0010135	352.8084505	235.1935764	333.9856986	366.5161691	357.923484	220.0263049	232.7985467	252.7558776
ENSG00000107372	ZFAND5	chr9:72351425-72365235	-	protein_coding	-0.584669946	0.084154434	-6.947583393	3.71596E-12	6.41813E-11	Yes	OK	5279.607396	6337.615195	4221.599596	6378.286627	6490.057055	6144.501904	4515.602618	3792.874281	4356.321891
ENSG00000116251	RPL22	chr1:6181269-6209389	-	protein_coding	-0.58455866	0.123761756	-4.723257658	2.32096E-06	1.98767E-05	Yes	OK	1471.035495	1767.447679	1174.623311	1780.206789	1763.759032	1758.377216	1255.714979	942.2798319	1325.875123
ENSG00000070371	CLTCL1	chr22:19179473-19291716	-	protein_coding	-0.584488578	0.127346888	-4.589735845	4.43807E-06	3.63798E-05	Yes	OK	420.2229769	504.8065146	335.6394392	489.4255834	500.1585277	524.8354329	317.6111933	316.7327166	372.5744078
ENSG00000171812	COL8A2	chr1:36095236-36125220	-	protein_coding	-0.583215346	0.123932593	-4.705907714	2.52739E-06	2.14991E-05	Yes	OK	1851.657975	2218.40236	1484.91359	2159.354076	2251.913755	2243.939249	1252.03253	1791.123512	1411.584728
ENSG00000079156	OSBPL6	chr2:178194481-178402891	+	protein_coding	-0.582597112	0.111631125	-5.218948686	1.79942E-07	1.83737E-06	Yes	OK	582.6726629	699.7592678	465.5860579	702.6302903	690.618895	706.028618	498.9717877	430.7564946	467.0298915
ENSG00000197070	ARRDC1	chr9:137605654-137615360	+	protein_coding	-0.58215051	0.175742609	-3.312517735	0.000924603	0.004630045	Yes	OK	310.6186462	371.5598697	249.6774227	348.6894714	331.3050087	434.6851289	197.0110011	291.3940993	260.6271679
ENSG00000178105	DDX10	chr11:108665025-108940930	+	protein_coding	-0.581996433	0.19425996	-2.995967021	0.00273576	0.011934962	Yes	OK	526.2395241	632.7712618	419.7077863	674.2730141	639.4026618	584.6381097	498.0511756	266.0554819	495.0167015
ENSG00000203880	PCMTD2	chr20:64255695-64287821	+	protein_coding	-0.581995156	0.155415462	-3.744769983	0.000180559	0.001069066	Yes	OK	783.0873963	940.743837	625.4309556	938.9409261	944.2993002	938.9912846	776.0760462	468.7644206	631.4524001
ENSG00000213614	HEXA	chr15:72340919-72376476	-	protein_coding	-0.580705209	0.082433419	-7.044536285	1.8608E-12	3.35474E-11	Yes	OK	1644.730129	1972.17329	1317.286968	1947.199639	1949.417877	2019.902355	1327.522727	1274.849184	1349.488994
ENSG00000198796	ALPK2	chr18:58481247-58628957	-	protein_coding	-0.579910355	0.085921116	-6.749334495	1.48525E-11	2.40385E-10	Yes	OK	3990.661415	4784.735729	3196.587101	4973.026046	4748.705125	4632.476015	3343.663345	2880.684057	3365.4139
ENSG00000133107	TRPC4	chr13:37636636-37870425	-	protein_coding	-0.579839848	0.106465263	-5.446282048	5.14336E-08	5.64774E-07	Yes	OK	635.0452523	760.8699974	509.2205072	740.4399921	798.653137	743.5168632	498.0511756	508.3560101	521.2543358
ENSG00000042286	AIFM2	chr10:70098223-70132934	-	protein_coding	-0.579112314	0.104659948	-5.533275389	3.14305E-08	3.52897E-07	Yes	OK	626.4592803	751.0026718	501.9158889	742.5405311	756.2396938	754.2277904	517.3840308	484.6010564	503.7625796
ENSG00000171365	CLCN5	chrX:49922615-50099235	+	protein_coding	-0.579088932	0.14039536	-4.124701362	3.71216E-05	0.000256732	Yes	OK	472.5240651	567.3271553	377.7209748	581.8492987	619.3963206	500.7358467	406.9105723	323.0673709	403.1849812
ENSG00000168542	COL3A1	chr2:188974320-189012746	+	protein_coding	-0.579028472	0.048376251	-11.96927121	5.14788E-33	2.62335E-31	Yes	OK	92461.45354	110769.6345	74153.27255	110050.3882	111552.157	110706.3584	72530.42859	75459.98606	74469.403
ENSG00000133678	TMEM254	chr10:80078646-80092557	+	protein_coding	-0.578844149	0.13964658	-4.145064981	3.39717E-05	0.00023724	Yes	OK	320.6853718	385.292	256.0787435	378.0970172	376.1192128	401.65977	278.0248706	221.7129016	268.4984582
ENSG00000166986	MARS	chr12:57475445-57517569	+	protein_coding	-0.578355127	0.078710332	-7.347893415	2.01355E-13	3.91299E-12	Yes	OK	5244.264424	6279.178506	4209.350342	6140.925721	6578.885209	6117.724586	3970.600222	4519.775866	4137.674938
ENSG00000138463	DIRC2	chr3:122794795-122881139	+	protein_coding	-0.577594397	0.122696269	-4.707513947	2.50756E-06	2.13525E-05	Yes	OK	392.7508114	470.6502353	314.8513874	462.1185766	455.3443236	494.4878058	324.0554784	305.6470715	314.8516123
ENSG00000178700	DHFR2	chr3:94047836-94063389	-	protein_coding	-0.576771333	0.183733099	-3.139180353	0.001694211	0.007888747	Yes	OK	186.788792	224.8565137	148.7210702	236.3106358	232.0735568	206.1853486	167.5514121	112.4401144	166.1716842
ENSG00000164010	ERMAP	chr1:42817088-42844993	+	protein_coding	-0.575447978	0.115390987	-4.986940402	6.1343E-07	5.80624E-06	Yes	OK	495.7301394	593.5506387	397.9096402	565.0449869	604.1915014	611.4154277	417.9579181	384.8302507	390.9407519
ENSG00000125827	TMX4	chr20:7977348-8019829	-	protein_coding	-0.574945688	0.122593864	-4.689840672	2.73418E-06	2.31284E-05	Yes	OK	1868.061371	2237.80938	1498.313361	2181.409736	2294.327198	2237.691208	1671.831673	1197.249669	1625.858742
ENSG00000187840	EIF4EBP1	chr8:38030341-38060365	+	protein_coding	-0.574835917	0.132235939	-4.347047563	1.37982E-05	0.000103613	Yes	OK	2246.808884	2686.122825	1807.494943	2659.282354	2685.65123	2713.434891	1467.455774	2232.965652	1722.063401
ENSG00000198860	TSEN15	chr1:184051651-184123978	+	protein_coding	-0.57424781	0.094643228	-6.067500224	1.29916E-09	1.72176E-08	Yes	OK	1225.214038	1466.233617	984.1944599	1428.36651	1463.663915	1506.670426	1057.783365	958.1164677	936.6835464
ENSG00000175155	YPEL2	chr17:59331689-59401729	+	protein_coding	-0.573667781	0.12788518	-4.485803442	7.26397E-06	5.75108E-05	Yes	OK	400.699079	478.5850452	322.8131128	448.4650732	486.5542157	500.7358467	332.3409878	340.4876703	295.6106804
ENSG00000104914	MTMR11	chr1:149928651-149936869	-	protein_coding	-0.572766359	0.132126303	-4.334991199	1.45766E-05	0.00010901	Yes	OK	505.7429948	603.3660698	408.1199199	588.1509157	603.3912478	618.5560459	348.9120065	468.7644206	406.6833325
ENSG00000133639	BTG1	chr12:92140278-92145897	-	protein_coding	-0.572553034	0.076620532	-7.472579688	7.86377E-14	1.57659E-12	Yes	OK	2315.558613	2769.774528	1861.342698	2868.285983	2708.858586	2732.179014	1873.445734	1835.466093	1875.116269
ENSG00000080561	MID2	chrX:107825755-107927193	+	protein_coding	-0.572504159	0.170762763	-3.352628819	0.00080048	0.004080489	Yes	OK	237.4034869	282.99052	191.8164538	273.070068	300.0951166	275.8063754	208.0583469	216.9619109	150.4291036
ENSG00000105088	OLFM2	chr19:9853718-9936552	-	protein_coding	-0.572325376	0.172543808	-3.316985887	0.000909942	0.004564964	Yes	OK	440.4456011	525.2878435	355.6033586	466.3196546	587.3861749	522.1577011	266.9775248	430.7564946	369.0760566
ENSG00000119655	NPC2	chr14:74476192-74494177	-	protein_coding	-0.571565419	0.084230015	-6.785768953	1.1547E-11	1.89118E-10	Yes	OK	2290.452549	2740.266644	1840.638644	2798.968197	2666.445143	2755.386023	1940.650422	1708.773006	1872.492505
ENSG00000151693	ASAP2	chr2:9206765-9405683	+	protein_coding	-0.570015618	0.086856683	-6.562714527	5.28369E-11	8.06768E-10	Yes	OK	1259.495462	1505.331249	1013.659675	1520.790225	1472.466705	1522.736817	993.3405147	1010.377366	1037.261145
ENSG00000121753	ADGRB2	chr1:31727117-31764893	-	protein_coding	-0.569935419	0.206001076	-2.766662343	0.005663338	0.022084469	Yes	OK	131.588393	156.6008996	106.5758865	152.2890764	156.8497143	160.663908	79.17264528	125.1094231	115.4455912
ENSG00000168003	SLC3A2	chr11:62856102-62888875	+	protein_coding	-0.567517297	0.108127536	-5.248591781	1.53266E-07	1.57574E-06	Yes	OK	4795.786101	5724.396605	3867.175597	5489.758636	5837.050081	5846.381097	3288.426616	4500.771903	3812.328272
ENSG00000253276	CCDC71L	chr7:106656765-106660996	-	protein_coding	-0.566743754	0.077299874	-7.331755227	2.27158E-13	4.40402E-12	Yes	OK	2405.735224	2873.381126	1938.089322	2855.68275	2860.906778	2903.553849	2021.664291	1862.388374	1930.215301

ENSG00000151572	ANO4	chr12:100717526-101128641	+	protein_coding	-0.566364312	0.225062555	-2.516475086	0.011853528	0.040963483	Yes	OK	108.490923	129.3414	87.6404459	135.4847645	108.8344956	143.70494	101.267337	93.43615139	68.21784932
ENSG00000096654	ZNF184	chr6:27450743-27473118	-	protein_coding	-0.565096028	0.20337736	-2.77855917	0.005460057	0.021433946	Yes	OK	201.8806005	242.2837312	161.4774698	233.1598273	254.4806589	239.2107075	185.043043	106.1054601	193.2839064
ENSG00000130158	DOCK6	chr19:11199295-11262481	-	protein_coding	-0.5648011	0.126149245	-4.477245172	7.56124E-06	5.95778E-05	Yes	OK	438.1345983	522.3316681	353.9375286	525.1347461	477.7514256	564.1088326	345.2295579	373.7446056	342.8384222
ENSG00000184517	ZFP1	chr16:75148492-75172236	+	protein_coding	-0.564314331	0.173972967	-3.243689766	0.001179922	0.005752818	Yes	OK	267.0647938	320.1165532	214.0130343	321.3824646	333.7057697	305.2614252	242.1209966	156.7826947	243.1354117
ENSG00000144445	KANSL1L	chr2:210021423-210171383	-	protein_coding	-0.564218762	0.155491799	-3.628607835	0.000284954	0.001613463	Yes	OK	259.005179	310.2108182	207.7995398	325.5835426	310.498414	294.550498	234.7560994	172.6193305	216.0231895
ENSG00000130856	ZNF236	chr18:76822607-76970727	+	protein_coding	-0.563578309	0.13713709	-4.109597984	3.96349E-05	0.000272397	Yes	OK	426.9796931	510.3266357	343.6327505	464.2191156	524.9663906	541.7944009	344.3089458	300.8960808	385.693225
ENSG00000090006	LTBP4	chr19:40592883-40629818	+	protein_coding	-0.563475108	0.184664964	-3.051337384	0.002278244	0.010173603	Yes	OK	990.6342612	1179.501953	801.7665693	1166.849406	1144.362711	1227.293742	604.8421855	1124.401144	676.0563785
ENSG00000156876	SASS6	chr1:100083563-100132955	-	protein_coding	-0.562944336	0.160313623	-3.511519018	0.000445554	0.002418006	Yes	OK	299.3359251	358.0481125	240.6237377	348.6894714	334.5060233	390.9488428	287.2309922	201.125275	233.5149458
ENSG00000144642	RBMS3	chr3:28574791-30010391	+	protein_coding	-0.562587099	0.153415927	-3.667071024	0.000245345	0.001410047	Yes	OK	1031.187051	1231.879301	830.4948021	1262.42393	1175.572603	1257.641369	980.4519445	604.9594887	906.072973
ENSG00000143819	EPHX1	chr1:225810092-225845563	+	protein_coding	-0.561575533	0.118852295	-4.724986876	2.3013E-06	1.97186E-05	Yes	OK	1329.355962	1582.457378	1076.254546	1541.795615	1590.904245	1614.672276	946.3892948	1274.849184	1007.525159
ENSG00000186493	C5orf38	chr5:2752131-2755397	+	protein_coding	-0.561571561	0.212957094	-2.637017394	0.008363854	0.030764032	Yes	OK	191.1389829	226.3471769	155.9307889	213.2047069	256.0811662	209.7556577	129.8063138	213.7945837	124.1914693
ENSG00000161281	COX7A1	chr19:36150922-36152869	-	protein_coding	-0.561421244	0.158639772	-3.538969053	0.000401693	0.002196633	Yes	OK	652.3772361	775.6440775	529.1103947	791.9031972	775.4457813	759.583254	435.4495491	685.7263314	466.1553037
ENSG00000147905	ZCCHC7	chr9:37120539-37358149	+	protein_coding	-0.56083783	0.190093719	-2.950322782	0.003174421	0.013528238	Yes	OK	450.9305276	539.0976889	362.7633663	509.3807038	570.5808483	537.3315146	450.1793435	237.5495374	400.5612178
ENSG00000146143	PRIM2	chr6:57314805-57646849	+	protein_coding	-0.559931067	0.131312869	-4.264098939	2.00711E-05	0.000145666	Yes	OK	490.8271637	586.5362618	395.1180656	601.8044191	571.381102	586.4232643	406.9105723	334.153016	444.2906084
ENSG00000104763	ASAH1	chr8:18055992-18084998	-	protein_coding	-0.559500119	0.117786647	-4.750114981	2.03301E-06	1.75753E-05	Yes	OK	3097.564974	3693.365887	2501.764061	3748.411818	3759.591621	3572.094222	2838.247272	2014.420078	2652.624833
ENSG00000083290	ULK2	chr17:19770829-19867936	-	protein_coding	-0.558743953	0.10312843	-5.417942997	6.02886E-08	6.54585E-07	Yes	OK	902.4617409	1074.689643	730.2338391	1123.788357	1036.328469	1063.952102	765.0287004	752.2402019	673.4326151
ENSG00000206418	RAB12	chr18:8609445-8639381	+	protein_coding	-0.557530088	0.105863885	-5.266480531	1.39064E-07	1.44325E-06	Yes	OK	1073.106953	1278.892201	867.3217043	1214.111533	1282.806592	1339.758477	959.277865	801.333773	841.353475
ENSG00000168300	PCMTD1	chr8:51817575-51899186	-	protein_coding	-0.555295914	0.167490724	-3.315383094	0.000915176	0.004589823	Yes	OK	746.1875123	889.8044702	602.5705545	887.477721	863.4736821	918.4620075	765.9493125	433.9238217	607.8385292
ENSG00000164039	BDH2	chr4:103079435-103099883	-	protein_coding	-0.554445189	0.150400543	-3.686457363	0.000227398	0.001318874	Yes	OK	220.3537552	261.7776787	178.9298316	259.4165646	253.6804052	272.2360664	165.7101878	190.03963	181.039677
ENSG00000134744	TUT4	chr1:52408282-52553487	-	protein_coding	-0.553820581	0.148093484	-3.739668806	0.000184263	0.001089037	Yes	OK	944.588239	1125.753122	763.4233557	1124.838626	1164.369052	1088.051688	885.6288926	568.5352263	836.1059481
ENSG00000126775	ATG14	chr14:55366392-55411858	-	protein_coding	-0.550761537	0.137133213	-4.016251971	5.9131E-05	0.000389163	Yes	OK	555.5162507	662.0972629	448.9352386	652.2173547	668.2117929	665.862641	487.9244419	359.4916333	499.3896405
ENSG00000158623	COPG2	chr7:130506238-130668748	-	protein_coding	-0.550711063	0.119252231	-4.618035714	3.8739E-06	3.20908E-05	Yes	OK	821.1201794	975.2389846	667.0013741	1042.917606	1025.925172	856.8741761	614.048307	712.6486123	674.3072029
ENSG00000177119	ANO6	chr12:45215987-45440404	+	protein_coding	-0.550645172	0.094072336	-5.853422989	4.81557E-09	5.95525E-08	Yes	OK	4647.112452	5526.173747	3768.051156	5603.187741	5375.303728	5600.029772	4109.612658	3270.265299	3924.275512
ENSG00000150593	PDCD4	chr10:110871795-110900006	+	protein_coding	-0.550359585	0.121799381	-4.518574549	6.22574E-06	5.00125E-05	Yes	OK	1142.154614	1358.967548	925.341681	1366.400609	1464.464169	1246.037864	1051.33908	794.9991186	929.686844
ENSG00000100711	ZFYVE21	chr14:103715730-103733668	+	protein_coding	-0.549862786	0.099024765	-5.552780517	2.81161E-08	3.17415E-07	Yes	OK	968.2534892	1151.112783	785.3941953	1080.727308	1174.77235	1197.838692	786.2027799	752.2402019	817.739604
ENSG00000104324	CPQ	chr8:96645227-97149654	+	protein_coding	-0.549653929	0.09003167	-6.105117536	1.02725E-09	1.38255E-08	Yes	OK	1432.895512	1704.087141	1161.703883	1688.833344	1667.728595	1755.699484	1194.954576	1083.225891	1206.93118
ENSG00000111801	BTN3A3	chr6:26440472-26453415	+	protein_coding	-0.549234726	0.140264438	-3.915709033	9.01389E-05	0.000569772	Yes	OK	301.4049154	359.0581772	243.7516535	386.4991732	349.7108425	340.9645159	252.2477303	220.129238	258.8779923
ENSG00000138829	FBN2	chr5:128257909-128659185	-	protein_coding	-0.549153809	0.061173154	-8.977039359	2.7815E-19	7.73371E-18	Yes	OK	19072.43445	22657.74698	15487.12192	23260.31845	22860.84586	21852.07665	15414.72991	15961.74525	15084.89058
ENSG00000163644	PPM1K	chr4:88257620-88284769	-	protein_coding	-0.548885253	0.149017692	-3.683356296	0.000230183	0.001331103	Yes	OK	266.6099943	317.5912283	215.6287603	320.3321952	289.6918192	342.7496704	222.7881414	193.2069571	230.8911823
ENSG00000187240	DYNC2H1	chr11:103109431-103479863	+	protein_coding	-0.548388241	0.133769636	-4.099497142	4.14049E-05	0.000282908	Yes	OK	1206.477084	1435.304528	977.6496403	1456.723786	1446.058335	1403.131463	1108.417034	760.1585198	1064.373367
ENSG00000013441	CLK1	chr2:200853009-200864744	-	protein_coding	-0.547958906	0.177836075	-3.081258441	0.002061277	0.009336437	Yes	OK	866.8098675	1031.276937	702.3427983	1094.380811	1028.325933	971.1240663	907.7235843	483.0173928	716.2874179
ENSG00000188878	FBF1	chr17:75909574-75941140	-	protein_coding	-0.547931792	0.216595029	-2.529752382	0.011414305	0.039771266	Yes	OK	96.90366171	115.3707167	78.43660674	120.7809916	104.8332274	120.497931	69.04591158	74.4321884	91.83172024
ENSG00000185875	THNSL1	chr10:25016658-25026664	+	protein_coding	-0.547727509	0.221760408	-2.469906668	0.013514831	0.045398799	Yes	OK	98.02876257	116.4701845	79.58734061	105.0269492	140.8446414	103.5389629	85.61693037	74.4321884	78.71290306
ENSG00000134086	VHL	chr3:10141008-10152220	+	protein_coding	-0.547725534	0.156626011	-3.497027932	0.000470473	0.002534854	Yes	OK	870.6290332	1035.909415	705.3486515	1064.973265	1001.117309	1041.63767	863.5342009	517.8579916	734.6537619
ENSG00000108821	COL1A1	chr17:50183289-50201632	-	protein_coding	-0.547338869	0.182405238	-3.000675164	0.002693818	0.011780085	Yes	OK	783290.4881	930117.1664	636463.8099	915184.8805	925426.9185	949739.7001	524014.2795	791662.3395	593714.8106
ENSG00000102096	PIM2	chrX:48913182-48919024	-	protein_coding	-0.546370711	0.202966097	-2.691930907	0.007103966	0.026831409	Yes	OK	150.5662932	178.2213291	122.9112573	163.8420408	151.2479388	219.5740076	109.5528464	139.3623953	119.8185302
ENSG00000154589	LY96	chr8:73991352-74029087	+	protein_coding	-0.545874131	0.177410852	-3.076892563	0.002091707	0.009442298	Yes	OK	162.6140588	193.0989756	132.129142	181.6966222	220.8700058	176.7302988	128.8857016	126.6930866	140.8086377
ENSG00000157184	CPT2	chr1:53196429-53214197	+	protein_coding	-0.545783608	0.176851886	-3.086105672	0.002027967	0.009193154	Yes	OK	240.0838804	285.2286416	194.9391193	248.9138697	280.0887755	326.6832796	168.4720243	183.7049756	232.6403579
ENSG00000164904	ALDH7A1	chr5:126531200-126595418	-	protein_coding	-0.545706877	0.113934106	-4.789670932	1.67055E-06	1.47202E-05	Yes	OK	1239.909769	1473.894034	1005.925505	1476.678906	1435.655038	1509.348158	1077.116221	845.6763533	1094.98394
ENSG00000237765	FAM200B	chr4:15681662-15705565	+	protein_coding	-0.545420789	0.18351347	-2.972102198	0.002957682	0.012741023	Yes	OK	399.9779423	476.1779912	323.7778933	434.8115698	461.7463527	531.976051	396.7838386	226.4638924	348.0859491
ENSG00000046651	OFD1	chrX:13734745-13769353	+	protein_coding	-0.5453119	0.203479579	-2.679934284	0.007363661	0.027659943	Yes	OK	248.315822	295.9317157	200.6999284	276.2208765	331.3050087	280.2692618	254.0889546	134.6114046	213.3994261
ENSG00000110436	SLC1A2	chr11:35251205-35420063	-	protein_coding	-0.545125159	0.196341534	-2.776412859	0.005496237	0.021550275	Yes	OK	122.7213614	146.4669383	98.97578449	154.3896154	136.8433732	148.1678263	107.7116221	80.76684273	108.4488887
ENSG00000122778	KIAA1549	chr7:138831381-138981318	-	protein_coding	-0.544668601	0.105296128	-5.172731524	2.30696E-07	2.31823E-06	Yes	OK	802.9435222	951.2647288	654.6223156	959.946316	961.1046267	932.7432438	615.8895313	717.3996031	630.5778123
ENSG00000123636	BAZ2B	chr2:159318979-159616692	-	protein_coding	-0.544568427	0.128337873	-4.243240258	2.20315E-05	0.000158358	Yes	OK	610.1812244	725.0523355	495.3101133	747.7918785	733.8325918	693.5325363	577.2238208	443.4258032	465.2807159
ENSG00000267680	ZNF224	chr19:44094339-44109886	+	protein_coding	-0.544421437	0.203318551	-2.677677139	0.007413464	0.027827963	Yes	OK	145.7093618	173.8892955	117.529428	170.1436578	179.2568163	172.2674125	146.3773326	85.51783348	120.693118
ENSG00000215375	MYL5	chr4:673580-682033	+	protein_coding	-0.544331652	0.174123889	-3.126117004	0.001771311	0.008192125	Yes	OK	200.4364389	237.0842757	163.7886022	260.4668341	247.2783761	203.5076168	160.1865149	186.8723028	144.3069889
ENSG00000119878	CRIPT	chr2:46616416-46625742	+	protein_coding	-0.544113589	0.155309573	-3.50341307	0.000459337	0.002482167	Yes	OK	381.0148852	453.5947859	308.4349845	475.77208	436.9384898	448.0737879	366.4036375	242.3005282	316.6007879
ENSG00000109475	RPL34	chr4:108620566-108630412	+	protein_coding	-0.542592006	0.166714975	-3.254608695	0.001135487	0.005572485	Yes	OK	1334.867856	1585.089847	1084.645866	1563.851274	1582.101455	1609.316812	1202.319474	737.9872297	1313.630893
ENSG00000104998	IL27RA	chr19:14031748-14053216	+	protein_coding	-0.542582803	0.221294683	-2.451856482	0.014212133	0.047268482	Yes	OK	102.9316501	122.6150057	83.24829444	138.635573	117.6372857	111.5721583	74.56958451	72.84852482	102.326774
ENSG00000134419	RPS15A	chr16:18781295-18790383	-	protein_coding	-0.541738209	0.18867863	-2.871221872	0.004088884	0.016737885	Yes	OK	959.7202633	1139.67966	779.7608667	1085.978655	1153.965755	1179.094569	948.2305191	489.3520471	901.700034
ENSG00000105281	SLC1A5	chr19:46774883-46788594	-	protein_coding	-0.541069232	0.107122666	-5.050931341	4.39661E-07	4.24447E-06	Yes	OK	8620.206723	10214.85265	7025.560792	10196.01623	10076.79389	10371.74784	6181.910617	8396.584317	6498.187442
ENSG00000172992	DCAKD	chr17:45023340-45061109	-	protein_coding	-0.540749395	0.141946074	-3.809541041	0.000139225	0.000844068	Yes	OK	590.6312821	698.3776579	482.8848964	730.9875666	693.8199096	670.3255273	399.545675	581.2045349	467.9044793
ENSG00000197892	KIF13B	chr8:29067279-29263124	-	protein_coding	-0.54067717	0.216953989	-2.492128279	0.012698017	0.043165914	Yes	OK	124.327641	146.8918355	101.7634464	140.736112	177.656309	122.2830855	110.4734585	110.8564508	83.96042993
ENSG00000130517	PGPEP1	chr19:18340587-18369950	+	protein_coding	-0.540371326	0.120629022	-4.47961292	7.47785E-06	5.9053E-05	Yes	OK	667.613253	789.7107884	545.5157176	809.7577786	757.8402011	801.5343856	539.4787225	616.0451338	481.0232965
ENSG00000166900	STX3	chr11:59713456-59805882	+	protein_coding	-0.538896839	0.129475715	-4.162146074	3.15271E-05	0.000221483	Yes	OK	570.2800286	676.9480581	463.6119942	733.0881056	689.0183877	608.7376959	474.1152595	408.5852044	508.1355187
ENSG00000171862	PTEN	chr10:87863113-87971930	+	protein_coding	-0.538593072	0.105253821	-5.117088068	3.10289E-07	3.05099E-06	Yes	OK	1674.640305	1985.969999	1363.310612	2090.03629	1915.807224	1952.066482	1483.106181	1182.996696	1423.828958
ENSG00000198839	ZNF277	chr7:112206588-112343096	+	protein_coding	-0.537839658	0.153901677	-3.494696542	0.000474601	0.002554126	Yes	OK	272.5493401	322.7791267	222.3195535	317.1813867	367.3164227	283.8395708	236.5973237	216.9619109	213.3994261
ENSG00000172986	GXYLT2	chr3:72888073-72998138	+	protein_coding	-0.537811652	0.184152567	-2.920467855	0.003495063	0.014651307	Yes	OK	139.8516704	165.3544913	114.3488495	171.1939272	147.2466705	177.6228761	104.9497856	123.5257595	114.5710033
ENSG00000162729	IGSF8	chr1:160091340-160098943	-	protein_coding	-0.537261669	0.144120507	-3.727864138	0.000193109	0.001136003	Yes	OK	519.0119869	612.7512139	425.2727598	569.2460648	597.7894723	671.2181046	382.9746563	511.5233373	381.3202859
ENSG00000060656	PTPRU	chr1:29236516-29326813	+	protein_coding	-0.537220851	0.178132264	-3.015853719	0.002562569	0.011278042	Yes	OK	539.4003832	636.7291736	442.0715927	655.3681632	643.40393	611.4154277	345.2295579	597.0411708	383.9440494
ENSG00000178163	ZNF518B	chr4:10439874-10457410	-	protein_coding	-0.536883457	0.130727091	-4.106902796	4.01E-05	0.000275134	Yes	OK	721.3202003	855.8375669	586.8028338	906.3825719	873.8769795	787.2531493	646.2697324	486.18472	627.9540489
ENSG00000107854	TNKS2	chr10:91798312-91865276	+	protein_coding	-0.536562274	0.0883878	-6.070546785	1.27475E-09	1.69077E-08	Yes	OK	3325.244518	3938.603979	2711.885058	4211.580664	3865.225102	3739.006171	2898.087062	2505.355788	2732.212324
ENSG00000180537	RNF182	chr6:13924446-13980302	+	protein_coding	-0.53631314	0.167425849	-3.203287569	0.001358682	0.006493586	Yes	OK	284.2219398	337.9971889	230.4466907	345.538663	325.7032332	342.7496704	264.2156883	172.6193305	254.5050532
ENSG00000159267	HLCS	chr21:36750888-36990236	-	protein_coding	-0.536062202	0.117099765	-4.577824748	4.69836E-06	3.83454E-05	Yes	OK	1028.988174	1219.829793	838.146555	1181.553179	1244.394417	1233.541783	940.8656219	709.4812852	864.0927581
ENSG00000197965	MPZL1	chr1:167721192-167791919	+	protein_coding	-0.535470543	0.082220222	-6.512638012	7.38423E-11	1.11509E-09	Yes	OK	6534.481839	7736.251228	5332.712449	7946.338979	7556.795163	7705.619544	5727.128213	4804.835311	5466.173824
ENSG00000136542	GALNT5	chr2:157257598-157314211	+	protein_coding	-0.535450679	0.106274882	-5.038355892	4.69548E-07	4.52239E-06	Yes	OK	6486.170008	7679.070265	5293.269751	7830.809335	7802.473031	7403.928428	5892.838401	4367.744162	5619.226691
ENSG00000170035	UBE2E3	chr2:180967248-181076585	+	protein_coding	-0.535218811	0.089139063	-6.004312738	1.92144E-09	2.50808E-08	Yes	OK	1435.961937	1698.678776	1173.245097	1781.257059	1626.915659	1687.863611	1160.891927	1221.004622	1137.838743
ENSG00000143669	LYST	chr1:235661041-235883640	-	protein_coding	-0.534913219	0.169787525	-3.150485992	0.001629991	0.007628561	Yes	OK	812.9507087	963.8733551	662.0280622	985.1527838	975.5091923	930.9580892	814.7417567	457.6787755	713.6636544
ENSG00000168528	SERINC2	chr1:31409565-31434680	+	protein_coding	-0.534869362	0.125256641	-4.270187652	1.95309E-05	0.000142121	Yes	OK	1119.185196	1322.025471	916.3449219	1249.820696	1337.22384	1379.031877	798.1707379	1091.144209	859.719819
ENSG00000132688	NES	chr1:156668763-156677397	-	protein_coding	-0.534808918	0.105718061	-5.058822631	4.21853E-07	4.09416E-06	Yes	OK	1072.575084	1268.251373	876.898796	1340.143872	1321.218767	1143.391479	843.2807335	931.1941868	856.2214678
ENSG00000178764	ZHX2	chr8:122781394-122974512	+	protein_coding	-0.53452023	0.125649746	-4.254049436	2.09939E-05	0.000151561	Yes	OK	570.8082904	674.7754172	466.8411637	743.5908005	681.8161049	598.919346	440.0526098	500.4376922	460.033189
ENSG00000139631	CSAD	chr12:53157663-53180909	-	protein_coding	-0.534421215	0.191101192	-2.796535229	0.005165378	0.020472459	Yes	OK	210.1856583	247.5475476	172.823769	199.5512035	290.4920729	252.5993665	147.2979447	205.8762658	165.2970964
ENSG00000092036	HAUS4	chr14:22946228-22957161	-	protein_coding	-0.534405509	0.191442387	-2.791469105	0.005246937	0.020740773	Yes	OK	167.6570559	197.1590924	138.1550194	179.5960832	197.6626501	214.218544	114.1559072	172.6193305	127.6898205
ENSG00000163430	FSTL1	chr3:120392293-120451253	-	protein_coding	-0.533954502	0.054844919	-9.735714958	2.12319E-22	6.94787E-21	Yes	OK	75649.37153	89493.61549	61805.12758	89846.35399	89821.26928	88813.2232	64252.2841	59135.58185	62027.51679
ENSG00000095739	BAMBI	chr10:28677342-28682939	+	protein_coding	-0.533303194	0.135880589	-3.924793075	8.68043E-05	0.000551022	Yes	OK	283.0091588	334.8235831	231.1947345	325.5835426	351.3113498	327.5758569	229.2324265	226.4638924	237.8878848
ENSG00000130779	CLIP1	chr12:122271432-122422632	-	protein_coding	-0.533251182	0.100942993	-5.282696361	1.27296E-07	1.3295E-06	Yes	OK	5505.75004	6514.311474	4497.188605	6588.340525	6478.05325	6476.540648	5159.110514	3872.05746	4460.39784
ENSG00000142875	PRKACB	chr1:84078062-84238498	+	protein_coding	-0.533070583	0.152618544	-3.492829706	0.000477931	0.002566685	Yes	OK	1324.258662	1567.943411	1080.573912	1553.348579	1503.676598	1646.805057	1370.791498	826.6723903	1044.257847
ENSG00000135148	TRAFD1	chr12:112125501-112153609	+	protein_coding	-0.532141829	0.100687882	-5.285063305	1.25661E-07	1.31326E-06	Yes	OK	795.3907506	941.4848995	649.2966017	959.443621	938.6975247	936.3135528	651.7934054	611.294143	684.8022566
ENSG00000215114	UBXN2B	chr8:58411264-58451501	+	protein_coding	-0.531936302	0.148712857	-3.576935519	0.000347646	0.001930584	Yes	OK	404.1214119	479.2891531	328.9536707	513.5817817	436.9384898	487.3471877	380.2128198	270.8064727	335.8417197
ENSG00000107679	PLEKHA1	chr10:122374696-122442602	+	protein_coding	-0.531386638	0.152431939	-3.486058389	0.000490194	0.002629063	Yes	OK	875.681458	1037.074474	714.2884421	1035.565719	1033.127455	1042.530248	869.978486	532.1109639	740.7758766

ENSG00000243943	ZNF512	chr2:27582969-27623215	+	protein_coding	-0.531204493	0.148034782	-3.588376235	0.000332744	0.001857846	Yes	OK	462.0270117	547.8133182	376.2407051	573.4471428	543.3722244	526.6205874	440.0526098	297.7287536	390.9407519
ENSG00000184384	MAML2	chr11:95976598-96343180	-	protein_coding	-0.531183539	0.083704809	-6.345914262	2.21108E-10	3.16473E-09	Yes	OK	1476.339055	1745.512642	1207.165469	1771.804634	1696.537726	1768.195565	1229.937838	1192.498678	1199.05989
ENSG00000197746	PSAP	chr10:71816298-71851375	-	protein_coding	-0.530737643	0.078068176	-6.79838665	1.05797E-11	1.73448E-10	Yes	OK	24891.38922	29415.62391	20367.15453	29033.64985	29867.06651	29346.15538	18747.34591	22548.20209	19805.91559
ENSG00000080573	COL5A3	chr19:9959561-10010471	-	protein_coding	-0.530640422	0.122349735	-4.337078625	1.44389E-05	0.000108078	Yes	OK	2148.982902	2537.354302	1760.611501	2408.267946	2591.2213	2612.57366	1475.741284	2101.521575	1704.571645
ENSG00000197603	CPLANE1	chr5:37106228-37249428	-	protein_coding	-0.530274239	0.164526994	-3.22302272	0.001268455	0.006128197	Yes	OK	1066.938384	1262.88692	870.9898481	1332.791986	1296.410904	1159.45787	1023.720716	609.7104794	979.5383492
ENSG00000178685	PARP10	chr8:143977153-144012772	-	protein_coding	-0.529471263	0.182225557	-2.905581806	0.00366571	0.015263746	Yes	OK	590.0165739	695.1227459	484.910402	669.0216666	744.2358892	672.1106819	361.8005767	655.6367233	437.2939059
ENSG00000122335	SERAC1	chr6:158109515-158168280	-	protein_coding	-0.529209163	0.144592506	-3.660004081	0.000252211	0.001443964	Yes	OK	654.6332784	775.178193	534.0883637	745.6913395	820.2599854	759.583254	584.5887181	413.3361952	604.340178
ENSG00000138172	CALHM2	chr10:103446786-103452402	-	protein_coding	-0.52909632	0.101023965	-5.237334715	1.62912E-07	1.67282E-06	Yes	OK	1849.971506	2183.090563	1516.852449	2118.393566	2157.483825	2273.394298	1400.251087	1715.10766	1435.198599
ENSG00000135835	KIAA1614	chr1:180913154-180951614	+	protein_coding	-0.528488948	0.183973662	-2.872633742	0.004070657	0.016682574	Yes	OK	297.7261018	350.2632531	245.1889505	384.3986342	351.3113498	315.0797752	190.566716	316.7327166	228.2674189
ENSG00000115459	ELMOD3	chr2:85354394-85391752	+	protein_coding	-0.528414552	0.186983733	-2.8259921	0.004713443	0.018963367	Yes	OK	355.2479703	417.9696425	292.5262981	344.4883935	496.1572594	413.2632745	243.0416088	372.160942	262.3763435
ENSG00000164300	SERINC5	chr5:80111651-80256079	-	protein_coding	-0.527870934	0.104386435	-5.056892079	4.26144E-07	4.13337E-06	Yes	OK	796.5780691	940.3770118	652.7791265	941.0414651	941.0982856	938.9912846	598.3979004	669.8896956	690.0497835
ENSG00000147889	CDKN2A	chr9:21967753-21995301	-	protein_coding	-0.527866826	0.164373731	-3.211381904	0.001320983	0.006337328	Yes	OK	326.60379	384.1035812	269.1039988	353.9408189	383.3214956	415.0484291	255.9301789	330.9856888	220.3961286
ENSG00000136156	ITM2B	chr13:48233158-48270357	+	protein_coding	-0.527847603	0.099355627	-5.312709721	1.08007E-07	1.13452E-06	Yes	OK	7195.761646	8500.445899	5891.077393	8653.170347	8372.253626	8475.913725	6662.470162	5015.462567	5995.29945
ENSG00000168283	BMI1	chr10:22321211-22331484	+	protein_coding	-0.527778562	0.19866097	-2.656679684	0.007891438	0.02928818	Yes	OK	673.7028267	797.3233599	550.0822934	809.7577786	761.0412157	821.1710854	709.7919711	340.4876703	599.9672389
ENSG00000145423	SFRP2	chr4:153780592-153789120	-	protein_coding	-0.527347109	0.129999742	-4.05652427	4.98084E-05	0.000334768	Yes	OK	340.6203666	401.3084159	279.9323172	387.5494427	412.9308804	403.4449246	268.8187491	302.4797443	268.4984582
ENSG00000184304	PRKD1	chr14:29576479-30191898	-	protein_coding	-0.527024282	0.088336824	-5.966076838	2.43026E-09	3.14673E-08	Yes	OK	2170.26812	2563.956091	1776.580149	2662.433163	2624.831953	2404.603157	1821.891454	1658.095771	1849.753222
ENSG00000185361	TNFAIP8L1	chr19:4639518-4655568	+	protein_coding	-0.526838292	0.158090341	-3.332514112	0.000860651	0.004353508	Yes	OK	246.7763076	290.5963489	202.9562663	284.6230324	272.086239	315.0797752	210.8201834	224.8802288	173.1683867
ENSG00000108771	DHX58	chr17:42101404-42112733	-	protein_coding	-0.526249684	0.178583068	-2.946806154	0.003210744	0.013663094	Yes	OK	195.6426669	229.7117187	161.5736151	238.4111748	216.8687376	233.8552439	144.5361083	201.125275	139.0594621
ENSG00000146281	PM20D2	chr6:89146050-89165565	+	protein_coding	-0.525971347	0.181563996	-2.896892337	0.003768791	0.015619062	Yes	OK	152.6991389	180.7359805	124.6622974	181.6966222	163.2517434	197.259576	140.8536596	112.4401144	120.693118
ENSG00000104774	MAN2B1	chr19:12646511-12666742	-	protein_coding	-0.525937947	0.133959056	-3.926109699	8.63308E-05	0.000548651	Yes	OK	1304.719173	1537.471788	1071.966558	1458.824325	1554.092577	1599.498462	931.6595003	1323.942755	960.2974174
ENSG00000155657	TTN	chr2:178525989-178830802	-	protein_coding	-0.525890751	0.171137557	-3.072912573	0.002119806	0.009551602	Yes	OK	154.6932588	183.0239785	126.3625392	181.6966222	188.85986	178.5154534	127.0444773	115.6074416	136.4356986
ENSG00000117984	CTSD	chr11:1752752-1764573	-	protein_coding	-0.525826373	0.129345508	-4.065285162	4.79738E-05	0.000323097	Yes	OK	4940.931486	5828.705753	4053.157218	5514.965104	6058.720341	5912.431815	3324.33049	5045.552175	3789.588989
ENSG00000141127	PRPSAP2	chr17:18840085-18931287	+	protein_coding	-0.525607934	0.130884233	-4.015823158	5.92387E-05	0.000389298	Yes	OK	367.9878996	434.6245664	301.3512329	415.9067189	434.5377288	453.4292255	331.4203756	286.6431085	285.9902145
ENSG00000164619	BMPER	chr7:33904911-34155872	+	protein_coding	-0.525228975	0.094241754	-5.573208829	2.50089E-08	2.85272E-07	Yes	OK	1444.80614	1706.712657	1182.899624	1764.452747	1713.343052	1642.342171	1210.604983	1084.809554	1253.284334
ENSG00000075340	ADD2	chr2:70607618-70768225	-	protein_coding	-0.52503952	0.094126676	-5.578009781	2.43286E-08	2.77896E-07	Yes	OK	967.7711657	1142.158596	793.3837359	1167.899675	1141.96195	1116.614161	767.7905368	790.2481279	822.1125431
ENSG00000139173	TMEM117	chr12:43835967-44389762	+	protein_coding	-0.524718041	0.151231527	-3.469633944	0.000521168	0.002782493	Yes	OK	323.0087071	382.4757807	263.5416336	373.8959393	366.5161691	407.0152336	275.2630342	215.3782473	299.9836195
ENSG00000145882	PCYOX1L	chr5:149358007-149369653	+	protein_coding	-0.524311291	0.15473965	-3.388344822	0.000703158	0.003628207	Yes	OK	192.8734272	227.6765484	158.0703061	235.2603663	219.2694985	228.4997803	156.5040663	156.7826947	160.9241574
ENSG00000131437	KIF3A	chr5:132692628-132737638	-	protein_coding	-0.5241496	0.213450448	-2.455603183	0.014064837	0.046863809	Yes	OK	508.016822	600.9206081	415.113036	653.2676242	573.7818629	575.7123371	553.2879048	248.6351825	443.4160206
ENSG00000166734	CASC4	chr15:44288729-44415758	+	protein_coding	-0.523925522	0.126321206	-4.147565852	3.36029E-05	0.000234863	Yes	OK	3075.601341	3630.386076	2520.816607	3616.077862	3507.511723	3767.568643	2976.339095	1993.832451	2592.278274
ENSG00000170581	STAT2	chr12:56341597-56360155	-	protein_coding	-0.523756743	0.074118678	-7.066460922	1.58935E-12	2.89388E-11	Yes	OK	4466.945787	5268.057471	3665.834104	5351.123063	5152.032962	5301.016387	3437.565785	3799.208936	3760.727591
ENSG00000134247	PTGFRN	chr1:116910057-116990358	+	protein_coding	-0.523516027	0.097881958	-5.34844255	8.87143E-08	9.42089E-07	Yes	OK	1610.760123	1898.513252	1323.006995	1822.217569	1961.421682	1911.900505	1185.748455	1411.044252	1372.228277
ENSG00000107249	GLIS3	chr9:3824127-4348392	-	protein_coding	-0.522036494	0.145599023	-3.585439555	0.000336511	0.001875075	Yes	OK	430.89185	507.2468098	354.5368901	573.4471428	460.9460991	487.3471877	360.8799645	397.4995593	305.2311463
ENSG00000187266	EPOR	chr19:11377205-11384342	-	protein_coding	-0.521542979	0.199607536	-2.612842131	0.008979277	0.032569171	Yes	OK	112.2690588	132.3683252	92.16979243	118.6804526	132.0418513	146.3826718	86.53754252	90.26882423	99.70301055
ENSG00000196141	SPATS2L	chr2:200305881-200482263	+	protein_coding	-0.521343794	0.070518233	-7.393035403	1.43514E-13	2.83583E-12	Yes	OK	3812.379123	4495.023405	3129.734841	4485.701002	4460.613813	4538.755402	3247.919681	3021.630116	3119.654725
ENSG00000196305	IARS	chr9:92210207-92293756	-	protein_coding	-0.520879833	0.074799501	-6.963680584	3.31496E-12	5.77399E-11	Yes	OK	9707.665393	11443.87368	7971.457102	11724.15834	11602.87759	11004.58512	8445.695905	7365.619324	8103.056076
ENSG00000108465	CDK5RAP3	chr17:47967810-47981774	+	protein_coding	-0.520733266	0.187549082	-2.776517272	0.005494472	0.021548487	Yes	OK	167.2686702	197.0899086	137.4474319	181.6966222	217.6689912	191.9041124	163.8689635	133.027741	115.4455912
ENSG00000115825	PRKD3	chr2:37250502-37324808	-	protein_coding	-0.520523245	0.117975094	-4.412145214	1.02351E-05	7.83948E-05	Yes	OK	2304.779235	2718.520226	1891.038245	2785.314694	2641.63728	2728.608705	2178.168357	1547.239321	1947.707057
ENSG00000196526	AFAP1	chr4:7758714-7939926	-	protein_coding	-0.520113019	0.074387605	-6.991931194	2.71128E-12	4.78837E-11	Yes	OK	4730.219182	5574.395515	3886.04285	5450.898665	5586.570691	5685.717189	3711.908207	3810.294581	4135.925762
ENSG00000137817	PARP6	chr15:72241181-72272999	-	protein_coding	-0.518671483	0.113410286	-4.573407763	4.79855E-06	3.90724E-05	Yes	OK	651.512375	767.8211341	535.2036159	787.7021192	749.037411	766.7238722	590.112391	517.8579916	497.6404649
ENSG00000171451	DSEL	chr18:67506582-67516980	-	protein_coding	-0.518671053	0.115578369	-4.487613547	7.20254E-06	5.71344E-05	Yes	OK	2502.028159	2949.304355	2054.751963	3036.329102	2955.336708	2856.247254	2382.544256	1707.189342	2074.52229
ENSG00000116717	GADD45A	chr1:67685061-67688338	+	protein_coding	-0.516116056	0.091454394	-5.643425461	1.667E-08	1.94463E-07	Yes	OK	1364.740405	1604.669945	1124.810866	1584.856664	1610.910586	1618.242585	1111.17887	1206.75165	1056.502077
ENSG00000118922	KLF12	chr13:73686089-73995056	-	protein_coding	-0.515442649	0.115148744	-4.476320225	7.59406E-06	5.97791E-05	Yes	OK	1033.207817	1217.74891	848.6667236	1225.664498	1224.388076	1203.194156	954.6748042	723.7342574	867.5911093
ENSG00000071967	CYBRD1	chr2:171522247-171558133	+	protein_coding	-0.515054777	0.10379569	-4.962198093	6.96999E-07	6.53337E-06	Yes	OK	13974.47846	16445.85847	11503.09845	16530.19154	16380.39184	16426.99202	12937.36261	9462.389908	12109.54284
ENSG00000088387	DOCK9	chr13:98793429-99086625	-	protein_coding	-0.514936263	0.101840803	-5.056286351	4.27499E-07	4.14407E-06	Yes	OK	804.2568843	947.2423449	661.2714236	993.5549397	931.4952419	916.676853	689.5385037	628.7144424	665.5613248
ENSG00000006756	ARSD	chrX:2903970-2929351	-	protein_coding	-0.514115535	0.127568498	-4.030113577	5.57499E-05	0.000369066	Yes	OK	412.0807577	484.6084609	339.5530546	474.7218105	526.5668979	452.5366743	322.2142541	340.4876703	355.9572394
ENSG00000174749	FAM241A	chr4:112145397-112195256	+	protein_coding	-0.513526583	0.198598891	-2.58574748	0.009716809	0.03487542	Yes	OK	379.4350471	447.6469627	311.2231314	461.0683071	417.7324023	464.1401787	421.6403667	213.7945837	298.2344438
ENSG00000184635	ZNF93	chr19:19900913-19963464	+	protein_coding	-0.513017909	0.132891315	-3.86043217	0.000113187	0.000700396	Yes	OK	303.4417112	357.2544902	249.6289321	345.538663	366.5161691	359.7086385	253.1683425	235.9658739	259.7525801
ENSG00000170145	SIK2	chr11:111602391-111730853	+	protein_coding	-0.512592136	0.084776462	-6.046396859	1.48121E-09	1.95672E-08	Yes	OK	1585.346224	1864.769512	1305.922936	1822.217569	1918.207985	1853.882983	1298.063138	1260.596212	1359.10946
ENSG00000196739	COL27A1	chr9:114155560-114312511	+	protein_coding	-0.511815746	0.136087928	-3.760919511	0.00016929	0.001006688	Yes	OK	488.7715621	573.751415	403.7917092	540.8887885	600.1902332	580.1752234	337.8646607	437.0911489	436.4193181
ENSG00000174718	KIAA1551	chr12:31959370-31993107	+	protein_coding	-0.511395669	0.169087637	-3.02444152	0.002490926	0.01099213	Yes	OK	462.968576	545.8093676	380.1277844	536.6877106	532.1686734	568.5717189	469.5121988	275.5574634	395.3136909
ENSG00000171791	BCL2	chr18:63123346-63320128	-	protein_coding	-0.511047335	0.204113197	-2.503744699	0.012288667	0.04209441	Yes	OK	190.8304651	225.2250189	156.4359113	201.6517425	216.0684839	257.9548301	199.7728375	115.6074416	153.9274549
ENSG00000138640	FAM13A	chr4:88725955-89111398	-	protein_coding	-0.510779629	0.159479891	-3.202783915	0.001361061	0.006501178	Yes	OK	419.1194598	494.1658904	344.0730292	488.3753139	450.5428017	543.5795555	382.0540441	264.4718184	385.693225
ENSG00000182372	CLN8	chr8:1755778-1801711	+	protein_coding	-0.510014286	0.115143106	-4.429394917	9.44978E-06	7.28589E-05	Yes	OK	521.3642824	612.7333568	429.995208	639.6141208	573.7818629	624.8040867	413.3548574	432.3401581	444.2906084
ENSG00000116584	ARHGEF2	chr1:155946851-156007070	-	protein_coding	-0.5087415	0.098424354	-5.168857911	2.35529E-07	2.36247E-06	Yes	OK	4691.529863	5507.198045	3875.861681	5384.731687	5592.172466	5544.689981	3577.498832	4481.76794	3568.318272
ENSG00000211584	SLC48A1	chr12:47753916-47782753	+	protein_coding	-0.508649848	0.119362599	-4.261383816	2.03165E-05	0.000147123	Yes	OK	433.2319923	508.6605372	357.8030475	477.872619	524.166137	523.9428556	351.673843	357.9079697	363.8285297
ENSG00000099875	MKNK2	chr19:2037465-2051244	-	protein_coding	-0.508381078	0.132318944	-3.842088378	0.000121992	0.000748411	Yes	OK	2244.342991	2633.171428	1855.514554	2606.76888	2624.831953	2667.91345	1543.866583	2313.732495	1708.944584
ENSG00000188243	COMMD6	chr13:75525219-75549439	-	protein_coding	-0.508284158	0.131445736	-3.866874451	0.000110239	0.000684214	Yes	OK	694.1290603	816.7837323	571.4743884	790.8529277	833.8642973	825.6339718	567.0970871	478.2664021	669.059676
ENSG00000188921	HACD4	chr9:20999515-21031636	-	protein_coding	-0.508188361	0.18384734	-2.764186636	0.005706489	0.022247467	Yes	OK	470.0489285	553.6707246	386.4271325	547.1904055	565.7793265	548.0424418	478.7183203	258.137164	422.4259131
ENSG00000263002	ZNF234	chr19:44141557-44160309	+	protein_coding	-0.507179008	0.172481915	-2.940476442	0.003277079	0.013801507	Yes	OK	218.6723706	257.9292755	179.4154657	249.9641392	264.0837026	259.7399846	208.0583469	137.7787317	192.4093186
ENSG00000143622	RIT1	chr1:155897808-155911404	-	protein_coding	-0.505331105	0.120102708	-4.207491331	2.58221E-05	0.000183679	Yes	OK	844.4362546	992.6162101	696.256299	1015.610599	979.5104606	982.7275707	791.7264528	593.8738436	703.1686007
ENSG00000126218	F10	chr13:113122814-113149529	+	protein_coding	-0.504272918	0.184624937	-2.731336989	0.006307794	0.024213219	Yes	OK	148.4323999	173.5915429	123.2732569	178.5458137	159.2504752	182.9783397	127.9650895	137.7787317	104.0759496
ENSG00000168303	MPLKIP	chr7:40126023-40134652	-	protein_coding	-0.503795974	0.129121912	-3.901707813	9.55164E-05	0.000599847	Yes	OK	385.0670953	450.7605965	319.3735941	473.671541	430.5364606	448.0737879	306.5638474	351.5733154	299.9836195
ENSG00000130635	COL5A1	chr9:134641774-134844843	+	protein_coding	-0.503570016	0.094449321	-5.331642526	9.73284E-08	1.02958E-06	Yes	OK	70575.12929	82765.10266	58385.15591	81096.55885	82796.64279	84402.10635	50910.77275	68278.07172	55966.62325
ENSG00000164603	BMT2	chr7:112819147-112939916	-	protein_coding	-0.502757349	0.175019293	-2.872582446	0.004071318	0.016682574	Yes	OK	578.0916296	679.5846766	476.5985825	716.2837937	621.7970816	700.6731544	593.7948396	335.7366796	500.2642284
ENSG00000106993	CDC37L1	chr9:4679559-4708398	+	protein_coding	-0.501957006	0.152805736	-3.28493562	0.001020057	0.005066426	Yes	OK	420.4849341	494.460166	346.5097022	468.4201936	509.7615714	505.198733	394.9426143	267.6391455	376.9473469
ENSG00000109046	WSB1	chr17:27294076-27315926	+	protein_coding	-0.500610579	0.109152573	-4.58633788	4.51088E-06	3.69215E-05	Yes	OK	2294.883779	2691.466723	1898.300835	2666.634241	2703.25681	2704.509118	2129.375913	1591.581901	1973.944691
ENSG00000058085	LAMC2	chr1:183186238-183244900	+	protein_coding	4.711228175	0.140827881	33.45380301	2.266E-245	2.8691E-242	Yes	OK	1086.087133	80.59119618	2091.58307	95.5745238	71.22257434	74.97649041	2108.201834	1916.232935	2250.31444
ENSG00000164659	KIAA1324L	chr7:86876906-87059699	-	protein_coding	3.986405843	0.13197795	30.2050899	2.0307E-200	1.7593E-197	Yes	OK	793.3147306	93.91347883	1492.715982	82.97128989	95.23018367	103.5389629	1552.152092	1409.460589	1516.535266
ENSG00000064300	NGFR	chr17:49495293-49515017	+	protein_coding	3.972073578	0.166776167	23.81679378	2.2375E-125	8.565E-123	Yes	OK	787.16739	93.59045961	1480.74432	78.77021192	113.6360175	88.36514941	1255.714979	1745.197268	1441.320714
ENSG00000178726	THBD	chr20:23045633-23049741	-	protein_coding	3.938303125	0.146199841	26.93780723	7.9263E-160	4.4989E-157	Yes	OK	1039.863655	127.3793486	1952.347962	133.3842255	118.4375393	130.3162809	1686.561467	2313.732495	1856.749924
ENSG00000157766	ACAN	chr15:88803442-88875354	+	protein_coding	3.929460707	0.11269507	34.86807999	2.2658E-266	3.108E-263	Yes	OK	3025.471721	372.5636498	5678.379792	389.6499816	381.7209883	346.3199795	5095.588275	6513.608317	5425.942784
ENSG00000175899	A2M	chr12:9067664-9116229	-	protein_coding	3.651697953	0.087435867	41.76430199	0	0	Yes	OK	2219.046738	326.8365474	4111.256929	321.3824646	336.9067842	322.2203933	4061.740825	4123.85997	4148.169991
ENSG00000050628	PTGER3	chr1:70852353-71047808	-	protein_coding	3.50461226	0.184746587	18.96983495	3.02856E-80	4.4509E-78	Yes	OK	589.3913883	95.4716417	1083.311135	81.9210204	92.02916909	112.4647356	1294.380689	820.337736	1135.21498
ENSG00000074410	CA12	chr15:63321378-63382161	-	protein_coding	3.452656162	0.084095991	41.05613282	0	0	Yes	OK	2466.857907	413.1986656	4520.517149	421.1580664	410.5301195	407.9078109	4506.396496	4421.588724	4633.566227
ENSG00000081059	TCF7	chr5:134114711-134151865	+	protein_coding	3.428243455	0.179908384	19.05549577	5.91535E-81	8.77177E-79	Yes	OK	584.3998873	98.75610621	1070.043668	74.56913395	97.6309446	124.0682401	933.5007246	1279.600175	997.0301055
ENSG00000135424	ITGA7	chr12:55684568-55716043	-	protein_coding	3.310575604	0.170186449	19.45263924	2.7681E-84	4.46696E-82	Yes	OK	735.6713402	134.2547884	1337.087892	117.6301831	128.0405831	157.093595	1125.908665	1664.430426	1220.924585
ENSG00000086289	EPDR1	chr7:37683843-37951941	+	protein_coding	3.192494815	0.126225322	25.29203141	3.9088E-141	1.8383E-138	Yes	OK	899.4866994	176.9520452	1622.021354	149.1382679	172.8547872	208.8630804	1651.578205	1594.749228	1619.736627
ENSG00000197632	SERPINB2	chr18:63871692-63903890	+	protein_coding	3.04172246	0.152765868	19.91100823	3.26674E-88	5.90885E-86	Yes	OK	502.906299	108.6319673	897.1806306	96.62479329	124.8395685	104.4315402	1018.197043	809.2520909	864.0927581
ENSG00000146197	SCUBE3	chr6:35214419-35253079	+	protein_coding	3.024024725	0.076484632	39.53767777	0	0	Yes	OK	6285.53351	1375.376715	11195.69031	1319.138482	1394.041848	1412.949813	10590.72222	11858.47291	11137.87578
ENSG00000163814	CDCP1	chr3:45082278-45146422	-	protein_coding	2.94456999	0.171982157	17.12136906	1.02828E-65	1.1836E-63	Yes	OK	251.3917927	57.93733961	444.8462458	60.91563055	50.41597959	62.48040867	415.1960817	467.180757	452.1618987

ENSG00000100302	RASD2	chr22:35540868-35554001	+	protein_coding	2.925731725	0.205646921	14.22696589	6.23279E-46	4.81651E-44	Yes	OK	336.0504604	77.82799043	594.2729304	67.21724751	75.22384256	91.04288121	430.8464883	712.6486123	639.3236904
ENSG00000187800	PEAR1	chr1:156893698-156916434	+	protein_coding	2.906421426	0.148089411	19.62612586	9.2505E-86	1.50756E-83	Yes	OK	1342.01793	315.1769933	2368.858867	291.9749189	323.3024723	330.2535887	1962.745113	2950.365255	2193.466232
ENSG00000122862	SRGN	chr10:69088106-69104811	+	protein_coding	2.901734083	0.185013659	15.68389112	1.94938E-55	1.82311E-53	Yes	OK	275.208589	65.08829297	485.3288851	60.91563055	65.62079883	68.72844954	574.4619844	402.2505501	479.2741209
ENSG00000123496	IL13RA2	chrX:115003975-115019977	-	protein_coding	2.846493985	0.157697984	18.05028777	7.84808E-73	1.01716E-70	Yes	OK	407.6637888	100.3006811	715.0268964	121.8312611	88.02790087	91.04288121	767.7905368	646.1347418	731.1554107
ENSG00000090530	P3H2	chr3:189956728-190122437	-	protein_coding	2.818196311	0.128112176	21.9978803	3.0175E-107	8.2781E-105	Yes	OK	809.6918068	201.5249408	1417.858673	235.2603663	195.2618892	174.052567	1404.854148	1504.480404	1344.241467
ENSG00000101680	LAMA1	chr18:6941744-7117814	-	protein_coding	2.796501725	0.124533897	22.45574733	1.1245E-111	3.5595E-109	Yes	OK	746.9023105	188.4904096	1305.314211	214.2549764	166.452758	184.7634942	1282.412731	1379.370981	1254.158922
ENSG00000145681	HAPLN1	chr5:83637805-83721613	-	protein_coding	2.76505801	0.136286762	20.28852971	1.62379E-91	3.25946E-89	Yes	OK	3893.491363	998.935804	6788.046922	972.5495499	1053.133796	971.1240663	8126.243487	5207.085861	7030.811419
ENSG00000153132	CLGN	chr4:140388455-140427968	-	protein_coding	2.758427429	0.191077757	14.43615142	3.06484E-47	2.43707E-45	Yes	OK	531.9214537	137.337425	926.5054824	147.0377289	148.0469242	116.9276219	1163.653763	673.0570228	942.8056611
ENSG00000143127	ITGA10	chr1:145891208-145910189	-	protein_coding	2.745531386	0.166151118	16.52430278	2.45259E-61	2.57132E-59	Yes	OK	272.9571434	71.38215957	474.5321272	81.9210204	60.81927696	71.40618134	474.1152595	446.5931304	502.8879918
ENSG00000159674	SPON2	chr4:1166932-1208962	-	protein_coding	2.7010606	0.243248761	11.10410834	1.19807E-28	5.09567E-27	Yes	OK	7047.65587	1878.368088	12216.94365	1766.553286	1925.410268	1943.14071	9355.260714	16145.45023	11150.12001
ENSG00000049759	NEDD4L	chr18:58044367-58401540	+	protein_coding	2.675738846	0.12666973	21.12374312	4.8126E-99	1.11572E-96	Yes	OK	562.1972564	151.8328484	972.5616643	145.9874594	148.8471778	160.663908	958.3572528	1043.634301	915.693439
ENSG00000203805	PLPP4	chr10:120456954-120589855	+	protein_coding	2.66241423	0.195424818	13.62372627	2.89416E-42	2.0358E-40	Yes	OK	234.1435204	64.00702204	404.2800187	66.16697801	46.41471137	79.43937674	372.8479226	448.176794	391.8153397
ENSG00000169851	PCDH7	chr4:30720415-31146805	+	protein_coding	2.661184468	0.119216117	22.32235488	2.2417E-110	6.5889E-108	Yes	OK	616.7516761	168.3926482	1065.110704	172.2441967	162.4514898	170.482258	1078.036833	1105.397181	1011.898098
ENSG00000104435	STMN2	chr8:79610814-79666175	+	protein_coding	2.65087424	0.128409925	20.64384235	1.10887E-94	2.3704E-92	Yes	OK	1049.526665	289.3208848	1809.732445	324.5332731	248.8788834	294.550498	1986.681029	1613.753191	1828.763114
ENSG00000175084	DES	chr2:219418377-219426739	+	protein_coding	2.63386373	0.13007036	20.24953062	3.58641E-91	7.11232E-89	Yes	OK	760.2668438	210.0605285	1310.473159	183.7971612	231.2733032	215.1111213	1215.208044	1458.55416	1257.657273
ENSG00000006016	CRLF1	chr19:18572220-18607741	-	protein_coding	2.618966586	0.15094417	17.35056474	1.95315E-67	2.31286E-65	Yes	OK	2387.454536	668.1032625	4106.80581	706.8313683	645.0044373	652.473982	3250.681517	5286.26904	3783.466874
ENSG00000144115	THNSL2	chr2:88170295-88186636	+	protein_coding	2.585709757	0.20850941	12.40092595	2.58321E-35	1.42683E-33	Yes	OK	177.3341727	50.226267	304.4420784	47.26212715	55.21750145	48.19917241	255.0095668	370.5772784	287.7393901
ENSG00000067177	PHKA1	chrX:72578814-72714319	-	protein_coding	2.566405484	0.13465603	19.05897191	5.53518E-81	8.28264E-79	Yes	OK	566.6440743	163.8743902	969.4137584	185.8977001	158.4502216	147.275249	897.5968506	1054.719946	955.9244783
ENSG00000138623	SEMA7A	chr15:74409289-74433958	-	protein_coding	2.56613722	0.109372851	23.46228694	9.9039E-122	3.4685E-119	Yes	OK	8884.818147	2565.983057	15203.65324	2462.881959	2676.048186	2559.019024	13208.02258	18080.68713	14322.25001
ENSG00000118473	SGIP1	chr1:66533383-66748299	+	protein_coding	2.527114635	0.162956945	15.507867	3.06906E-54	2.77564E-52	Yes	OK	376.1881513	111.8208469	640.5554557	120.7809916	88.82815451	125.8533946	711.6331954	581.2045349	628.8286367
ENSG00000147862	NFIB	chr9:14081843-14398983	-	protein_coding	2.48507223	0.146839958	16.92367838	3.01028E-64	3.28141E-62	Yes	OK	319.0012307	96.53976989	541.4626915	91.37344583	92.02916909	106.2166947	530.272601	560.6169084	533.4985652
ENSG00000114948	ADAM23	chr2:206443539-206621130	+	protein_coding	2.483765493	0.115225387	21.55571406	4.6799E-103	1.1497E-100	Yes	OK	612.9112142	185.7094607	1040.112968	179.5960832	193.6613819	183.870917	1055.942141	1034.13232	1030.264442
ENSG00000104723	TUSC3	chr8:15417215-15766649	+	protein_coding	2.473977234	0.109612201	22.57027249	8.4921E-113	2.7956E-110	Yes	OK	1423.891103	434.7732737	2413.008931	420.1077969	436.1382361	448.0737879	2584.158318	2114.190883	2540.677593
ENSG00000165323	FAT3	chr11:92352096-92896470	+	protein_coding	2.439372771	0.129357717	18.85757446	2.54636E-79	3.64462E-77	Yes	OK	513.9874599	160.1646972	867.8102226	163.8420408	172.0545335	144.5975172	906.8029721	812.4194181	884.2082777
ENSG00000173376	NDNF	chr4:121035613-121073021	-	protein_coding	2.436368338	0.098629734	24.70216885	1.0136E-134	4.5092E-132	Yes	OK	1001.004493	312.223187	1689.785798	309.8295002	307.2973994	319.5426615	1658.943102	1686.601716	1723.812577
ENSG00000108511	HOXB6	chr17:48595751-48604992	-	protein_coding	2.418874032	0.212669024	11.37388973	5.64162E-30	2.54414E-28	Yes	OK	201.6333235	63.29527676	339.9713703	71.41832548	64.02029154	54.44721327	301.0401745	435.5074853	283.366451
ENSG00000157570	TSPAN18	chr11:44726465-44932421	+	protein_coding	2.396729499	0.156416222	15.32276815	5.38734E-53	4.76751E-51	Yes	OK	891.8857471	284.3753701	1499.396124	264.6679121	237.6753323	350.7828658	1338.570073	1751.531923	1408.086377
ENSG00000162981	FAM84A	chr2:14632686-14650814	+	protein_coding	2.369520797	0.204124859	11.60819318	3.74444E-31	1.79167E-29	Yes	OK	176.2983299	56.54237267	296.0542872	44.11131868	64.82054518	60.69525414	265.1363005	348.4059883	274.6205729
ENSG00000184160	ADRA2C	chr4:3766348-3768526	+	protein_coding	2.3594166	0.207735602	11.35778645	6.78419E-30	3.04272E-28	Yes	OK	464.4288715	151.0389949	777.818748	142.836651	134.4426122	175.8377216	578.144433	1062.638264	692.673547
ENSG00000170961	HAS2	chr8:121612116-121641390	-	protein_coding	2.348073364	0.108111621	21.71897295	1.3578E-104	3.5475E-102	Yes	OK	4684.350196	1538.953135	7829.747257	1615.314479	1541.288519	1460.256408	8835.114846	6607.044468	8047.082456
ENSG00000183578	TNFAIP8L3	chr15:51056598-51105276	-	protein_coding	2.342930297	0.162183844	14.44613869	2.65139E-47	2.11854E-45	Yes	OK	242.4177266	79.84507948	404.9903736	80.87075091	79.22511078	79.43937674	371.0066982	410.168868	433.7955547
ENSG00000113645	WWC1	chr5:168291651-168472303	+	protein_coding	2.316891582	0.181567925	12.76046739	2.72561E-37	1.62549E-35	Yes	OK	188.4777316	63.06705045	313.8884127	69.31778649	67.22130612	52.66205874	292.7546651	318.3163802	330.5941929
ENSG00000171873	ADRA1D	chr20:4220631-4249074	-	protein_coding	2.294909761	0.234831591	9.7725768	1.47648E-22	4.86058E-21	Yes	OK	148.8631148	50.13124719	247.5949824	53.56374411	50.41597959	46.41401787	184.1224309	319.9000438	238.7624726
ENSG00000124126	PREX1	chr20:48624252-48827883	-	protein_coding	2.287871477	0.215631852	10.61008131	2.67516E-26	1.03852E-24	Yes	OK	286.4071997	97.03969027	475.774709	106.0772187	90.4286618	94.61319028	370.0860861	654.0530598	403.1849812
ENSG00000103196	CRISPLD2	chr16:84819984-84920768	+	protein_coding	2.276225119	0.076084545	29.91704978	1.1811E-196	9.7201E-194	Yes	OK	3853.475503	1318.381028	6388.569978	1299.183362	1279.605577	1376.354145	6203.084697	6657.721703	6304.903535
ENSG00000183691	NOG	chr17:56593699-56595590	+	protein_coding	2.275364288	0.193041951	11.78689024	4.56072E-32	2.24759E-30	Yes	OK	462.5294438	157.7000292	767.3588584	153.3393459	176.0558017	143.70494	605.7627976	1024.630338	671.6834395
ENSG00000105825	TFPI2	chr7:93885397-93890991	-	protein_coding	2.256017777	0.10525787	21.43324555	6.5455E-102	1.5844E-99	Yes	OK	6455.607046	2235.52217	10675.69192	2139.398956	2299.12872	2268.038835	12007.54433	8960.368552	11059.16288
ENSG00000197106	SLC6A17	chr1:110150486-110202202	+	protein_coding	2.226969318	0.192578079	11.56398135	6.27313E-31	2.97567E-29	Yes	OK	228.5424685	79.8337365	377.2512004	71.41832548	90.4286618	77.65422221	310.2462961	457.6787755	363.8285297
ENSG00000104490	NCALD	chr8:101686543-102124907	-	protein_coding	2.195064959	0.116333406	18.86874143	2.0615E-79	2.97652E-77	Yes	OK	642.8653835	229.9290516	1055.801715	205.8528205	238.475586	245.4587484	1052.259693	1067.389255	1047.756199
ENSG00000135454	B4GALNT1	chr12:57623410-57633355	-	protein_coding	2.185494281	0.14917796	14.65024912	1.34223E-48	1.08833E-46	Yes	OK	428.635264	153.9219331	703.3485948	151.2388069	163.2517434	147.275249	610.3658584	814.0030816	685.6768445
ENSG00000095752	IL11	chr19:55364389-55370463	-	protein_coding	2.177188218	0.160815578	13.53841611	9.27676E-42	6.38893E-40	Yes	OK	421.0166123	152.2415685	689.7916561	170.1436578	146.4464169	140.1346309	620.4925921	831.4233811	617.4589951
ENSG00000060718	COL11A1	chr1:102876467-103108872	-	protein_coding	2.135146766	0.082971087	25.73362419	4.9166E-146	2.3802E-143	Yes	OK	5642.734728	2093.206885	9192.26257	2024.919581	2106.267592	2148.433481	9678.39558	8326.903119	9571.489012
ENSG00000197757	HOXC6	chr12:53990624-54030823	+	protein_coding	2.108217217	0.103524423	20.36444302	3.45762E-92	7.02622E-90	Yes	OK	1635.560757	614.7880727	2656.33344	556.6428309	628.9993644	658.7220229	2514.191794	2887.018712	2567.789815
ENSG00000154277	UCHL1	chr4:41256413-41268455	+	protein_coding	2.094575251	0.067617219	30.97695035	1.1019E-210	1.0669E-207	Yes	OK	6302.331504	2390.423158	10214.23985	2320.045308	2405.562455	2445.661711	10075.17942	10578.87273	9988.667399
ENSG00000154175	ABI3BP	chr3:100749156-100993515	-	protein_coding	2.093203325	0.104149037	20.09815349	7.65908E-90	1.44906E-87	Yes	OK	2832.546555	1076.407747	4588.685363	1076.52623	1077.141405	1075.555606	5044.954606	3927.485686	4793.615797
ENSG00000164220	F2RL2	chr5:76615482-76623434	-	protein_coding	2.089282126	0.200009193	10.44593046	1.52948E-25	5.82761E-24	Yes	OK	166.4670668	63.62627325	269.3078603	65.11670852	70.42232069	55.33979054	295.5165016	218.5455744	293.8615048
ENSG00000127241	MASP1	chr3:187217285-187292022	-	protein_coding	2.050969911	0.09158522	22.3941147	4.4915E-111	1.3442E-108	Yes	OK	5078.263828	1973.177625	8183.350031	1869.479696	2007.836393	2042.216786	7711.968018	9158.3265	7679.755575
ENSG00000092068	SLC7A8	chr14:23125295-23183674	-	protein_coding	2.04619704	0.100130294	20.43534445	8.11184E-93	1.66901E-90	Yes	OK	1921.111413	748.5541698	3093.668656	745.6913395	700.2219387	799.749231	2864.024413	3379.538086	3037.44347
ENSG00000182253	SYNM	chr15:99098217-99135593	+	protein_coding	2.043632258	0.131134265	15.58427358	9.31158E-55	8.56249E-53	Yes	OK	432.8342158	169.5082863	696.1601453	178.5458137	164.8522507	165.1267944	742.0133965	639.8000875	706.6669519
ENSG00000072041	SLC6A15	chr12:84859488-84913615	-	protein_coding	2.02444857	0.2239019	9.041676596	1.54287E-19	4.40896E-18	Yes	OK	191.3551944	76.00408836	306.7063004	86.12209837	77.62460349	64.26556321	391.2601656	220.129238	308.7294976
ENSG00000173641	HSPB7	chr1:16014028-16019594	-	protein_coding	2.022819863	0.10453621	19.3504229	2.02176E-83	3.13946E-81	Yes	OK	20895.79759	8251.976007	33539.61917	8241.464706	8154.584635	8359.878681	29466.95384	40052.43568	31099.468
ENSG00000048740	CELF2	chr10:10798397-11336675	+	protein_coding	2.008847471	0.105364328	19.06572663	4.86473E-81	7.34618E-79	Yes	OK	1003.929716	399.8854835	1607.973948	432.7110308	408.1293586	358.8160612	1578.849845	1620.087845	1624.984154
ENSG00000198513	ATL1	chr14:50532509-50633068	+	protein_coding	2.007546478	0.162307158	12.36881046	3.85488E-35	2.08037E-33	Yes	OK	240.1283564	95.70616109	384.5505517	97.67506278	106.4337347	83.00968581	409.6724087	362.6589605	381.3202859
ENSG00000189056	RELN	chr7:103471784-103989516	-	protein_coding	1.999763905	0.251132418	7.962985909	1.67937E-15	3.75066E-14	Yes	OK	129.5025969	51.60618702	207.3990069	51.46320512	71.22257434	32.1327816	231.0736508	207.4599294	183.6634405
ENSG00000180806	HOXC9	chr12:53994895-54003337	+	protein_coding	1.989678165	0.152005779	13.08949031	3.78137E-39	2.4313E-37	Yes	OK	378.9925392	152.034231	605.9508473	142.836651	147.2466705	166.0193716	516.4634187	677.8080135	623.5811098
ENSG00000163064	EN1	chr2:118842171-118847678	-	protein_coding	1.978449368	0.131340028	15.06356744	2.81195E-51	2.38581E-49	Yes	OK	1412.508233	570.7812074	2254.235259	536.6877106	571.381102	604.2748096	2049.282656	2725.485026	1987.938096
ENSG00000132205	EMILIN2	chr18:2847030-2915993	+	protein_coding	1.946014548	0.088812541	21.91148386	2.019E-106	5.36E-104	Yes	OK	2910.778878	1198.878083	4622.679673	1188.905065	1164.369052	1243.360133	4298.338149	5029.715539	4539.985331
ENSG00000136928	GABBR2	chr9:98288082-98709197	-	protein_coding	1.945449196	0.102920229	18.9024957	1.08783E-79	1.58457E-77	Yes	OK	1438.312579	593.4078837	2283.217274	659.5692412	552.9752682	567.6791417	2156.073666	2388.164683	2305.413472
ENSG00000162595	DIRAS3	chr1:68045896-68051631	-	protein_coding	1.943992083	0.160519663	12.11061649	9.27941E-34	4.7731E-32	Yes	OK	322.3745789	132.8917193	511.8574385	110.2782967	138.4438805	149.9529808	574.4619844	460.8461026	500.2642284
ENSG00000187479	C11orf96	chr11:43942505-43944338	+	protein_coding	1.939399124	0.272959135	7.105089653	1.20244E-12	2.20896E-11	Yes	OK	195.5687722	80.49516436	310.6423801	84.02155938	66.42105247	91.04288121	219.1056928	476.6827385	236.1387092
ENSG00000128567	PODXL	chr7:131500262-131558217	-	protein_coding	1.926539314	0.069070218	27.89247479	3.2922E-171	2.2579E-168	Yes	OK	9623.309034	4007.296944	15239.32112	3909.10305	4060.486991	4052.300791	15029.91403	16137.53191	14550.51743
ENSG00000104332	SFRP1	chr8:41261958-41309497	-	protein_coding	1.914958574	0.066003925	29.01279858	4.5371E-185	3.3946E-182	Yes	OK	5676.101145	2378.749342	8973.452947	2304.291266	2402.36144	2429.59532	8854.447702	9083.894312	8982.016827
ENSG00000120093	HOXB3	chr17:48548870-48604912	-	protein_coding	1.893121868	0.114087492	16.59359698	7.75345E-62	8.18088E-60	Yes	OK	726.6733597	308.7010617	1144.645658	341.337585	292.8928338	291.8727662	1088.163567	1170.327388	1175.446019
ENSG00000152284	TCF7L1	chr2:85133410-85310388	+	protein_coding	1.877767562	0.161240044	11.64578921	2.4108E-31	1.16711E-29	Yes	OK	699.5078925	298.5523874	1100.463398	305.6284223	293.6930874	296.3356526	916.9297058	1411.044252	973.4162345
ENSG00000182575	NXPH3	chr17:49575858-49583827	+	protein_coding	1.871912143	0.207670372	9.013862307	1.98924E-19	5.63562E-18	Yes	OK	260.3882424	111.0914698	409.6850149	111.3285662	126.4400758	95.50576755	353.5150673	543.196609	332.3433685
ENSG00000090975	PITPNM2	chr12:122983480-123150015	-	protein_coding	1.87049865	0.166679112	11.22215391	3.17443E-29	1.38597E-27	Yes	OK	576.6879645	247.3371731	906.0387559	263.6176426	216.8687376	261.5251392	728.2042142	1127.568471	862.3435824
ENSG00000176435	CLEC14A	chr14:38254103-38256369	-	protein_coding	1.857063719	0.200521691	9.261161286	2.02221E-20	6.09967E-19	Yes	OK	184.99477	79.39132304	290.598217	70.36805598	92.82942273	74.97649041	238.438548	349.9896518	283.366451
ENSG00000163710	PCOLCE2	chr3:142815922-142889203	-	protein_coding	1.845301933	0.093051236	19.83103094	1.60712E-87	2.81339E-85	Yes	OK	1201.120218	523.133272	1879.107165	526.1850156	496.9575131	546.2572873	1843.986145	1897.228972	1896.106376
ENSG00000113504	SLC12A7	chr5:1050376-1112035	-	protein_coding	1.841668382	0.221984441	8.296384968	1.07327E-16	2.56774E-15	Yes	OK	308.1208927	133.6623218	482.5794637	119.7307221	138.4438805	142.8123627	375.609759	687.309995	384.8186372
ENSG00000227051	C14orf132	chr14:96039324-96093889	+	protein_coding	1.832090118	0.081283285	22.53956791	1.6997E-112	5.4857E-110	Yes	OK	2669.950868	1170.355816	4169.54592	1154.246172	1149.164233	1207.657042	3954.029203	4374.078816	4180.52974
ENSG00000149948	HMGA2	chr12:65824131-65966295	+	protein_coding	1.820674642	0.127394802	14.29159281	2.46897E-46	1.92603E-44	Yes	OK	537.2146582	237.1951302	837.2341863	224.7576714	252.0798979	234.7478212	918.7709302	758.5748562	834.3567725
ENSG00000160255	ITGB2	chr21:44885953-44931989	-	protein_coding	1.81772038	0.20088185	9.048703917	1.44675E-19	4.1487E-18	Yes	OK	145.9376984	64.13809746	227.7372993	55.66428309	68.02155976	68.72844954	190.566716	248.6351825	244.0099995
ENSG00000106003	LFNG	chr7:2512529-2529177	+	protein_coding	1.809041566	0.340632183	5.310835729	1.09124E-07	1.14479E-06	Yes	OK	113.956562	50.41821507	177.4949089	70.36805598	41.6131895	39.27339974	113.235295	289.8104357	129.4389961
ENSG00000198108	CHSY3	chr5:129904472-130186634	+	protein_coding	1.80193794	0.12933516	13.93231309	4.03086E-44	2.98865E-42	Yes	OK	476.0253584	212.2455295	739.8051874	200.601473	227.272035	208.8630804	805.5356352	680.9753407	732.9045863
ENSG00000188153	COL4A5	chrX:108439844-108697545	+	protein_coding	1.781651164	0.201024069	8.862874861	7.79771E-19	2.12149E-17	Yes	OK	204.0115126	92.00343064	316.0195945	115.5296442	94.42993002	66.05071774	283.5485436	353.156979	311.353261
ENSG00000161544	CYGB	chr17:76527356-76551175	-	protein_coding	1.77028	0.174817809	10.12642824	4.21775E-24	1.51913E-22	Yes	OK	388.5156642	175.7356609	601.2956674	186.9479696	160.8509825	179.4080306	476.877096	750.6565383	576.353368
ENSG00000006747	SCIN	chr7:12570577-12660179	+	protein_coding	1.769550956	0.191147504	9.257515356	2.09245E-20	6.29646E-19	Yes	OK	255.676224	116.6644863	394.6879618	130.233417	91.22891544	128.5311264	469.5121988	326.2346981	388.3169884
ENSG00000159167	STC1	chr8:23841915-23854807	-	protein_coding	1.763153079	0.091690546	19.22938796	2.10093E-82	3.20197E-80	Yes	OK	1404.577009	639.9901602	2169.163858	662.7200496	614.5947988	642.6556321	2272.991409	2077.766621	2156.733544
ENSG00000170989	S1PR1	chr1:101236888-101241518	+	protein_coding	1.744058592	0.104457816	16.69629584	1.39458E-62	1.49057E-60	Yes	OK	779.6804388	358.2765657	1201.084312	342.3878545	351.3113498	381.1304929	1173.780497	1214.669968	1214.802471
ENSG00000136960	ENPP2	chr8:119557086-119673453	-	protein_coding	1.731009426	0.089255477	19.39387354	8.69365E-84	1.36283E-81	Yes	OK	7065.968603	3272.476813	10859.46039	3228.528419	3381.871901	3207.03012	11675.20334	9543.156751	11360.02109

ENSG00000135678	CPM	chr12:68842197-68971570	-	protein_coding	1.727987758	0.17736481	9.742562559	1.98485E-22	6.50809E-21	Yes	OK	265.3563987	123.5565544	407.156243	128.1328781	133.6423586	108.8944265	427.1640397	324.6510345	469.6536549
ENSG00000173705	SUSD5	chr3:33150042-33219215	-	protein_coding	1.727021203	0.236084929	7.315253916	2.56895E-13	4.96885E-12	Yes	OK	122.2867176	57.19728908	187.3761462	68.267517	56.0177551	47.30659514	236.5973237	148.8643768	176.666738
ENSG00000164949	GEM	chr8:94249253-94262350	-	protein_coding	1.726248579	0.145479294	11.86594007	1.77893E-32	8.92719E-31	Yes	OK	348.9321003	162.1307908	535.7334099	152.2890764	160.0507288	174.052567	603.9215733	492.5193743	510.7592821
ENSG00000064309	CDON	chr11:125955796-126063335	-	protein_coding	1.719836824	0.14013244	12.27293855	1.26575E-34	6.6991E-33	Yes	OK	408.498967	189.6507527	627.3471814	163.8420408	217.6689912	187.441226	594.7154518	636.6327603	650.693332
ENSG00000168477	TNXB	chr6:32041154-32115334	-	protein_coding	1.716696261	0.177374018	9.678397565	3.72509E-22	1.20699E-20	Yes	OK	967.1097122	450.6231167	1483.596308	478.9228885	394.5250466	478.421415	1181.145394	1987.497797	1282.145732
ENSG00000145358	DDIT4L	chr4:100185870-100190782	-	protein_coding	1.716543305	0.197254206	8.702188628	3.25544E-18	8.62875E-17	Yes	OK	228.387276	107.2823723	349.4921796	117.6301831	94.42993002	109.7870038	420.7197546	261.3044912	366.4522931
ENSG00000151617	EDNRA	chr4:147480917-147544954	+	protein_coding	1.710082126	0.162511442	10.52284135	6.77976E-26	2.60128E-24	Yes	OK	195.1410568	91.58819484	298.6939188	94.52425431	85.62713993	94.61319028	301.0401745	289.8104357	305.2311463
ENSG00000184227	ACOT1	chr14:73537114-73543794	+	protein_coding	1.704859261	0.245379998	6.947833045	3.7094E-12	6.41351E-11	Yes	OK	116.4761669	54.19282985	178.7595039	52.51347461	67.22130612	42.84370881	140.8536596	224.8802288	170.5446233
ENSG00000164283	ESM1	chr5:54977864-55022671	-	protein_coding	1.697273873	0.193895131	8.753566251	2.06714E-18	5.52357E-17	Yes	OK	127.6993741	60.23802656	195.1607217	63.01616954	55.21750145	62.48040867	200.6934497	201.125275	183.6634405
ENSG00000119771	KLHL29	chr2:23385217-23708611	+	protein_coding	1.695741002	0.159354271	10.64132758	1.91383E-26	7.53627E-25	Yes	OK	260.7516393	122.5562641	398.9470145	123.9318001	123.2390612	120.497931	352.5944552	457.6787755	386.5678128
ENSG00000082497	SERTAD4	chr1:210232799-210246631	+	protein_coding	1.691648977	0.143500404	11.78846139	4.47644E-32	2.21934E-30	Yes	OK	370.1170752	175.6550185	564.5791318	179.5960832	168.8535189	178.5154534	603.9215733	484.6010564	605.2147658
ENSG00000135480	KRT7	chr12:52232520-52252186	+	protein_coding	1.678859302	0.112784017	14.88561362	4.08719E-50	3.41498E-48	Yes	OK	3118.449061	1483.092069	4753.806053	1395.808155	1524.483192	1528.984858	4075.550008	5519.067587	4666.800564
ENSG00000120594	PLXDC2	chr10:19816239-20289856	+	protein_coding	1.668044757	0.182442801	9.14283681	6.08352E-20	1.79775E-18	Yes	OK	298.7557035	143.4880838	454.0233232	134.434495	126.4400758	169.5896807	544.0817833	365.8262877	452.1618987
ENSG00000138696	BMPR1B	chr4:94757968-95158448	+	protein_coding	1.665138628	0.201789048	8.251878092	1.55924E-16	3.69815E-15	Yes	OK	184.1993065	88.9950363	279.4035766	96.62479329	78.42485714	91.93545848	324.9760905	210.6272565	302.6073829
ENSG00000135299	ANKRD6	chr6:89433170-89633834	+	protein_coding	1.660460331	0.237760319	6.983757172	2.87389E-12	5.05386E-11	Yes	OK	135.6711612	65.68031008	205.6620123	84.02155938	41.6131895	71.40618134	186.8842674	202.7089386	227.3928311
ENSG00000180921	FAM83H	chr8:143723933-143733801	-	protein_coding	1.658335216	0.240093062	6.907051811	4.94829E-12	8.40546E-11	Yes	OK	174.5337471	83.5063662	265.561128	88.22263735	86.42739358	75.86906768	179.5193701	362.6589605	254.5050532
ENSG00000055163	CYFIP2	chr5:157266079-157395595	+	protein_coding	1.655844341	0.224047283	7.390602198	1.46165E-13	2.87785E-12	Yes	OK	112.4841622	53.93360209	171.0347224	58.81509157	51.21623323	51.76948147	165.7101878	207.4599294	139.9340499
ENSG00000148344	PTGES	chr9:129738331-129753047	-	protein_coding	1.648541617	0.152614866	10.8019727	3.36887E-27	1.35578E-25	Yes	OK	447.6038946	216.2716147	678.9361745	238.4111748	187.2593528	223.1443167	616.8101435	798.1664458	621.8319342
ENSG00000111859	NEDD9	chr6:11183298-11382348	-	protein_coding	1.641693845	0.100179509	16.38752143	2.34826E-60	2.38595E-58	Yes	OK	894.8744802	434.6642147	1355.084746	423.2586054	435.3379825	445.3960561	1393.806802	1284.351166	1387.09627
ENSG00000119630	PGF	chr14:74941834-74955784	-	protein_coding	1.641656434	0.29268108	5.609028213	2.03466E-08	2.34856E-07	Yes	OK	127.8941921	61.46418519	194.3241991	58.81509157	72.02282798	53.55463601	149.139169	300.8960808	132.9373474
ENSG00000101000	PROCR	chr20:35172073-35216240	+	protein_coding	1.62887764	0.149213108	10.9164514	9.61806E-28	3.92837E-26	Yes	OK	369.050236	180.0217659	558.0787061	184.8474306	193.6613819	161.5564853	479.6389325	603.3758251	591.2213608
ENSG00000143469	SYT14	chr1:209938174-210171389	+	protein_coding	1.624373814	0.217666874	7.462659722	8.47931E-14	1.69793E-12	Yes	OK	350.0548916	172.1718676	527.9379156	183.7971612	172.0545335	160.663908	666.5231998	327.8183617	589.4721852
ENSG00000162599	NFIA	chr1:60865259-61462793	+	protein_coding	1.616092004	0.207584703	7.785217234	6.95935E-15	1.50329E-13	Yes	OK	165.8445568	81.74592003	249.9431936	66.16697801	88.02790087	91.04288121	290.9134408	194.7906207	264.1255192
ENSG00000157551	KCNJ15	chr21:38155549-38307357	+	protein_coding	1.607684402	0.228483013	7.036341039	1.97354E-12	3.54246E-11	Yes	OK	115.108551	57.07059403	173.1465079	48.31239665	48.8154723	74.08391314	184.1224309	147.2807132	188.0363795
ENSG00000073756	PTGS2	chr1:186671791-186680427	-	protein_coding	1.597360751	0.142153096	11.23690443	2.68638E-29	1.176E-27	Yes	OK	560.8582142	279.4646864	842.251742	293.0251884	278.4882682	266.8806028	958.3572528	692.0609858	876.3369874
ENSG00000170745	KCNS3	chr2:17877847-18361616	+	protein_coding	1.587414958	0.163642534	9.700503382	3.00015E-22	9.74012E-21	Yes	OK	235.5777817	117.5884032	353.5671602	105.0269492	127.2403294	120.497931	398.6250629	326.2346981	335.8417197
ENSG00000142910	TINAGL1	chr1:31576485-31587686	+	protein_coding	1.580672728	0.25590914	6.176695087	6.54573E-10	8.96362E-09	Yes	OK	142.3476222	70.70017682	213.9950676	69.31778649	77.62460349	65.15814047	156.5040663	308.8143987	176.666738
ENSG00000023171	GRAMD1B	chr11:123358428-123627774	+	protein_coding	1.57467488	0.108931915	14.45558798	2.31147E-47	1.85594E-45	Yes	OK	1016.387155	510.0925717	1522.681738	508.3304343	526.5668979	495.3803831	1386.441905	1683.434389	1498.168922
ENSG00000250722	SELENOP	chr5:42799880-42887392	-	protein_coding	1.557259966	0.244886172	6.359117597	2.02916E-10	2.91998E-09	Yes	OK	98.71591311	50.41091425	147.020912	47.26212715	50.41597959	53.55463601	146.3773326	109.2727872	185.4126161
ENSG00000100558	PLEK2	chr14:67386983-67412200	-	protein_coding	1.549983551	0.221809041	6.987918736	2.78994E-12	4.91674E-11	Yes	OK	221.0913799	111.7885064	330.3942533	76.66967294	156.0494606	102.6463857	311.1669082	372.160942	307.8549098
ENSG00000164929	BAALC	chr8:103140710-103230305	+	protein_coding	1.539309676	0.099751671	15.43141746	1.00635E-53	9.0025E-52	Yes	OK	1330.892554	680.5488026	1981.236306	664.8205886	669.0120466	707.8137726	1839.383085	2160.117127	1944.208706
ENSG00000115041	KCNIP3	chr2:95297304-95386083	+	protein_coding	1.536096663	0.212211062	7.238532477	4.53566E-13	8.62089E-12	Yes	OK	311.6949176	158.8418372	464.5479979	130.233417	193.6613819	152.6307126	385.7364927	623.9634517	383.9440494
ENSG00000168874	ATOH8	chr2:85751344-85788066	+	protein_coding	1.528406016	0.118967608	12.84724512	8.91281E-38	5.39356E-36	Yes	OK	2511.238403	1291.690524	3730.786282	1235.116923	1263.600504	1376.354145	3257.125802	4434.258032	3500.975011
ENSG00000137507	LRRC32	chr11:76657524-76670747	-	protein_coding	1.52542922	0.123187734	12.38296359	3.23185E-35	1.75565E-33	Yes	OK	2287.723156	1178.335555	3397.110757	1143.743477	1198.779959	1192.483228	2969.89481	4117.525316	3103.912144
ENSG00000134508	CABLES1	chr18:23134564-23260467	+	protein_coding	1.515814203	0.129807888	11.67736592	1.66372E-31	8.0781E-30	Yes	OK	693.5857238	358.329468	1028.841979	326.6338121	368.1166764	380.2379156	939.0243976	1184.58036	962.9211808
ENSG00000125384	PTGER2	chr14:52314305-52328606	+	protein_coding	1.5001166	0.206132784	7.277428514	3.40243E-13	6.52728E-12	Yes	OK	127.9570457	66.73990279	189.1741886	69.31778649	56.81800874	74.08391314	162.0277392	212.2109201	193.2839064
ENSG00000184349	EFNA5	chr5:107376889-107670895	-	protein_coding	1.498997881	0.160951975	9.313323936	1.23893E-20	3.79048E-19	Yes	OK	232.3263291	121.066166	343.5864921	121.8312611	136.0431195	105.3241175	323.1348662	367.4099512	340.2146588
ENSG00000095303	PTGS1	chr9:122370530-122395703	+	protein_coding	1.496830043	0.103754743	14.42661796	3.51919E-47	2.7849E-45	Yes	OK	1005.939432	525.7566511	1486.122212	497.8277393	569.7805947	509.6616193	1547.549032	1504.480404	1406.337201
ENSG00000158859	ADAMTS4	chr1:161184308-161199056	-	protein_coding	1.491806798	0.161056137	9.262651061	1.99418E-20	6.03387E-19	Yes	OK	592.7582274	310.2978608	875.2185941	329.7846206	290.4920729	310.6168888	721.7599291	1105.397181	798.4986722
ENSG00000205213	LGR4	chr11:27365961-27472775	-	protein_coding	1.491585241	0.119535413	12.478187	9.81971E-36	5.4977E-34	Yes	OK	2995.265019	1572.499306	4418.030732	1536.544267	1587.70323	1593.250421	5069.811135	3574.328707	4609.952356
ENSG00000221890	NPTXR	chr22:38818452-38843982	-	protein_coding	1.491205425	0.162630984	9.169257867	4.76287E-20	1.41001E-18	Yes	OK	198.1195408	103.9917303	292.2473514	111.3285662	94.42993002	106.2166947	286.31038	308.8143987	281.6172754
ENSG00000110900	TSPAN11	chr12:30926428-30996599	+	protein_coding	1.482802632	0.209978357	7.061692692	1.64486E-12	2.98835E-11	Yes	OK	151.3815046	79.24755867	223.5154506	78.77021192	92.02916909	66.94329501	221.8675292	267.6391455	181.039677
ENSG00000106366	SERPINE1	chr7:101127089-101139266	+	protein_coding	1.462575489	0.073039732	20.02438185	3.37677E-89	6.31609E-87	Yes	OK	74637.47976	39741.1876	109533.7719	38829.5134	40149.52559	40244.5238	101061.1199	120767.0175	106773.1784
ENSG00000185760	KCNQ5	chr6:72621792-73198851	+	protein_coding	1.450457821	0.115607866	12.54635927	4.16211E-36	2.36235E-34	Yes	OK	695.085359	372.9372134	1017.233505	367.5943223	389.7235247	361.493793	1072.51316	912.1902238	1066.99713
ENSG00000147813	NAPRT	chr8:143574785-143578649	-	protein_coding	1.437645825	0.291259587	4.935960535	7.97572E-07	7.39191E-06	Yes	OK	168.7368371	90.37899848	247.0946757	87.17236786	92.02916909	91.93545848	139.0124353	387.9975778	214.2740139
ENSG00000166106	ADAMTS15	chr11:130448974-130476641	+	protein_coding	1.435467198	0.273167154	5.254904105	1.48102E-07	1.52741E-06	Yes	OK	98.49256811	52.55649385	144.4286424	48.31239665	65.62079883	43.73628607	109.5528464	199.5416115	124.1914693
ENSG00000118242	MREG	chr2:215942805-216034096	-	protein_coding	1.433969413	0.217797374	6.583960974	4.58077E-11	7.04669E-10	Yes	OK	112.9991273	61.5241614	164.4740931	74.56913395	60.01902332	49.98432694	178.598758	142.5297225	172.2937989
ENSG00000152495	CAMK4	chr5:111223653-111494886	+	protein_coding	1.429246937	0.223851619	6.384796065	1.71626E-10	2.48896E-09	Yes	OK	175.6198135	95.65988583	255.5797411	101.8761408	97.6309446	87.47257214	336.9440485	180.5376485	249.2575264
ENSG00000076716	GPC4	chrX:133300103-133415490	-	protein_coding	1.426644793	0.110950196	12.85842512	7.71322E-38	4.70221E-36	Yes	OK	940.0628567	508.8648386	1371.260875	451.6158817	523.3658833	551.6127509	1302.666199	1445.884851	1365.231574
ENSG00000163618	CADPS	chr3:62398346-62875389	-	protein_coding	1.416217622	0.153360595	9.23456001	2.5935E-20	7.77577E-19	Yes	OK	269.1563504	147.2108083	391.1018925	150.1885374	135.2428659	156.2010217	436.3701612	354.7406426	382.1948738
ENSG00000137727	ARHGAP20	chr11:110577042-110713189	-	protein_coding	1.415572066	0.205351612	6.893406151	5.44721E-12	9.2149E-11	Yes	OK	124.6140573	68.39016665	180.837948	85.07182888	57.61826239	62.48040867	180.4399823	175.7866577	186.2872039
ENSG00000160539	PLPP7	chr9:131289459-131359022	+	protein_coding	1.415412356	0.213327511	6.634926515	3.24664E-11	5.06538E-10	Yes	OK	175.5805707	95.14839557	256.0127459	86.12209837	90.4286618	108.8944265	195.1697767	326.2346981	246.6337629
ENSG00000116132	PRRX1	chr1:170662728-170739419	+	protein_coding	1.415319237	0.101079178	14.00208496	1.51364E-44	1.13248E-42	Yes	OK	13182.22893	7190.641009	19173.81686	7191.195214	7207.08432	7173.643493	21676.73379	16036.17744	19808.53935
ENSG00000164530	PI16	chr6:36948263-36964837	+	protein_coding	1.414951109	0.109831794	12.88289167	5.61901E-38	3.46401E-36	Yes	OK	1914.241598	1043.186612	2785.296584	1033.46518	1033.927708	1062.166947	2410.16262	3168.910829	2776.816302
ENSG00000022267	FHL1	chrX:136146702-136211359	+	protein_coding	1.411676414	0.064362953	21.93305834	1.2569E-106	3.3915E-104	Yes	OK	7853.335098	4290.439091	11416.23111	4384.87513	4264.55167	4221.890472	11246.19808	11833.13429	11169.36094
ENSG00000169071	ROR2	chr9:91563091-91950162	-	protein_coding	1.411506635	0.222286448	6.349944624	2.15392E-10	3.0856E-09	Yes	OK	126.0443694	68.18294475	183.905794	57.76482208	81.62587171	65.15814047	155.5834541	226.4638924	169.6700355
ENSG00000187210	GCNT1	chr9:76419850-76507416	+	protein_coding	1.410775678	0.113830074	12.39369903	2.82696E-35	1.55106E-33	Yes	OK	829.8654236	454.2672254	1205.463622	454.7666902	461.7463527	446.2886334	1336.728848	1083.225891	1196.436127
ENSG00000132561	MATN2	chr8:97868840-98036716	+	protein_coding	1.408837103	0.105306696	13.37841901	8.08488E-41	5.43172E-39	Yes	OK	721.7407119	395.4701029	1048.011321	421.1580664	384.1217492	381.1304929	1066.068875	1021.463011	1056.502077
ENSG00000107362	ABHD17B	chr9:71862452-71910931	-	protein_coding	1.404807356	0.149157431	9.418286065	4.58511E-21	1.44028E-19	Yes	OK	436.685497	240.1188356	633.2521583	241.5619832	252.0798979	226.7146258	711.6331954	514.6906645	673.4326151
ENSG00000084710	EFR3B	chr2:25042130-25159137	+	protein_coding	1.403942672	0.194087122	7.23356944	4.70462E-13	8.91116E-12	Yes	OK	122.7203796	67.40414243	178.0366167	75.61940345	63.2200379	63.37298594	172.1544729	185.2886392	176.666738
ENSG00000109472	CPE	chr4:165361194-165498320	+	protein_coding	1.40240625	0.098523134	14.2342838	5.61351E-46	4.35841E-44	Yes	OK	1015.243762	557.842374	1472.645149	552.4417529	556.9765364	564.1088326	1558.596378	1377.787317	1481.551753
ENSG00000182378	PLCXD1	chrX:276322-303356	+	protein_coding	1.390430485	0.143785605	9.670164711	4.03728E-22	1.30451E-20	Yes	OK	346.6295867	190.6673245	502.5918489	181.6966222	191.260621	199.0447305	466.7503623	574.8698806	466.1553037
ENSG00000102468	HTR2A	chr13:46831550-46897076	-	protein_coding	1.387642233	0.194753635	7.125115977	1.03993E-12	1.91898E-11	Yes	OK	249.391882	138.4210539	360.36271	152.2890764	157.6499679	105.3241175	408.7517966	286.6431085	385.693225
ENSG00000099139	PCSK5	chr9:75890644-76362339	+	protein_coding	1.382822602	0.199957403	6.915585915	4.65934E-12	7.95567E-11	Yes	OK	122.8429051	68.53393101	177.1518793	76.66967294	58.41851603	70.51360408	177.6781458	163.117349	190.660143
ENSG00000072952	MRVI1	chr11:10573091-10693988	-	protein_coding	1.370996781	0.076580191	17.9027599	1.1222E-71	1.41003E-69	Yes	OK	2878.256593	1604.944439	4151.568747	1642.621486	1539.688012	1632.523821	4053.455316	4293.311973	4107.938952
ENSG00000259207	ITGB3	chr17:47253846-47311816	+	protein_coding	1.370589188	0.197820781	6.928438871	4.2551E-12	7.31095E-11	Yes	OK	154.8675434	86.72018531	223.0149014	90.32317634	98.43119824	71.40618134	232.9148751	186.8723028	249.2575264
ENSG00000242265	PEG10	chr7:94656325-94669695	+	protein_coding	1.37035892	0.080740368	16.97241352	1.31418E-64	1.45177E-62	Yes	OK	3380.305416	1885.804429	4874.806403	1858.977001	1844.58465	1953.851637	5206.061733	4787.415011	4630.942464
ENSG00000121297	TSHZ3	chr19:31274945-31349547	-	protein_coding	1.369890201	0.10974474	12.48251358	9.30035E-36	5.2247E-34	Yes	OK	990.7366576	552.3247265	1429.148589	588.1509157	545.7729854	523.0502783	1318.316605	1574.161601	1394.96756
ENSG00000106772	PRUNE2	chr9:76611376-76906087	-	protein_coding	1.367387615	0.085232598	16.0430123	6.39746E-58	6.2309E-56	Yes	OK	2939.279439	1642.688778	4235.870101	1616.364749	1638.11921	1673.582375	4595.695875	3965.493612	4146.420816
ENSG00000186854	TRABD2A	chr2:84821650-84907008	-	protein_coding	1.366700027	0.196274273	6.963215318	3.32593E-12	5.78121E-11	Yes	OK	171.639183	96.55762858	246.7207375	97.67506278	92.02916909	99.96865388	273.4218099	186.8723028	279.8680998
ENSG00000109846	CRYAB	chr11:111908565-111923722	-	protein_coding	1.364617157	0.068382948	19.95551824	1.34224E-88	2.48239E-86	Yes	OK	10059.5847	5626.293523	14492.87588	5582.182352	5785.033594	5511.664622	13840.48313	15304.52487	14333.61965
ENSG00000166922	SCG5	chr15:32641676-32697098	+	protein_coding	1.363346878	0.189545643	7.192710176	6.35177E-13	1.19078E-11	Yes	OK	185.6700165	104.6073367	266.7326962	111.3285662	101.6322128	100.8612311	310.2462961	207.4599294	282.4918632
ENSG00000102755	FLT1	chr13:28300344-28495145	-	protein_coding	1.362986966	0.202937685	6.716283222	1.86418E-11	2.99361E-10	Yes	OK	222.8627326	125.5288255	320.1966398	130.233417	116.0367784	130.3162809	388.4983292	224.8802288	347.2113613
ENSG00000204103	MAFB	chr20:40685848-40689240	-	protein_coding	1.360410483	0.150890945	9.015852342	1.95345E-19	5.54377E-18	Yes	OK	443.1161592	247.6478416	638.5844768	248.9138697	236.0748251	257.9548301	553.2879048	771.2441649	591.2213608
ENSG00000143816	WNT9A	chr1:227918656-227947898	-	protein_coding	1.358860847	0.196076009	6.930275934	4.20021E-12	7.22418E-11	Yes	OK	169.9088083	94.78932073	245.0282959	86.12209837	92.02916909	106.2166947	197.9316132	291.3940993	245.7591751
ENSG00000170485	NPAS2	chr2:100820152-100996829	+	protein_coding	1.358725738	0.142469636	9.536949567	1.47095E-21	4.67411E-20	Yes	OK	347.9956411	195.391681	500.5996013	185.8977001	174.4552944	225.8220485	518.304643	489.3520471	494.1421137
ENSG00000117597	UTP25	chr1:209828007-209857565	+	protein_coding	1.352527376	0.110560752	12.23334092	2.06277E-34	1.08131E-32	Yes	OK	1668.527976	939.7286111	2397.327341	913.7344583	961.1046267	944.3467482	2745.265445	2114.190883	2332.525694
ENSG00000146426	TIAM2	chr6:154832697-155257723	+	protein_coding	1.350488257	0.126140678	10.70620741	9.51817E-27	3.78428E-25	Yes	OK	417.8733619	235.9992965	599.7474273	254.1652171	220.8700058	232.9626666	628.7781015	574.8698806	595.5942998
ENSG00000143786	CNIH3	chr1:224434660-224740549	+	protein_coding	1.349608139	0.159889463	8.44088229	3.14951E-17	7.86661E-16	Yes	OK	327.3951884	184.8227184	469.9676584	201.6517425	144.8459096	207.9705032	443.7350585	487.7683836	478.3995331
ENSG00000102287	GABRE	chrX:151953124-151974680	-	protein_coding	1.345685766	0.155270864	8.666698538	4.44831E-18	1.17338E-16	Yes	OK	268.9094419	151.7750033	386.0438805	149.1382679	131.2415977	174.9451443	382.0540441	407.0015408	369.0760566

ENSG00000165030	NFIL3	chr9:91409045-91423862	-	protein_coding	1.345093304	0.108552442	12.39118419	2.91704E-35	1.59516E-33	Yes	OK	711.6021956	402.5508561	1020.653535	397.0018681	392.9245393	417.7261609	1070.671936	947.0308226	1044.257847
ENSG00000121068	TBX2	chr17:61399896-61409466	+	protein_coding	1.343513298	0.193327198	6.949427243	3.66772E-12	6.35481E-11	Yes	OK	194.8360277	109.4745611	280.1974943	89.27290685	108.8344956	130.3162809	239.3591602	330.9856888	270.2476338
ENSG00000081189	MEF2C	chr5:88717117-88904257	-	protein_coding	1.342588305	0.159204071	8.433127992	3.36546E-17	8.39326E-16	Yes	OK	584.9667242	331.7749797	838.1584687	332.9354291	324.1027259	338.2867841	985.9756174	630.298106	898.2016827
ENSG00000013297	CLDN11	chr3:170418865-170454733	+	protein_coding	1.333668634	0.078191759	17.05638364	3.13364E-65	3.53286E-63	Yes	OK	5605.156107	3183.60901	8026.703203	3221.176533	3114.587183	3215.063315	8181.480217	8515.359086	7383.270307
ENSG00000137872	SEMA6D	chr15:47184101-47774223	+	protein_coding	1.319979014	0.238903753	5.525149755	3.29205E-08	3.68369E-07	Yes	OK	106.9506456	60.60765926	153.293632	46.21185766	62.41978425	73.19133588	125.203253	186.8723028	147.8053402
ENSG00000168214	RBPJ	chr4:26163455-26435131	+	protein_coding	1.317004621	0.133865726	9.838251041	7.70384E-23	2.58259E-21	Yes	OK	3282.461129	1881.38739	4683.534867	1921.993171	1971.824979	1750.34402	5504.340072	3617.087623	4929.176907
ENSG00000181751	C5orf30	chr5:103258702-103278660	+	protein_coding	1.316518902	0.114722816	11.47565014	1.74858E-30	8.0621E-29	Yes	OK	1795.969024	1029.52783	2562.410217	928.4382312	1066.738108	1093.407152	2798.66095	2204.459707	2684.109994
ENSG00000082397	EPB41L3	chr18:5392381-5630700	-	protein_coding	1.313889788	0.087893019	14.94873879	1.58727E-50	1.33298E-48	Yes	OK	2348.412754	1348.229083	3348.596424	1361.149262	1326.820542	1356.717445	3624.450052	3111.898941	3309.44028
ENSG00000196611	MMP1	chr11:102789920-102798160	-	protein_coding	1.313295943	0.066490136	19.75174097	7.74996E-87	1.3151E-84	Yes	OK	7374.083949	4231.974778	10516.19312	4201.077969	4377.387434	4117.458932	10883.47689	10247.88705	10417.21543
ENSG00000141052	MYOCD	chr17:12665890-12768949	+	protein_coding	1.312494746	0.197534744	6.644374141	3.04508E-11	4.76445E-10	Yes	OK	222.3921365	127.8390583	316.9452147	97.67506278	129.6410904	156.2010217	347.0707822	256.5535004	347.2113613
ENSG00000147119	CHST7	chrX:46573784-46598408	+	protein_coding	1.310047095	0.207094943	6.325828505	2.51877E-10	3.59264E-09	Yes	OK	203.252067	115.9750914	290.5290425	102.9264102	120.0380466	124.9608173	233.8354872	383.2465871	254.5050532
ENSG00000116729	WLS	chr1:68098473-68233120	-	protein_coding	1.293963702	0.100660409	12.85474312	8.08941E-38	4.91335E-36	Yes	OK	809.7570487	468.9498852	1150.564212	464.2191156	494.5567521	448.0737879	1155.368254	1149.739761	1146.584621
ENSG00000141338	ABCA8	chr17:68867292-68955392	-	protein_coding	1.288662276	0.240936094	5.348564663	8.86545E-08	9.42089E-07	Yes	OK	120.2791105	69.8035574	170.7546636	82.97128989	80.02536442	46.41401787	140.8536596	186.8723028	184.5380283
ENSG00000095637	SORBS1	chr10:95311771-95561414	-	protein_coding	1.288580154	0.231593578	5.563971863	2.63702E-08	2.99141E-07	Yes	OK	95.22643617	55.21086325	135.2420091	63.01616954	54.41724781	48.19917241	109.5528464	153.6153675	142.5578133
ENSG00000162772	ATF3	chr1:212565334-212620777	+	protein_coding	1.287402822	0.246278745	5.227421575	1.7189E-07	1.75734E-06	Yes	OK	129.4342248	74.6108056	184.257644	45.16158817	99.23145189	79.43937674	179.5193701	202.7089386	170.5446233
ENSG00000249992	TMEM158	chr3:45224466-45226278	-	protein_coding	1.286768402	0.184631379	6.969391724	3.18314E-12	5.56205E-11	Yes	OK	557.3432608	323.2208595	791.4656622	343.438124	304.8966385	321.327816	629.6987137	1072.140246	672.5580273
ENSG00000140323	DISP2	chr15:40358235-40378639	+	protein_coding	1.283055847	0.268365536	4.781000826	1.74425E-06	1.5304E-05	Yes	OK	111.5496083	64.51132279	158.5878938	50.41293563	59.21876967	83.90226308	108.6322342	202.7089386	164.4225086
ENSG00000000971	CFH	chr1:196651878-196747504	+	protein_coding	1.272229224	0.14312921	8.888676336	6.18412E-19	1.68807E-17	Yes	OK	540.5823519	317.450053	763.7146508	339.237046	325.7032332	287.4098799	867.2166495	635.0490968	788.8782063
ENSG00000011332	DPF1	chr19:38211006-38229714	-	protein_coding	1.263170216	0.231754436	5.450468335	5.02373E-08	5.52745E-07	Yes	OK	113.5468896	67.16659289	159.9272063	91.37344583	51.21623323	58.91009961	162.0277392	164.7010126	153.0528671
ENSG00000204291	COL15A1	chr9:98943179-99070792	+	protein_coding	1.259677345	0.101557076	12.40363938	2.49719E-35	1.38396E-33	Yes	OK	759.8131118	447.6898723	1071.936351	436.9121088	462.5466064	443.6109016	1056.862753	1049.968956	1108.977345
ENSG00000162745	OLFML2B	chr1:161983192-162023854	-	protein_coding	1.258654283	0.0996685	12.62840604	1.4723E-36	8.47347E-35	Yes	OK	1794.817338	1056.816529	2532.818148	1029.264102	1053.133796	1088.051688	2327.307526	2828.423159	2442.723758
ENSG00000139998	RAB15	chr14:64945814-64973226	-	protein_coding	1.256520951	0.114127956	11.00975599	3.42931E-28	1.41825E-26	Yes	OK	641.0236294	377.831844	904.2154148	393.8510596	381.7209883	357.923484	843.2807335	975.5367671	893.8287437
ENSG00000179104	TMTC2	chr12:82686880-83134870	+	protein_coding	1.251164111	0.158988285	7.869536477	3.55958E-15	7.78096E-14	Yes	OK	201.3027792	119.1426353	283.4629231	109.2280272	123.2390612	124.9608173	285.3897679	267.6391455	297.359856
ENSG00000153790	C7orf31	chr7:25134697-25180356	-	protein_coding	1.243954991	0.255032112	4.877640636	1.07362E-06	9.76224E-06	Yes	OK	97.16359801	57.32724117	136.9999549	48.31239665	80.82561807	42.84370881	125.203253	144.113386	141.6832255
ENSG00000146374	RSPO3	chr6:127118604-127197765	+	protein_coding	1.243205366	0.182185256	6.823852786	8.86307E-12	1.46472E-10	Yes	OK	151.9813199	90.70336793	213.2592719	92.42371532	90.4286618	89.25772668	235.6767115	183.7049756	220.3961286
ENSG00000149218	ENDOD1	chr11:95089810-95132645	+	protein_coding	1.235208858	0.093287265	13.24091624	5.09238E-40	3.33946E-38	Yes	OK	1819.867907	1086.016506	2553.719309	1090.179733	1007.519338	1160.350447	2672.537084	2410.335973	2578.284869
ENSG00000106034	CPED1	chr7:120988677-121297444	+	protein_coding	1.231195114	0.136862791	8.995835237	2.34443E-19	6.60775E-18	Yes	OK	1348.36714	806.5614938	1890.172787	787.7021192	840.2663265	791.7160356	2159.756114	1471.223469	2039.538777
ENSG00000147224	PRPS1	chrX:107628424-107651026	+	protein_coding	1.224192112	0.07213543	16.97074664	1.35202E-64	1.48362E-62	Yes	OK	4895.653221	2934.599914	6856.706529	3042.630719	2974.542796	2786.626227	6882.496467	7034.633636	6652.989484
ENSG00000110660	SLC35F2	chr11:107790991-107928293	-	protein_coding	1.222643923	0.142830047	8.560131057	1.12739E-17	2.89951E-16	Yes	OK	383.6698373	230.4933286	536.846346	205.8528205	239.2758396	246.3513256	582.7474938	467.180757	560.6107874
ENSG00000131773	KHDRBS3	chr8:135457457-135656722	+	protein_coding	1.221317133	0.125632055	9.721381452	2.44442E-22	7.96737E-21	Yes	OK	368.8190233	221.6389864	515.9990602	222.6571324	212.8674694	229.3923576	527.5107645	503.6050194	516.8813968
ENSG00000132170	PPARG	chr3:12287368-12434356	+	protein_coding	1.217935397	0.180357384	6.75290011	1.44919E-11	2.35011E-10	Yes	OK	200.7545193	120.786499	280.7225397	128.1328781	136.0431195	98.18349935	316.6905811	269.2228091	256.2542289
ENSG00000171444	MCC	chr5:113022099-113488830	-	protein_coding	1.210456904	0.123745816	9.781800692	1.34791E-22	4.45513E-21	Yes	OK	437.8292055	264.5721086	611.0863024	262.5673731	262.4831953	268.6657573	661.9201391	574.8698806	596.4688877
ENSG00000169258	GPRIN1	chr5:176595802-176610133	-	protein_coding	1.209242973	0.175750986	6.880433524	5.96707E-12	1.0053E-10	Yes	OK	316.6457379	190.1726852	443.1187906	162.7917713	210.4667084	197.259576	365.4830253	543.196609	420.6767375
ENSG00000049323	LTBP1	chr2:32946972-33399509	+	protein_coding	1.20272112	0.066742231	18.02039136	1.34787E-72	1.73327E-70	Yes	OK	5405.843475	3273.638273	7538.048677	3247.43327	3353.062769	3220.418779	7494.703549	7761.53522	7357.907261
ENSG00000102802	MEDAG	chr13:30906191-30925572	+	protein_coding	1.200585054	0.093079882	12.89843767	4.59315E-38	2.85295E-36	Yes	OK	984.1754184	596.8552085	1371.495628	582.8995682	603.3912478	604.2748096	1395.648026	1341.363055	1377.475804
ENSG00000120549	KIAA1217	chr10:23694746-24547848	+	protein_coding	1.19971272	0.106253604	11.29103087	1.45322E-29	6.41288E-28	Yes	OK	936.6805543	567.217414	1306.143695	533.5369021	582.584653	585.530687	1311.87232	1403.125935	1203.432829
ENSG00000110092	CCND1	chr11:69641087-69654474	+	protein_coding	1.199057508	0.053537071	22.39677072	4.2316E-111	1.2899E-108	Yes	OK	34827.84916	21133.06917	48522.62915	20883.55858	21164.30813	21351.3408	48148.93629	49628.84936	47790.1018
ENSG00000147231	CXorf57	chrX:106611930-106679442	+	protein_coding	1.19802294	0.222436832	5.385901821	7.20823E-08	7.74967E-07	Yes	OK	93.39736243	56.6471626	130.1475623	63.01616954	48.01521865	58.91009961	119.6795801	145.6970496	125.0660571
ENSG00000074416	MGLL	chr3:127689062-127823250	-	protein_coding	1.194288327	0.09018122	13.24320433	4.93955E-40	3.2522E-38	Yes	OK	5446.690886	3311.397193	7581.984578	3302.047284	3277.838927	3354.305369	6813.450555	8475.767496	7456.735683
ENSG00000122861	PLAU	chr10:73909177-73917497	+	protein_coding	1.192896906	0.135874516	8.779401297	1.64347E-18	4.42019E-17	Yes	OK	627.0723189	380.8351799	873.3094579	400.1526766	373.7184519	368.6344112	833.1539998	1029.381329	757.393045
ENSG00000160284	SPATC1L	chr21:46161148-46184476	-	protein_coding	1.192474966	0.211062685	5.649861634	1.60577E-08	1.87853E-07	Yes	OK	110.3475067	66.8376188	153.8573946	56.71455258	68.82181341	74.97649041	133.4887624	166.2846762	161.7987452
ENSG00000129596	CDO1	chr5:115804733-115816954	-	protein_coding	1.190968458	0.214039271	5.56425208	2.63279E-08	2.98867E-07	Yes	OK	101.8938835	62.46817942	141.3195877	66.16697081	63.2200379	58.01752234	156.5040663	118.7747687	148.679928
ENSG00000183963	SMTN	chr22:31064105-31104757	+	protein_coding	1.190635094	0.125081145	9.518901475	1.75019E-21	5.54573E-20	Yes	OK	5736.952975	3494.01087	7979.895079	3458.537438	3405.87951	3617.615662	6632.089961	9870.975113	7436.620164
ENSG00000162757	C1orf74	chr1:209779208-209784559	-	protein_coding	1.185402314	0.229325004	5.169093177	2.35233E-07	2.36093E-06	Yes	OK	104.9116255	63.76767965	146.0555714	46.21185766	80.82561807	64.26556321	154.6628419	140.9460589	142.5578133
ENSG00000179750	APOBEC3B	chr22:38982347-38992804	+	protein_coding	1.176870288	0.192360436	6.118047507	9.47288E-10	1.28121E-08	Yes	OK	167.8011685	102.4236818	233.1786552	87.17236786	96.03043731	124.0682401	231.9942629	262.8881548	204.653548
ENSG00000196196	HRCT1	chr9:35906192-35907141	+	protein_coding	1.176332536	0.261761221	4.493914453	6.99257E-06	5.56566E-05	Yes	OK	97.79496954	59.55199739	136.0379417	55.66428309	48.01521865	74.97649041	108.6322342	180.5376485	118.9439424
ENSG00000106688	SLC1A1	chr9:4490444-4587469	+	protein_coding	1.174223877	0.119287795	9.843621256	7.30337E-23	2.46339E-21	Yes	OK	559.3498388	344.263204	774.4364736	351.8402799	348.9105889	332.0387432	800.0119622	696.8119765	826.4854822
ENSG00000069702	TGFBR3	chr1:91680343-91906335	-	protein_coding	1.174144643	0.091420169	12.84338743	9.36838E-38	5.64848E-36	Yes	OK	1709.583503	1050.645542	2368.521464	1033.46518	1087.544703	1030.926743	2481.970368	2179.12109	2444.472934
ENSG00000144843	ADPRH	chr3:119579268-119589945	+	protein_coding	1.17164834	0.187781552	6.239421969	4.39191E-10	6.09535E-09	Yes	OK	211.4103753	130.1281796	292.69251	107.1274882	152.0481924	131.2088582	296.4371137	242.3005282	339.340071
ENSG00000006453	BAIAP2L1	chr7:98291651-98401068	-	protein_coding	1.169662123	0.157591136	7.422131406	1.1525E-13	2.28557E-12	Yes	OK	276.1649149	169.6017332	382.7280966	187.9982391	159.2504752	161.5564853	360.8799645	441.8421396	345.4621857
ENSG00000064763	FAR2	chr12:29149103-29340980	+	protein_coding	1.162771109	0.180910375	6.427332385	1.29863E-10	1.90512E-09	Yes	OK	144.4006711	89.02221822	199.7791239	85.07182888	93.62967638	88.36514941	215.4232441	201.125275	182.7888527
ENSG00000175130	MARCKSL1	chr1:32333832-32336379	-	protein_coding	1.162372162	0.156647299	7.420314089	1.16843E-13	2.31436E-12	Yes	OK	924.4782103	569.7794598	1279.176961	548.240675	573.7818629	587.3158415	1020.038267	1635.924481	1181.568134
ENSG00000152127	MGAT5	chr2:134119983-134454621	+	protein_coding	1.158593153	0.08552918	13.54617406	8.34686E-42	5.79702E-40	Yes	OK	2052.947245	1269.855116	2836.039374	1258.222852	1211.584017	1339.758477	2865.865637	2934.528619	2707.723865
ENSG00000197461	PDGFA	chr7:497258-520296	-	protein_coding	1.153707354	0.20602929	5.599724946	2.14692E-08	2.46949E-07	Yes	OK	188.7206322	116.5581235	260.8831408	127.0826086	97.6309446	124.9608173	227.3912022	335.7366796	219.5215408
ENSG00000173917	HOXB2	chr17:48540894-48544989	-	protein_coding	1.152248394	0.153660014	7.4986873	6.44601E-14	1.30346E-12	Yes	OK	585.5665158	362.4212601	808.7117774	350.7900104	347.3100816	389.1636883	669.2850363	997.7080573	759.1422207
ENSG00000198805	PNP	chr14:20468954-20477094	+	protein_coding	1.149850777	0.116150407	9.899670694	4.17648E-23	1.42035E-21	Yes	OK	541.2873434	335.8855295	746.6891573	312.9803087	330.5047551	364.1715248	739.25156	769.6605013	731.1554107
ENSG00000037965	HOXC8	chr12:54009106-54012362	+	protein_coding	1.146162245	0.088865528	12.89771492	4.63642E-38	2.86901E-36	Yes	OK	1253.777579	780.7895117	1726.765647	779.2999633	795.4521224	767.6164494	1774.940234	1659.679435	1745.677272
ENSG00000154822	PLCL2	chr3:16802651-17090594	+	protein_coding	1.143860657	0.172351844	6.636776427	3.20617E-11	5.00698E-10	Yes	OK	173.7885632	108.4318194	239.1453071	100.8258713	120.0380466	104.4315402	245.8034452	215.3782473	256.2542289
ENSG00000176788	BASP1	chr5:17065598-17276843	+	protein_coding	1.142712796	0.064905279	17.60585282	2.22136E-69	2.66888E-67	Yes	OK	7488.512129	4667.784825	10309.23943	4698.905709	4647.873166	4656.575601	10174.60553	10753.07573	10000.03704
ENSG00000135414	GDF11	chr12:55743280-55757278	+	protein_coding	1.142674441	0.133251944	8.575298249	9.8835E-18	2.55388E-16	Yes	OK	819.089455	509.6179179	1128.560992	506.2298953	489.7552303	532.8686283	986.8962296	1349.281373	1049.505374
ENSG00000161638	ITGA5	chr12:54395261-54419460	-	protein_coding	1.142185117	0.087519537	13.05063029	6.30238E-39	4.0053E-37	Yes	OK	26061.47329	16250.33302	35872.61355	16011.35841	16402.79895	16336.84171	31848.57748	40533.86941	35235.39376
ENSG00000131459	GFPT2	chr5:180300690-180353387	-	protein_coding	1.136055903	0.121541915	9.347029788	9.01436E-21	2.7838E-19	Yes	OK	777.0647866	484.7680599	1069.361513	436.9121088	527.3671516	490.0249195	980.4519445	1187.747687	1039.884908
ENSG00000136856	SLC2A8	chr9:127397138-127408424	+	protein_coding	1.133622669	0.215594003	5.258136366	1.45523E-07	1.50553E-06	Yes	OK	102.7261692	64.1583414	141.2939043	72.46859497	58.41851603	61.58783141	125.203253	163.117349	135.5611108
ENSG00000197977	ELOVL2	chr6:10980759-11044314	-	protein_coding	1.132655598	0.25297769	4.477294409	7.5595E-06	5.95778E-05	Yes	OK	115.5093291	72.81228569	158.2063726	76.66967294	86.42739358	55.33979054	170.3132486	109.2727872	195.033082
ENSG00000122547	EEPD1	chr7:36153149-36301543	+	protein_coding	1.128673418	0.200566708	5.62742157	1.82923E-08	2.12485E-07	Yes	OK	204.4048061	127.4629141	281.3466981	126.0323391	137.6436268	118.7127765	223.7087535	365.8262877	254.5050532
ENSG00000154928	EPHB1	chr3:134597801-135260467	+	protein_coding	1.125994936	0.162047729	6.948538808	3.69089E-12	6.38823E-11	Yes	OK	244.5354644	154.2049659	334.8659629	164.8923103	127.2403294	170.482258	355.3562916	315.149053	334.0925441
ENSG00000260027	HOXB7	chr17:48607227-48633572	-	protein_coding	1.124915512	0.148952986	7.552151484	4.28126E-14	8.77579E-13	Yes	OK	302.838037	189.9643374	415.7117367	194.2998561	187.2593528	188.3338033	357.1975159	464.0134298	425.9242644
ENSG00000150967	ABCB9	chr12:122920951-122981649	-	protein_coding	1.124842933	0.250696989	4.486862549	7.22797E-06	5.73084E-05	Yes	OK	159.4134269	99.59960071	219.2272531	93.47398481	84.82688629	120.497931	155.5834541	308.8143987	193.2839064
ENSG00000103034	NDRG4	chr16:58462846-58513628	+	protein_coding	1.122572497	0.220404811	5.093230467	3.52013E-07	3.43866E-06	Yes	OK	160.0505166	99.97541518	220.125618	98.72533228	112.8357638	88.36514941	173.075085	291.3940993	195.9076698
ENSG00000138821	SLC39A8	chr4:102251041-102431258	-	protein_coding	1.122464442	0.180911931	6.204479927	5.48781E-10	7.57796E-09	Yes	OK	175.8165803	110.2248356	241.4083251	90.32317634	121.6385539	118.7127765	258.6920154	248.6351825	216.8977773
ENSG00000128274	A4GALT	chr22:42692112-42721298	-	protein_coding	1.122330745	0.154558791	7.26151349	3.82783E-13	7.31778E-12	Yes	OK	1098.007991	690.08232	1505.933662	675.3232835	674.6138221	720.3098543	1182.986618	1916.232935	1418.581431
ENSG00000172301	COPRS	chr17:31851864-31859337	-	protein_coding	1.122181946	0.086906822	12.91247246	3.82809E-38	2.38676E-36	Yes	OK	1619.059738	1019.261907	2218.857568	1016.660869	1077.141405	963.9834481	2218.675292	2190.206735	2247.690676
ENSG00000135318	NT5E	chr6:85449584-85495791	+	protein_coding	1.12194912	0.100420543	11.172506	5.559E-29	2.39532E-27	Yes	OK	10271.83636	6469.081837	14074.59088	6412.94552	6598.091297	6396.208694	15599.77296	11776.1224	14847.87728
ENSG00000198353	HOXC4	chr12:54016931-54056030	+	protein_coding	1.117968327	0.129147868	8.656498509	4.86477E-18	1.27914E-16	Yes	OK	450.0438333	283.3069122	616.7807543	288.8241104	273.6867463	287.4098799	559.7321899	688.8936586	601.7164145
ENSG00000004660	CAMKK1	chr17:3860315-3894891	-	protein_coding	1.115155656	0.176991094	6.30063148	2.96435E-10	4.20269E-09	Yes	OK	405.4129856	254.9428976	555.8830735	223.7074019	268.8852245	272.2360664	453.8617922	712.6486123	501.1388162
ENSG00000162631	NTNG1	chr1:107140007-107483458	+	protein_coding	1.113903074	0.094499842	11.78735386	4.53569E-32	2.24196E-30	Yes	OK	1500.195725	949.372869	2051.018582	965.1976634	924.2929591	958.6279845	2149.629381	1870.306691	2133.119673
ENSG00000166949	SMAD3	chr15:67063763-67195195	+	protein_coding	1.112094617	0.07148921	15.5561185	1.44607E-54	1.32235E-52	Yes	OK	3037.3355	1921.520446	4153.150555	1946.149369	1872.593528	1945.818442	4112.374494	4184.039186	4163.037984
ENSG00000189410	SH2D5	chr1:20719732-20732837	-	protein_coding	1.108598143	0.146825812	7.550430891	4.33821E-14	8.88145E-13	Yes	OK	323.823208	204.4957051	443.1507108	197.4506646	208.0659475	207.9705032	382.9746563	500.4376922	446.039784
ENSG00000240764	PCDHGC5	chr5:141489121-141512979	+	protein_coding	1.104958365	0.242531026	4.555946443	5.21502E-06	4.21815E-05	Yes	OK	91.82672245	58.73130444	124.9221405	55.66428309	61.61953061	58.91009961	141.7742718	88.68516065	144.3069889
ENSG00000187678	SPRY4	chr5:142310427-142326455	-	protein_coding	1.10402833	0.094978073	11.62403385	3.11093E-31	1.49725E-29	Yes	OK	931.2759083	590.9443416	1271.607475	590.2514547	604.1915014	578.3900689	1265.841712	1309.689783	1239.290929

ENSG00000130751	NPAS1	chr19:47019820-47045775	+	protein_coding	1.103827809	0.20514887	5.380618509	7.42304E-08	7.9702E-07	Yes	OK	456.7486162	288.9848411	624.5123914	266.768451	321.701965	278.4841072	446.4968949	864.6803163	562.359963
ENSG00000172731	LRRC20	chr10:70298970-70382650	-	protein_coding	1.101719604	0.207370607	5.312805039	1.07951E-07	1.13452E-06	Yes	OK	139.1350152	88.33450487	189.9355256	101.8761408	79.22511078	83.90226308	150.9803933	218.5455744	200.2806089
ENSG00000113361	CDH6	chr5:31193750-31329146	+	protein_coding	1.101286333	0.14523619	7.582726655	3.38367E-14	6.97062E-13	Yes	OK	487.0881969	310.047398	664.1289959	283.5727629	329.7045014	316.8649297	783.4409434	574.8698806	634.0761636
ENSG00000118503	TNFAIP3	chr6:137867188-137883312	+	protein_coding	1.098542112	0.146963155	7.47494918	7.72337E-14	1.55222E-12	Yes	OK	304.8136959	194.0420903	415.5853015	215.3052459	170.4540262	196.3669987	393.10139	451.3441211	402.3103934
ENSG00000163637	PRICKLE2	chr3:64092242-64445476	-	protein_coding	1.098463741	0.129615524	8.474785305	2.35513E-17	5.95476E-16	Yes	OK	575.9095613	367.3481311	784.4709916	378.0970172	378.5199737	345.4274022	894.8350141	696.8119765	761.7659841
ENSG00000182704	TSKU	chr11:76782251-76798154	+	protein_coding	1.097816138	0.180297308	6.088921404	1.13674E-09	1.5212E-08	Yes	OK	614.8728496	390.5166236	839.2290756	397.0018681	427.335446	347.2125568	663.7613634	1114.899162	739.026701
ENSG00000145555	MYO10	chr5:16661914-16936276	-	protein_coding	1.093603238	0.068746574	15.90774884	5.59908E-57	5.38953E-55	Yes	OK	4204.387585	2683.54292	5725.23225	2783.214155	2620.030431	2647.384173	5751.064129	5724.943852	5699.68877
ENSG00000101311	FERMT1	chr20:6074845-6123544	-	protein_coding	1.0934882	0.182090575	6.005188366	1.9111E-09	2.49656E-08	Yes	OK	144.0096732	92.20896672	195.8103797	90.32317634	87.22764722	99.07607661	193.3285524	178.9539849	215.1486017
ENSG00000157827	FMNL2	chr2:152335237-152649834	+	protein_coding	1.0926578	0.114789512	9.518794706	1.75199E-21	5.54573E-20	Yes	OK	1572.411541	1005.220116	2139.602965	1011.409521	986.7127434	1017.538084	2275.753246	1789.539849	2353.515802
ENSG00000183287	CCBE1	chr18:59430940-59697380	-	protein_coding	1.092372705	0.088567618	12.33377091	5.95913E-35	3.19502E-33	Yes	OK	1866.818302	1192.967439	2540.669164	1179.45264	1158.767277	1240.682401	2609.014846	2361.242402	2651.750245
ENSG00000088538	DOCK3	chr3:50675241-51384198	+	protein_coding	1.09122325	0.199159969	5.479129444	4.27424E-08	4.73127E-07	Yes	OK	114.8781057	73.47528641	156.2809251	73.51886446	72.82308163	74.08391314	175.8369215	150.4480404	142.5578133
ENSG00000138316	ADAMTS14	chr10:70672803-70762441	+	protein_coding	1.09049831	0.197309313	5.526846611	3.26038E-08	3.65322E-07	Yes	OK	218.9392868	139.2879066	298.5906671	148.0879984	129.6410904	140.1346309	231.0736508	384.8302507	279.8680998
ENSG00000138639	ARHGAP24	chr4:85475114-86002670	+	protein_coding	1.090497514	0.0986881	11.04993933	2.19363E-28	9.16425E-27	Yes	OK	955.034138	610.6384329	1299.429843	603.9049581	581.7843994	646.2259411	1351.458643	1271.681857	1275.14903
ENSG00000142634	EFHD2	chr1:15409895-15430343	+	protein_coding	1.088754667	0.138652001	7.85242666	4.08064E-15	8.88456E-14	Yes	OK	1546.970978	988.1845436	2105.757411	982.0019753	965.9061486	1016.645507	1744.560033	2624.130557	1948.581645
ENSG00000028137	TNFRSF1B	chr1:12167003-12209228	+	protein_coding	1.078475248	0.257307795	4.191381948	2.7726E-05	0.000196288	Yes	OK	129.6473688	82.70352768	176.59121	86.12209837	73.62333527	88.36514941	126.1238652	254.9698369	148.679928
ENSG00000006118	TMEM132A	chr11:60924463-60937159	+	protein_coding	1.074930501	0.181744551	5.914512964	3.32859E-09	4.22752E-08	Yes	OK	293.3157387	188.8042211	397.8272562	236.3106358	156.0494606	174.052567	354.4356795	465.5970934	373.4489956
ENSG00000073150	PANX2	chr22:50170731-50180294	+	protein_coding	1.071674952	0.249716166	4.291572192	1.77413E-05	0.000130653	Yes	OK	102.403711	65.75623655	139.0511855	70.36805598	52.81674052	74.08391314	101.267337	174.2029941	141.6832255
ENSG00000114790	ARHGEF26	chr3:154121003-154257827	+	protein_coding	1.069705607	0.284266111	3.763043031	0.000167858	0.000999258	Yes	OK	100.4555962	65.42647897	135.4847133	86.12209837	66.42105247	43.73628607	166.6308	90.26882423	149.5545158
ENSG00000081803	CADPS2	chr7:122318425-122886759	-	protein_coding	1.069020995	0.17683429	6.045326348	1.49108E-09	1.96818E-08	Yes	OK	189.9869858	122.3654394	257.6085323	110.2782967	133.6423586	123.1756628	291.834053	259.7208276	221.2707164
ENSG00000162337	LRP5	chr11:68312609-68449275	+	protein_coding	1.067563693	0.131726086	8.104421285	5.29972E-16	1.20822E-14	Yes	OK	2225.58436	1436.336702	3014.832018	1368.501148	1397.242863	1543.266094	2555.619341	3704.189121	2784.687593
ENSG00000099953	MMP11	chr22:23768226-23784316	+	protein_coding	1.067024219	0.208999114	5.10540068	3.30094E-07	3.23606E-06	Yes	OK	149.3427045	95.83835558	202.8470535	88.22263735	98.43119824	100.8612311	155.5834541	251.8025097	201.1551967
ENSG00000106991	ENG	chr9:127815012-127854756	-	protein_coding	1.066044199	0.125552683	8.490811764	2.05199E-17	5.20427E-16	Yes	OK	11112.22499	7182.128886	15042.3211	6981.141315	7224.6899	7340.555442	12283.72798	18763.24613	14079.98918
ENSG00000134769	DTNA	chr18:34493290-34891844	+	protein_coding	1.059589275	0.275202087	3.850222519	0.000118011	0.000727511	Yes	OK	84.17245293	53.91215077	114.4327551	40.9605102	67.22130612	53.55463601	110.4734585	148.8643768	83.96042993
ENSG00000176170	SPHK1	chr17:76376584-76387860	+	protein_coding	1.056521972	0.162645799	6.495845447	8.25684E-11	1.2423E-09	Yes	OK	2420.421118	1570.447833	3270.394402	1477.729176	1494.873807	1738.740516	2503.144448	4326.568909	2981.46985
ENSG00000123191	ATP7B	chr13:51930436-52012125	-	protein_coding	1.054380646	0.16222044	6.499678126	8.0492E-11	1.21328E-09	Yes	OK	201.9460462	130.7423245	273.1497679	114.4793747	145.6461633	132.1014355	266.0569126	286.6431085	266.7492826
ENSG00000181744	C3orf58	chr3:143971798-144048719	+	protein_coding	1.053531916	0.157762754	6.677950849	2.42306E-11	3.84235E-10	Yes	OK	413.0963405	269.5914102	556.6012708	273.070068	261.6829417	274.0212209	649.0315689	433.9238217	586.8484217
ENSG00000178031	ADAMTSL1	chr9:17906563-18910950	+	protein_coding	1.047024553	0.079028856	13.2486361	4.59478E-40	3.03735E-38	Yes	OK	10268.22014	6695.73777	13840.70251	6567.335135	6870.177536	6649.700637	12779.93793	15149.32583	13592.84377
ENSG00000177508	IRX3	chr16:54283304-54286763	-	protein_coding	1.04655859	0.161141868	6.494641058	8.32316E-11	1.24885E-09	Yes	OK	724.7662043	471.8859372	977.6464714	491.5261224	430.5364606	493.5952285	808.2974716	1252.677894	871.9640484
ENSG00000256235	SMIM3	chr5:150777946-150796734	+	protein_coding	1.045895553	0.106916083	9.782396841	1.33999E-22	4.43787E-21	Yes	OK	840.1746442	547.439753	1132.909535	518.8331292	559.3772973	564.1088326	1043.974183	1209.918977	1144.835446
ENSG00000140526	ABHD2	chr15:89087459-89202360	+	protein_coding	1.045893342	0.075604665	13.83371448	1.59564E-43	1.15194E-41	Yes	OK	3770.764971	2459.897744	5081.632199	2300.090188	2553.609379	2525.993665	5046.795831	5134.237336	5063.86343
ENSG00000160233	LRRC3	chr21:44455486-44462196	+	protein_coding	1.044808351	0.217605465	4.801388385	1.57569E-06	1.3944E-05	Yes	OK	123.0581097	79.82587482	166.2903447	81.9210204	88.82815451	68.72844954	133.4887624	202.7089386	162.673333
ENSG00000176641	RNF152	chr18:61808063-61894247	-	protein_coding	1.042006614	0.128382435	8.11642662	4.80111E-16	1.09911E-14	Yes	OK	368.7392327	240.8834832	496.5949822	220.5565934	251.2796443	250.814212	487.9244419	489.3520471	512.5084577
ENSG00000198598	MMP17	chr12:131828393-131851783	+	protein_coding	1.039974284	0.152951253	6.799383856	1.05068E-11	1.72596E-10	Yes	OK	851.1598253	555.819116	1146.500535	541.939058	611.3937842	514.1245057	918.7709302	1412.627916	1108.102758
ENSG00000187583	PLEKHN1	chr1:966497-975865	+	protein_coding	1.037639041	0.243400245	4.263097767	2.01612E-05	0.000146191	Yes	OK	103.4063	67.29531034	139.5172897	70.36805598	82.42612536	49.09174967	133.4887624	167.8683398	117.1947668
ENSG00000153721	CNKSR3	chr6:154387504-154510659	-	protein_coding	1.036740481	0.126214411	8.214121293	2.13723E-16	5.03274E-15	Yes	OK	430.8252424	282.2803433	579.3701415	254.1652171	301.6956239	290.980189	598.3979004	551.1149269	588.5975973
ENSG00000164620	RELL2	chr5:141636950-141641077	+	protein_coding	1.036609839	0.241771397	4.287561942	1.80645E-05	0.000132564	Yes	OK	80.19565822	52.30232026	108.0889962	58.81509157	47.21496501	50.87690421	95.74366406	131.4440774	97.07924711
ENSG00000228300	C19orf24	chr19:1275438-1279249	+	protein_coding	1.035627335	0.395811499	2.616466015	0.008884521	0.032284814	Yes	OK	670.7722997	439.4920194	902.05258	462.1185766	437.7387434	418.6187381	580.9062695	1414.21158	711.039891
ENSG00000065882	TBC1D1	chr4:37891087-38139175	+	protein_coding	1.034307623	0.096222844	10.74908596	5.98518E-27	2.38538E-25	Yes	OK	1825.880144	1197.197492	2454.562796	1216.212072	1127.557385	1247.823019	2346.640382	2690.644427	2326.403579
ENSG00000157557	ETS2	chr21:38805307-38824955	+	protein_coding	1.032402624	0.090457843	11.41308028	3.5976E-30	1.63581E-28	Yes	OK	1033.686851	678.9713237	1388.402378	675.3232835	685.0171195	676.5735682	1399.330475	1368.285336	1397.591323
ENSG00000141401	IMPA2	chr18:11981025-12030883	+	protein_coding	1.028372381	0.155855443	6.598244904	4.16054E-11	6.44237E-10	Yes	OK	338.4382835	221.883163	454.993404	195.3501256	232.8738105	237.425553	382.0540441	511.5233373	471.4028306
ENSG00000173114	LRRN3	chr7:111091006-111125454	+	protein_coding	1.026494469	0.204213696	5.026570158	4.9933E-07	4.78961E-06	Yes	OK	176.3834972	116.7290698	236.0379245	107.1274882	113.6360175	129.4237037	288.1516043	174.2029941	245.7591751
ENSG00000006638	TBXA2R	chr19:3594506-3606840	-	protein_coding	1.024103983	0.28247247	3.625500147	0.000288403	0.00163187	Yes	OK	148.464501	97.1669419	199.7620602	84.02155918	81.62587171	125.8533946	146.3773326	302.4797443	150.4291036
ENSG00000156711	MAPK13	chr6:36127809-36144524	+	protein_coding	1.020999743	0.162358491	6.288551568	3.20442E-10	4.51581E-09	Yes	OK	387.8749382	255.2594011	520.4904753	231.0592883	239.2758396	295.4430753	437.2907734	616.0451338	508.1355187
ENSG00000214530	STARD10	chr11:72754729-72794168	-	protein_coding	1.017210461	0.217242498	4.682373258	2.83573E-06	2.39364E-05	Yes	OK	307.2011772	202.3741617	412.0281928	216.3555154	187.2593528	203.5076168	293.6752773	581.2045349	361.2047663
ENSG00000126368	NR1D1	chr17:40092787-40100725	-	protein_coding	1.017200089	0.143908182	7.068396513	1.56734E-12	2.85697E-11	Yes	OK	868.3064937	573.2926018	1163.320386	594.4525326	565.7793265	559.6459463	940.8656219	1417.378907	1131.716628
ENSG00000159251	ACTC1	chr15:34788096-34796139	-	protein_coding	1.0145502	0.100501747	10.09485139	5.82192E-24	2.08323E-22	Yes	OK	9407.271266	6227.521772	12587.02076	6183.986771	6430.838285	6067.74026	11335.49746	14737.5733	11687.99152
ENSG00000128536	CDHR3	chr7:105876796-106033773	+	protein_coding	1.009871762	0.205779431	4.90754472	9.22236E-07	8.45683E-06	Yes	OK	106.2228957	70.46543479	141.9803557	70.36805598	69.62206705	71.40618134	158.3452906	142.5297225	125.0660571
ENSG00000105329	TGFB1	chr19:41301587-41353911	-	protein_coding	1.009138959	0.135893848	7.425935551	1.11986E-13	2.2235E-12	Yes	OK	7586.2045	5034.467128	10137.94187	4907.909338	4978.377921	5217.114124	8322.333876	13035.13495	9056.356791
ENSG00000029153	ARNTL2	chr12:27332854-27425289	+	protein_coding	1.008149876	0.144667258	6.968749473	3.19771E-12	5.58157E-11	Yes	OK	1325.059289	881.5081136	1768.610465	967.2982024	796.252376	880.9737623	2063.091838	1374.61999	1868.119566
ENSG00000135540	NHSL1	chr6:138422043-138692571	-	protein_coding	1.006241871	0.127422126	7.89691634	2.85888E-15	6.27429E-14	Yes	OK	499.7797914	331.5441685	668.0154144	328.7343511	316.9004431	348.9977113	601.1597369	744.321884	658.5646223
ENSG00000275342	PRAG1	chr8:8317736-8386498	-	protein_coding	1.006041876	0.162813607	6.179101957	6.44673E-10	8.83872E-09	Yes	OK	488.9676542	324.1563378	653.7789707	302.4776138	296.894102	373.0972975	545.0023954	799.7501094	616.5844073
ENSG00000221866	PLXNA4	chr7:132123332-132648688	-	protein_coding	1.001461269	0.151620357	6.605058114	3.97361E-11	6.17035E-10	Yes	OK	352.3804771	234.761568	469.9993863	257.3160256	195.2618892	251.7067892	425.3228154	511.5233373	473.1520062
ENSG00000144354	CDCA7	chr2:173354820-173368997	+	protein_coding	1.000610749	0.159075215	6.290173811	3.17111E-10	4.47654E-09	Yes	OK	229.5297698	152.9885843	306.0709554	134.434495	165.6525044	158.8787535	296.4371137	285.0594449	336.7163075
ENSG00000084234	APLP2	chr11:130069837-130144811	+	protein_coding	1.000188333	0.050395998	19.84658248	1.17956E-87	2.11038E-85	Yes	OK	52916.72635	35274.88425	70558.56845	34730.31157	35545.66637	35548.6748	71303.25259	69663.77735	70708.67541
ENSG00000137309	HMGA1	chr6:34236873-34246231	+	protein_coding	0.998115168	0.234931847	4.248530709	2.15177E-05	0.000155139	Yes	OK	6552.499483	4371.53336	8733.465606	4403.779981	4431.804682	4279.015417	6857.639939	11519.5689	7823.187977
ENSG00000066629	EML1	chr14:99737693-99942060	+	protein_coding	0.997635183	0.09014656	11.06681364	1.81748E-28	7.61216E-27	Yes	OK	1234.650889	823.4973221	1645.804457	785.6015802	822.6607463	862.2296397	1623.95984	1677.099734	1636.353796
ENSG00000262576	PCDHGA4	chr5:141355025-141512979	+	protein_coding	0.995146149	0.14890939	6.68289722	2.34264E-11	3.72199E-10	Yes	OK	347.3148877	231.8837158	462.7910053	206.90309	275.2872536	213.3259968	459.3854651	441.8421396	487.1454112
ENSG00000113070	HBEGF	chr5:140332843-140346631	-	protein_coding	0.992457209	0.160530045	6.182376682	6.31436E-10	8.66843E-09	Yes	OK	179.6877701	120.3954107	238.9801295	118.6804526	118.4375393	124.0682401	244.8828331	228.0475559	244.0099995
ENSG00000154358	OBSCN	chr1:228208130-228378874	+	protein_coding	0.990131683	0.280927694	3.524507214	0.000424271	0.002311653	Yes	OK	112.2706525	74.34248152	150.1988235	60.91563055	88.02790087	74.08391314	120.6001922	223.2965652	106.699713
ENSG00000179242	CDH4	chr20:61252426-61940617	+	protein_coding	0.986912914	0.138611519	7.119992066	1.07933E-12	1.98723E-11	Yes	OK	630.5710507	422.025265	839.1168365	449.5153427	411.3303731	405.2300791	740.1721722	1004.042712	773.1356256
ENSG00000115884	SDC1	chr2:20200797-20225433	-	protein_coding	0.983731684	0.120764879	8.145842482	3.7665E-16	8.73192E-15	Yes	OK	4146.087653	2783.128489	5509.046816	2668.73478	2860.106525	2820.544163	4677.630357	6697.313292	5152.196799
ENSG00000164045	CDC25A	chr3:48157146-48188402	-	protein_coding	0.981951606	0.164492802	5.969571892	2.37877E-09	3.0879E-08	Yes	OK	258.7016479	172.9866099	344.4166859	161.7415018	184.0583382	173.1599898	333.2615999	407.0015408	292.986917
ENSG00000011422	PLAUR	chr19:43646095-43670547	-	protein_coding	0.978947343	0.093817226	10.43462259	1.72297E-25	6.53458E-24	Yes	OK	1792.981964	1205.584497	2380.379432	1177.352101	1237.992388	1201.409001	2228.802026	2613.044912	2299.291357
ENSG00000125637	PSD4	chr2:113157325-113209396	+	protein_coding	0.975283325	0.170797702	5.710166544	1.12866E-08	1.34523E-07	Yes	OK	256.3047742	171.95813	340.6514185	159.6409628	184.8585918	171.3748352	278.9454828	405.4178772	337.5908954
ENSG00000110987	BCL7A	chr12:122019422-122062044	+	protein_coding	0.971367429	0.138611532	7.007839936	2.42025E-12	4.29282E-11	Yes	OK	767.8905895	517.5022991	1018.27888	518.8331292	517.7641078	515.9096602	879.1846075	1241.592249	934.059783
ENSG00000069399	BCL3	chr19:44747705-44760044	+	protein_coding	0.970572957	0.171561577	5.657286285	1.53785E-08	1.80164E-07	Yes	OK	825.3556696	556.5981298	1094.11321	558.7433699	520.1648688	590.8861506	880.1052197	1458.55416	943.6802489
ENSG00000151789	ZNF385D	chr3:21412222-22373321	-	protein_coding	0.968639447	0.140143989	6.911744534	4.78729E-12	8.1488E-11	Yes	OK	426.2598013	289.2574273	563.2621753	288.8241104	273.6867463	305.2614252	606.6834098	473.5154113	609.5877048
ENSG00000108960	MMD	chr17:55392613-55421992	-	protein_coding	0.96835578	0.132801876	7.291732668	3.05994E-13	5.90464E-12	Yes	OK	312.619637	211.7003458	413.5389282	207.9533595	220.0697522	207.0779259	424.4022032	391.164905	425.0496765
ENSG00000184226	PCDH9	chr13:66302834-67230445	-	protein_coding	0.967879116	0.199354288	4.855070472	1.20344E-06	1.08422E-05	Yes	OK	203.4181573	138.3672509	268.4690637	141.7863815	137.6436268	135.6717445	338.7852728	196.3742843	270.2476338
ENSG00000087903	RFX2	chr19:5993164-6199572	-	protein_coding	0.964371959	0.163281409	5.906195706	3.50098E-09	4.42937E-08	Yes	OK	500.0331027	337.7006735	662.3655319	286.7235714	349.7108425	376.6676066	548.6848441	806.0847637	632.3269879
ENSG00000168917	SLC35G2	chr3:136818647-136855892	+	protein_coding	0.962602849	0.132871845	7.244596068	4.3373E-13	8.2534E-12	Yes	OK	388.9843971	264.1440725	513.8247218	247.8636002	277.6880146	266.8806028	562.4940264	465.5970934	513.3830455
ENSG00000240184	PCDHGC3	chr5:141475947-141512979	+	protein_coding	0.958241918	0.114472403	8.37094262	5.71591E-17	1.40006E-15	Yes	OK	1803.550907	1224.752157	2382.349658	1290.781206	1134.759668	1248.715596	2120.169792	2755.574634	2271.304547
ENSG00000124216	SNAI1	chr20:49982999-49988886	+	protein_coding	0.957793803	0.120971829	7.917494616	2.42344E-15	5.35434E-14	Yes	OK	458.8820537	311.5600707	606.2040356	312.9803087	297.6943557	324.0055478	567.0970871	644.5510783	606.9639414
ENSG00000134569	LRP4	chr11:46856868-46918642	-	protein_coding	0.952028742	0.147637918	6.448402656	1.13035E-10	1.6627E-09	Yes	OK	872.0285545	593.268611	1150.788498	629.1114259	563.3785656	587.3158415	966.6427622	1437.966533	1047.756199
ENSG00000113805	CNTN3	chr3:74262568-74521140	-	protein_coding	0.951414002	0.126623708	7.513711416	5.74742E-14	1.17082E-12	Yes	OK	969.7121474	661.9848382	1277.439457	635.4130428	703.4229533	647.1185184	1425.107615	1059.470937	1347.739818
ENSG00000129038	LOXL1	chr15:73925989-73952137	+	protein_coding	0.949723131	0.142328639	6.67274791	2.51057E-11	3.97347E-10	Yes	OK	5912.930249	4032.587092	7793.273406	4122.307757	4028.476845	3946.976674	6078.802056	10059.43108	7241.587082
ENSG00000131196	NFATC1	chr18:79395856-79529325	+	protein_coding	0.949598053	0.150488111	6.310120099	2.78819E-10	3.95976E-09	Yes	OK	586.8992436	399.2416816	774.5568056	349.7397409	409.7298659	438.255438	650.8727932	923.2758689	749.5217547
ENSG00000166396	SERPINB7	chr18:63752935-63805376	+	protein_coding	0.948362465	0.111136458	8.533315585	1.42212E-17	3.6348E-16	Yes	OK	734.8505881	502.5808594	967.1203169	532.4866326	505.7603032	469.4956423	1007.149697	888.43527	1005.775984
ENSG00000116774	OLFML3	chr1:113979391-114035572	+	protein_coding	0.948238659	0.089392506	10.60758564	2.74757E-26	1.06412E-24	Yes	OK	10666.65681	7280.131486	14053.18214	7144.983356	7450.361428	7245.049674	13103.99341	15991.83486	13063.71814
ENSG00000137501	SYTL2	chr11:85694224-85811159	-	protein_coding	0.947189046	0.107876844	8.780281361	1.63066E-18	4.39291E-17	Yes	OK	693.5526055	473.9554816	913.1497294	446.3645342	488.154723	487.3471877	916.9297058	863.0966527	959.4228296

ENSG00000177374	HIC1	chr17:2054154-2063241	+	protein_coding	0.947029168	0.296239154	3.19683997	0.00138942	0.006609784	Yes	OK	1737.773773	1186.495675	2289.051872	1087.028925	1276.404563	1196.053537	1676.434733	3187.914792	2002.806089
ENSG00000165633	VSTM4	chr10:49014245-49115509	-	protein_coding	0.945173832	0.094851204	9.964805801	2.17298E-23	7.54584E-22	Yes	OK	1254.402119	857.9203585	1650.88388	897.9804159	870.6759649	805.1046946	1735.353911	1626.4225	1590.87523
ENSG00000179604	CDC42EP4	chr17:73283624-73312175	-	protein_coding	0.944081901	0.14118459	6.686862205	2.28006E-11	3.63309E-10	Yes	OK	1365.331199	932.705018	1797.957379	951.54416	883.4800233	963.0908709	1475.741284	2234.549316	1683.581538
ENSG00000135821	GLUL	chr1:182381704-182392206	-	protein_coding	0.940694553	0.06775415	13.88394009	7.92642E-44	5.79862E-42	Yes	OK	4490.066383	3075.41957	5904.713195	3048.932336	3034.561819	3142.764556	5932.424723	6032.174587	5749.540275
ENSG00000154217	PITPNC1	chr17:67377281-67697261	+	protein_coding	0.938970742	0.104399697	8.993998744	2.38395E-19	6.70767E-18	Yes	OK	679.2129397	465.7702064	892.655673	482.073697	445.7412798	469.4956423	864.454813	907.439233	906.072973
ENSG00000196782	MAML3	chr4:139716753-140154184	-	protein_coding	0.935585147	0.185877416	5.033344906	4.81995E-07	4.62872E-06	Yes	OK	225.2167769	153.7925461	296.6410076	145.9874594	175.2555481	140.1346309	239.3591602	361.0752969	289.4885657
ENSG00000225968	ELFN1	chr7:1688119-1747954	+	protein_coding	0.935113235	0.237037019	3.945009254	7.97969E-05	0.000509684	Yes	OK	100.5840239	68.67286812	132.4951796	55.66428309	81.62587171	68.72844954	103.1085613	145.6970496	148.679928
ENSG00000175567	UCP2	chr11:73974667-73983307	-	protein_coding	0.934607249	0.235875287	3.962294067	7.4233E-05	0.000477109	Yes	OK	132.9535639	90.74683568	175.1602921	102.9264102	95.23018367	74.08391314	137.171211	234.3822103	153.9274549
ENSG00000112137	PHACTR1	chr6:12716805-13290484	+	protein_coding	0.934071754	0.24894948	3.752053447	0.000175392	0.001039594	Yes	OK	74.53992531	51.01090861	98.06894201	59.86536106	51.21623323	41.95113154	91.14060329	115.6074416	87.45878118
ENSG00000130164	LDLR	chr19:11089362-11133816	+	protein_coding	0.931514625	0.093276157	9.986631661	1.74408E-23	6.09501E-22	Yes	OK	3313.66373	2278.012427	4349.315033	2271.732912	2287.124915	2275.179453	3964.155937	4884.01849	4199.770672
ENSG00000034152	MAP2K3	chr17:21284672-21315240	+	protein_coding	0.930931052	0.122266447	7.613953602	2.65836E-14	5.55287E-13	Yes	OK	3476.42926	2390.524578	4562.333941	2310.592883	2398.360172	2462.620679	3832.508399	5534.904222	4319.589203
ENSG00000253159	PCDHGA12	chr5:141430589-141512979	+	protein_coding	0.929958948	0.145304573	6.400066614	1.55309E-10	2.26229E-09	Yes	OK	901.1574899	619.0884369	1183.226543	609.1563055	600.9904868	647.1185184	1001.626024	1477.558123	1070.495482
ENSG00000253953	PCDHGB4	chr5:141387698-141512979	+	protein_coding	0.929110006	0.127684868	7.276586676	3.42372E-13	6.56047E-12	Yes	OK	367.8166764	253.0229637	482.6103892	231.0592883	266.4844635	261.5251392	486.0832176	479.8500656	481.8978843
ENSG00000061337	LZTS1	chr8:20246165-20303963	-	protein_coding	0.928796161	0.167461154	5.546337981	2.91715E-08	3.28653E-07	Yes	OK	453.1671781	311.3544233	594.9799329	346.5889325	308.097653	279.3766845	487.0038297	744.321884	553.6140849
ENSG00000240583	AQP1	chr7:30911694-30925516	+	protein_coding	0.928763775	0.255485498	3.63528961	0.000277669	0.001577096	Yes	OK	76.93706073	53.1050432	100.7690783	60.91563055	36.81166764	61.58783141	96.66427622	107.6891236	97.95383492
ENSG00000153391	INO80C	chr18:35452230-35497991	-	protein_coding	0.927317023	0.219010304	4.234125097	2.29443E-05	0.000164416	Yes	OK	107.7528858	73.81257171	141.6931999	72.46859497	70.42232069	78.54679948	113.235295	171.035667	140.8086377
ENSG00000188158	NHS	chrX:17375420-17735994	+	protein_coding	0.924825206	0.14034834	6.589498709	4.41314E-11	6.79516E-10	Yes	OK	269.0874654	186.0222603	352.1526705	183.7971612	183.2580845	191.0115351	372.8479226	329.4020253	354.2080638
ENSG00000101412	E2F1	chr20:33675683-33686404	-	protein_coding	0.921766999	0.148435226	6.209893852	5.30204E-10	7.32759E-09	Yes	OK	770.9593162	531.4642243	1010.454408	526.1850156	543.3722244	524.8354329	827.6303269	1257.428885	946.3040124
ENSG00000128591	FLNC	chr7:128830377-128859274	+	protein_coding	0.919240392	0.108046182	8.507847042	1.77193E-17	4.51485E-16	Yes	OK	38123.12432	26370.60824	49875.64039	25992.0694	26051.45713	27068.29819	42614.21602	60269.48498	46743.22019
ENSG00000169710	FASN	chr17:82078333-82098332	-	protein_coding	0.919075822	0.145550806	6.314467424	2.71093E-10	3.8567E-09	Yes	OK	7111.603318	4918.446877	9304.759759	4878.501792	4769.51172	5107.32712	7210.234394	12151.45067	8552.594212
ENSG00000094804	CDC6	chr17:40287633-40304657	+	protein_coding	0.918626521	0.103188824	8.902383876	5.46598E-19	1.50201E-17	Yes	OK	852.0173585	590.0349134	1113.999803	593.4022631	577.7831311	598.919346	1203.240086	1067.389255	1071.370069
ENSG00000196639	HRH1	chr3:11137093-11263557	+	protein_coding	0.91851448	0.112977756	8.130047143	4.29124E-16	9.89268E-15	Yes	OK	610.7325361	421.9852812	799.4797911	390.7002511	444.1407726	431.1148199	754.9019667	826.6723903	816.8650162
ENSG00000173546	CSPG4	chr15:75674322-75712848	-	protein_coding	0.918409143	0.127761654	7.188456874	6.55277E-13	1.22706E-11	Yes	OK	5811.723022	4020.121098	7603.324947	3840.835533	4063.688005	4155.839754	6312.637543	9481.393871	7015.943426
ENSG00000106976	DNM1	chr9:128191655-128255248	+	protein_coding	0.917538997	0.147103835	6.237356055	4.45028E-10	6.17116E-09	Yes	OK	806.9686017	557.4497214	1056.487482	575.5476818	581.7843994	515.0170829	906.8029721	1312.85711	949.8023636
ENSG00000082482	KCNK2	chr1:215005775-215237093	+	protein_coding	0.916222346	0.087136177	10.51483292	7.38115E-26	2.82544E-24	Yes	OK	5024.366086	3481.760108	6566.972065	3406.023964	3578.734297	3460.522063	7104.363996	5867.473575	6729.078624
ENSG00000092853	CLSPN	chr1:35720218-35769967	-	protein_coding	0.911364353	0.13162363	6.924017784	4.39011E-12	7.53505E-11	Yes	OK	716.6879669	498.7951242	934.5808096	524.0844767	484.9537084	487.3471877	1058.703978	775.9951556	969.0432955
ENSG00000163110	PDLIM5	chr4:94451857-94668227	+	protein_coding	0.910701471	0.099939062	9.112567725	8.04548E-20	2.3648E-18	Yes	OK	5453.199007	3788.803704	7117.594311	3896.499816	3698.772344	3771.138952	7912.661468	6063.847859	7376.273605
ENSG00000136274	NACAD	chr7:45080438-45088914	-	protein_coding	0.910360312	0.225643443	4.034508162	5.47168E-05	0.000362868	Yes	OK	192.4973912	132.8874503	252.1073322	132.333956	120.8383003	145.4900945	188.7254917	351.5733154	216.0231895
ENSG00000106351	AGFG2	chr7:100539211-100568219	+	protein_coding	0.909933865	0.165673629	5.492327709	3.9667E-08	4.39676E-07	Yes	OK	367.8404484	254.4068103	481.2740865	221.6068629	256.8814198	284.7321481	424.4022032	589.1228529	430.2972034
ENSG00000160613	PCSK7	chr11:117204337-117232525	-	protein_coding	0.909200944	0.128990862	7.048568647	1.80768E-12	3.26612E-11	Yes	OK	1674.829116	1162.556579	2187.101653	1203.608838	1153.165501	1130.895397	1866.080837	2665.30581	2029.918311
ENSG00000109610	SOD3	chr4:24789912-24800842	+	protein_coding	0.905510617	0.181303597	4.994443764	5.90056E-07	5.59609E-06	Yes	OK	1047.506599	727.8858426	1367.127555	698.4292124	704.2232069	781.0051084	1024.641328	1875.057682	1201.683653
ENSG00000178409	BEND3	chr6:107065182-107115269	-	protein_coding	0.902776244	0.223135392	4.045867566	5.21297E-05	0.000348519	Yes	OK	122.6255699	85.1543834	160.0967564	85.07182888	93.62967638	76.76164494	117.8383558	180.5376485	181.9142649
ENSG00000152527	PLEKHH2	chr2:43637273-43767987	+	protein_coding	0.900284694	0.199493032	4.51286285	6.39584E-06	5.1229E-05	Yes	OK	161.9620358	113.6418344	210.2822372	133.3842255	112.0355102	95.50576755	250.406506	175.7866577	204.653548
ENSG00000166471	TMEM41B	chr11:9280654-9314780	-	protein_coding	0.899542134	0.165971792	5.419849508	5.96492E-08	6.4807E-07	Yes	OK	1098.720863	768.0728384	1429.368887	775.0988853	780.2473031	748.8723268	1715.100444	996.1243937	1576.881825
ENSG00000107282	APBA1	chr9:69427530-69672306	-	protein_coding	0.895981968	0.124873271	7.175130128	7.22384E-13	1.34507E-11	Yes	OK	392.9100986	275.0418936	510.7783035	266.768451	266.4844635	291.8727662	521.0664794	483.0173928	528.2510383
ENSG00000167191	GPRC5B	chr16:19856691-19886167	-	protein_coding	0.895967409	0.273733056	3.273142904	0.001063587	0.005265155	Yes	OK	87.31148169	60.49961228	114.1233511	67.21724751	61.61953061	52.66205874	80.09325744	159.9500219	102.326774
ENSG00000116791	CRYZ	chr1:74705482-74733408	-	protein_coding	0.894749749	0.142295905	6.2879515	3.21682E-10	4.52597E-09	Yes	OK	736.2638371	516.1223748	956.4052995	506.2298953	475.3506647	566.7865644	1078.036833	769.6605013	1021.518564
ENSG00000187837	HIST1H1C	chr6:26055787-26056428	-	protein_coding	0.894515522	0.133797771	6.685578643	2.30014E-11	3.66154E-10	Yes	OK	377.5875907	263.8318311	491.3433503	278.3214155	248.0786297	265.0954482	443.7350585	535.278291	495.0167015
ENSG00000174804	FZD4	chr11:86945679-86955391	-	protein_coding	0.892898828	0.103177915	8.65397236	4.97373E-18	1.3057E-16	Yes	OK	702.2433593	491.9213686	912.5653499	515.6823207	482.5529475	477.5288597	936.2625611	905.8555695	895.5779193
ENSG00000143434	SEMA6C	chr1:151131685-151146664	-	protein_coding	0.889580249	0.212740176	4.181533861	2.89549E-05	0.000204636	Yes	OK	289.7175365	202.5536392	376.8814339	212.1544374	161.6512361	233.8552439	273.4218099	492.5193743	364.7031175
ENSG00000168077	SCARA3	chr8:27633868-27676776	+	protein_coding	0.885097733	0.099288012	8.914447116	4.90256E-19	1.35396E-17	Yes	OK	4599.733445	3229.518641	5969.948249	3074.138804	3361.865559	3252.55156	5315.61458	6801.835089	5792.395078
ENSG00000111913	RIPOR2	chr6:24804281-25042170	-	protein_coding	0.882263346	0.11004638	8.017195516	1.08187E-15	2.43273E-14	Yes	OK	577.7155429	406.9428744	748.4882115	423.2586054	405.7285976	391.8414201	752.1401302	715.8159395	777.5085647
ENSG00000079308	TNS1	chr2:217799588-218010421	-	protein_coding	0.880224188	0.096658199	9.106565139	8.50314E-20	2.49042E-18	Yes	OK	15652.4308	11018.28704	20286.57455	10951.16	10971.47746	11132.22367	18147.10679	23653.59928	19059.01759
ENSG00000168404	MLKL	chr16:74671855-74700960	-	protein_coding	0.87592189	0.162805444	5.380175695	7.44132E-08	7.97942E-07	Yes	OK	245.0969362	172.1143606	318.0795118	170.1436578	171.2542799	174.9451443	310.2462961	373.7446056	270.2476338
ENSG00000185614	INKA1	chr3:49803254-49805030	+	protein_coding	0.874488797	0.323431327	2.703785082	0.006855461	0.026060253	Yes	OK	81.05404756	56.60751437	105.5005808	55.66428309	47.21496501	66.94329501	69.04591158	167.8683398	79.58749087
ENSG00000205542	TMSB4X	chrX:12975108-12977227	+	protein_coding	0.872966811	0.066147415	13.19729298	9.09449E-40	5.91681E-38	Yes	OK	5648.314627	3989.340033	7307.289221	3966.867872	3937.24793	4063.904296	7054.65094	7438.467849	7428.748874
ENSG00000138166	DUSP5	chr10:110497838-110511544	+	protein_coding	0.871554093	0.130694045	6.668659581	2.5815E-11	4.07397E-10	Yes	OK	453.8961057	320.1317114	587.6604999	325.5835426	300.0951166	334.716475	540.3993347	661.9713777	560.6107874
ENSG00000197728	RPS26	chr12:56041351-56044676	+	protein_coding	0.869538098	0.267098305	3.255498375	0.001131935	0.005556713	Yes	OK	71.51770094	50.34038016	92.69502172	48.31239665	53.61699416	49.09174967	109.5528464	102.9381329	65.59408589
ENSG00000171097	KYAT1	chr9:128832942-128882494	-	protein_coding	0.869230693	0.242013146	3.591667257	0.000328569	0.001837041	Yes	OK	145.2603112	101.9822803	188.5383421	103.9766797	98.43119824	103.5389629	138.0918232	269.2228091	158.3003939
ENSG00000274070	CASTOR2	chr7:74964818-75024798	+	protein_coding	0.866781579	0.247145316	3.507173815	0.000452893	0.002452206	Yes	OK	108.0066913	75.88750164	140.125881	74.56913395	65.62079883	87.47257214	111.3940707	190.03963	118.9439424
ENSG00000109881	CCDC34	chr11:27330827-27363868	-	protein_coding	0.861726113	0.159787935	5.392936042	6.93156E-08	7.47174E-07	Yes	OK	370.6873584	263.5890074	477.7857094	238.4111748	295.2935947	257.0622528	563.4146385	395.9158957	474.026594
ENSG00000144369	FAM171B	chr2:186693971-186765965	+	protein_coding	0.860660232	0.140978059	6.104923257	1.0285E-09	1.3831E-08	Yes	OK	1262.703908	898.1965136	1627.211302	895.8798769	841.8668337	956.84283	1873.445734	1259.012548	1749.175624
ENSG00000139132	FGD4	chr12:32399529-32646050	+	protein_coding	0.860399365	0.208122829	4.134094139	3.56357E-05	0.000247495	Yes	OK	162.4765547	116.1656893	208.7874202	124.9820696	89.62840816	133.88659	228.3118143	166.2846762	231.7657701
ENSG00000106733	NMRK1	chr9:75060573-75088217	-	protein_coding	0.858981129	0.14805396	5.801811242	6.56024E-09	7.9868E-08	Yes	OK	313.8172804	223.9815683	403.6529926	251.0144087	212.0672157	208.8630804	437.2907734	354.7406426	418.9275619
ENSG00000109586	GALNT7	chr4:173168753-173323967	+	protein_coding	0.858744827	0.140615834	6.107027917	1.01503E-09	1.36722E-08	Yes	OK	622.9931273	443.9860185	802.0002361	445.3142647	453.7438163	432.8999744	900.3586871	639.8000875	865.8419337
ENSG00000197774	EME2	chr16:1773207-1781708	+	protein_coding	0.857740485	0.210620702	4.072441479	4.65229E-05	0.000314483	Yes	OK	259.3528759	183.4323588	335.2733931	145.9874594	193.6613819	210.648235	253.1683425	438.6748125	313.9770244
ENSG00000164330	EBF1	chr5:158695916-159099761	-	protein_coding	0.857245428	0.098941207	8.664190163	4.54734E-18	1.19759E-16	Yes	OK	1160.118765	826.0414037	1494.196127	887.477721	819.4597317	771.1867585	1571.484948	1434.799206	1476.304226
ENSG00000167393	PPP2R3B	chrX:333963-386955	-	protein_coding	0.856633264	0.174814877	4.900230904	9.57241E-07	8.76317E-06	Yes	OK	697.2275825	494.8396844	899.6154806	476.8223495	518.5643615	489.1323422	678.4911578	1187.747687	832.6075968
ENSG00000101955	SRPX	chrX:38149336-38220924	-	protein_coding	0.855720902	0.08400423	10.18664064	2.2749E-24	8.28425E-23	Yes	OK	2087.892062	1486.35395	2689.430173	1565.951813	1398.043117	1495.066922	2681.743206	2761.909289	2624.638023
ENSG00000150995	ITPR1	chr3:4493348-4847840	+	protein_coding	0.854861773	0.150642509	5.674771219	1.38874E-08	1.63744E-07	Yes	OK	252.476272	179.2906962	325.6618479	182.7468917	171.2542799	183.870917	322.2142541	362.6589605	292.1123291
ENSG00000128656	CHN1	chr2:174799363-175005369	-	protein_coding	0.853963218	0.0929858	9.183802498	4.16131E-20	1.23415E-18	Yes	OK	2470.986539	1761.655083	3180.317995	1774.955442	1783.765373	1726.244434	3445.851294	2844.259795	3250.842896
ENSG00000114923	SLC4A3	chr2:219627327-219641980	+	protein_coding	0.852516203	0.197290874	4.321113221	1.55244E-05	0.000115468	Yes	OK	204.444955	144.963135	263.9267749	123.9318001	144.045656	166.9119489	205.2965104	321.4837073	265.000107
ENSG00000184497	TMEM255B	chr13:113759240-113816995	+	protein_coding	0.851417749	0.205271562	4.147762806	3.3574E-05	0.000234761	Yes	OK	301.7809721	214.3762779	389.1856664	232.1095578	189.6601137	221.3591622	313.9287447	535.278291	318.3499635
ENSG00000188015	S100A3	chr1:153547329-153549372	-	protein_coding	0.850265093	0.239908886	3.544116708	0.000393931	0.002159208	Yes	OK	139.7459333	98.77654978	180.7153167	82.97128989	108.034242	105.3241175	153.7422298	250.2188461	138.1848743
ENSG00000142303	ADAMTS10	chr19:8580242-8610735	-	protein_coding	0.849735977	0.191634442	4.434150609	9.2436E-06	7.14318E-05	Yes	OK	441.377358	313.931296	568.82342	322.4327341	325.7032332	293.6579208	429.005264	772.8278285	504.6371674
ENSG00000006468	ETV1	chr7:13891228-13991425	-	protein_coding	0.84894066	0.17739904	4.785486223	1.70574E-06	1.49981E-05	Yes	OK	364.5847309	261.1770122	467.9924495	288.8241104	245.6778688	249.0290574	597.4772882	375.3282692	431.1717912
ENSG00000119900	OGFRL1	chr6:71288803-71308950	+	protein_coding	0.848931737	0.193270872	4.392445316	1.12083E-05	8.5214E-05	Yes	OK	706.6325689	505.5132143	907.7519235	496.7774699	537.7704489	481.9917241	1150.765193	585.9555257	986.5350517
ENSG00000188766	SPRED3	chr19:38388421-38399587	+	protein_coding	0.848888511	0.149920629	5.662252859	1.49398E-08	1.75525E-07	Yes	OK	236.6080538	168.7340912	304.4820164	157.5404238	180.8573236	167.8045262	324.9760905	302.4797443	285.9902145
ENSG00000168778	TCTN2	chr12:123671113-123708403	+	protein_coding	0.848208916	0.124048165	6.837738497	8.04531E-12	1.33225E-10	Yes	OK	401.74058	286.508265	516.9728951	272.0197985	300.0951166	287.4098799	533.9550496	528.9436367	488.019999
ENSG00000170017	ALCAM	chr3:105366909-105576900	+	protein_coding	0.847322246	0.114721774	7.385888619	1.51439E-13	2.97456E-12	Yes	OK	15645.09333	11180.24521	20109.94145	11445.83693	10985.88203	11109.01666	23484.81606	16055.1814	20789.82687
ENSG00000134369	NAV1	chr1:201622885-201826969	+	protein_coding	0.84666719	0.085532734	9.898750406	4.21509E-23	1.43052E-21	Yes	OK	8765.229235	6262.96718	11267.49129	6413.995789	6117.93911	6256.96664	10455.39224	12628.13341	10718.94822
ENSG00000131470	PSMC3IP	chr17:42572315-42577831	-	protein_coding	0.846115983	0.165850007	5.10169398	3.36627E-07	3.29618E-06	Yes	OK	228.2667137	163.6331325	292.900295	190.0987781	139.2441341	161.5564853	271.5805856	291.3940993	315.7262001
ENSG00000197380	DACT3	chr19:46647612-46661138	-	protein_coding	0.845563077	0.141793597	5.963337542	2.47137E-09	3.193E-08	Yes	OK	758.8672322	541.2356252	976.4988393	495.7272004	551.3747609	576.6049143	838.6776727	1182.996696	907.8221487
ENSG00000160013	PTGIR	chr19:46620468-46625118	-	protein_coding	0.84445515	0.169925685	4.969555651	6.71065E-07	6.30824E-06	Yes	OK	425.3728314	303.4344361	547.3112267	312.9803087	276.8877609	320.4352388	435.4495491	690.4773222	516.006809
ENSG00000153551	CMTM7	chr3:32391671-32483067	+	protein_coding	0.844264176	0.165297225	5.107552023	3.26359E-07	3.20326E-06	Yes	OK	197.5807216	141.3861989	253.7752443	135.4847645	136.0431195	152.6307126	283.5485436	248.6351825	229.1420067
ENSG00000108797	CNTNAP1	chr17:42682613-42699814	+	protein_coding	0.839489278	0.097715194	8.591184657	8.60772E-18	2.24182E-16	Yes	OK	2315.651161	1658.659145	2972.643176	1621.616096	1686.134428	1668.226912	2728.694426	3349.448478	2839.786625
ENSG00000156299	TIAM1	chr21:31118416-31559977	-	protein_coding	0.837833536	0.152640027	5.488950389	4.04329E-08	4.47864E-07	Yes	OK	216.2731102	154.7682351	277.7779853	155.4398849	154.4489533	154.4158672	276.1836463	300.8960808	256.2542289
ENSG00000197122	SRC	chr20:37344685-37406050	+	protein_coding	0.837255109	0.11014021	7.601720625	2.92219E-14	6.07314E-13	Yes	OK	2486.973929	1783.384345	3190.563514	1791.759754	1758.95751	1799.43577	2810.628908	3735.862392	3025.199241
ENSG00000167157	PRRX2	chr9:129665641-129722674	+	protein_coding	0.837088724	0.129670272	6.455517633	1.0785E-10	1.59211E-09	Yes	OK	4084.322058	2929.857567	5238.786549	2853.582211	2936.930874	2999.059616	4309.385495	6521.526635	4885.447517
ENSG00000153162	BMP6	chr6:7726797-7881422	+	protein_coding	0.836789753	0.236000989	3.545704437	0.000391565	0.002146956	Yes	OK	109.7248235	78.02286159	141.4267855	75.61940345	88.82815451	69.62102681	127.0444773	185.2886392	111.9472399

ENSG00000115325	DOK1	chr2:74549026-74557554	+	protein_coding	0.836366153	0.159875812	5.231348899	1.68278E-07	1.72254E-06	Yes	OK	952.7159204	682.7494156	1222.682425	669.0216666	671.4128075	707.8137726	953.754192	1577.328929	1136.964155
ENSG00000159840	ZYX	chr7:143381080-143391111	+	protein_coding	0.832648809	0.128187596	6.495548969	8.27312E-11	1.2432E-09	Yes	OK	20421.78673	14685.12199	26158.45147	14667.01346	14403.76534	14984.58715	21104.11303	33079.56492	24291.67647
ENSG00000171033	PKIA	chr8:78516139-78605267	+	protein_coding	0.831466386	0.228350153	3.64119041	0.00027138	0.001546182	Yes	OK	214.3277648	154.9800643	273.6754654	143.8869204	164.8522507	156.2010217	325.8967027	166.2846762	328.8450172
ENSG00000196155	PLEKHG4	chr16:67277510-67289499	+	protein_coding	0.831121602	0.128159369	6.48506315	8.86946E-11	1.32358E-09	Yes	OK	620.2314845	445.3690757	795.0938933	401.2029461	437.7387434	497.1655376	730.0454385	882.1006157	773.1356256
ENSG00000182195	LDOC1	chrX:141175745-141177125	-	protein_coding	0.830875721	0.151246766	5.493510659	3.94021E-08	4.37329E-07	Yes	OK	1310.254487	941.6581995	1678.850775	931.5890397	918.6911836	974.6943753	1349.617418	2153.782473	1533.152434
ENSG00000113356	POLR3G	chr5:90471748-90514553	+	protein_coding	0.829321764	0.243065737	3.411923766	0.000645062	0.003358973	Yes	OK	92.01198965	66.77955102	117.2444283	76.66967294	57.61826239	66.05071774	149.139169	95.01981498	107.5743009
ENSG00000102024	PLS3	chrX:115561174-115650861	+	protein_coding	0.829216756	0.10060144	8.242593283	1.68518E-16	3.98535E-15	Yes	OK	13135.17905	9462.745538	16807.61256	9692.937144	9738.286597	8957.012872	18612.93654	14123.11183	17686.78932
ENSG00000185972	CCIN	chr9:36169394-36171334	+	protein_coding	0.828897271	0.252322708	3.285068072	0.001019577	0.005065573	Yes	OK	90.60909818	64.57298746	116.6452089	50.41293563	80.82561807	62.48040867	96.66427622	145.6970496	107.5743009
ENSG00000109466	KLHL2	chr4:165207618-165323156	+	protein_coding	0.827048034	0.129553437	6.38383705	1.72705E-10	2.5024E-09	Yes	OK	559.6555408	404.5829963	714.7280853	431.6607613	392.9245393	389.1636883	801.8531865	620.7961245	721.5349447
ENSG00000254087	LYN	chr8:55879813-56014168	+	protein_coding	0.826657318	0.12143769	6.807254939	9.94786E-12	1.6407E-10	Yes	OK	596.0656734	428.795241	763.3361057	380.1975562	454.5440699	451.644097	724.5217656	820.337736	745.1488157
ENSG00000214575	CPEB1	chr15:82543201-82648861	-	protein_coding	0.826383992	0.202003558	4.090937802	4.29632E-05	0.000292343	Yes	OK	128.0074748	92.07722177	163.9377278	74.56913395	108.8344956	92.82803574	179.5193701	158.3663583	153.9274549
ENSG00000183876	ARSI	chr5:150296343-150339307	-	protein_coding	0.825502756	0.156627676	5.27047824	1.36069E-07	1.41395E-06	Yes	OK	536.066609	386.0758539	686.0573642	432.7110308	341.7083061	383.8082247	584.5887181	837.7580354	635.8253392
ENSG00000143867	OSR1	chr2:19351485-19358653	-	protein_coding	0.824822708	0.178236203	4.627694561	3.69759E-06	3.06766E-05	Yes	OK	442.3996905	318.162021	566.6373601	317.1813867	301.6956239	335.6090523	454.7824043	747.4892112	497.6404649
ENSG00000102967	DHODH	chr16:72008588-72027664	+	protein_coding	0.821792172	0.271169447	3.030548541	0.002441099	0.010801209	Yes	OK	146.9358766	105.2812335	188.5905198	74.56913395	113.6360175	127.6385491	162.0277392	270.8064727	132.9373474
ENSG00000069667	RORA	chr15:60488284-61229319	-	protein_coding	0.820529578	0.191980569	4.274024088	1.91976E-05	0.000139882	Yes	OK	437.7425441	317.5297905	557.9552976	311.9300392	288.0913119	352.5680204	687.6972794	380.0792599	606.0893536
ENSG00000128298	BAIAP2L2	chr22:38084889-38110670	-	protein_coding	0.820120177	0.196661707	4.170207764	3.04322E-05	0.000214341	Yes	OK	360.3588416	259.4968729	461.2208103	222.6571324	272.8864927	282.9469936	338.7852728	604.9594887	439.9176693
ENSG00000167513	CDT1	chr16:88803213-88809258	+	protein_coding	0.819447871	0.171235168	4.785511533	1.70552E-06	1.49981E-05	Yes	OK	675.8889164	487.6232468	864.1545859	473.671541	477.7514256	511.4467739	707.0301346	1148.156098	737.2775254
ENSG00000130830	MPP1	chrX:154778684-154821007	-	protein_coding	0.812777563	0.122312903	6.645068058	3.03077E-11	4.74657E-10	Yes	OK	383.7092347	278.086228	489.3322415	281.4722239	276.0875073	276.6989527	483.3213811	511.5233373	473.1520062
ENSG00000141574	SECTM1	chr17:82321024-82334074	-	protein_coding	0.812452698	0.23001997	3.532096353	0.000412279	0.002252278	Yes	OK	225.2673312	162.3733945	288.161268	140.736112	169.6537726	176.7302988	224.6293657	413.3361952	226.5182433
ENSG00000100911	PSME2	chr14:24143362-24147570	-	protein_coding	0.812228525	0.113856216	7.133809207	9.76286E-13	1.80355E-11	Yes	OK	571.7431771	414.3992109	729.0871433	404.3537545	396.1255539	442.7183243	704.2682982	772.8278285	710.1653032
ENSG00000108691	CCL2	chr17:34255218-34257203	+	protein_coding	0.809850557	0.189945787	4.263587896	2.0117E-05	0.000145935	Yes	OK	137.4122343	99.23300241	175.5914661	87.17236786	101.6322128	108.8944265	160.1865149	199.5416115	167.0462721
ENSG00000112139	MDGA1	chr6:37630679-37699306	-	protein_coding	0.809553774	0.164059915	4.934500747	8.0356E-07	7.43903E-06	Yes	OK	689.1724056	499.5403403	878.804471	512.5315122	473.7501574	512.3393511	718.9980926	1143.405107	774.0102135
ENSG00000067057	PFKP	chr10:3066333-3137712	+	protein_coding	0.8073493	0.093781733	8.608811892	7.38217E-18	1.92568E-16	Yes	OK	5484.810596	3986.998581	6982.62261	3888.09766	4011.671519	4061.226564	6376.159782	7956.325841	6615.382209
ENSG00000130653	PNPLA7	chr9:137459953-137550534	-	protein_coding	0.806915259	0.232501112	3.470586664	0.000519323	0.002773541	Yes	OK	128.8372525	93.05920383	164.6153012	97.67506278	105.633481	75.86906768	126.1238652	213.7945837	153.9274549
ENSG00000175352	NRIP3	chr11:8980576-9004049	-	protein_coding	0.806657831	0.124799795	6.463615051	1.02231E-10	1.51324E-09	Yes	OK	505.1068413	368.3111311	641.9025516	385.4489037	378.5199737	340.9645159	660.0789147	576.4535442	689.1751957
ENSG00000136830	FAM129B	chr9:127505339-127578989	-	protein_coding	0.804498951	0.117831162	6.827556802	8.63729E-12	1.42884E-10	Yes	OK	17474.37525	12722.71282	22226.03768	12273.44929	12826.46541	13068.22376	18315.57881	27142.41015	21220.12408
ENSG00000077238	IL4R	chr16:27313668-27364778	+	protein_coding	0.804412127	0.119214203	6.747619916	1.5029E-11	2.43003E-10	Yes	OK	1443.089876	1049.47677	1836.702982	1042.917606	1021.923904	1083.588802	1608.309434	2164.868118	1736.931394
ENSG00000187634	SAMD11	chr1:923928-944581	+	protein_coding	0.803928737	0.307981551	2.610314589	0.009045899	0.032774709	Yes	OK	1109.904099	807.868967	1411.939232	748.842148	856.2713993	818.4933536	1032.926837	1998.583442	1204.307417
ENSG00000159176	CSRP1	chr1:201483530-201509456	-	protein_coding	0.803220122	0.066301174	12.11471951	8.82656E-34	4.55439E-32	Yes	OK	16093.96495	11724.3934	20463.5365	11463.69151	11659.6956	12049.7931	19542.75481	21626.50989	20221.3448
ENSG00000122707	RECK	chr9:36036433-36124451	+	protein_coding	0.80248649	0.069918999	11.4773739	1.71408E-30	7.92521E-29	Yes	OK	8676.440241	6324.869751	11028.01073	6222.846742	6418.034227	6333.728285	11623.64906	10353.99251	11106.39062
ENSG00000168140	VASN	chr16:4371848-4383528	+	protein_coding	0.801332722	0.272021062	2.945848079	0.003220705	0.013690054	Yes	OK	10890.72246	7940.976397	13840.46852	7490.522019	8076.960031	8255.44714	10322.82409	18867.76793	12330.81356
ENSG00000162576	MXRA8	chr1:1352689-1361777	-	protein_coding	0.800737099	0.238568547	3.356423589	0.000789575	0.004033647	Yes	OK	21016.89346	15329.00783	26704.77909	14816.15173	15184.8129	15986.05885	20444.03411	35131.99292	24538.31024
ENSG00000148926	ADM	chr11:10304680-10307397	+	protein_coding	0.800726753	0.08900017	8.996912586	2.32154E-19	6.55448E-18	Yes	OK	8594.143874	6266.656302	10921.63145	5890.961582	6238.777411	6670.229915	10040.19616	12122.94473	10601.75345
ENSG00000102683	SGCG	chr13:23180952-23325165	+	protein_coding	0.800484058	0.152233888	5.258251429	1.45432E-07	1.50553E-06	Yes	OK	290.2380145	211.0839543	369.3920747	181.6966222	232.8738105	218.6814304	389.4189413	386.4139142	332.3433685
ENSG00000244486	SCARF2	chr22:20424815-20437826	-	protein_coding	0.800331105	0.299334641	2.673700254	0.007501947	0.028076864	Yes	OK	3317.726518	2419.883591	4215.569445	2435.574953	2292.726691	2531.349129	3011.322357	5899.146847	3736.239132
ENSG00000135074	ADAM19	chr5:157395534-157575775	-	protein_coding	0.79999949	0.092903408	8.611088777	7.23699E-18	1.89081E-16	Yes	OK	2752.596952	2006.913729	3498.280176	2016.517425	1995.032335	2009.191428	3199.127237	3900.563405	3395.149885
ENSG00000136490	LIMD2	chr17:63695902-63701172	-	protein_coding	0.7993346	0.169058254	4.728160732	2.26563E-06	1.9423E-05	Yes	OK	281.9983541	205.1315239	358.8651842	205.8528205	179.2568163	230.2849348	336.9440485	429.172831	310.4786732
ENSG00000165804	ZNF219	chr14:21090046-21104722	-	protein_coding	0.79929565	0.165366803	4.833471026	1.34173E-06	1.19766E-05	Yes	OK	570.7885916	415.2906691	726.2865142	403.303485	425.7349387	416.8335836	586.4299424	939.1125047	653.3170954
ENSG00000107611	CUBN	chr10:16823964-17129831	-	protein_coding	0.798995212	0.156004579	5.121613839	3.02932E-07	2.98221E-06	Yes	OK	216.1886534	157.8717563	274.5055504	151.2388069	145.6461633	176.7302988	265.1363005	272.3901363	285.9902145
ENSG00000142102	PGGHG	chr11:289135-296107	+	protein_coding	0.79777288	0.192813385	4.137538896	3.51051E-05	0.000244345	Yes	OK	253.9926792	184.4410732	323.5442852	182.7468917	192.0608746	178.5154534	264.2156883	429.172831	277.2443363
ENSG00000125966	MMP24	chr20:35226654-35277000	+	protein_coding	0.796389655	0.256797232	3.10123925	0.001927125	0.008811244	Yes	OK	95.0784535	68.7881046	121.3688024	66.16697801	76.82434985	63.37298594	87.45815467	164.7010126	111.9472399
ENSG00000174437	ATP2A2	chr12:110280756-110351093	+	protein_coding	0.796384857	0.055640994	14.31291572	1.81731E-46	1.42443E-44	Yes	OK	19031.65826	13907.36978	24155.94674	13878.26107	13934.81671	13909.03155	23867.79072	24694.06625	23905.98325
ENSG00000170522	ELOVL6	chr4:110045846-110199199	-	protein_coding	0.796363261	0.132783202	5.997469924	2.00415E-09	2.61398E-08	Yes	OK	686.845157	503.339665	870.350649	549.2909445	476.951172	483.7768786	918.7709302	728.4852482	963.7957686
ENSG00000128872	TMOD2	chr15:51751561-51816368	+	protein_coding	0.796152848	0.119090835	6.68525709	2.3052E-11	3.66604E-10	Yes	OK	1237.87264	906.1738964	1569.571384	951.54416	877.0779941	889.899535	1775.860846	1327.110083	1605.743222
ENSG00000182752	PAPPA	chr9:116153804-116402322	+	protein_coding	0.79580765	0.078257409	10.16910298	2.72406E-24	9.87621E-23	Yes	OK	2401.1196	1754.936766	3047.302435	1810.664605	1710.942291	1743.203402	2933.070324	3145.155876	3063.681105
ENSG00000160072	ATAD3B	chr1:1471769-1497848	+	protein_coding	0.794384636	0.136686662	5.811720202	6.18341E-09	7.54477E-08	Yes	OK	537.3697694	391.9801006	682.7594383	377.0467477	409.7298659	389.1636883	578.144433	787.0808007	683.053081
ENSG00000127920	GNG11	chr7:93921699-93928610	+	protein_coding	0.793870222	0.12424089	6.389766061	1.6614E-10	2.41365E-09	Yes	OK	2025.695402	1483.586375	2567.804428	1516.589147	1490.872539	1443.29744	2843.770945	2046.093349	2813.548991
ENSG00000134333	LDHA	chr11:18394388-18408425	+	protein_coding	0.793390991	0.088369477	8.978111231	2.75455E-19	7.67171E-18	Yes	OK	28926.20913	21168.82615	36683.59211	21207.04159	21638.05829	20661.37857	40714.07253	31880.73159	37455.97222
ENSG00000117318	ID3	chr1:23557918-23559794	-	protein_coding	0.792454519	0.121641555	6.514669417	7.28499E-11	1.10212E-09	Yes	OK	6352.045765	4648.211582	8055.879947	4513.008008	4721.496501	4710.130237	6688.247302	9875.726103	7603.666436
ENSG00000106089	STX1A	chr7:73699206-73719672	-	protein_coding	0.791462349	0.202748941	3.903657138	9.475E-05	0.000595716	Yes	OK	179.5323046	130.6538161	228.410793	109.2280272	125.6398221	157.093599	198.8522254	283.4757813	202.9043723
ENSG00000126215	XRCC3	chr14:103697609-103715504	-	protein_coding	0.790953837	0.211617889	3.73765111	0.000185747	0.001095843	Yes	OK	237.7691927	173.504496	302.0338894	165.9425798	152.8484461	201.7224623	213.5820198	386.4139142	306.1057341
ENSG00000091073	DTX2	chr7:76461676-76505995	+	protein_coding	0.78953464	0.198894829	3.969608671	7.19908E-05	0.000463784	Yes	OK	212.5705554	154.8914446	270.2496661	147.0377289	148.0469242	169.5896807	213.5820198	353.156979	244.0099995
ENSG00000196781	TLE1	chr9:81583683-81689305	-	protein_coding	0.788591715	0.159459826	4.94539431	7.59899E-07	7.07482E-06	Yes	OK	530.015051	387.5360807	672.4940213	392.8007901	383.3214956	386.4859565	539.4787225	845.6763533	632.3269879
ENSG00000140285	FGF7	chr15:49423096-49488775	+	protein_coding	0.783696268	0.144184881	5.435356774	5.46869E-08	5.98502E-07	Yes	OK	3814.083822	2804.408718	4823.758926	2857.783289	2808.090038	2747.352827	5840.363508	3526.818799	5104.09447
ENSG00000172164	SNTB1	chr8:120535745-120813273	-	protein_coding	0.779546637	0.120496992	6.469428216	9.83744E-11	1.45878E-09	Yes	OK	741.955045	546.4058781	937.504212	597.6033411	495.3570058	546.2572873	1001.626024	966.0347856	844.8518262
ENSG00000162512	SDC3	chr1:30869467-30908761	-	protein_coding	0.776914506	0.091028152	8.534881662	1.40299E-17	3.59148E-16	Yes	OK	7052.381311	5196.266134	8908.496487	5108.510811	5101.616982	5378.67061	8033.26166	10045.17811	8647.049695
ENSG00000054967	RELT	chr11:73376264-73397474	+	protein_coding	0.776577504	0.239340967	3.244649311	0.001175953	0.005737694	Yes	OK	170.5233576	124.7136362	216.333079	109.2280272	140.8446414	124.0682401	196.0903889	302.4797443	150.4291036
ENSG00000163251	FZD5	chr2:207762586-207769563	-	protein_coding	0.774669687	0.197812943	3.916172911	8.99657E-05	0.000568896	Yes	OK	122.2738431	90.13538276	154.4123035	80.87075091	82.42612536	107.109272	161.107127	155.1990311	146.9307524
ENSG00000161682	FAM171A2	chr17:44353215-44363875	-	protein_coding	0.773898022	0.258417173	2.99476235	0.002746587	0.011960008	Yes	OK	190.3751469	139.7549171	240.9953767	135.4847645	132.0418513	151.7381354	141.7742718	346.8223247	234.3895336
ENSG00000091436	MAP3K20	chr2:173075435-173268010	+	protein_coding	0.773777962	0.134465933	5.754453523	8.69225E-09	1.04282E-07	Yes	OK	3881.087586	2866.0234	4896.151772	2803.169275	2883.31388	2911.587044	5803.539022	3707.356448	5177.559846
ENSG00000074181	NOTCH3	chr19:15159038-15200981	-	protein_coding	0.773485534	0.270066441	2.864056458	0.004182535	0.017053386	Yes	OK	4663.455613	3441.860524	5885.050702	3457.487169	3406.679764	3461.414641	4398.684874	8038.676347	5217.790885
ENSG00000219607	PPP1R3G	chr6:5084581-5089487	+	protein_coding	0.770967315	0.265928896	2.899148334	0.003741778	0.015525503	Yes	OK	96.08607332	70.20789548	121.9642512	60.91563055	87.22764722	62.48040867	94.82305191	167.8683398	103.2013618
ENSG00000173334	TRIB1	chr8:125430321-125438405	+	protein_coding	0.770729479	0.207643657	3.711789181	0.000205799	0.001205501	Yes	OK	125.5907924	92.50896604	158.6726188	100.8258713	100.8319592	75.86906768	165.7101878	177.3703213	132.9373474
ENSG00000169432	SCN9A	chr2:166195185-166376001	-	protein_coding	0.770170225	0.175365546	4.39179898	1.12417E-05	8.53495E-05	Yes	OK	260.0640661	193.157379	326.9707533	213.2047069	198.4629038	167.8045262	374.6891469	258.137164	348.0859491
ENSG00000183715	OPCML	chr11:132414977-133532519	-	protein_coding	0.769539333	0.137708958	5.588157406	2.29492E-08	2.63052E-07	Yes	OK	578.1393201	426.5893513	729.6892889	388.5997121	460.9460991	430.2222426	783.4409434	793.4154551	612.2114683
ENSG00000128578	STRIP2	chr7:129434433-129488399	+	protein_coding	0.7671159	0.1876194	4.088681128	4.33833E-05	0.000294834	Yes	OK	124.043538	91.59976472	156.4873114	95.5745238	94.42993002	84.79484034	154.6628419	167.8683398	146.9307524
ENSG00000163661	PTX3	chr3:157436789-157443628	+	protein_coding	0.766218032	0.073533692	10.41995862	2.01041E-25	7.57238E-24	Yes	OK	5618.670733	4162.077767	7075.2637	4168.519615	4226.139495	4091.574191	7438.546208	6608.628132	7178.616759
ENSG00000129009	ISLR	chr15:74173671-74176872	+	protein_coding	0.765883607	0.125330308	6.110920948	9.90578E-10	1.33757E-08	Yes	OK	9411.101311	6968.212311	11853.99031	6773.187956	6969.408988	7162.039989	9785.18659	14794.58519	10982.19915
ENSG00000129667	RHBDF2	chr17:76470891-76501790	-	protein_coding	0.765700745	0.242133398	3.162309496	0.001565231	0.007350558	Yes	OK	232.2482135	171.0327541	293.4636729	123.9318001	185.6588455	203.5076168	208.0583469	403.8342137	268.4984582
ENSG00000187961	KLHL17	chr1:960587-965715	+	protein_coding	0.764982262	0.160783483	4.757841104	1.95674E-06	1.70143E-05	Yes	OK	446.2531743	329.4947363	563.0116122	339.237046	329.7045014	319.5426615	475.9564839	711.0649487	502.013404
ENSG00000161647	MPP3	chr17:43800799-43833170	-	protein_coding	0.76456371	0.170431649	4.486043019	7.25581E-06	5.74738E-05	Yes	OK	170.2079613	125.7060606	214.709862	115.5296442	123.2390612	138.3494764	197.9316132	232.7985467	213.3994261
ENSG00000177283	FZD8	chr10:35638249-35642278	-	protein_coding	0.764001704	0.194609184	3.92582554	8.64328E-05	0.000549087	Yes	OK	561.3700399	414.8546558	707.8854239	399.1024071	416.131895	429.3296653	522.9077037	985.0387486	615.7098195
ENSG00000129467	ADCY4	chr14:24318349-24335093	-	protein_coding	0.763555199	0.226110951	3.376904999	0.000733064	0.003771875	Yes	OK	106.0875907	78.58410415	133.5910773	98.72533228	65.62079883	71.40618134	118.7589679	155.1990311	126.8152327
ENSG00000184232	OAF	chr11:120210767-120230332	+	protein_coding	0.762911707	0.156202221	4.884128424	1.03887E-06	9.47211E-06	Yes	OK	1788.566282	1324.858092	2252.274472	1276.077433	1359.630942	1338.8659	1728.909626	2929.777628	2098.136161
ENSG00000115687	PASK	chr2:241106099-241150264	-	protein_coding	0.758588902	0.170097852	4.459720639	8.20666E-06	6.42023E-05	Yes	OK	171.4373183	127.020612	215.8540246	116.5799136	136.8433732	127.6385491	229.2324265	223.2965652	195.033082
ENSG00000116667	C1orf21	chr1:184387058-184629020	+	protein_coding	0.758411884	0.099540787	7.619106779	2.55437E-14	5.34923E-13	Yes	OK	1015.565637	755.6907543	1275.440521	776.1491548	755.4394402	735.4836678	1330.284563	1171.911051	1324.125947
ENSG00000078804	TP53INP2	chr20:34704290-34713439	+	protein_coding	0.757415226	0.138751632	5.458784264	4.79406E-08	5.28181E-07	Yes	OK	1576.153687	1170.157696	1982.149678	1202.558569	1170.771082	1137.143438	1693.926364	2491.102816	1761.419853
ENSG00000140332	TLE3	chr15:70047790-70098176	-	protein_coding	0.757030576	0.119536459	6.333051721	2.40359E-10	3.4343E-09	Yes	OK	744.8310239	553.1609159	936.5011319	575.5476818	515.3633469	568.5717189	831.3127755	1038.88331	939.3073099
ENSG00000130309	COLGALT1	chr19:17555594-17583162	+	protein_coding	0.755608217	0.107411586	7.034699388	1.99691E-12	3.58052E-11	Yes	OK	5790.652089	4306.183353	7275.120825	4313.456805	4257.349387	4347.743866	6292.384076	8591.374938	6941.603462
ENSG00000133627	ACTR3B	chr7:152759749-152855378	+	protein_coding	0.755261086	0.193137177	3.910490447	9.21089E-05	0.000580441	Yes	OK	143.5323707	106.3054487	180.7592928	107.1274882	121.6385539	90.15030394	179.5193701	202.7089386	160.0495696

ENSG00000110104	CCDC86	chr11:60842071-60851081	+	protein_coding	0.754089928	0.14135054	5.334892459	9.56012E-08	1.01196E-06	Yes	OK	455.7956509	338.0707574	573.5205444	321.3824646	346.509828	346.3199795	533.0344374	679.3916771	508.1355187
ENSG00000148834	GSTO1	chr10:104235356-104267459	+	protein_coding	0.752855711	0.081801817	9.203410593	3.46765E-20	1.03401E-18	Yes	OK	2132.88507	1587.779829	2677.990311	1539.695076	1650.923268	1572.721144	2558.381177	2763.492952	2712.096804
ENSG00000025772	TOMM34	chr20:44942130-44960486	-	protein_coding	0.75241447	0.091961921	8.181804619	2.79624E-16	6.5471E-15	Yes	OK	1651.891102	1229.665559	2074.116645	1153.195903	1307.614455	1228.186319	1959.983277	2145.864155	2116.502505
ENSG00000119943	PYROXD2	chr10:98383565-98415184	-	protein_coding	0.751750842	0.17013925	4.418444539	9.94138E-06	7.6335E-05	Yes	OK	169.9332394	126.0279607	213.8385181	118.6804526	134.4426122	124.9608173	208.0583469	237.5495374	195.9076698
ENSG00000213445	SIPA1	chr11:65638097-65650930	+	protein_coding	0.751537423	0.186106979	4.038201195	5.38626E-05	0.000358069	Yes	OK	496.4092191	368.998502	623.8199362	377.0467477	349.7108425	380.2379156	445.5762828	817.1704088	608.713117
ENSG00000123146	ADGRE5	chr19:14380501-14408725	+	protein_coding	0.751129188	0.099993841	7.511754546	5.83401E-14	1.18699E-12	Yes	OK	2368.683627	1764.431646	2972.935608	1755.000322	1739.751423	1798.543193	2606.253009	3290.852925	3021.70089
ENSG00000119640	ACYP1	chr14:75053221-75069483	-	protein_coding	0.751009031	0.257470793	2.916870767	0.003535622	0.014789414	Yes	OK	76.30228651	57.4193379	95.18523513	56.71455258	58.41851603	57.12494507	109.5528464	64.9302069	111.0726521
ENSG00000103226	NOMO3	chr16:16232495-16294814	+	protein_coding	0.750614311	0.174945403	4.290563221	1.78221E-05	0.000131136	Yes	OK	185.3599401	138.3246839	232.3951964	157.5404238	136.0431195	121.3905083	205.2965104	239.133201	252.7558776
ENSG00000169174	PCSK9	chr1:55039548-55064852	+	protein_coding	0.750527724	0.208335916	3.602488422	0.000315185	0.001771237	Yes	OK	162.5820864	120.3097141	204.8544587	107.1274882	128.8408367	124.9608173	159.2659027	262.8881548	192.4093186
ENSG00000137409	MTCH1	chr6:36968141-36986298	-	protein_coding	0.750499842	0.090001283	8.338768263	7.50684E-17	1.82515E-15	Yes	OK	7913.713593	5898.454259	9928.968926	5706.114152	5971.492693	6017.755933	8994.380749	11204.41985	9588.106181
ENSG00000187957	DNER	chr2:229357629-229714558	-	protein_coding	0.750000545	0.240955042	3.112616112	0.001854371	0.008533111	Yes	OK	100.0680022	74.16805393	125.9679505	60.91563055	84.82688629	76.76164494	92.9818276	144.113386	140.8086377
ENSG00000134201	GSTM5	chr1:109712255-109775428	+	protein_coding	0.749765081	0.217934799	3.440318315	0.00058103	0.003062363	Yes	OK	151.4509979	111.9740501	190.9279458	94.52425431	128.0405831	113.3573129	168.4720243	248.6351825	155.6766305
ENSG00000185453	ZSWIM9	chr19:48170692-48197620	+	protein_coding	0.749447891	0.204627846	3.662492203	0.000249773	0.001431002	Yes	OK	223.96022	166.3801637	281.5402763	187.9982391	142.4451487	168.6971034	231.0736508	372.160942	241.3862361
ENSG00000099822	HCN2	chr19:589893-617159	+	protein_coding	0.748360751	0.237798338	3.147039444	0.001649327	0.007708099	Yes	OK	189.0476546	140.3316603	237.763649	154.3896154	118.4375393	148.1678263	179.5193701	340.4876703	193.2839064
ENSG00000180263	FGD6	chr12:95076749-95217482	-	protein_coding	0.748124628	0.165576007	4.518315426	6.23336E-06	5.00493E-05	Yes	OK	347.9437293	260.7504334	435.1370253	258.3662951	248.0786297	275.8063754	492.5275026	330.9856888	481.8978843
ENSG00000171793	CTPS1	chr1:40979335-41012565	+	protein_coding	0.747991003	0.076404258	9.789912535	1.24403E-22	4.12839E-21	Yes	OK	2022.919939	1509.909599	2535.93028	1522.890764	1487.671525	1519.166508	2525.23914	2557.616686	2524.935013
ENSG00000123505	AMD1	chr6:110874770-110895713	+	protein_coding	0.747680652	0.113252118	6.601913217	4.05885E-11	6.29677E-10	Yes	OK	1671.633622	1249.097615	2094.169629	1187.854796	1265.201012	1294.237037	2332.831199	1773.703213	2175.974476
ENSG00000253731	PCDHGA6	chr5:141373914-141512979	+	protein_coding	0.747303074	0.170045725	4.394718399	1.10916E-05	8.44833E-05	Yes	OK	205.4216777	152.7795626	258.0637927	131.2836865	159.2504752	167.8045262	225.5499778	281.8921178	266.7492826
ENSG00000143797	MBOAT2	chr2:8852690-9003813	-	protein_coding	0.745953135	0.114604852	6.508914088	7.5696E-11	1.14203E-09	Yes	OK	1978.872006	1479.874324	2477.869688	1484.030793	1514.079895	1441.512286	2743.42422	2052.428004	2637.75684
ENSG00000142252	GEMIN7	chr19:45079195-45091524	+	protein_coding	0.744981185	0.189928336	3.92243307	8.76592E-05	0.000556234	Yes	OK	130.1333123	97.57858508	162.6880395	93.47398481	106.4337347	92.82803574	173.075085	140.9460589	174.0429745
ENSG00000047648	ARHGAP6	chrX:11137543-11665701	-	protein_coding	0.743677002	0.204878081	3.629851465	0.000283584	0.001606815	Yes	OK	99.36705463	74.37797201	124.3561372	81.9210204	68.02155976	73.19133588	120.6001922	128.2767502	124.1914693
ENSG00000162062	TEDC2	chr16:2460080-2464963	+	protein_coding	0.742967835	0.184980169	4.016472897	5.90757E-05	0.000388954	Yes	OK	181.9501514	135.2077957	228.6925071	116.5799136	156.0494606	132.9940127	203.4552861	269.2228091	213.3994261
ENSG00000143942	CHAC2	chr2:53767792-53775196	+	protein_coding	0.741974738	0.264934111	2.800601006	0.005100754	0.020230943	Yes	OK	82.58335439	61.99892226	103.1677865	59.86536106	67.22130612	58.91009961	141.7742718	85.51783348	82.21125431
ENSG00000176887	SOX11	chr2:5692667-5701385	+	protein_coding	0.741059471	0.203076823	3.649158286	0.000263101	0.00150161	Yes	OK	127.6968504	95.54981483	159.843886	87.17236786	96.83069095	102.6463857	189.6461038	155.1990311	134.686523
ENSG00000175048	ZDHHC14	chr6:157381133-157678146	+	protein_coding	0.740455299	0.152580946	4.852868716	1.21688E-06	1.09463E-05	Yes	OK	321.7350141	240.3493043	403.1207238	254.1652171	216.0684839	250.814212	392.1807778	465.5970934	351.5843003
ENSG00000115266	APC2	chr19:1446302-1473244	+	protein_coding	0.739352516	0.238174159	3.104251601	0.00190761	0.008734149	Yes	OK	107.7997746	80.10812249	135.4914267	69.31778649	72.82308163	98.18349935	115.0765193	174.2029941	117.1947668
ENSG00000166341	DCHS1	chr11:6621323-6655854	-	protein_coding	0.73804775	0.143378901	5.147533879	2.63933E-07	2.62975E-06	Yes	OK	819.9011642	613.2678696	1026.534459	580.7990292	599.3899795	659.6146001	865.3754252	1257.428885	956.7990661
ENSG00000177542	SLC25A22	chr11:790475-798333	-	protein_coding	0.737595896	0.179593098	4.10703922	4.00763E-05	0.000275086	Yes	OK	827.162591	618.8515681	1035.473614	599.7038801	649.0057055	607.8451187	765.9493125	1396.79128	943.6802489
ENSG00000166337	TAF10	chr11:6606296-6612667	-	protein_coding	0.737590383	0.170748337	4.319751502	1.56205E-05	0.000116131	Yes	OK	190.0332047	142.4475887	237.6188208	164.8923103	131.2415977	131.2088582	240.2797723	256.5535004	216.0231895
ENSG00000087303	NID2	chr14:52004803-52069228	-	protein_coding	0.735060486	0.104326882	7.045743823	1.84473E-12	3.32942E-11	Yes	OK	2664.175504	1997.965716	3330.385292	1964.003951	2004.635379	2025.257818	3107.986633	3856.220825	3026.948417
ENSG00000143013	LMO4	chr1:87328468-87348923	+	protein_coding	0.73482822	0.084468648	8.699419708	3.33586E-18	8.82768E-17	Yes	OK	2553.448032	1915.608576	3191.287488	1952.450986	1926.210522	1868.164219	3045.385007	3450.802947	3077.67451
ENSG00000183762	KREMEN1	chr22:29073078-29168333	+	protein_coding	0.73480897	0.143879974	5.107096892	3.27146E-07	3.20907E-06	Yes	OK	364.5269702	272.5132072	456.5407331	254.1652171	284.8902974	278.4841072	427.1640397	530.5273003	411.9308594
ENSG00000169914	OTUD3	chr1:19882513-19912945	+	protein_coding	0.734601641	0.131270652	5.596084352	2.19247E-08	2.51836E-07	Yes	OK	339.8013522	254.8664102	424.7362941	270.969529	247.2783761	246.3513256	403.2281237	452.9277847	418.052974
ENSG00000188549	CCDC9B	chr15:40331452-40340967	-	protein_coding	0.734095064	0.105850914	6.935179244	4.05706E-12	6.98528E-11	Yes	OK	1963.465969	1472.943398	2453.988541	1513.438338	1477.268227	1428.123627	2254.579166	2822.088505	2285.297952
ENSG00000138134	STAMBPL1	chr10:88879734-88975153	+	protein_coding	0.733218204	0.142170598	5.157312503	2.5052E-07	2.50672E-06	Yes	OK	695.8228113	523.7713503	867.8742723	578.6984903	540.9714635	451.644097	993.3405147	734.8199025	875.4623996
ENSG00000183160	TMEM119	chr12:108589846-108598320	-	protein_coding	0.733089778	0.096278963	7.614225944	2.65276E-14	5.54822E-13	Yes	OK	6926.966562	5202.065273	8651.867851	5237.693958	5227.256804	5141.245057	7713.809242	9953.325619	8288.468693
ENSG00000262814	MRPL12	chr17:81703357-81707526	+	protein_coding	0.732873282	0.218628351	3.352142027	0.000801889	0.00408514	Yes	OK	369.4579682	276.732929	462.1830073	299.3268053	264.8839562	265.9880255	301.0401745	642.9674147	442.5414328
ENSG00000106246	PTCD1	chr7:99416739-99466163	-	protein_coding	0.73253493	0.282565082	2.592446753	0.009529593	0.034338245	Yes	OK	77.3776456	58.16320794	96.59208325	52.51347461	56.81800874	65.15814047	64.44285081	95.01981498	130.313584
ENSG00000155016	CYP2U1	chr4:107931369-107953457	+	protein_coding	0.731859923	0.140899	5.194216571	2.05584E-07	2.0824E-06	Yes	OK	549.4205444	413.7249785	685.1161104	389.6499816	417.7324023	433.7925517	807.3768595	578.0372078	669.9342638
ENSG00000054282	SDCCAG8	chr1:243256034-243500092	+	protein_coding	0.730176328	0.11312821	6.454414229	1.08638E-10	1.60232E-09	Yes	OK	633.4934129	477.3249663	789.6618595	484.1742359	449.7425481	498.0581149	845.1219578	733.2362389	790.6273819
ENSG00000133816	MICAL2	chr11:12094008-12359144	+	protein_coding	0.728376291	0.083126921	8.762219036	1.91444E-18	5.13219E-17	Yes	OK	6297.37386	4738.514224	7856.233497	4604.381454	4944.767268	4666.393951	7473.52947	8651.554154	7443.616866
ENSG00000142156	COL6A1	chr21:45981737-46005050	+	protein_coding	0.725974793	0.103994988	6.980863264	2.93372E-12	5.15357E-11	Yes	OK	119228.1306	89844.82894	148611.4324	87443.33739	90690.34474	91400.8047	127333.5495	180040.3781	138460.3694
ENSG00000185070	FLRT2	chr14:85530144-85654426	+	protein_coding	0.724342237	0.102866004	7.041609529	1.90032E-12	3.41476E-11	Yes	OK	5737.141813	4328.321687	7145.96194	4439.489144	4302.163591	4243.312326	7960.5333	6000.501316	7476.851203
ENSG00000164093	PITX2	chr4:110617423-110642123	-	protein_coding	0.724088808	0.137317494	5.273099482	1.34139E-07	1.39654E-06	Yes	OK	447.4134053	336.5842477	558.2425629	322.4327341	316.9004431	370.4195657	499.8923999	633.4654332	541.3698555
ENSG00000115594	IL1R1	chr2:102064544-102179874	+	protein_coding	0.722205342	0.079914119	9.037268439	1.60636E-19	4.58243E-18	Yes	OK	5716.089498	4316.155357	7116.023639	4338.663273	4286.158519	4323.64428	7665.016798	6500.939008	7182.115111
ENSG00000198225	FKBP1C	chr6:63211446-63213024	+	protein_coding	0.721828469	0.126592863	5.701968098	1.18432E-08	1.40852E-07	Yes	OK	644.2599924	485.1420704	803.3779144	457.9174986	498.5580204	498.9506921	705.1889103	916.9412145	788.0036184
ENSG00000171208	NETO2	chr16:47077703-47143997	-	protein_coding	0.721605326	0.16004919	4.508647167	6.52423E-06	5.21558E-05	Yes	OK	400.8499187	303.4885773	498.2112602	306.6786918	313.6994285	290.0876117	609.4452463	400.6668865	484.5216477
ENSG00000186230	ZNF749	chr19:57435329-57445485	+	protein_coding	0.720778577	0.233068546	3.092560486	0.001984378	0.009020398	Yes	OK	86.95108162	65.35616752	108.5459957	70.36805598	63.2200379	62.48040867	113.235295	126.6930866	85.70960556
ENSG00000023330	ALAS1	chr3:52198086-52214327	+	protein_coding	0.720747265	0.107482778	6.705700014	2.00443E-11	3.21255E-10	Yes	OK	1450.026064	1093.737022	1806.315106	1094.380811	1067.538361	1119.291893	1693.005752	2065.097312	1660.842255
ENSG00000261236	BOP1	chr8:144262102-144291370	-	protein_coding	0.720022484	0.173558442	4.148588077	3.34532E-05	0.000234016	Yes	OK	1153.407901	869.8847274	1436.931074	780.3502328	870.6759649	958.6279845	1158.13009	1935.236898	1217.426234
ENSG00000104812	GYS1	chr19:48968125-48993310	-	protein_coding	0.719380256	0.102908595	6.990477867	2.73952E-12	4.83306E-11	Yes	OK	2232.942363	1686.026462	2779.858263	1694.084691	1664.52758	1699.467116	2466.319962	3159.408848	2713.84598
ENSG00000143476	DTL	chr1:212035553-212107400	+	protein_coding	0.719343448	0.107693994	6.679513157	2.39737E-11	3.80528E-10	Yes	OK	822.6896816	621.5910732	1023.78829	659.5692412	616.9955597	588.2084188	1070.671936	1057.887273	942.8056611
ENSG00000179431	FJX1	chr11:35618419-35620868	+	protein_coding	0.718431816	0.161875999	4.438161423	9.07306E-06	7.02788E-05	Yes	OK	744.7706113	561.6197963	927.9214262	519.8833987	586.5859212	578.3900689	739.25156	1190.915014	853.5977043
ENSG00000055813	CCDC85A	chr2:56184123-56386173	+	protein_coding	0.718313708	0.135246343	5.311150684	1.08935E-07	1.14354E-06	Yes	OK	618.1515824	466.6240361	769.6791288	500.9785478	448.1420408	450.7515197	652.7140175	888.43527	767.8880988
ENSG00000273899	NOL12	chr22:37681673-37693478	+	protein_coding	0.718072066	0.264334323	2.716529803	0.006597026	0.025205905	Yes	OK	80.8011436	60.62652997	100.9757572	70.36805598	62.41978425	49.09174967	92.06121545	133.027741	77.83831525
ENSG00000149090	PAMR1	chr11:35431823-35530300	-	protein_coding	0.716154804	0.102824582	6.964820971	3.28822E-12	5.73348E-11	Yes	OK	2795.00508	2113.410232	3476.599929	2060.628744	2176.689912	2102.912041	3085.891942	3974.995593	3368.912251
ENSG00000197903	HIST1H2BK	chr6:27146418-27146798	-	protein_coding	0.715622931	0.25526007	2.803505184	0.005055042	0.020098065	Yes	OK	83.16640574	62.34642722	103.9863843	58.81509157	56.81800874	71.40618134	90.21999114	137.7787317	83.96042993
ENSG00000100292	HMOX1	chr22:35380361-35394214	+	protein_coding	0.71412494	0.101733784	7.019545642	2.22591E-12	3.9652E-11	Yes	OK	6799.844072	5148.434009	8451.254136	4867.999097	5196.847166	5380.455764	7427.498862	9750.61668	8175.646865
ENSG00000114529	C3orf52	chr3:112086335-112131004	+	protein_coding	0.713088414	0.230561348	3.092835893	0.001982537	0.009014521	Yes	OK	102.489279	77.36637231	127.6121857	67.21724751	87.22764722	77.65422221	150.9803933	133.027741	98.82842273
ENSG00000239306	RBM14	chr11:66616582-66627347	+	protein_coding	0.712999724	0.166390098	4.285109096	1.8265E-05	0.000133856	Yes	OK	1087.710694	822.7731148	1352.648273	780.3502328	881.0792623	806.8898492	1038.45051	1778.454204	1241.040105
ENSG00000159640	ACE	chr17:63477061-63498380	+	protein_coding	0.712014854	0.165977124	4.289837267	1.78804E-05	0.000131506	Yes	OK	320.5376546	242.5330749	398.5422344	278.3214155	244.8776151	204.4001941	341.5471093	464.0134298	390.0661641
ENSG00000175727	MLXIP	chr12:122078722-122147347	+	protein_coding	0.710855365	0.137283616	5.178005835	2.2427E-07	2.26056E-06	Yes	OK	1931.806861	1463.589356	2400.024366	1422.064893	1470.065944	1498.637231	2006.013885	3013.711798	2180.347415
ENSG00000175305	CCNE2	chr8:94879770-94896678	-	protein_coding	0.709006663	0.168842931	4.199208447	2.6785E-05	0.000190199	Yes	OK	163.6593294	124.5014688	202.81719	137.5853035	113.6360175	122.2830855	205.2965104	201.125275	202.0297845
ENSG00000257704	INAFM1	chr19:47274453-47275707	+	protein_coding	0.70797869	0.237193296	2.98481746	0.002837476	0.012313434	Yes	OK	349.8720098	264.7743876	434.9696319	277.271146	237.6753323	279.3766845	280.7867071	644.5510783	379.5711103
ENSG00000181104	F2R	chr5:76716043-76735781	+	protein_coding	0.707676997	0.098315419	7.198026586	6.10902E-13	1.14716E-11	Yes	OK	1052.725286	799.7369323	1305.713641	751.9929965	847.4686093	799.749231	1308.189871	1246.34324	1362.607811
ENSG00000213853	EMP2	chr16:10528422-10580698	-	protein_coding	0.707629165	0.109066623	6.488045039	8.69573E-11	1.30002E-09	Yes	OK	1916.527931	1457.188454	2375.867408	1439.919474	1474.067213	1457.578677	2462.637513	2017.587405	2647.377306
ENSG00000100739	BDKRB1	chr14:96255824-96268967	+	protein_coding	0.70569698	0.166500383	4.238410548	2.25108E-05	0.00016152	Yes	OK	193.6643718	146.5389339	240.7898098	145.9874594	147.2466705	146.3826718	214.502632	278.7247906	229.1420067
ENSG00000130052	STARD8	chrX:68647666-68725842	+	protein_coding	0.70446752	0.176028965	4.001997752	6.28099E-05	0.000410258	Yes	OK	266.0880647	201.3165913	330.859538	190.0987781	196.0621428	217.7888531	291.834053	416.5035223	284.2410388
ENSG00000138669	PRKG2	chr4:81087370-81215117	-	protein_coding	0.704342045	0.246414938	2.858357745	0.0042584	0.017336944	Yes	OK	72.99528477	55.86476765	90.12580189	69.31778649	45.61445772	52.66205874	93.90243976	85.51783348	90.95713243
ENSG00000151491	EPS8	chr12:15620158-15882329	-	protein_coding	0.703867113	0.117931766	5.968426779	2.39552E-09	3.10719E-08	Yes	OK	4254.500657	3238.658137	5270.343177	3362.962914	3120.989213	3232.022283	5944.392681	4222.047112	5644.589737
ENSG00000179833	SERTAD2	chr2:64631621-64751005	-	protein_coding	0.703183647	0.092196586	7.627003089	2.40274E-14	5.05097E-13	Yes	OK	3713.089536	2827.454786	4598.724286	2911.347033	2843.301198	2727.716127	4708.93117	4081.101053	5006.140635
ENSG00000187955	COL14A1	chr8:120059780-120372036	+	protein_coding	0.701600861	0.094461191	7.427397957	1.10755E-13	2.20172E-12	Yes	OK	1353.354666	1030.554412	1676.15492	988.3035923	1117.954341	985.4053025	1712.338607	1626.4225	1689.703652
ENSG00000183696	UPP1	chr7:48088628-48108733	+	protein_coding	0.700661744	0.139083866	5.037692457	4.71178E-07	4.53543E-06	Yes	OK	414.7230114	315.5497691	513.8962537	311.9300392	300.8953702	333.8238978	437.2907734	554.282254	550.1157336
ENSG00000151632	AKR1C2	chr10:4987400-5018031	-	protein_coding	0.7004307	0.181656065	3.855806856	0.000115349	0.000712702	Yes	OK	191.1888791	145.3210865	237.0566717	114.4793747	145.6461633	175.8377216	229.2324265	242.3005282	239.6370604
ENSG00000172594	SMPDL3A	chr6:122789049-122809720	+	protein_coding	0.699794314	0.137969548	5.072092522	3.93465E-07	3.82768E-06	Yes	OK	371.8813602	282.9297518	460.8329687	290.9246494	255.2809125	302.5836934	449.2587314	509.9396737	423.3005009
ENSG00000001630	CYP51A1	chr7:92112151-92142952	-	protein_coding	0.698214798	0.284364986	2.455347294	0.014074854	0.046887693	Yes	OK	68.98552302	52.45349677	85.51754927	65.11670852	36.01141399	56.23236781	73.64897236	107.6891236	75.21455182
ENSG00000174807	CD248	chr11:66314487-66317044	-	protein_coding	0.69706996	0.107822566	6.464972811	1.01317E-10	1.50106E-09	Yes	OK	19249.24046	14685.21096	23813.26996	14169.18572	14790.28785	15096.15931	20173.37414	28336.49249	22929.94325
ENSG00000175166	PSMD2	chr3:184298709-184309054	+	protein_coding	0.696495887	0.070793987	9.838348132	7.69641E-23	2.58259E-21	Yes	OK	9634.128085	7351.097944	11917.15823	7419.103694	7284.708924	7349.481215	11326.29134	12789.6671	11635.51625
ENSG00000069974	RAB27A	chr15:55202966-55319113	-	protein_coding	0.696364405	0.169624078	4.105339359	4.03722E-05	0.000276806	Yes	OK	751.1010796	574.2298841	927.9722751	537.7379801	614.5947988	570.3568735	1160.891927	671.4733592	951.5515392
ENSG00000102760	RGCC	chr13:41457559-41470882	+	protein_coding	0.69434252	0.241570227	2.874288481	0.004049389	0.01661206	Yes	OK	81.05313994	62.12078578	99.9854941	58.81509157	70.42232069	57.12494507	122.4414165	83.9341699	93.58089586

ENSG00000159200	RCAN1	chr21:34513142-34615142	-	protein_coding	0.694024959	0.078693941	8.819293414	1.15184E-18	3.11831E-17	Yes	OK	4330.886219	3310.011123	5351.761316	3411.275311	3401.878242	3116.879816	5571.544759	5045.552175	5438.187014
ENSG00000171408	PDE7B	chr6:135851696-136195574	+	protein_coding	0.692864559	0.195203051	3.549455587	0.000386029	0.00212013	Yes	OK	262.2252524	201.3908336	323.0596712	200.601473	200.0634111	203.5076168	399.545675	223.2965652	346.3367735
ENSG00000167173	C15orf39	chr15:75195643-75212169	+	protein_coding	0.692762716	0.143268548	4.835413813	1.32869E-06	1.18795E-05	Yes	OK	526.4888395	401.2231688	651.7545102	350.7900104	436.9384898	415.9410063	562.4940264	749.0728747	643.6966295
ENSG00000079462	PAFAH1B3	chr19:42297033-42303546	-	protein_coding	0.691875472	0.181383731	3.814429597	0.000136498	0.000828755	Yes	OK	332.7791579	254.3232464	411.2350693	305.6284223	213.667723	243.6735938	324.9760905	476.6827385	432.046379
ENSG00000109762	SNX25	chr4:185204237-185370185	+	protein_coding	0.69142737	0.116253648	5.947575665	2.72143E-09	3.49141E-08	Yes	OK	722.0190688	552.9704336	891.067704	553.4920224	568.9803411	536.4389373	997.9435754	807.6684273	867.5911093
ENSG00000140511	HAPLN3	chr15:88877288-88895626	-	protein_coding	0.690911803	0.1113693	6.203790485	5.51192E-10	7.60487E-09	Yes	OK	767.3337082	586.118668	948.5487484	570.2963343	572.1813556	615.8783141	865.3754252	1035.715983	944.5548368
ENSG00000139318	DUSP6	chr12:89347232-89353271	-	protein_coding	0.690602064	0.171012654	4.038309733	5.38377E-05	0.000358048	Yes	OK	262.6446837	201.7679898	323.5213777	190.0987781	192.0608746	223.1443167	371.0066982	254.9698369	344.5875979
ENSG00000116396	KCNC4	chr1:110211343-110283100	+	protein_coding	0.690222138	0.163726195	4.215709878	2.48994E-05	0.000177575	Yes	OK	596.5059593	455.4304805	737.5814382	441.1131868	436.9384898	488.2397649	560.6528021	912.1902238	739.9012888
ENSG00000108679	LGALS3BP	chr17:78971238-78980109	-	protein_coding	0.689049877	0.113430146	6.074662715	1.24249E-09	1.65063E-08	Yes	OK	2897.657635	2216.954371	3578.3609	2149.901651	2199.897268	2301.064194	3026.052152	4163.45156	3545.578989
ENSG00000128228	SDF2L1	chr22:21642261-21644298	+	protein_coding	0.688972956	0.201060352	3.426697256	0.00061097	0.003198652	Yes	OK	401.2364267	306.15227	496.3205834	303.5278833	317.7006968	297.2282298	341.5471093	669.8896956	477.5249452
ENSG00000176909	MAMSTR	chr19:48712742-48719721	-	protein_coding	0.68768141	0.205686821	3.34334211	0.000827758	0.004203917	Yes	OK	108.4492357	82.74587211	134.1525992	87.17236786	81.62587171	79.43937674	114.1559072	153.6153675	134.686523
ENSG00000121390	PSPC1	chr13:19674752-19783019	-	protein_coding	0.686200493	0.101858697	6.73678846	1.61926E-11	2.60537E-10	Yes	OK	881.4192411	675.2727425	1087.56574	662.7200496	641.003169	722.0950088	1043.974183	1130.735798	1087.987238
ENSG00000136068	FLNB	chr3:58008400-58172251	+	protein_coding	0.686061685	0.068498068	10.01578154	1.29931E-23	4.56005E-22	Yes	OK	14749.63354	11305.31985	18193.94723	11197.97333	11457.23142	11260.7548	17214.52668	19442.63781	17924.6772
ENSG00000110427	KIAA1549L	chr11:33376466-33674102	+	protein_coding	0.685335663	0.083452308	8.212303307	2.16985E-16	5.09498E-15	Yes	OK	2646.893692	2028.719488	3265.067895	2079.533595	2020.640452	1985.984419	3061.956026	3499.896518	3233.35114
ENSG00000073111	MCM2	chr3:127598223-127622436	+	protein_coding	0.683127109	0.121976251	5.60049276	2.13743E-08	2.46029E-07	Yes	OK	1871.109311	1434.668251	2307.55037	1456.723786	1408.446414	1438.834554	1985.760417	2771.41127	2165.479422
ENSG00000064042	LIMCH1	chr4:41359607-41700044	+	protein_coding	0.682184402	0.108880652	6.265432712	3.71792E-10	5.19498E-09	Yes	OK	1183.155223	909.2750537	1457.035391	851.7685583	875.4774868	1000.579116	1562.278826	1325.526419	1483.300929
ENSG00000131153	GINS2	chr16:85676198-85690073	-	protein_coding	0.679860577	0.143643141	4.732983236	2.21244E-06	1.89769E-05	Yes	OK	419.4360009	321.3155299	517.5564719	328.7343511	335.3062769	299.9059616	478.7183203	611.294143	462.6569524
ENSG00000142173	COL6A2	chr21:46098097-46132849	+	protein_coding	0.679462912	0.223274427	3.043173917	0.00234097	0.010425424	Yes	OK	140440.7176	107966.4958	172914.9393	105321.0247	108279.1196	110299.3432	136974.2	224999.0035	156771.6144
ENSG00000157227	MMP14	chr14:22836557-22849027	+	protein_coding	0.679369863	0.085617638	7.934928774	2.10616E-15	4.68479E-14	Yes	OK	35870.01168	27574.59961	44165.42376	27045.4897	27873.63468	27804.67444	40323.73298	50287.65341	41884.8849
ENSG00000108479	GALK1	chr17:75751594-75765711	-	protein_coding	0.679013712	0.198131317	3.427089285	0.000610088	0.003195054	Yes	OK	391.1854817	299.5991756	482.7717877	288.8241104	306.4971457	303.4762707	351.673843	661.9713777	434.6701425
ENSG00000147408	CSGALNACT1	chr8:19404161-19758029	-	protein_coding	0.678169518	0.21955822	3.088791297	0.002009726	0.00912554	Yes	OK	96.65048909	73.85772365	119.4432545	58.81509157	82.42612536	80.33195401	112.3146828	131.4440774	114.5710033
ENSG00000104524	PYCR3	chr8:143603913-143609773	-	protein_coding	0.678167052	0.213024098	3.183522692	0.001454947	0.006875805	Yes	OK	473.5577833	363.1546879	583.9608787	363.3932443	360.1141399	365.9566794	389.4189413	829.8397175	532.6239774
ENSG00000171914	TLN2	chr15:62390526-62844631	+	protein_coding	0.675059242	0.077559408	8.703769915	3.21038E-18	8.52303E-17	Yes	OK	2821.108881	2171.542666	3470.675096	2113.142219	2234.308175	2167.177604	3353.790079	3645.593568	3412.641642
ENSG00000138796	HADH	chr4:107989714-108035175	+	protein_coding	0.674529008	0.116610028	5.784485412	7.27346E-09	8.80303E-08	Yes	OK	723.6990678	558.7742613	888.6238743	576.5979513	540.9714635	558.753369	966.6427622	779.1624828	920.066378
ENSG00000120156	TEK	chr9:27109141-27230175	+	protein_coding	0.674235611	0.163035754	4.135507664	3.54171E-05	0.000246185	Yes	OK	222.7827684	171.8574909	273.708046	186.9479696	145.6461633	182.9783397	251.3271182	280.3084542	289.4885657
ENSG00000064393	HIPK2	chr7:139561570-139777778	-	protein_coding	0.673076885	0.080442269	8.367204106	5.90014E-17	1.44304E-15	Yes	OK	1952.481112	1504.408013	2400.554211	1520.790225	1496.474315	1495.959499	2396.353438	2499.021134	2306.28806
ENSG00000164970	FAM219A	chr9:34398184-34458570	-	protein_coding	0.672379862	0.128509989	5.232121401	1.67576E-07	1.71749E-06	Yes	OK	888.0605699	683.3340248	1092.787115	685.8259785	685.8173731	678.3587227	975.8488837	1308.10612	994.406342
ENSG00000214087	ARL16	chr17:81681174-81683924	-	protein_coding	0.671244678	0.134212941	5.00134097	5.69329E-07	5.4106E-06	Yes	OK	488.3522453	375.8287941	600.8756965	383.3483647	373.7184519	370.4195657	518.304643	690.4773222	593.8451242
ENSG00000172893	DHCR7	chr11:71428193-71452868	-	protein_coding	0.67107979	0.154643092	4.339539387	1.42782E-05	0.000106924	Yes	OK	999.3257077	769.3806456	1229.27077	711.0324463	786.6493323	810.4601582	1072.51316	1578.912592	1036.386557
ENSG00000138675	FGF5	chr4:80266599-80336680	+	protein_coding	0.670967761	0.112589473	5.959418261	2.53137E-09	3.26028E-08	Yes	OK	2577.130875	1990.059311	3164.202438	2018.617964	1878.995557	2072.564413	3594.990463	2666.889474	3230.727377
ENSG00000103888	CEMIP	chr15:80779343-80951776	+	protein_coding	0.670851888	0.064604441	10.38399029	2.93256E-25	1.09456E-23	Yes	OK	44857.20702	34609.79573	55104.6213	34505.5539	34683.7932	34640.03115	51965.79428	59007.3051	54340.76451
ENSG00000108179	PPIF	chr10:79347469-79355337	+	protein_coding	0.669637899	0.124875727	5.362434434	8.21078E-08	8.74753E-07	Yes	OK	1540.752179	1187.993525	1893.510834	1163.698597	1244.394417	1155.88756	1574.246784	2225.047334	1881.238383
ENSG00000113448	PDE4D	chr5:58969038-60522120	-	protein_coding	0.669169131	0.174366321	3.837720073	0.000124182	0.00076043	Yes	OK	203.885412	157.6206171	250.1502069	138.635573	151.2479388	182.9783397	285.3897679	228.0475559	237.013297
ENSG00000185885	IFITM1	chr11:313506-315272	+	protein_coding	0.668823786	0.215315831	3.106245296	0.001894795	0.00869482	Yes	OK	163.5472763	125.7986154	201.2959372	115.5296442	167.2530116	94.61319028	188.7254917	220.129238	195.033082
ENSG00000123610	TNFAIP6	chr2:151357592-151380048	+	protein_coding	0.668774528	0.220487671	3.033160658	0.002420067	0.010716789	Yes	OK	115.3642368	89.63647439	141.0919992	84.02155938	84.02663265	100.8612311	172.1544729	107.6891236	143.4324011
ENSG00000188483	IER5L	chr9:129175552-129178262	-	protein_coding	0.668269781	0.17613934	3.793983669	0.000148249	0.000895032	Yes	OK	890.7603836	686.6914927	1094.829275	679.5243615	675.4140757	705.1360408	829.4715512	1480.72545	974.2908224
ENSG00000166250	CLMP	chr11:123069865-123195281	-	protein_coding	0.668226502	0.077292852	8.645385461	5.3624E-18	1.40549E-16	Yes	OK	8995.123084	6950.271502	11039.97467	7022.101825	7055.836381	6772.8763	11606.15743	10035.67613	11478.09044
ENSG00000132357	CARD6	chr5:40841184-40860175	+	protein_coding	0.668202647	0.128082979	5.21695118	1.81892E-07	1.85384E-06	Yes	OK	379.369323	293.474006	465.2646399	297.2262663	283.2897901	299.9059616	442.8144463	446.5931304	506.386343
ENSG00000213523	SRA1	chr5:140537340-140558252	-	protein_coding	0.666226521	0.117512908	5.669390146	1.43307E-08	1.6885E-07	Yes	OK	1438.105776	1110.554806	1765.656747	1096.48135	1050.733035	1184.450033	1541.104747	2038.175031	1717.690462
ENSG00000152256	PDK1	chr2:172555373-172608669	+	protein_coding	0.66471956	0.161523638	4.115308248	3.86662E-05	0.00026663	Yes	OK	480.2164772	372.8095079	587.6234465	406.4542935	358.5136326	353.4605976	679.41177	445.0094668	638.4491026
ENSG00000136950	ARPC5L	chr9:124862130-124877733	+	protein_coding	0.664703453	0.089824408	7.400031506	1.36152E-13	2.69359E-12	Yes	OK	1265.986468	978.7291993	1553.243738	976.7506278	965.105895	994.3310752	1477.582508	1628.006163	1554.142542
ENSG00000172935	MRGPRF	chr11:69004395-69013409	-	protein_coding	0.664333284	0.161474469	4.114169193	3.88576E-05	0.000267838	Yes	OK	1058.496074	817.5598384	1299.432309	779.2999633	845.0678483	828.3117036	988.7374539	1675.516071	1234.043402
ENSG00000163132	MSX1	chr4:4859666-4863936	+	protein_coding	0.664142968	0.170089225	3.904673966	9.43525E-05	0.000593444	Yes	OK	851.4349239	657.5490155	1045.320832	692.1275954	645.0044373	635.5150139	822.1066539	1392.040289	921.8155536
ENSG00000096060	FKBP5	chr6:35573585-35728583	-	protein_coding	0.663054451	0.123354074	5.375213241	7.64921E-08	8.18635E-07	Yes	OK	408.211572	316.2342254	500.1889187	335.035968	297.6943157	315.9723524	520.1458673	502.0213558	478.3995331
ENSG00000026103	FAS	chr10:88990582-89015785	+	protein_coding	0.662888673	0.141638901	4.680131435	2.86691E-06	2.41873E-05	Yes	OK	1827.114513	1416.195711	2238.033314	1477.729176	1413.247936	1357.610023	2565.746074	1680.267062	2468.086805
ENSG00000100726	TELO2	chr16:1493344-1510457	+	protein_coding	0.661350372	0.165078567	4.006276434	6.16835E-05	0.000403702	Yes	OK	1023.566406	791.7749378	1255.357874	845.4669413	750.6379183	779.2199539	968.4839865	1642.259136	1155.330499
ENSG00000157216	SSBP3	chr1:54225432-54413479	-	protein_coding	0.659315807	0.121925916	5.407511599	6.39064E-08	6.91584E-07	Yes	OK	1058.516527	819.4580957	1297.574958	839.1653243	819.4597317	799.749231	1126.829277	1518.733376	1247.16222
ENSG00000158186	MRAS	chr3:138347648-138405534	+	protein_coding	0.659165428	0.073619066	8.953732545	3.43661E-19	9.53905E-18	Yes	OK	3563.672673	2762.513403	4364.822342	2810.521161	2799.287248	2677.7318	4230.21285	4557.783792	4306.470385
ENSG00000107562	CXCL12	chr10:44370165-44386493	-	protein_coding	0.658299468	0.056699814	11.6102579	3.65511E-31	1.75403E-29	Yes	OK	22798.23911	17685.24006	27911.23816	17606.71777	17768.83192	17680.1705	28754.40003	27790.12855	27189.18589
ENSG00000175182	FAM131A	chr3:184335926-184348421	+	protein_coding	0.658291917	0.184586861	3.566298879	0.000362058	0.002004535	Yes	OK	217.2708434	167.6040757	266.9376111	164.8923103	165.6525044	172.2674125	255.0095668	334.153016	211.6502505
ENSG00000007237	GAS7	chr17:9910609-10198551	-	protein_coding	0.657421568	0.122529826	5.365400315	8.07699E-08	8.62675E-07	Yes	OK	583.6756645	451.8516402	715.4996887	425.3591444	478.5516793	451.644097	642.5872838	790.2481279	713.6636544
ENSG00000180758	GPR157	chr1:9100305-9129170	-	protein_coding	0.657176198	0.21887159	3.0025651	0.002677147	0.011719639	Yes	OK	125.2450397	96.68061543	153.8094641	106.0772187	92.02916909	91.93545848	116.9177436	193.2069571	151.3036914
ENSG00000111885	MAN1A1	chr6:119177209-119349761	-	protein_coding	0.656286228	0.123945608	5.294953493	1.19047E-07	1.24651E-06	Yes	OK	1793.372923	1393.587557	2193.158289	1302.33417	1386.039312	1492.38919	2500.382612	1799.04183	2280.050425
ENSG00000023902	PLEKHO1	chr1:150149183-150164720	+	protein_coding	0.656051937	0.099557986	6.589646557	4.40875E-11	6.79476E-10	Yes	OK	2127.333991	1650.540805	2604.127178	1704.587386	1579.700694	1667.334334	2388.988541	2928.193965	2495.199027
ENSG00000121210	TMEM131L	chr4:153466346-153636711	+	protein_coding	0.654881719	0.110619744	5.920116015	3.21715E-09	4.1018E-08	Yes	OK	616.9983995	478.789124	755.1986351	439.0126478	492.1559912	505.198735	756.743191	768.0768377	740.7758766
ENSG00000188130	MAPK12	chr22:50245450-50261825	-	protein_coding	0.654513032	0.132697464	4.932370297	8.12377E-07	7.51643E-06	Yes	OK	670.129672	519.4340255	820.8253184	495.7272004	522.5656297	540.0092464	697.8240131	956.5328041	808.1191381
ENSG00000169750	RAC3	chr17:82031624-82034204	+	protein_coding	0.654183356	0.262284109	2.494178385	0.012624908	0.042970633	Yes	OK	145.2237551	111.8763341	178.5711762	107.1274882	116.0367784	112.4647356	138.0918232	270.8064727	126.8152327
ENSG00000133805	AMPD3	chr11:10308313-10507579	+	protein_coding	0.653816472	0.147298121	4.438729215	9.04916E-06	7.01267E-05	Yes	OK	295.1606573	228.9563944	361.3649202	241.5619832	232.8738105	212.4333895	315.769969	397.4995593	370.8252322
ENSG00000174371	EXO1	chr1:241847967-241895148	+	protein_coding	0.653548432	0.117623666	5.556266488	2.75606E-08	3.12E-07	Yes	OK	447.3179799	347.1644462	547.4715136	332.9354291	364.9156618	343.6422477	536.7168861	566.9515627	538.7460921
ENSG00000135905	DOCK10	chr2:224765090-225042445	-	protein_coding	0.653229782	0.087279763	7.484321181	7.19179E-14	1.44715E-12	Yes	OK	2159.193634	1679.538527	2638.84874	1724.52506	1741.35193	1572.721144	2716.726468	2481.600834	2718.218919
ENSG00000153048	CARHSP1	chr16:8852942-8869012	-	protein_coding	0.653229341	0.138378426	4.720601039	2.35149E-06	2.01277E-05	Yes	OK	1293.586645	1004.145134	1583.028155	1021.912216	958.7038658	1031.81932	1397.48925	1973.244824	1378.350391
ENSG00000147065	MSN	chrX:65588377-65741931	+	protein_coding	0.653150193	0.064864272	10.06949077	7.53673E-24	2.67359E-22	Yes	OK	29627.22313	23031.11823	36223.32802	23317.033	22935.26944	22841.05226	35693.05384	38598.63251	34378.29771
ENSG00000167693	NXN	chr17:799313-979770	-	protein_coding	0.651179405	0.106253162	6.12856495	8.86752E-10	1.20203E-08	Yes	OK	3824.661447	2973.946666	4675.376227	2975.413472	3078.575769	2867.850758	4182.341018	5469.974016	4373.813647
ENSG00000139668	WDFY2	chr13:51584455-51767707	+	protein_coding	0.649909436	0.108031889	6.015903646	1.78886E-09	2.34432E-08	Yes	OK	712.3701937	555.263139	869.4772483	557.6931004	522.5656297	585.530687	861.6929766	826.6723903	920.066378
ENSG00000085978	ATG16L1	chr2:233210051-233295674	+	protein_coding	0.649235091	0.115040821	5.643519273	1.66609E-08	1.94463E-07	Yes	OK	642.9836986	500.4620079	785.5053894	536.6877106	465.747621	498.9506921	730.0454385	839.341699	787.1290306
ENSG00000082438	COBLL1	chr2:164653624-164843679	-	protein_coding	0.649209265	0.107853766	6.019347186	1.75122E-09	2.29865E-08	Yes	OK	755.1293416	589.0419282	921.2167549	620.7092699	584.9854139	561.4311008	972.1664351	850.427344	941.0564855
ENSG00000004776	HSPB6	chr19:35754569-35758079	-	protein_coding	0.64859844	0.136274284	4.759507231	1.94066E-06	1.68833E-05	Yes	OK	4620.888954	3597.479896	5644.298013	3569.866004	3606.743175	3615.830508	4602.14016	7205.669302	5125.084577
ENSG00000001036	FUCA2	chr6:143494811-143511690	-	protein_coding	0.64842419	0.077704761	8.344716386	7.1385E-17	1.74074E-15	Yes	OK	1967.006458	1531.884957	2402.127959	1496.634027	1562.895367	1536.125476	2420.289354	2415.086964	2371.007558
ENSG00000074800	ENO1	chr1:8861000-8879250	-	protein_coding	0.648285572	0.085546951	7.578125995	3.50582E-14	7.20422E-13	Yes	OK	36677.45381	28570.41365	44784.49397	27812.18643	28755.5142	29143.54034	40082.53259	50569.54553	43701.40378
ENSG00000115107	STEAP3	chr2:119223831-119265652	+	protein_coding	0.647951073	0.10317178	6.280313033	3.37892E-10	4.74143E-09	Yes	OK	2041.062099	1588.840196	2493.284003	1602.711245	1542.889026	1620.920316	2232.484475	2830.006823	2417.360712
ENSG00000136854	STXBP1	chr9:127579370-127696027	+	protein_coding	0.647873005	0.109711686	5.905232397	3.5215E-09	4.44849E-08	Yes	OK	1929.902502	1502.485714	2357.319291	1577.504777	1426.852248	1503.100117	2099.916324	2711.232054	2260.809494
ENSG00000051596	THOC3	chr5:175917873-176034680	-	protein_coding	0.646950874	0.219899531	2.942029349	0.00326069	0.013839046	Yes	OK	87.15648381	67.92484033	106.3881273	73.51886446	62.41978425	67.83587227	93.90243976	112.4401144	112.8218277
ENSG00000276043	UHRF1	chr19:4903080-4962154	+	protein_coding	0.646130455	0.136496321	4.733684031	2.20481E-06	1.89481E-05	Yes	OK	914.5312832	711.5368643	1117.525702	651.1670852	727.4305626	756.012945	962.0397014	1344.530382	1046.007023
ENSG00000127418	FGFRL1	chr4:1009936-1026897	+	protein_coding	0.646087046	0.105566529	6.120188385	9.34648E-10	1.26516E-08	Yes	OK	4089.296762	3186.899978	4991.693546	3185.46737	3052.167399	3323.065164	4469.57201	5824.714658	4680.793969
ENSG00000166833	NAV2	chr11:19350724-20121598	+	protein_coding	0.645728122	0.088600087	7.288120652	3.14308E-13	6.0509E-12	Yes	OK	3077.779365	2398.430526	3757.128205	2341.050698	2433.571332	2420.669547	3520.420879	4155.533242	3595.430494
ENSG00000162409	PRKAA2	chr1:56645322-56715335	+	protein_coding	0.645725357	0.224560781	2.875503695	0.004033834	0.016557833	Yes	OK	95.20328181	74.77939127	115.6271723	82.97128989	74.42358892	66.94329501	136.2505989	93.43615139	117.1947668
ENSG00000109323	MANBA	chr4:102630770-102760994	-	protein_coding	0.645041051	0.132199688	4.879293301	1.06467E-06	9.68735E-06	Yes	OK	1090.853687	852.3972014	1329.310172	869.6231396	845.868102	841.7003626	1542.025359	1065.805591	1380.099567
ENSG00000130204	TOMM40	chr19:44890569-44903689	+	protein_coding	0.643693552	0.173129958	3.717979031	0.000200823	0.001178448	Yes	OK	984.2903835	766.913397	1201.66737	706.8313683	806.6556734	787.2531493	869.978486	1563.075956	1171.947668
ENSG00000259330	INAFM2	chr15:40323664-40326715	+	protein_coding	0.643284941	0.121474056	5.29565706	1.18589E-07	1.24251E-06	Yes	OK	614.925803	480.0404352	749.8111708	524.0844767	423.3341778	492.7026513	717.1568683	794.9991186	737.2775254
ENSG00000149541	B3GAT3	chr11:62615296-62622175	-	protein_coding	0.643269948	0.146956333	4.377286332	1.20166E-05	9.10651E-05	Yes	OK	555.1415775	432.0680286	678.2151265	388.5997121	480.9524402	426.6519335	573.5413722	798.1664458	662.9375614

ENSG00000151320	AKAP6	chr14:32329273-32837681	+	protein_coding	0.641912449	0.200162429	3.206957738	0.001341467	0.006422498	Yes	OK	300.1852254	235.6120278	364.758423	247.8636002	207.2656939	251.7067892	429.005264	245.4678554	419.8021497
ENSG00000065328	MCM10	chr10:13161554-13211104	+	protein_coding	0.64012034	0.180175993	3.552750439	0.000381226	0.002097954	Yes	OK	427.6931019	335.5603766	519.8258272	358.1418969	328.1039941	320.4352388	623.2544286	364.2426241	571.9804289
ENSG00000054392	HHAT	chr1:210328252-210676296	+	protein_coding	0.640075891	0.217396956	2.94427255	0.003237148	0.013754118	Yes	OK	97.1289764	76.00959206	118.2483607	65.11670852	88.82815451	74.08391314	124.2826409	104.5217965	125.9406449
ENSG00000140280	LYSMD2	chr15:51723011-51751585	-	protein_coding	0.639232894	0.19031178	3.35887191	0.000782613	0.00400554	Yes	OK	138.3752671	107.948523	168.8020111	91.37344583	128.0405831	104.4315402	173.075085	166.2846762	167.0462721
ENSG00000184216	IRAK1	chrX:154010500-154019980	-	protein_coding	0.639150444	0.127923295	4.996356947	5.84235E-07	5.54585E-06	Yes	OK	4696.659787	3671.119279	5722.200825	3651.787025	3693.970822	3667.599989	4723.660965	7128.069787	5314.870132
ENSG00000008382	MPND	chr19:4343527-4360086	+	protein_coding	0.638769223	0.227907309	2.802758832	0.005066754	0.020134904	Yes	OK	104.4967079	81.57991659	127.4134992	98.72533228	72.82308163	73.19133588	102.1879491	148.8643768	131.1881718
ENSG00000106100	NOD1	chr7:30424527-30478784	-	protein_coding	0.637241324	0.186563653	3.415677776	0.000636235	0.003318259	Yes	OK	159.5013924	124.5591984	194.4435864	134.434495	108.034242	131.2088582	165.7101878	221.7129016	195.9076698
ENSG00000100401	RANGAP1	chr22:41245611-41286251	-	protein_coding	0.636229509	0.115671639	5.500306834	3.79131E-08	4.22511E-07	Yes	OK	5754.741035	4504.024325	7005.457745	4309.255727	4592.655664	4610.161583	5876.267382	8383.915008	6756.190846
ENSG00000167508	MVD	chr16:88651935-88663161	-	protein_coding	0.634928796	0.142693248	4.449606448	8.60278E-06	6.70145E-05	Yes	OK	627.429892	490.2462835	764.6135004	453.7164207	507.3608105	509.6616193	633.3811623	907.439233	753.020106
ENSG00000124343	XG	chrX:2752048-2816500	+	protein_coding	0.634152866	0.124244111	5.104087906	3.32394E-07	3.25667E-06	Yes	OK	835.6204221	656.0047789	1015.236065	628.0611564	671.4128075	668.5403728	1117.623156	848.8436805	1079.24136
ENSG00000105063	PPP6R1	chr19:55229780-55258995	-	protein_coding	0.634122288	0.12170673	5.210248337	1.88588E-07	1.91852E-06	Yes	OK	3127.944955	2449.669765	3806.220145	2454.479803	2401.561186	2492.968306	3203.730298	4611.628354	3603.301785
ENSG00000175643	RMI2	chr16:11249619-11351762	+	protein_coding	0.632366722	0.148968826	4.244960096	2.18632E-05	0.000157354	Yes	OK	244.4033449	192.0189393	296.7877504	187.9982391	195.2618892	192.7966896	282.6279314	277.141127	330.5941929
ENSG00000135048	CEMIP2	chr9:71683366-71816690	-	protein_coding	0.63096784	0.087659295	7.19795705	6.11214E-13	1.14716E-11	Yes	OK	1894.14133	1487.487007	2300.795653	1475.626837	1514.079895	1472.75249	2440.542821	2128.443855	2333.400282
ENSG00000070404	FSTL3	chr19:676365-683399	+	protein_coding	0.630775481	0.17107896	3.68704299	0.000226875	0.001316307	Yes	OK	2076.352775	1627.874827	2524.830722	1574.353969	1666.928341	1642.342171	1942.491646	3476.141565	2155.858956
ENSG00000164649	CDCA7L	chr7:21900900-21946084	-	protein_coding	0.63052175	0.100098587	6.29900751	2.99557E-10	4.2433E-09	Yes	OK	729.9336735	572.9476775	886.9196696	589.2011852	560.177551	569.4642962	899.4380749	896.353588	864.9673459
ENSG00000175426	PCSK1	chr5:96390415-96434143	-	protein_coding	0.630411251	0.184046483	3.425282795	0.00061416	0.003214331	Yes	OK	211.9120956	166.9515215	256.8726696	159.6409628	191.260621	149.9529808	282.6279314	201.125275	286.8648023
ENSG00000166925	TSC22D4	chr7:100463359-100479279	-	protein_coding	0.629259639	0.171994709	3.658598818	0.000253598	0.00145039	Yes	OK	1568.202996	1230.052821	1906.35317	1219.362881	1244.394417	1226.401165	1393.806802	2573.453322	1751.799387
ENSG00000173267	SNCG	chr10:86958618-86963260	+	protein_coding	0.629121265	0.239192347	2.630189777	0.008533722	0.031230009	Yes	OK	72.6307121	56.93963419	88.32179001	58.81509157	50.41597959	61.58783141	92.9818276	95.01981498	76.96372744
ENSG00000114767	RRP9	chr3:51933430-51941941	-	protein_coding	0.628682269	0.206940157	3.037990677	0.002381613	0.010572102	Yes	OK	490.9737462	384.4778143	597.4696781	358.1418969	348.1103352	447.1812107	450.1793435	839.341699	502.8879918
ENSG00000031081	ARHGAP31	chr3:119294373-119420714	+	protein_coding	0.628428727	0.076960547	8.165595863	3.19852E-16	7.47837E-15	Yes	OK	2152.472424	1690.798226	2614.146622	1720.341428	1706.14077	1645.91248	2558.381177	2646.301847	2637.75684
ENSG00000128891	CCDC32	chr15:40528683-40565057	-	protein_coding	0.627478114	0.191425843	3.277917457	0.00104576	0.005186262	Yes	OK	200.4032768	156.4739819	244.3325718	149.1382679	156.0494606	164.2342171	220.0263049	313.5653894	199.4060211
ENSG00000196352	CD55	chr1:207321376-207386804	+	protein_coding	0.626495579	0.102209071	6.129549709	8.81281E-10	1.19587E-08	Yes	OK	1811.892511	1425.580978	2198.204045	1398.958964	1507.677866	1370.106105	2420.289354	1958.991852	2215.330927
ENSG00000084731	KIF3C	chr2:25926596-25982749	-	protein_coding	0.625472324	0.114258762	5.474174255	4.39556E-08	4.85577E-07	Yes	OK	1373.880054	1079.107046	1668.653062	1094.380811	1084.343688	1058.596638	1585.29413	1939.987889	1480.677165
ENSG00000138080	EMILIN1	chr2:27078567-27086408	+	protein_coding	0.624990921	0.226399931	2.76056145	0.00577021	0.022442736	Yes	OK	10318.27415	8116.837631	12519.71066	7764.642357	8224.206702	8361.663835	9661.824561	16237.30272	11660.00471
ENSG00000112852	PCDHB2	chr5:141094578-141098703	+	protein_coding	0.624841264	0.253693013	2.462981757	0.013778693	0.046087641	Yes	OK	74.2225441	58.02966323	90.41542496	60.91563055	48.01521865	65.15814047	80.09325744	112.4401144	78.71290306
ENSG00000136295	TTYH3	chr7:2631951-2664802	+	protein_coding	0.624477436	0.241704068	2.583644703	0.009776245	0.035035269	Yes	OK	7553.391207	5942.956874	9163.81554	5677.756875	5976.294215	6174.849532	7109.887669	12132.44671	8249.112241
ENSG00000081913	PHLPP1	chr18:62715450-62980433	+	protein_coding	0.624295664	0.139202655	4.484797091	7.29834E-06	5.77551E-05	Yes	OK	397.0229793	311.6078033	482.4381553	293.0251884	293.6930874	348.105134	447.4175071	541.6129454	458.2840134
ENSG00000130956	HABP4	chr9:96450201-96491336	+	protein_coding	0.623837672	0.105550919	5.910300731	3.41484E-09	4.32704E-08	Yes	OK	911.7583854	717.0514816	1106.465289	646.9660073	741.0348746	763.1535631	1136.956011	1118.06649	1064.373367
ENSG00000170525	PFKFB3	chr10:6144934-6254644	+	protein_coding	0.623651525	0.105886044	5.889836858	3.86577E-09	4.83515E-08	Yes	OK	1325.154609	1042.397145	1607.912072	1084.928386	1036.328469	1005.93458	1416.822106	1745.197268	1661.716842
ENSG00000165490	DDIAS	chr11:82899975-82958277	+	protein_coding	0.622219838	0.180803831	3.441408489	0.000578694	0.003051027	Yes	OK	347.4703663	274.8172642	420.1234684	284.6230324	280.0887755	259.7399846	466.7503623	297.7287536	495.8912893
ENSG00000164038	SLC9B2	chr4:103019868-103085829	-	protein_coding	0.621583038	0.107343483	5.79059872	7.0136E-09	8.50728E-08	Yes	OK	958.2059001	754.9729359	1161.438864	819.210204	698.6214314	747.0871723	1203.240086	1203.584323	1077.492184
ENSG00000134996	OSTF1	chr9:75088543-75147265	+	protein_coding	0.621162604	0.101849704	6.098816012	1.06857E-09	1.43347E-08	Yes	OK	917.1834145	721.8617675	1112.505061	739.3897226	736.2333527	689.9622272	1055.942141	1198.833332	1082.739711
ENSG00000164309	CMYA5	chr5:79689877-79800240	+	protein_coding	0.617773613	0.211698621	2.918174949	0.003520867	0.014738932	Yes	OK	195.5216111	155.2144681	235.8287541	159.6409628	156.0494606	149.9529808	245.8034452	159.9500219	301.7327951
ENSG00000162433	AK4	chr1:65147549-65232145	+	protein_coding	0.617536563	0.153087422	4.033881793	5.48629E-05	0.000363691	Yes	OK	270.5111647	213.5610929	327.4612364	219.5063239	240.8763469	180.3006079	334.1822121	316.7327166	331.4687807
ENSG00000127946	HIP1	chr7:75533300-75738962	-	protein_coding	0.616579209	0.092004564	6.701615451	2.06128E-11	3.29725E-10	Yes	OK	2419.413545	1908.367998	2930.459091	1877.881852	1971.024726	1876.197415	2763.677688	3246.510345	2781.189242
ENSG00000106683	LIMK1	chr7:74082933-74122525	+	protein_coding	0.616573189	0.139275754	4.426995875	9.55546E-06	7.35654E-05	Yes	OK	2902.466648	2289.554833	3515.378464	2101.589254	2376.753323	2390.32192	2993.830726	4458.012986	3094.291678
ENSG00000168496	FEN1	chr11:61792637-61797244	+	protein_coding	0.61646734	0.106855126	5.769188292	7.96543E-09	9.59114E-08	Yes	OK	1166.6725	919.6558979	1413.689102	909.5333803	951.501583	897.9327304	1301.745586	1591.581901	1347.739818
ENSG00000130558	OLFM1	chr9:135075422-135121179	+	protein_coding	0.61621321	0.215857711	2.854719465	0.004307486	0.017506474	Yes	OK	131.047114	103.6183894	158.4758386	116.5799136	80.02536442	114.2498901	130.7269259	166.2846762	178.4159136
ENSG00000152977	ZIC1	chr3:147393422-147510293	+	protein_coding	0.615805162	0.108486537	5.676327933	1.37617E-08	1.62378E-07	Yes	OK	846.2882399	667.5576304	1025.018849	594.4525326	698.6214314	709.5989271	1015.435206	1054.719946	1004.901396
ENSG00000153558	FBXL2	chr3:33277025-33403662	+	protein_coding	0.615285276	0.224014472	2.746631814	0.006021069	0.023231783	Yes	OK	151.2867428	120.3018524	182.2716333	117.6301831	127.2403294	116.0350447	217.2644685	118.7747687	210.7756626
ENSG00000099250	NRP1	chr10:33177492-33336262	-	protein_coding	0.615059154	0.062902722	9.777941734	1.40029E-22	4.619E-21	Yes	OK	12294.52234	9714.362804	14874.68187	9738.098733	9667.864276	9737.125403	15160.64096	14156.36877	15307.03588
ENSG00000125835	SNRPB	chr20:2461634-2470853	-	protein_coding	0.613908818	0.158031072	3.884734883	0.000102442	0.000639435	Yes	OK	2414.111505	1906.473971	2921.749238	1855.826193	1890.999361	1972.59576	2217.75468	3854.637161	2692.855873
ENSG00000111252	SH2B3	chr12:111405948-111451623	+	protein_coding	0.613714998	0.092168989	6.65858446	2.76477E-11	4.35069E-10	Yes	OK	2314.805104	1828.010082	2801.600127	1788.608945	1894.200376	1801.220924	2617.300355	3092.894978	2694.605048
ENSG00000137166	FOXP4	chr6:41546426-41602384	+	protein_coding	0.61370924	0.153113934	4.008186744	6.11867E-05	0.000400769	Yes	OK	981.9371175	774.7324069	1189.141828	784.5513107	781.8478104	757.7980995	952.8335799	1518.733376	1095.858528
ENSG00000112118	MCM3	chr6:52264009-52284881	-	protein_coding	0.61284577	0.096373091	6.359096355	2.02944E-10	2.91998E-09	Yes	OK	1945.350319	1536.698712	2354.001927	1438.869204	1540.488265	1630.738666	2277.59447	2573.453322	2210.957988
ENSG00000008513	ST3GAL1	chr8:133454848-133571940	-	protein_coding	0.612207219	0.0873975	7.004859654	2.47234E-12	4.37577E-11	Yes	OK	2292.903599	1812.191005	2773.616193	1712.989542	1882.196571	1841.386901	2597.046888	2940.863274	2782.938417
ENSG00000162769	FLVCR1	chr1:212858255-212899363	+	protein_coding	0.611927331	0.211173958	2.897740502	0.003758614	0.015587501	Yes	OK	125.2197408	99.8187307	150.6207509	105.0269492	86.42739358	108.0018493	165.7101878	115.6074416	170.5446233
ENSG00000110876	SELPLG	chr12:108622277-108633959	-	protein_coding	0.611619605	0.171792643	3.560219998	0.000370544	0.002044215	Yes	OK	235.5441549	185.2619931	285.8263167	174.3447357	198.4629038	182.9783397	249.4858939	349.9896518	258.0034045
ENSG00000114268	PFKFB4	chr3:48517684-48562015	-	protein_coding	0.611554159	0.206327963	2.963990681	0.003036775	0.01303347	Yes	OK	109.0836875	86.38817681	131.7791982	78.77021192	92.02916909	88.36514941	118.7589679	123.5257595	153.0528671
ENSG00000106868	SUSD1	chr9:112040785-112175408	-	protein_coding	0.611290554	0.11377295	5.372898888	7.74808E-08	8.28677E-07	Yes	OK	617.6282554	489.225213	746.0312978	534.5871716	445.7412798	487.3471877	748.4576816	742.7382204	746.8979913
ENSG00000179958	DCTPP1	chr16:30423619-30430075	-	protein_coding	0.611046741	0.15906614	3.841463308	0.000122303	0.000749907	Yes	OK	404.5627502	319.2095985	489.9159019	335.035968	320.9017113	301.6911162	393.10139	593.8738436	482.7724721
ENSG00000103187	COTL1	chr16:84565594-84618077	-	protein_coding	0.610962358	0.083829569	7.288148592	3.14243E-13	6.0509E-12	Yes	OK	9397.757343	7434.909147	11360.60554	7257.362192	7563.997445	7483.367805	10372.53714	12618.63143	11090.64804
ENSG00000184557	SOCS3	chr17:78356778-78360077	-	protein_coding	0.610894939	0.117273262	5.209157888	1.897E-07	1.92863E-06	Yes	OK	1147.764445	906.7602995	1388.768591	859.1204447	919.4914372	941.6690164	1229.017226	1602.667546	1334.621001
ENSG00000180447	GAS1	chr9:86944363-86947189	-	protein_coding	0.6102438	0.128703773	4.741460082	2.12183E-06	1.83047E-05	Yes	OK	1668.602208	1319.363907	2017.84051	1340.143872	1253.197207	1364.750641	1733.512687	2456.262217	1863.746627
ENSG00000112759	SLC29A1	chr6:44219505-44234151	+	protein_coding	0.610115487	0.149048463	4.093403422	4.25087E-05	0.000289848	Yes	OK	441.6719215	348.4462033	534.8976396	306.6768918	405.7285976	332.9313205	515.5428065	608.1268159	481.0232965
ENSG00000181588	MEX3D	chr19:1554669-1568058	-	protein_coding	0.609017539	0.187701405	3.244608312	0.001176123	0.005737694	Yes	OK	1229.597507	972.5603308	1486.634683	974.6500889	890.682306	1052.348598	1064.227651	2061.929985	1333.746413
ENSG00000176490	DIRAS1	chr19:2714567-2721418	-	protein_coding	0.608392938	0.13218615	4.602546764	4.17356E-06	3.43484E-05	Yes	OK	1177.353679	930.9783877	1423.72897	846.5172108	985.1122361	961.3057163	1242.826409	1710.35667	1318.003832
ENSG00000162073	PAQR4	chr16:2969245-2973489	+	protein_coding	0.608343669	0.191875899	3.170505893	0.001521737	0.007164702	Yes	OK	305.567968	241.5972525	369.5386835	251.0144087	233.6740641	240.1032848	255.9301789	435.5074853	417.1783862
ENSG00000104885	DOT1L	chr19:2164149-2232578	+	protein_coding	0.60817801	0.133122953	4.568543554	4.91125E-06	3.98812E-05	Yes	OK	1410.354207	1115.683963	1705.024452	1078.626769	1133.959414	1134.465706	1457.329041	2098.354247	1559.390068
ENSG00000184967	NOC4L	chr12:132144448-132152473	+	protein_coding	0.607726915	0.150735948	4.031731805	5.53673E-05	0.000366739	Yes	OK	447.7022461	353.8031258	541.6013664	367.5943223	368.91693	324.8981251	448.3381192	642.9674147	533.4985652
ENSG00000214193	SH3D21	chr1:36306387-36324886	+	protein_coding	0.606611865	0.203454849	2.981555206	0.002867883	0.012410481	Yes	OK	107.7940534	85.42001128	130.1680956	75.61940345	97.6309446	83.00968581	127.0444773	123.5257595	139.9340499
ENSG00000205364	MT1M	chr16:56632233-56633986	+	protein_coding	0.60638101	0.215550178	2.813177957	0.00490545	0.019588476	Yes	OK	124.8312312	98.24663997	151.4158223	94.52425431	90.4286618	109.7870038	130.7269259	193.2069571	130.313584
ENSG00000101846	STS	chrX:7219456-7354810	+	protein_coding	0.606252603	0.100492538	6.032812104	1.61131E-09	2.12177E-08	Yes	OK	1680.00513	1333.270924	2026.739335	1437.818935	1343.625869	1218.367969	2174.485909	1884.559664	2021.172433
ENSG00000168480	INSIG1	chr7:155297776-155310235	+	protein_coding	0.605762885	0.089811125	6.744853524	1.53181E-11	2.47193E-10	Yes	OK	1885.379058	1495.603771	2275.154345	1400.009233	1583.701962	1503.100117	2358.60834	2147.447818	2319.406877
ENSG00000131351	HAUS8	chr19:17049729-17075625	-	protein_coding	0.604860026	0.141654077	4.269979637	1.95491E-05	0.000142191	Yes	OK	293.9552375	232.5580389	355.3524362	207.9533595	242.4768542	247.2439029	335.1028242	378.4955963	352.4588882
ENSG00000111962	UST	chr6:148747328-149076990	+	protein_coding	0.604700314	0.102557198	5.896225013	3.71912E-09	4.66946E-08	Yes	OK	748.2387811	593.8482774	902.6292849	633.3125039	576.9828775	571.2494507	902.1999114	915.3575509	890.3303924
ENSG00000135049	AGTPBP1	chr9:85546539-85742029	-	protein_coding	0.604165366	0.117939583	5.122668327	3.01242E-07	2.97091E-06	Yes	OK	518.5791823	412.2467859	624.9115786	440.0629173	405.7285976	390.9488428	667.443812	590.7065164	616.5844073
ENSG00000072195	SPEG	chr2:219434846-219498287	+	protein_coding	0.602573357	0.144717524	4.163789834	3.13008E-05	0.000220082	Yes	OK	2793.527161	2216.779716	3370.274605	2158.303807	2176.689912	2315.34543	2668.854636	4317.066927	3124.902252
ENSG00000162746	FCRLB	chr1:161721563-161728143	+	protein_coding	0.601815145	0.22416433	2.684705214	0.00725938	0.02734937	Yes	OK	122.3590905	96.5460587	148.1721224	96.62479329	83.226379	109.7870038	125.203253	191.6232935	127.6898205
ENSG00000197321	SVIL	chr10:29457338-29736781	-	protein_coding	0.601465568	0.120093635	5.008305145	5.49114E-07	5.2427E-06	Yes	OK	719.9425792	571.2463137	868.6388447	496.7774699	623.3975889	593.5638824	902.1999114	886.8516065	816.8650162
ENSG00000151247	EIF4E	chr4:98871684-98930637	-	protein_coding	0.601290827	0.16316324	3.685210143	0.000228514	0.001324416	Yes	OK	393.1160304	313.1852864	473.0467745	290.9246494	304.0963848	344.534825	571.7001479	376.9119327	470.5282428
ENSG00000058668	ATP2B4	chr1:203626561-203744081	+	protein_coding	0.601028738	0.059782227	10.05363577	8.85392E-24	3.12068E-22	Yes	OK	18302.49588	14544.46143	22060.53034	14927.48029	14655.84524	14050.05876	22515.41146	22077.85401	21588.32555
ENSG00000120306	CYSTM1	chr5:140174642-140282052	+	protein_coding	0.600631194	0.106793388	5.624235771	1.86331E-08	2.1601E-07	Yes	OK	765.991239	607.8072822	924.1751958	587.1006462	625.7983498	610.5228505	856.1693037	994.5407301	921.8155536
ENSG00000100644	HIF1A	chr14:61695513-61748259	+	protein_coding	0.600573042	0.113880074	5.273732441	1.33677E-07	1.39261E-06	Yes	OK	24142.26649	19190.62612	29093.90687	19872.14906	18862.77865	18836.95064	34032.26951	23237.09575	30012.35535
ENSG00000165244	ZNF367	chr9:96385941-96418329	-	protein_coding	0.600529105	0.110289439	5.445028211	5.17972E-08	5.68009E-07	Yes	OK	726.1951772	578.0066273	874.3837271	607.0557665	560.177551	566.7865644	953.754192	821.9213996	847.4755896
ENSG00000130066	SAT1	chrX:23783173-23786226	+	protein_coding	0.599948085	0.115045013	5.214898679	1.83918E-07	1.87216E-06	Yes	OK	966.8513745	770.0017531	1163.700996	758.2945734	754.6391865	797.0714992	1240.985184	997.7080573	1252.409747
ENSG00000006327	TNFRSF12A	chr16:3018445-3022383	+	protein_coding	0.59922947	0.143936625	4.163147973	3.1389E-05	0.000220607	Yes	OK	5301.537951	4214.298276	6388.777626	4194.776352	3970.058329	4478.060147	5340.471108	8339.572428	5486.289343
ENSG00000185989	RASA3	chr13:113977783-114132611	-	protein_coding	0.599208941	0.083843805	7.146728849	8.88702E-13	1.6436E-11	Yes	OK	3014.13628	2395.686865	3632.585696	2371.508514	2420.767274	2394.784807	3344.583957	3897.396078	3655.777053
ENSG00000168646	AXIN2	chr17:65528563-65561647	-	protein_coding	0.598414866	0.196850533	3.03994537	0.002366211	0.010513086	Yes	OK	161.8940899	127.9411563	195.8470236	131.2836865	132.0418513	120.497931	180.4399823	247.0515189	160.0495696
ENSG00000196549	MME	chr3:155024124-155183729	+	protein_coding	0.597409963	0.126206033	4.733608584	2.20563E-06	1.89481E-05	Yes	OK	4870.400467	3877.924664	5862.876271	3867.092271	3958.054524	3808.627197	6712.183218	4518.192202	6358.253392

ENSG00000184307	ZDHHC23	chr3:113947901-113965401	+	protein_coding	0.597324528	0.169458597	3.524899528	0.000423643	0.002309762	Yes	OK	161.6220425	128.9312227	194.3128623	127.0826086	124.0393149	135.6717445	211.7407955	180.5376485	190.660143
ENSG00000169744	LDB2	chr4:16501541-16898809	-	protein_coding	0.596906722	0.121855603	4.898475797	9.65829E-07	8.83688E-06	Yes	OK	876.9930819	699.3643685	1054.621795	706.8313683	721.8287871	669.4329501	1173.780497	891.6025972	1098.482292
ENSG00000266074	BAHCC1	chr17:81395475-81466332	+	protein_coding	0.596818403	0.138852033	4.298233103	1.72165E-05	0.000127135	Yes	OK	1381.98119	1098.393209	1665.569171	1050.269492	1121.155356	1123.754779	1414.980881	2080.933948	1500.792685
ENSG00000122490	PQLC1	chr18:79902420-79951664	-	protein_coding	0.596470849	0.193799493	3.077773017	0.002085537	0.009422986	Yes	OK	1176.954505	935.6344541	1418.274555	938.9409261	914.6899154	953.2725209	1031.085613	2033.424041	1190.314012
ENSG00000267534	S1PR2	chr19:10221433-10231331	-	protein_coding	0.595982616	0.126400655	4.715027898	2.41678E-06	2.06543E-05	Yes	OK	495.6296779	393.5265823	597.7327735	383.3483647	400.9270758	396.3043064	551.4466805	679.3916771	562.359963
ENSG00000108175	ZMIZ1	chr10:79069035-79316528	+	protein_coding	0.595527526	0.090343626	6.591804549	4.34512E-11	6.70926E-10	Yes	OK	7782.044808	6196.117221	9367.972394	6065.306318	6288.393137	6234.652208	8485.282228	10612.12967	9006.505286
ENSG00000106624	AEBP1	chr7:44104361-44114562	+	protein_coding	0.595317866	0.093656949	6.356366181	2.06582E-10	2.96973E-09	Yes	OK	15508.54905	12351.20081	18665.89729	12143.21587	12468.75203	12441.63452	16568.25694	21477.64551	17951.78943
ENSG00000165655	ZNF503	chr10:75397830-75401906	-	protein_coding	0.594352946	0.138769866	4.283011597	1.84381E-05	0.000134945	Yes	OK	1412.064578	1123.64864	1700.480517	1098.581889	1084.343688	1188.020342	1434.313737	2117.35821	1549.769603
ENSG00000176454	LPCAT4	chr15:34358618-34367278	-	protein_coding	0.593158987	0.127139676	4.665412149	3.07999E-06	2.58261E-05	Yes	OK	725.4204872	577.2136771	873.6272973	561.8941784	599.3899795	570.3568735	741.0927843	992.9570665	886.8320412
ENSG00000129968	ABHD17A	chr19:1876810-1885547	-	protein_coding	0.592341805	0.203169499	2.915505569	0.003551128	0.014845647	Yes	OK	300.403696	238.6437802	362.1636117	243.6625222	208.0659475	264.202871	272.5011977	487.7683836	326.2212538
ENSG00000099785	MARCH2	chr19:8413270-8439017	+	protein_coding	0.592094024	0.192846839	3.070281205	0.002138573	0.009630893	Yes	OK	497.6351885	395.4972832	599.7730938	332.9354291	423.3341778	430.2222426	479.6389325	812.4194181	507.2609308
ENSG00000105722	ERF	chr19:42247572-42255157	-	protein_coding	0.591918991	0.133523256	4.433077885	9.28973E-06	7.17246E-05	Yes	OK	2072.426684	1651.352068	2493.5013	1594.309089	1654.924536	1704.82258	2094.392651	3100.813295	2285.297952
ENSG00000140564	FURIN	chr15:90868592-90883458	+	protein_coding	0.591525397	0.11452722	5.164932826	2.40525E-07	2.40818E-06	Yes	OK	5443.87016	4341.263308	6546.477012	4222.083359	4262.951163	4538.755402	5600.083736	7853.387708	6185.959593
ENSG00000114450	GNB4	chr3:179396089-179451590	-	protein_coding	0.589549437	0.147098579	4.007852707	6.12733E-05	0.000401177	Yes	OK	2708.634642	2164.473242	3252.796043	2266.481564	2060.653134	2166.285026	3896.95125	2377.079038	3484.357842
ENSG00000144136	SLC20A1	chr2:112645857-112663827	+	protein_coding	0.587102911	0.070683782	8.306048359	9.89419E-17	2.37403E-15	Yes	OK	3535.269553	2826.719888	4243.819218	2889.291373	2764.076087	2826.792204	4315.82978	4122.276306	4293.351568
ENSG00000247077	PGAM5	chr12:132710819-132722734	+	protein_coding	0.587064134	0.138781554	4.230130855	2.33555E-05	0.000167217	Yes	OK	630.2956869	502.9912551	757.6001188	515.6823207	457.7450845	535.5463601	636.1429987	875.7659614	760.8913963
ENSG00000112964	GHR	chr5:42423777-42721878	+	protein_coding	0.58700641	0.179991052	3.261308849	0.001108992	0.005458733	Yes	OK	180.812438	144.7036889	216.9211872	155.4398849	160.8509825	117.8201992	239.3591602	205.8762658	205.5281358
ENSG00000149212	SESN3	chr11:95165513-95232541	-	protein_coding	0.585743992	0.1587857	3.688896373	0.000225229	0.001309064	Yes	OK	940.5012585	753.4652044	1127.537313	725.7362192	755.4394402	779.2199539	1328.443339	814.0030816	1240.165517
ENSG00000126778	SIX1	chr14:60643415-60658259	-	protein_coding	0.585337117	0.133834991	4.373573105	1.22229E-05	9.25009E-05	Yes	OK	316.7595425	253.2480443	380.2710408	264.6679121	242.4768542	252.5993665	394.0220021	394.3322322	352.4588882
ENSG00000165246	NLGN4Y	chrY:14522573-14845650	+	protein_coding	0.585327423	0.154380654	3.791455782	0.000149767	0.000902659	Yes	OK	478.8192184	384.2632974	573.3751393	392.8007901	383.3214956	376.6676066	650.8727932	438.6748125	630.5778123
ENSG00000093009	CDC45	chr22:19479459-19520612	+	protein_coding	0.584323278	0.138200734	4.228076527	2.35698E-05	0.000168531	Yes	OK	499.7236982	398.8449783	600.6024181	369.6948613	384.1217492	442.7183243	530.272601	687.309995	584.2246583
ENSG00000106348	IMPDH1	chr7:128392277-128410252	-	protein_coding	0.583519563	0.130899734	4.457759719	8.28207E-06	6.47614E-05	Yes	OK	1557.883866	1245.531795	1870.235938	1217.262342	1225.988583	1293.34446	1538.34291	2256.720606	1815.644297
ENSG00000075142	SRI	chr7:88205118-88226993	-	protein_coding	0.58252406	0.088938502	6.549739952	5.76374E-11	8.7925E-10	Yes	OK	1207.80795	966.9712597	1448.64464	933.6895787	982.7114751	984.5127253	1488.629854	1447.468515	1409.835553
ENSG00000197785	ATAD3A	chr1:1512151-1534687	+	protein_coding	0.582489592	0.132074117	4.410323563	1.03216E-05	7.8947E-05	Yes	OK	950.9412455	759.8765568	1142.005934	707.8816378	789.8503469	781.8976857	997.0229633	1369.868999	1059.12584
ENSG00000169122	FAM110B	chr8:57994509-58204279	+	protein_coding	0.582217794	0.126487655	4.602961395	4.16525E-06	3.42972E-05	Yes	OK	377.2402214	301.8393212	452.6411215	300.3770748	286.4908046	318.6500842	427.1640397	475.0990749	455.66025
ENSG00000166508	MCM7	chr7:100092728-100101940	-	protein_coding	0.581920661	0.097033745	5.997095728	2.00878E-09	2.61793E-08	Yes	OK	3441.150094	2754.570602	4127.729587	2732.801219	2688.051991	2842.858595	3915.363493	4693.97886	3773.846408
ENSG00000178718	RPP25	chr15:74954416-74957464	-	protein_coding	0.580399887	0.142814393	4.064015375	4.82356E-05	0.000324595	Yes	OK	528.4955298	422.5806683	634.4103913	442.1634563	410.5301195	415.0484291	532.1138253	755.4075291	615.7098195
ENSG00000177311	ZBTB38	chr3:141324213-141449792	+	protein_coding	0.578163663	0.095579609	6.049027308	1.45723E-09	1.92659E-08	Yes	OK	3875.219843	3110.627007	4639.81265	3139.255512	3188.210519	3004.41508	5095.588275	4057.3461	4766.503574
ENSG00000165802	NSMF	chr9:137447573-137459334	-	protein_coding	0.57815712	0.139541865	4.143252075	3.42415E-05	0.00023882	Yes	OK	1558.929722	1248.863446	1868.995998	1180.502909	1346.826883	1219.260546	1577.929233	2327.985467	1701.073294
ENSG00000253537	PCDHGA7	chr5:141382739-141512979	+	protein_coding	0.578020856	0.195620839	2.954802048	0.003128697	0.013358847	Yes	OK	130.7390749	104.4691874	157.0089624	90.32317634	108.8344956	114.2498901	168.4720243	167.8683398	134.686523
ENSG00000100139	MICALL1	chr22:37905657-37942822	+	protein_coding	0.576866055	0.12675509	4.551028734	5.33843E-06	4.31372E-05	Yes	OK	2611.487418	2094.573766	3128.40107	2095.287637	2023.041213	2165.392449	2608.094234	3816.629235	2960.479743
ENSG00000160326	SLC2A6	chr9:133471095-133479137	-	protein_coding	0.5768301	0.204459976	2.821237249	0.004783881	0.019172797	Yes	OK	426.9608376	341.5865955	512.3350797	361.2927054	345.7095743	317.757507	366.4036375	715.8159395	454.7856621
ENSG00000147536	GINS4	chr8:41529206-41545046	+	protein_coding	0.576745457	0.118275281	4.876297489	1.08096E-06	9.81928E-06	Yes	OK	456.8721251	367.0880069	546.6562433	366.5440528	385.7222565	348.9977113	563.4146385	519.4416552	557.1124361
ENSG00000163431	LMOD1	chr1:201896452-201946588	-	protein_coding	0.576286935	0.073330637	7.858747139	3.87995E-15	8.4588E-14	Yes	OK	3352.078495	2691.17156	4012.98543	2685.539092	2720.062137	2667.91345	4174.055508	4019.338174	3845.562609
ENSG00000198121	LPAR1	chr9:110873263-111038458	-	protein_coding	0.576158668	0.093987086	6.130189713	8.77743E-10	1.19205E-08	Yes	OK	3822.053672	3070.571337	4573.536007	3103.54635	3076.975262	3031.192398	5055.08134	4024.089164	4641.437517
ENSG00000184207	PGP	chr16:2211997-2214807	-	protein_coding	0.576077163	0.183233739	3.14394699	0.001666856	0.007772367	Yes	OK	617.5893339	494.5212655	740.6574023	493.6266614	494.5567521	495.3803831	568.9383115	1011.96103	641.0728661
ENSG00000100297	MCM5	chr22:35400063-35425430	+	protein_coding	0.575651498	0.120830826	4.764111269	1.89688E-06	1.65199E-05	Yes	OK	2394.216979	1921.13003	2867.303927	1894.686164	1862.19023	2006.513696	2436.860373	3430.215321	2734.836088
ENSG00000119632	IFI27L2	chr14:94127779-94130253	-	protein_coding	0.575391414	0.183650286	3.133082051	0.001729811	0.008029522	Yes	OK	677.2045475	542.4925584	811.9165366	491.5261224	567.3798338	568.5717189	602.080349	1091.144209	742.5250522
ENSG00000049130	KITLG	chr12:88492793-88580851	-	protein_coding	0.575061858	0.133651957	4.302681901	1.68743E-05	0.000124776	Yes	OK	2204.589944	1772.401381	2636.778507	1787.558676	1714.143306	1815.502161	3208.333358	2098.354247	2603.647916
ENSG00000007866	TEAD3	chr6:35473597-35497076	-	protein_coding	0.574469489	0.143221181	4.011065147	6.04454E-05	0.000396703	Yes	OK	1989.466115	1596.818362	2382.113868	1607.962593	1582.101455	1600.391039	1972.871847	3051.719724	2121.750031
ENSG00000172340	SUCLG2	chr3:67360460-67654614	-	protein_coding	0.572655673	0.179960745	3.182114366	0.001462041	0.006907345	Yes	OK	954.6796986	768.8837613	1140.475636	749.8924175	788.2498396	768.5090267	1432.472512	760.1585198	1228.795876
ENSG00000174516	PELI3	chr11:66466327-66477337	+	protein_coding	0.572622603	0.171480941	3.339278413	0.000839963	0.004258022	Yes	OK	352.4455199	282.2902248	422.600815	270.969529	276.8877609	299.0133844	332.3409878	530.5273003	404.9341569
ENSG00000058056	USP13	chr3:179652755-179789401	+	protein_coding	0.572513694	0.089627942	6.387669743	1.68433E-10	2.4448E-09	Yes	OK	1254.391698	1008.250191	1500.533206	971.4992804	1057.135064	996.1162297	1530.057401	1509.231395	1462.310821
ENSG00000158470	B4GALT5	chr20:49632945-49713878	-	protein_coding	0.571825332	0.074193848	7.70717983	1.28628E-14	2.74252E-13	Yes	OK	3132.256303	2520.186207	3744.3264	2498.591122	2590.421046	2471.546452	3755.176978	3606.001978	3871.800243
ENSG00000177469	CAVIN1	chr17:42402452-42423517	-	protein_coding	0.571705282	0.10021146	5.704989075	1.16351E-08	1.38477E-07	Yes	OK	59737.99327	48051.66872	71424.31783	47546.75019	47487.05125	49121.20472	63005.77524	85270.78196	65996.39628
ENSG00000161980	POLR3K	chr16:46407-53628	-	protein_coding	0.570541387	0.15468494	3.688409397	0.00022566	0.001311108	Yes	OK	200.3700667	161.2712347	239.4688987	171.1939272	152.8484461	159.7713308	226.47059	247.0515189	244.8845873
ENSG00000107404	DVL1	chr1:1335276-1349350	-	protein_coding	0.570500999	0.158116068	3.608115267	0.000308429	0.001738613	Yes	OK	2322.418075	1867.446782	2777.389369	1822.217569	1825.378563	1954.744214	2164.359175	3688.352485	2479.456446
ENSG00000135047	CTSL	chr9:87725519-87731393	+	protein_coding	0.569539034	0.067619839	8.422661747	3.68026E-17	9.13681E-16	Yes	OK	5646.813255	4546.403862	6747.222648	4442.639952	4755.107154	4441.464479	6810.688719	6645.052394	6785.926832
ENSG00000177606	JUN	chr1:58780788-58784327	-	protein_coding	0.568123054	0.110433533	5.144479554	2.68263E-07	2.67127E-06	Yes	OK	4365.837608	3515.318504	5216.356712	3474.291481	3437.089402	3634.57463	4487.063641	6154.116683	5007.88981
ENSG00000189077	TMEM120A	chr7:75986837-75994659	-	protein_coding	0.566607706	0.153589624	3.68910146	0.000225047	0.001308471	Yes	OK	675.1603665	542.8789323	807.4418007	556.6428309	580.1838921	491.810074	689.5385037	1010.377366	722.4095326
ENSG00000171488	LRRC8C	chr1:89633072-89769903	+	protein_coding	0.566193433	0.149733821	3.781332965	0.000155991	0.0009364	Yes	OK	403.9559879	326.3328075	481.5791682	303.5278833	334.5060233	340.9645159	572.6207601	408.5852044	463.5315403
ENSG00000162434	JAK1	chr1:64833229-64966504	-	protein_coding	0.566060874	0.06819354	8.300799104	1.03413E-16	2.47771E-15	Yes	OK	8586.309242	6924.583076	10248.03541	6912.873798	7001.419134	6859.456295	10736.17895	9657.180529	10350.74675
ENSG00000135622	SEMA4F	chr2:74654228-74683853	+	protein_coding	0.56567702	0.151523835	3.733254372	0.000189022	0.001113164	Yes	OK	379.1139059	304.6630683	453.5647435	319.2819257	307.2973994	287.4098799	421.6403667	546.3639361	392.6899275
ENSG00000068001	HYAL2	chr3:50317790-50322906	-	protein_coding	0.565459695	0.182340511	3.101119398	0.001927905	0.008812364	Yes	OK	649.7468908	522.6972587	776.7965229	521.9839377	487.3544693	558.753369	608.5246341	1056.30361	665.5613248
ENSG00000178695	KCTD12	chr13:76880166-76886390	-	protein_coding	0.565056104	0.122010557	4.631206657	3.63541E-06	3.02216E-05	Yes	OK	2185.783592	1764.534754	2607.03243	1729.793854	1766.159793	1797.650615	3073.003372	2142.696828	2605.397091
ENSG00000172061	LRRC15	chr3:194355247-194369743	-	protein_coding	0.564724023	0.072524692	7.786644923	6.8812E-15	1.48836E-13	Yes	OK	10270.36723	8283.631674	12257.10279	8254.06794	8183.393766	8413.433317	11651.26743	13245.76221	11874.27872
ENSG00000167775	CD320	chr19:8302127-8308356	-	protein_coding	0.564650579	0.173116334	3.261682866	0.00110753	0.005453168	Yes	OK	880.2870035	708.91944	1051.654567	709.9821768	655.4077346	761.3684086	789.8852285	1387.289299	977.7891736
ENSG00000170791	CHCHD7	chr8:56211686-56218798	+	protein_coding	0.563839646	0.179810482	3.13574404	0.001714187	0.007963894	Yes	OK	398.0409354	322.4171245	473.6647463	323.4830036	315.2999358	328.4684342	565.2558628	330.9856888	524.7526871
ENSG00000141753	IGFBP4	chr17:40443461-40457731	+	protein_coding	0.562864934	0.218958181	2.570650392	0.010150774	0.03608677	Yes	OK	28848.6363	23290.53632	34406.73629	23237.21252	22948.0735	23686.32293	28299.61763	44779.67147	30140.91976
ENSG00000125746	EML2	chr19:45606894-45645629	-	protein_coding	0.562620917	0.126380873	4.451788495	8.5158E-06	6.63867E-05	Yes	OK	494.9130845	398.8920387	590.9341304	391.7505206	372.9181982	432.0073971	584.5887181	647.7184054	540.4952677
ENSG00000213366	GSTM2	chr1:109668022-109709551	+	protein_coding	0.562109859	0.219096192	2.565584795	0.010300204	0.036549824	Yes	OK	94.28983137	76.07203757	112.5076252	82.97128989	64.02029154	81.22453128	99.42611268	123.5257595	114.5710033
ENSG00000105355	PLIN3	chr19:4838341-4867768	-	protein_coding	0.561753731	0.124494211	4.512287973	6.4132E-06	5.13182E-05	Yes	OK	7715.010137	6229.607335	9200.412939	6016.993921	6244.379186	6427.448898	7633.715985	11424.54909	8542.973746
ENSG00000103647	CORO2B	chr15:68578969-68727806	+	protein_coding	0.561351824	0.109327485	5.134590107	2.8276E-07	2.80037E-06	Yes	OK	705.4377996	569.8114775	841.0641217	566.0952563	619.3963206	523.9428556	846.04257	831.4233811	845.726414
ENSG00000176438	SYNE3	chr14:95407266-95475836	-	protein_coding	0.560460459	0.10340739	5.419926537	5.96235E-08	6.4807E-07	Yes	OK	742.7190483	599.4647642	885.9733323	594.4525326	596.9892186	606.9525414	882.8670561	940.6961683	834.3567725
ENSG00000109084	TMEM97	chr17:28319095-28328685	+	protein_coding	0.559777501	0.168523591	3.321656617	0.000894847	0.004494717	Yes	OK	292.8055696	237.6438324	347.9673068	231.0592883	240.8763469	240.995862	412.4342452	267.6391455	363.8285297
ENSG00000103061	SLC7A6OS	chr16:68284503-68310946	-	protein_coding	0.559508173	0.186324055	3.002876757	0.002674407	0.011713875	Yes	OK	149.1537583	120.009719	178.2977975	105.0269492	141.644895	113.3573129	169.3926364	193.2069571	172.2937989
ENSG00000169499	PLEKHA2	chr8:38901235-38973909	+	protein_coding	0.559446413	0.094248222	5.935882921	2.92268E-09	3.73795E-08	Yes	OK	1174.978783	951.1027633	1398.854802	970.4490109	917.0906763	965.7686027	1460.090877	1306.522456	1429.951072
ENSG00000088832	FKBP1A	chr20:1369878-1393172	-	protein_coding	0.559115435	0.067228143	8.316687211	9.04561E-17	2.17995E-15	Yes	OK	7514.829048	6077.946001	8951.712096	6345.728272	5927.478743	5960.630988	9233.739909	8692.729407	8928.666971
ENSG00000123353	ORMDL2	chr12:55817919-55821879	+	protein_coding	0.55909506	0.149712421	3.734460084	0.000188118	0.001108639	Yes	OK	387.4088268	312.301022	462.5166317	331.8851596	295.2935947	309.7243116	418.8785303	555.8659176	412.8054472
ENSG00000091651	ORC6	chr16:46689643-46698394	+	protein_coding	0.558616693	0.123236836	4.532871092	5.81873E-06	4.68344E-05	Yes	OK	492.3559783	398.3292275	586.382729	392.8007901	412.1306268	390.0562656	649.9521811	566.9515627	542.2444433
ENSG00000126391	FRMD8	chr11:65386599-65413525	+	protein_coding	0.557918497	0.177545809	3.142391816	0.001675736	0.00781156	Yes	OK	1351.869103	1092.144487	1611.593718	1075.47596	1061.136332	1139.82117	1227.176002	2226.630998	1380.974155
ENSG00000183617	MRPL54	chr19:3762664-3768575	+	protein_coding	0.556490183	0.154084154	3.611599051	0.000304315	0.001716594	Yes	OK	516.5220992	416.8218727	616.2223257	397.0018681	400.9270758	452.5366743	521.0664794	761.7421834	565.8583142
ENSG00000156381	ANKRD9	chr14:102501760-102509799	-	protein_coding	0.556448291	0.187769533	2.963464213	0.003041974	0.013049491	Yes	OK	634.9430201	512.6541664	757.2318788	471.571002	528.1674052	538.2240919	541.3199468	1016.71202	713.6636544
ENSG00000123737	EXOSC9	chr4:121801317-121817021	+	protein_coding	0.556143914	0.124537821	4.4656662781	7.98213E-06	6.25945E-05	Yes	OK	1106.968289	897.4433357	1316.493243	874.8744871	957.9036122	859.5519079	1449.043531	1087.976881	1412.459316
ENSG00000043143	JADE2	chr5:134524312-134583230	+	protein_coding	0.55601698	0.117842011	4.718325642	2.37794E-06	2.03329E-05	Yes	OK	1120.333716	905.4993284	1335.168104	885.377182	932.2954956	898.8253076	1174.701109	1550.406648	1280.396556
ENSG00000170430	MGMT	chr10:129467184-129768007	+	protein_coding	0.555998935	0.164332183	3.383384347	0.000715984	0.003689759	Yes	OK	401.7282794	324.3363863	479.1201725	358.1418969	310.498414	304.368848	395.8632264	595.4575072	446.039784
ENSG00000062822	POLD1	chr19:50384204-50418016	+	protein_coding	0.555215822	0.168230258	3.30033271	0.000965703	0.004824117	Yes	OK	712.7652918	575.9758373	849.5547463	573.4471428	576.9828775	577.4974916	637.984223	1099.062527	811.6174894
ENSG00000113119	TMCO6	chr5:140639427-140645408	+	protein_coding	0.555199683	0.203065594	2.734090357	0.006255288	0.024017269	Yes	OK	159.8295701	128.6822188	190.9769213	141.7863815	126.4400758	117.8201992	164.7895756	245.4678554	162.673333
ENSG00000162542	TMCO4	chr1:19682213-19799945	-	protein_coding	0.55473644	0.114211648	4.857091647	1.19122E-06	1.07439E-05	Yes	OK	582.5996501	471.520916	693.6783842	477.872619	484.1534548	452.5366743	635.2223866	726.9015846	718.9111813
ENSG00000154380	ENAH	chr1:225486835-225653142	-	protein_coding	0.554301758	0.092939682	5.964102169	2.45983E-09	3.18058E-08	Yes	OK	9126.442938	7396.490785	10856.39509	7371.841566	7327.122367	7490.508423	11958.75189	9348.36613	11262.06725

ENSG00000074855	ANO8	chr19:17323223-17334829	-	protein_coding	0.554126468	0.189761995	2.920113	0.003499045	0.014662494	Yes	OK	453.7101828	366.4198321	541.0005334	357.0916274	352.1116035	390.0562656	398.6250629	728.4852482	495.8912893
ENSG00000147872	PLIN2	chr9:19108375-19149290	-	protein_coding	0.553494375	0.080453558	6.879675521	5.9989E-12	1.00963E-10	Yes	OK	1966.979556	1594.808123	2339.15099	1565.951813	1579.700694	1638.771862	2421.209966	2256.720606	2339.522397
ENSG00000084070	SMAP2	chr1:40344850-40423326	+	protein_coding	0.55206204	0.102001036	5.412317945	6.22141E-08	6.74601E-07	Yes	OK	940.3824528	761.7413434	1119.023562	774.0486158	757.8402011	753.3352132	1032.926837	1209.918977	1114.224872
ENSG00000128805	ARHGAP22	chr10:48446034-48656265	-	protein_coding	0.55083145	0.120765603	4.561161773	5.08714E-06	4.11875E-05	Yes	OK	853.7023215	691.3044322	1016.100211	676.373553	667.4115393	730.1282042	941.786234	1175.078379	931.4360196
ENSG00000180448	ARHGAP45	chr19:1065923-1086628	+	protein_coding	0.550816965	0.216169721	2.548076405	0.010831874	0.038039824	Yes	OK	102.7411024	83.05867168	122.4235331	91.37344583	71.22257434	86.57999488	107.7116221	144.113386	115.4455912
ENSG00000128016	ZFP36	chr19:39406813-39409412	+	protein_coding	0.55029083	0.102212198	5.383807804	7.29264E-08	7.8353E-07	Yes	OK	1514.501711	1227.413163	1801.590259	1165.799136	1258.798982	1257.641369	1670.91106	2014.420078	1719.439638
ENSG00000176834	VSIG10	chr12:118063593-118136026	-	protein_coding	0.548828594	0.137064839	4.004153047	6.22401E-05	0.000407021	Yes	OK	560.2779496	453.8181807	666.7377186	456.8672291	429.736207	474.8511059	585.5093302	790.2481279	624.4556976
ENSG00000177663	IL17RA	chr22:17084954-17115694	+	protein_coding	0.548676634	0.122290184	4.48667764	7.23424E-06	5.73306E-05	Yes	OK	2254.455664	1829.373253	2679.538075	1915.691554	1753.355735	1819.07247	2279.435694	3213.25341	2545.92512
ENSG00000103066	PLA2G15	chr16:68245304-68261062	+	protein_coding	0.548604194	0.106905208	5.131688219	2.87155E-07	2.84219E-06	Yes	OK	974.8325622	790.6938922	1158.971232	765.6464599	775.4457813	830.9894354	1048.577244	1259.012548	1169.323904
ENSG00000164574	GALNT10	chr5:154190730-154420984	+	protein_coding	0.548477336	0.070467719	7.783384292	7.06097E-15	1.52325E-13	Yes	OK	7473.76028	6068.470208	8879.050351	6102.06575	5988.29802	6115.046855	8416.236316	9441.802282	8779.112455
ENSG00000144749	LRIG1	chr3:66378797-66501263	-	protein_coding	0.548304046	0.154494434	3.549021362	0.000386666	0.002122921	Yes	OK	465.7862534	377.056076	554.5164308	388.5997121	387.3227638	355.2457522	479.6389325	688.8936586	495.0167015
ENSG00000120693	SMAD9	chr13:36844831-36920765	-	protein_coding	0.548246262	0.106420668	5.151689714	2.5815E-07	2.57524E-06	Yes	OK	827.1014519	672.7598967	981.4430071	705.7810988	661.8097638	650.6888275	1062.386426	913.7738874	968.1687077
ENSG00000074047	GLI2	chr2:120735623-120992653	+	protein_coding	0.54724853	0.153661295	3.561394754	0.00036889	0.002036877	Yes	OK	1075.119658	872.1674417	1278.071875	852.8188277	828.2625218	935.4209756	1024.641328	1628.006163	1181.568134
ENSG00000111145	ELK3	chr12:96194382-96269835	+	protein_coding	0.547207387	0.102823209	5.321827539	1.0273E-07	1.08532E-06	Yes	OK	3122.554854	2539.218745	3705.890962	2627.77427	2441.573869	2548.308097	4149.19898	3199.000438	3769.473469
ENSG00000102359	SRPX2	chrX:100644166-100675788	+	protein_coding	0.546934025	0.093972464	5.820152027	5.87941E-09	7.18983E-08	Yes	OK	985.2778321	800.4321622	1170.123502	801.3556226	769.8440058	830.0968581	1147.082744	1203.584323	1159.703438
ENSG00000160285	LSS	chr21:46188141-46228824	-	protein_coding	0.546763348	0.123755295	4.418100628	9.95721E-06	7.64082E-05	Yes	OK	3812.420455	3096.576022	4528.264888	2973.312933	3074.574501	3241.840633	3814.096156	5533.320559	4237.377948
ENSG00000110880	CORO1C	chr12:108645109-108731596	-	protein_coding	0.546481219	0.071804184	7.610715585	2.72582E-14	5.67938E-13	Yes	OK	16482.79257	13399.44714	19566.13799	13127.31838	13453.86427	13617.15878	20895.13407	18099.69109	19703.58881
ENSG00000197386	HTT	chr4:3074681-3243960	+	protein_coding	0.545527324	0.089459818	6.098015133	1.07394E-09	1.43949E-08	Yes	OK	3015.000024	2450.099041	3579.901006	2333.698253	2480.786297	2535.812015	3268.173148	3875.224787	3596.305082
ENSG00000158122	AAED1	chr9:96639577-96655303	-	protein_coding	0.54543069	0.120795048	4.515339824	6.32153E-06	5.06831E-05	Yes	OK	711.6248819	579.9257761	843.3239877	573.4471428	582.584653	583.7455325	904.0411357	718.9832667	906.9475608
ENSG00000127666	TICAM1	chr19:4815932-4831704	-	protein_coding	0.544520917	0.122236085	4.454665887	8.40239E-06	6.56711E-05	Yes	OK	690.0778121	560.2516704	819.9039539	576.5979513	548.974	555.1830599	731.8866628	940.6961683	787.1290306
ENSG00000154065	ANKRD29	chr18:23598926-23662885	-	protein_coding	0.544440499	0.177499043	3.067286956	0.002160114	0.009719922	Yes	OK	144.8643768	118.2971184	171.4316352	131.2836865	112.0355102	111.5721583	177.6781458	159.9500219	176.666738
ENSG00000092470	WDR76	chr15:43826963-43868419	+	protein_coding	0.544075473	0.122572278	4.438813424	9.04562E-06	7.01267E-05	Yes	OK	625.3851993	509.6992368	741.0711618	479.973158	515.3633469	533.7612055	811.9799202	647.7184054	763.5151597
ENSG00000110697	PITPNM1	chr11:67491768-67506263	-	protein_coding	0.543483339	0.155548229	3.493986037	0.000475866	0.002558051	Yes	OK	1515.886646	1232.072373	1799.70092	1150.045094	1233.190866	1312.981159	1399.330475	2315.316158	1684.456126
ENSG00000172375	C2CD2L	chr11:119102198-119118544	+	protein_coding	0.542710485	0.153242425	3.541515894	0.000397835	0.002177706	Yes	OK	396.5757168	321.4955725	471.6558612	299.3268053	346.509828	318.6500842	437.2907734	570.1188899	407.5579203
ENSG00000213337	ANKRD39	chr2:96836611-96858095	-	protein_coding	0.542205072	0.199009409	2.724519788	0.006439507	0.02467853	Yes	OK	103.5258319	84.05783865	122.9938252	86.12209837	84.82688629	81.22453128	118.7589679	133.027741	117.1947668
ENSG00000152642	GPD1L	chr3:32105689-32168713	+	protein_coding	0.542116248	0.133697172	4.054807134	5.01757E-05	0.000336686	Yes	OK	325.7817922	265.5739717	385.9896128	251.0144087	283.2897901	262.4177164	398.6250629	357.9079697	401.4358056
ENSG00000085840	ORC1	chr1:52372829-52404459	-	protein_coding	0.542071045	0.180645513	3.000744583	0.002693204	0.011780085	Yes	OK	243.0107336	196.7115135	289.3099536	182.7468917	205.6651866	201.7224623	264.2156883	365.8262877	237.8878848
ENSG00000130304	SLC27A1	chr19:17468769-17506168	+	protein_coding	0.541968136	0.147902828	3.664352753	0.000247965	0.001423118	Yes	OK	1107.714037	900.6624205	1314.765654	915.8349973	857.8719066	928.2803574	1057.783365	1653.344781	1233.168815
ENSG00000189306	RRP7A	chr22:42509968-42519802	-	protein_coding	0.541491763	0.144189296	3.75542276	0.000173049	0.001026816	Yes	OK	789.2435465	641.4791333	937.0079598	578.6984903	628.9993644	716.7395452	788.9646164	1121.233817	900.8254462
ENSG00000049541	RFC2	chr7:74231499-74254458	-	protein_coding	0.541134626	0.108625971	4.981632119	6.30502E-07	5.95073E-06	Yes	OK	724.614777	590.3796135	858.8499405	538.7882495	613.7945451	618.5560459	875.5021589	820.337736	880.7099265
ENSG00000177105	RHOG	chr11:3826978-3840983	-	protein_coding	0.541040483	0.168892084	3.203468579	0.001357829	0.006492393	Yes	OK	1486.551813	1209.359325	1763.7443	1125.888896	1204.381735	1297.807346	1389.203741	2380.246365	1521.782793
ENSG00000197586	ENTPD6	chr20:25195693-25226729	+	protein_coding	0.54015969	0.136698173	3.951477022	7.76703E-05	0.000497066	Yes	OK	1484.306727	1208.013449	1760.600006	1251.921235	1209.98351	1162.135601	1533.739849	2196.54139	1551.518778
ENSG00000166825	ANPEP	chr15:89784889-89815401	-	protein_coding	0.53988233	0.099577401	5.421735502	5.90232E-08	6.42966E-07	Yes	OK	26556.89161	21642.74281	31471.04041	21751.08119	21581.24028	21595.90697	27770.26564	37162.24964	29480.60596
ENSG00000085999	RAD54L	chr1:46247688-46278473	+	protein_coding	0.539878314	0.15412134	3.502943283	0.000460147	0.002485732	Yes	OK	265.8114278	215.9287171	315.6941385	200.601473	232.0735568	215.1111213	271.5805856	348.4059883	327.0958416
ENSG00000151929	BAG3	chr10:119651370-119677819	+	protein_coding	0.539445925	0.114517052	4.710616593	2.46968E-06	2.10627E-05	Yes	OK	3574.609441	2911.898192	4237.32069	2772.71146	2927.327831	3035.655284	3785.557179	5067.723465	3858.681426
ENSG00000131016	AKAP12	chr6:151239999-151358557	+	protein_coding	0.539068822	0.100789449	5.348464833	8.87034E-08	9.42089E-07	Yes	OK	3332.41959	2718.935689	3945.903491	2797.917927	2670.446411	2688.442728	4325.035902	3381.12175	4131.552823
ENSG00000170364	SETMAR	chr3:4303304-4317567	+	protein_coding	0.53849588	0.142111038	3.789261472	0.000151096	0.000909337	Yes	OK	247.5086152	201.4320547	293.5851756	192.1993171	211.2669621	200.829885	289.9928287	305.6470715	285.1156266
ENSG00000196449	YRDC	chr1:37802944-37808185	-	protein_coding	0.537340739	0.153234058	3.506666502	0.000453757	0.00245605	Yes	OK	307.6483609	249.987169	365.3095529	248.9138697	268.0849708	232.9626666	321.2936419	429.172831	345.4621857
ENSG00000158882	TOMM40L	chr1:161225939-161230744	+	protein_coding	0.536888562	0.139385605	3.851822168	0.000117242	0.000723316	Yes	OK	275.3596482	224.1374634	326.581833	210.0538985	232.0735568	230.2849348	309.3256839	346.8223247	323.5974904
ENSG00000157193	LRP8	chr1:53242364-53328070	-	protein_coding	0.536649279	0.127898171	4.195910509	2.71778E-05	0.000192738	Yes	OK	491.7129355	402.0842994	581.3415717	392.8007901	430.5364606	382.9156474	613.1276949	509.9396737	620.9573464
ENSG00000104886	PLEKHJ1	chr19:2230084-2237704	-	protein_coding	0.536338524	0.159873571	3.35476665	0.00079432	0.004054598	Yes	OK	862.5211152	702.3622033	1022.680027	665.8708581	753.0386792	688.1770727	849.7250186	1327.110083	891.2049802
ENSG00000132661	NXT1	chr20:23350736-23354777	+	protein_coding	0.536054616	0.165592217	3.237196919	0.001207101	0.00586795	Yes	OK	319.2444846	259.4758685	379.0131007	247.8636002	252.0798979	278.4841072	316.6905811	468.7644206	351.5843003
ENSG00000235173	HGH1	chr8:144137769-144140843	+	protein_coding	0.535837233	0.132437639	4.045958812	5.21094E-05	0.000348519	Yes	OK	707.2597843	576.1160133	838.4035553	561.8941784	620.1965743	546.2572873	715.315644	970.7857764	829.1092456
ENSG00000101974	ATP11C	chrX:139726346-139945276	-	protein_coding	0.535691744	0.136462972	3.925546506	8.6533E-05	0.000549512	Yes	OK	2428.319317	1984.258184	2872.38045	1997.612574	1940.615087	2014.546891	3399.820686	2188.623072	3028.697592
ENSG00000156515	HK1	chr10:69269984-69401884	+	protein_coding	0.535679231	0.072693289	7.369032792	1.7187E-13	3.35984E-12	Yes	OK	6879.835056	5615.157347	8144.512765	5587.433699	5648.190221	5609.848122	7949.485954	8746.573969	7737.478371
ENSG00000101665	SMAD7	chr18:48919853-48950711	-	protein_coding	0.534467372	0.108293602	4.935355022	8.00051E-07	7.41071E-06	Yes	OK	1266.710188	1033.19125	1500.229127	1031.364641	1020.323396	1047.885711	1379.077007	1713.523997	1408.086377
ENSG00000239672	NME1	chr17:51153536-51162428	+	protein_coding	0.534447525	0.125050446	4.273855404	1.92122E-05	0.000139926	Yes	OK	1265.75716	1032.45075	1499.06357	1063.922996	1012.32086	1021.108393	1306.348647	1795.874503	1394.96756
ENSG00000253626	EIF5AL1	chr10:79512601-79516440	+	protein_coding	0.533511035	0.21206473	2.515793341	0.01187648	0.041025574	Yes	OK	275.6286165	224.1550952	327.1021378	224.7576714	212.0672157	235.6403984	236.5973237	456.0951119	288.6139779
ENSG00000167700	MFSD3	chr8:144509074-144511213	+	protein_coding	0.53346682	0.202529152	2.634024851	0.008437931	0.030974208	Yes	OK	248.354962	201.8718833	294.8380407	191.1490476	198.4629038	216.0036986	218.1850806	394.3322322	271.9968095
ENSG00000160256	FAM207A	chr21:44940010-44976989	+	protein_coding	0.53046069	0.164151555	3.231530082	0.001231294	0.005971448	Yes	OK	246.2118032	200.6687531	291.7548533	205.8528205	202.464172	193.6892669	242.1209966	343.6549975	289.4885657
ENSG00000100714	MTHFD1	chr14:64388031-64463457	+	protein_coding	0.529845996	0.082711366	6.405963582	1.49423E-10	2.17847E-09	Yes	OK	2086.386943	1708.234092	2464.539794	1809.614335	1686.134428	1628.953512	2522.477303	2421.421618	2449.720461
ENSG00000100599	RIN3	chr14:92513774-92688994	+	protein_coding	0.528687521	0.135812423	3.89277733	9.91031E-05	0.000619771	Yes	OK	1562.612249	1277.926279	1847.298219	1298.133092	1231.590358	1304.055387	1523.613116	2280.475559	1737.805982
ENSG00000143774	GUK1	chr1:228139962-228148984	+	protein_coding	0.528368991	0.116989177	4.516392058	6.29022E-06	5.04812E-05	Yes	OK	4283.622826	3505.912477	5061.333175	3381.867765	3545.923898	3589.945767	4247.704481	6054.345878	4881.949166
ENSG00000103365	GGA2	chr16:23463542-23521995	-	protein_coding	0.526622478	0.104229196	5.052542821	4.35967E-07	4.21548E-06	Yes	OK	1038.726193	850.2043457	1227.24804	803.4561616	925.0932128	822.0636627	1248.350081	1285.934829	1147.459209
ENSG00000164211	STARD4	chr5:111496033-111512590	-	protein_coding	0.526353706	0.168405431	3.125515034	0.00177494	0.0082043	Yes	OK	651.3825529	535.0525077	767.7125982	532.4866326	566.5795801	506.0913103	931.6595003	551.1149269	820.3633675
ENSG00000109066	TMEM104	chr17:74776483-74839779	+	protein_coding	0.526126602	0.143649994	3.662559155	0.000249708	0.001431002	Yes	OK	1512.942426	1238.378158	1787.506694	1248.770426	1282.806592	1183.557456	1498.756587	2269.389914	1594.373581
ENSG00000073921	PICALM	chr11:85957684-86069882	-	protein_coding	0.525850172	0.130167098	4.039808682	5.34948E-05	0.000356199	Yes	OK	9612.243408	7881.521527	11342.96534	8034.561616	7971.32655	7638.676249	13178.56299	8509.024431	12341.30861
ENSG00000151474	FRMD4A	chr10:13643706-14462142	-	protein_coding	0.525799128	0.147593601	3.562479159	0.000367369	0.002029841	Yes	OK	645.247304	527.4172417	763.0773662	496.7774699	532.9689271	552.5053281	701.5064617	942.2798319	645.4458051
ENSG00000037042	TUBG2	chr17:42659305-42667006	+	protein_coding	0.52529588	0.153106553	3.430917026	0.000601545	0.003156335	Yes	OK	385.6669777	315.2716749	456.0622805	302.4776138	311.2986676	332.0387432	370.0860861	533.6946275	464.4061281
ENSG00000089639	GMIP	chr19:19629476-19643667	-	protein_coding	0.524585711	0.161416734	3.249884307	0.00115452	0.005650727	Yes	OK	377.4900285	308.4671031	446.5129538	291.9749189	288.8915656	344.534825	386.6571049	547.9475997	404.9341569
ENSG00000176463	SLCO3A1	chr15:91853695-92172435	+	protein_coding	0.524146359	0.145460365	3.603362057	0.000314127	0.00176831	Yes	OK	325.5576912	266.7710331	384.3443493	282.5224934	231.2733032	286.5173026	376.5303712	419.6708495	356.8318272
ENSG00000121904	CSMD2	chr1:33513999-34165842	-	protein_coding	0.523915149	0.129708598	4.039170557	5.36406E-05	0.000357025	Yes	OK	1585.862109	1299.514259	1872.20996	1350.646567	1272.403294	1275.492914	1580.691069	2278.891896	1757.046914
ENSG00000168785	TSPAN5	chr4:98470367-98658629	-	protein_coding	0.523750511	0.08810728	5.944463501	2.77364E-09	3.55561E-08	Yes	OK	1294.227079	1062.729086	1525.725072	1082.827847	1061.936586	1043.422825	1561.358214	1453.803169	1562.013832
ENSG00000134107	BHLHE40	chr3:4979116-4985323	+	protein_coding	0.5236209	0.120610313	4.341427254	1.4156E-05	0.000106106	Yes	OK	1080.663868	885.167045	1276.160691	895.8798769	863.4736821	896.1475758	1159.050702	1504.480404	1164.950965
ENSG00000173846	PLK3	chr1:44800225-44805990	+	protein_coding	0.523203861	0.132371056	3.95255485	7.73212E-05	0.000495217	Yes	OK	475.1829426	388.907354	561.4585312	381.2478257	417.7324023	367.7418339	497.1305634	631.8817696	555.3632605
ENSG00000034510	TMSB10	chr2:84905625-84906675	+	protein_coding	0.523203252	0.0952525	5.492803371	3.95603E-08	4.38789E-07	Yes	OK	36715.81831	30127.84236	43303.79425	29966.28915	29256.47298	31160.76496	39434.42164	50653.4797	39823.48142
ENSG00000115541	HSPE1	chr2:197499994-197503457	+	protein_coding	0.523151044	0.154329462	3.389832624	0.000699353	0.003611971	Yes	OK	253.2672811	207.9326921	298.6018702	189.0485086	192.8611283	241.8884393	295.5165016	289.8104357	310.4786732
ENSG00000153395	LPCAT1	chr5:1456480-1523977	-	protein_coding	0.521773959	0.083998881	6.211677467	5.24219E-10	7.25097E-09	Yes	OK	1902.602551	1561.030019	2244.175083	1486.131332	1603.708303	1593.250421	2193.818764	2346.98943	2191.717056
ENSG00000262919	CCNQ	chrX:153587919-153600045	-	protein_coding	0.521248293	0.167518687	3.111582971	0.001860872	0.008558242	Yes	OK	431.5572512	353.5754534	509.5390491	391.7505206	305.6968921	363.2789476	435.4495491	636.6327603	456.5348378
ENSG00000132361	CLUH	chr17:2689386-2712663	-	protein_coding	0.521133172	0.146650406	3.553574695	0.000380033	0.002092789	Yes	OK	1735.58893	1423.950688	2047.227171	1451.472438	1347.627137	1472.75249	1648.816369	2609.877585	1882.987559
ENSG00000113648	H2AFY	chr5:135333900-135399914	-	protein_coding	0.520767997	0.083640613	6.226257562	4.77709E-10	6.61876E-09	Yes	OK	2444.119772	2006.300045	2881.9395	1932.495866	2118.271396	1968.132873	2854.818291	3034.299425	2756.700783
ENSG00000117152	RGS4	chr1:163068775-163076802	+	protein_coding	0.520564865	0.121689551	4.277810706	1.8874E-05	0.00013789	Yes	OK	1188.457491	977.8828973	1399.032085	972.5495499	973.9086851	987.1904571	1542.025359	1148.156098	1506.9148
ENSG00000134375	TIMM17A	chr1:201955491-201970661	+	protein_coding	0.52020198	0.097871985	5.315126464	1.06583E-07	1.12315E-06	Yes	OK	2022.79832	1662.971764	2382.624876	1658.375528	1753.355735	1577.18403	2621.903416	2175.953763	2350.01745
ENSG00000099821	POLRMT	chr19:617224-633604	-	protein_coding	0.519574269	0.188319261	2.759007586	0.005797719	0.022528436	Yes	OK	536.6482525	439.8117757	633.4847294	421.1580664	399.3265685	498.9506921	486.0832176	852.0110076	562.359963
ENSG00000182718	ANXA2	chr15:60347134-60402883	-	protein_coding	0.519012443	0.065424323	7.933019722	2.1388E-15	4.75097E-14	Yes	OK	37606.19854	30911.85306	44300.54401	31413.56051	30992.22313	30329.77553	43960.15099	47228.01537	41713.46568
ENSG00000110921	MVK	chr12:109573255-109598117	+	protein_coding	0.51866005	0.150111774	3.45515902	0.000549968	0.002913575	Yes	OK	347.8214305	285.2596473	410.3832137	248.9138697	325.7032332	281.161839	362.7211889	435.5074853	432.9209668
ENSG00000136122	BORA	chr13:72727749-72756198	+	protein_coding	0.518255539	0.199895128	2.592637168	0.009524319	0.034326756	Yes	OK	232.7299186	192.4584339	273.0014032	212.1544374	192.0608746	173.1599898	300.1195624	190.03963	328.8450172
ENSG00000132819	RBM38	chr20:57391407-57409333	+	protein_coding	0.517946857	0.196857385	2.631076584	0.008511485	0.031181627	Yes	OK	267.2535742	218.6471048	315.8600435	217.4057849	206.4654402	232.0700894	268.8187491	426.0055038	252.7558776
ENSG00000146909	NOM1	chr7:156949723-156973182	+	protein_coding	0.517681226	0.100043577	5.174557365	2.28452E-07	2.29989E-06	Yes	OK	895.8852317	737.8469508	1053.923513	757.2443039	707.4242215	748.8723268	1060.545202	991.3734029	1109.851933
ENSG00000109971	HSPA8	chr11:123057489-123063230	-	protein_coding	0.517271589	0.057827418	8.94509227	3.71639E-19	1.0281E-17	Yes	OK	24081.28902	19809.85738	28352.72067	20154.67156	20110.37408	19164.52649	28886.96818	28377.66774	27793.52607

ENSG00000106077	ABHD11	chr7:73736094-73738867	-	protein_coding	0.51597062	0.176983313	2.915363092	0.003552749	0.014845965	Yes	OK	189.9643987	156.1354619	223.7933355	149.1382679	164.8522507	154.4158672	183.2018187	232.7985467	255.379641
ENSG00000102547	CAB39L	chr13:49308650-49444126	-	protein_coding	0.513674227	0.186504972	2.754211975	0.005883365	0.022785926	Yes	OK	305.8021054	252.998251	358.6059598	244.7127917	238.475586	275.8063754	426.2434275	253.3861733	396.1882787
ENSG00000131473	ACLY	chr17:41866908-41930542	-	protein_coding	0.513400338	0.067435713	7.613181727	2.67429E-14	5.57907E-13	Yes	OK	10622.31924	8750.104678	12494.5338	8730.890289	8874.812915	8644.610829	11998.33821	13274.26815	12210.99503
ENSG00000169032	MAP2K1	chr15:66386817-66492312	+	protein_coding	0.513380407	0.086989006	5.901670009	3.5984E-09	4.53173E-08	Yes	OK	1334.220856	1100.015276	1568.426435	1127.989435	1056.33481	1115.721583	1585.29413	1528.235358	1591.749817
ENSG00000130706	ADRM1	chr20:62302093-62308862	+	protein_coding	0.512935035	0.13875745	3.696630586	0.00021848	0.001273435	Yes	OK	1585.467085	1304.73921	1866.194959	1259.273121	1311.615723	1343.328786	1586.214742	2345.405766	1666.964369
ENSG00000102977	ACD	chr16:67657512-67660815	-	protein_coding	0.512614582	0.163461962	3.13598696	0.001712768	0.007961639	Yes	OK	471.655898	387.5334959	555.7783002	390.7002511	372.9181982	398.9820382	441.8938341	701.5629673	523.8780993
ENSG00000173068	BNC2	chr9:16409503-16870843	-	protein_coding	0.512596475	0.097479454	5.258507865	1.45229E-07	1.50439E-06	Yes	OK	1090.746102	899.4658145	1282.02639	944.1922736	925.8934664	828.3117036	1278.730283	1251.094231	1316.254657
ENSG00000171206	TRIM8	chr10:102642310-102660680	+	protein_coding	0.51255019	0.127273633	4.027151408	5.64567E-05	0.000373204	Yes	OK	5078.931033	4184.07726	5973.784805	4128.609374	4213.335437	4210.286967	4971.305634	7455.888149	5494.160634
ENSG00000011009	LYPLA2	chr1:23790970-23795539	+	protein_coding	0.512328644	0.169327787	3.025661957	0.002480895	0.010956676	Yes	OK	470.939727	386.8007488	555.0787052	352.8905494	420.1331632	387.3785338	438.2113855	703.1466308	523.8780993
ENSG00000122952	ZWINT	chr10:56357228-56361275	-	protein_coding	0.512182892	0.087805129	5.833177361	5.43817E-09	6.68501E-08	Yes	OK	1182.356031	974.88787	1389.824191	998.8062872	946.7000611	979.1572617	1373.553334	1390.456626	1405.462614
ENSG00000160818	GPATCH4	chr1:156594487-156601496	-	protein_coding	0.511853135	0.11884263	4.306982567	1.65497E-05	0.00012254	Yes	OK	731.2409762	603.7675934	858.7143589	640.6643903	529.7679125	640.8704775	914.1678694	809.2520909	852.7231165
ENSG00000068724	TTC7A	chr2:46916157-47076137	+	protein_coding	0.51168894	0.102621212	4.986190768	6.15813E-07	5.82545E-06	Yes	OK	1586.428117	1306.700919	1866.155314	1269.775816	1258.798982	1391.527959	1697.608813	2065.097312	1835.759817
ENSG00000197948	FCHSD1	chr5:141639302-141651419	-	protein_coding	0.511439063	0.13256741	3.857954705	0.00011434	0.000707	Yes	OK	703.7660489	578.7670085	828.7650894	539.838519	592.1876967	604.2748096	737.4103357	981.8714214	767.013511
ENSG00000112576	CCND3	chr6:41934933-42050357	-	protein_coding	0.511268271	0.112378417	4.549523713	5.37675E-06	4.34042E-05	Yes	OK	1820.848347	1499.881442	2141.815252	1493.483218	1501.275837	1504.885272	1863.319001	2491.102816	2071.023938
ENSG00000119927	GPAM	chr10:112149864-112215377	-	protein_coding	0.510712131	0.124201727	4.111956755	3.9232E-05	0.000270192	Yes	OK	704.5979261	582.4929874	826.7028648	583.9498377	568.1800874	595.3490369	913.2472572	698.3956401	868.4656971
ENSG00000144320	LNPK	chr2:175923892-176002839	-	protein_coding	0.509827268	0.139117477	3.664724788	0.000247605	0.001421546	Yes	OK	2309.063509	1906.832401	2711.294618	1889.434817	1902.202912	1928.859474	3255.284578	2057.178994	2821.420281
ENSG00000104356	POP1	chr8:98117297-98159834	+	protein_coding	0.50883329	0.145450971	3.498314844	0.000468208	0.002523479	Yes	OK	415.8247457	344.2735393	487.3759522	351.8402799	364.1154081	316.8649297	508.1779093	403.8342137	550.1157336
ENSG00000141458	NPC1	chr18:23506184-23586898	-	protein_coding	0.508271834	0.08360608	6.079364494	1.2066E-09	1.60815E-08	Yes	OK	2282.739476	1883.538675	2681.940278	1843.222959	1859.789469	1947.603596	2533.524649	2847.427122	2664.869063
ENSG00000132507	EIF5A	chr17:7306999-7312463	+	protein_coding	0.507686849	0.130142112	3.901018972	9.57886E-05	0.000601098	Yes	OK	8191.289328	6762.573554	9620.005101	6580.988639	6697.322749	7009.409276	8183.321441	12229.05019	8447.643674
ENSG00000166166	TRMT61A	chr14:103529184-103537073	+	protein_coding	0.507350105	0.170752461	2.971260865	0.002965797	0.012772639	Yes	OK	585.1914113	481.9231971	688.4596255	451.6158817	488.9549766	505.198733	533.0344374	894.7699244	637.5745148
ENSG00000042753	AP2S1	chr19:46838136-46850992	-	protein_coding	0.506658938	0.140286184	3.611609669	0.000304302	0.001716594	Yes	OK	3692.601664	3048.892164	4336.311163	3000.619939	3112.186422	3033.87013	3468.866598	5547.573531	3992.493361
ENSG00000172009	THOP1	chr19:2785460-2815807	+	protein_coding	0.506185949	0.150957128	3.353176868	0.000798896	0.004073679	Yes	OK	1576.263068	1300.874407	1851.651728	1304.434709	1285.207353	1312.981159	1494.153527	2400.833992	1659.967667
ENSG00000144504	ANKMY1	chr2:240479422-240569209	-	protein_coding	0.505563371	0.176090362	2.871045102	0.004091171	0.016743084	Yes	OK	203.9733637	167.8506103	240.0961172	148.0879984	176.0558017	179.4080306	197.9316132	273.9737999	248.3829386
ENSG00000117410	ATP6V0B	chr1:43974487-43978295	+	protein_coding	0.504819198	0.143843521	3.509502513	0.000448946	0.002434008	Yes	OK	1589.352148	1312.459836	1866.244461	1178.40237	1392.441341	1366.535795	1478.50312	2275.724569	1844.505695
ENSG00000137563	GGH	chr8:63015079-63039171	-	protein_coding	0.504207899	0.112859478	4.467572487	7.91122E-06	6.20976E-05	Yes	OK	1225.727824	1015.269733	1436.185915	1075.47596	1009.920099	960.4131391	1566.881887	1240.008585	1501.667273
ENSG00000133315	MACROD1	chr11:63998558-64166106	-	protein_coding	0.503406718	0.19367089	2.59928953	0.009341694	0.033749844	Yes	OK	192.933475	158.6902111	227.1767388	149.1382679	191.260621	135.6717445	209.8995712	273.9737999	197.6568455
ENSG00000181472	ZBTB2	chr6:151364117-151391548	-	protein_coding	0.502862787	0.108401895	4.638874503	3.50312E-06	2.91956E-05	Yes	OK	783.4673993	649.4596592	917.4751393	671.1222056	634.6011399	642.6556321	987.8168417	831.4233811	933.1851952
ENSG00000100580	TMED8	chr14:77335021-77377109	-	protein_coding	0.502486796	0.112372687	4.471609691	7.7633E-06	6.09947E-05	Yes	OK	689.4784441	571.3429037	807.6139845	542.9893275	588.1864285	582.8529552	820.2654296	733.2362389	869.3402849
ENSG00000105486	LIG1	chr19:48115445-48170603	-	protein_coding	0.502367576	0.112919605	4.448895971	8.63128E-06	6.72048E-05	Yes	OK	1057.152827	873.3129664	1240.992688	858.0701752	905.8871253	855.9815988	1150.765193	1422.129897	1150.082973
ENSG00000197860	SGTB	chr5:65665928-65723035	-	protein_coding	0.501407498	0.128672417	3.896775325	9.7482E-05	0.000610328	Yes	OK	1724.354897	1429.264827	2019.444967	1408.411389	1416.44895	1462.93414	2333.751812	1601.083882	2123.499207
ENSG00000116096	SPR	chr2:72887360-72892158	+	protein_coding	0.500906226	0.137428092	3.644860518	0.000267537	0.001526397	Yes	OK	340.8742146	281.6338511	400.114578	262.5673731	298.4946093	283.8395708	419.7991424	422.8381766	357.706415
ENSG00000138641	HERC3	chr4:88592423-88708542	+	protein_coding	0.500832681	0.108500691	4.615939996	3.9132E-06	3.23837E-05	Yes	OK	1399.677721	1160.800065	1638.555377	1252.971504	1131.558653	1097.870038	1699.450037	1444.301188	1771.914907
