# Supplementary material for: Genetic Investigation and Transcriptome Profiling in a Nuclear Family With Peutz–Jeghers Syndrome
Source: Hum Mutat. 2025 Aug 15;2025:5530710. doi: 10.1155/humu/5530710 (PMC12373473; doi:10.1155/humu/5530710)
Supplement: Supporting Information — Additional supporting information can be found online in the Supporting Information section. Table S1: List of primer sequences. Figure S1: Principal component analysis of RNA-seq. Figure S2: Volcano plot of differentially expressed protein coding genes obtained from RNA-seq analysis of PJS1-2 versus healthy matched control. Figure S3: Sketch of human p53 signaling pathway obtained from KEGG. Figure S4: Sketch of human Wnt signaling pathway obtained from KEGG. Figure S5: Sketch of the eukaryotic DNA mismatch repair pathway obtained from KEGG. Table S2: Differentially expressed genes in dermal fibroblasts from PJS1-2 versus a healthy control individual; n = 3 triplicates per genotype. Table S3: Filtered exome sequencing data showing 134 rare variants (MAF < 1% in public databases) present in both siblings (PJS1-1 and PJS1-2) and with mucocutaneous pigmentation that were absent from the mother (PJS1-6), who did not present with pigmentation. [file 5530710.f1.zip › Khan et al, Supplementary Table 3_R3_ES.pdf]

Gene	Variation Type	Chr	Start Position	Stop Position	dbSNP	Transcript	AA Change	Nucleotide	Exon	Zygosity	Region	Effect
OR5M8	SNP	11	56258695	56258696	rs1296364075	NM_001005282.1	p.Asn51Asp	c.151A>G	1	het	EXONIC	NON_SYNONYMOUS
PLCB3	SNP	11	64022270	64022271	rs764499162	NM_000932.2	p.Tyr77Asn	c.229T>A	3	het	EXONIC	NON_SYNONYMOUS
ROCK2	SNP	2	11337476	11337477	rs937064236	NM_004850.5		c.3280-3T>C	26	het	SPLICE_REGION	
ADRA2C	SNP	4	3769416	3769417	rs1170395344	NM_000683.3	p.Arg362Gly	c.1084C>G	1	het	EXONIC	NON_SYNONYMOUS
PMS2	SNP	7	6027079	6027080	rs1187749040	NM_000535.7	p.Thr439Ile	c.1316C>T	11	het	EXONIC	NON_SYNONYMOUS
ABI3BP	SNP	3	100645150	100645151	rs760200730	NM_001349329.1	p.Ile92Thr	c.275T>C	2	het	EXONIC	NON_SYNONYMOUS
GLCCI1	SNP	7	8126022	8126023	rs541845412	NM_138426.4	p.Arg500Gln	c.1499G>A	8	het	EXONIC	NON_SYNONYMOUS
TRIML1	INDEL	4	189068400	189068404	rs757294900	NM_178556.4	p.Ile429del	c.1285_1287delATC	6	het	EXONIC	NON_FRAMESHIFT
WDR97	SNP	8	145163641	145163642	rs1284540187	NM_001316309.1	p.Phe225Ile	c.673T>A	3	het	EXONIC	NON_SYNONYMOUS
PI4KB	SNP	1	151274319	151274320	rs769381237	NM_002651.3	p.Gln595Gln	c.1785G>A	9	het	SPLICE_REGION	
P2RX2	SNP	12	133196869	133196870	rs879315490	NM_170682.3	p.Met191Thr	c.572T>C	6	het	EXONIC	NON_SYNONYMOUS
ABCG2	SNP	4	89015757	89015758	rs767083239	NM_001257386.2	p.Gln594Pro	c.1781A>C	15	het	EXONIC	NON_SYNONYMOUS
ZNF140	SNP	12	133682962	133682963	rs766513108	NM_003440.3	p.Thr367Asn	c.1100C>A	5	het	EXONIC	NON_SYNONYMOUS
MEGF11	SNP	15	66257348	66257349	rs369781201	NM_032445.2	p.Pro337Ser	c.1009C>T	9	het	EXONIC	NON_SYNONYMOUS
KRR1	SNP	12	75900282	75900283	rs755848514	NM_007043.7	p.Pro167Arg	c.500C>G	4	het	EXONIC	NON_SYNONYMOUS
ABCA1	SNP	9	107571771	107571772	rs749089795	NM_005502.4	p.Arg1417Cys	c.4249C>T	30	het	EXONIC	NON_SYNONYMOUS
CCDC66	SNP	3	56651319	56651320	rs374670135	NM_001141947.2	p.Asn675Ser	c.2024A>G	14	het	EXONIC	NON_SYNONYMOUS
SLC35D1	SNP	1	67487262	67487263	rs368326325	NM_015139.3	p.Ala251Val	c.752C>T	9	het	EXONIC	NON_SYNONYMOUS
PTPRD	INDEL	9	8331573	8331574	rs3215098	NM_002839.3		c.5534+7_5534+8insAGTTACAGTTCAAGAATGGTAAGTT	44	het	SPLICE_REGION	
PPP2R2B	INDEL	5	146258289	146258290	rs10591869	NM_181675.3	p.Ser18_Ser19dup	c.52_57dupAGCAGC	1	het	EXONIC	NON_FRAMESHIFT
KLF4	SNP	9	110249806	110249807	rs1218480702	NM_004235.6	p.Ala290Ser	c.868G>T	3	het	EXONIC	NON_SYNONYMOUS
OR10R2	SNP	1	158450070	158450071	rs774306437	NM_001004472.1	p.Leu135Ser	c.404T>C	1	het	EXONIC	NON_SYNONYMOUS
KMT2E	SNP	7	104749529	104749530	rs775045007	NM_182931.2	p.Asp1204His	c.3610G>C	23	het	EXONIC	NON_SYNONYMOUS
AP5B1	SNP	11	65545574	65545575	rs372599668	NM_138368.4	p.Arg797Cys	c.2389C>T	2	het	EXONIC	NON_SYNONYMOUS
ZBTB4	SNP	17	7366367	7366368	rs761673519	NM_001128833.2	p.Glu645Lys	c.1933G>A	4	het	EXONIC	NON_SYNONYMOUS
LCN2	SNP	9	130914544	130914545	rs149368200	NM_005564.5	p.Val187Ile	c.559G>A	5	het	EXONIC	NON_SYNONYMOUS
STAT3	INDEL	17	40475649	40475650	rs1555564070	NM_139276.2		c.1601-8_1601-7insG	17	het	SPLICE_REGION	
LMF1	SNP	16	919980	919981	rs778629426	NM_022773.3	p.Glu440Lys	c.1318G>A	9	het	EXONIC	NON_SYNONYMOUS
ADAM19	SNP	5	156908884	156908885	rs770053170	NM_033274.4	p.Leu873Ile	c.2617C>A	22	het	EXONIC	NON_SYNONYMOUS
SERPINA11	SNP	14	94909109	94909110	rs367819510	NM_001080451.2	p.Gly368Arg	c.1102G>A	5	het	EXONIC	NON_SYNONYMOUS
OPLAH	SNP	8	145111554	145111555	rs377166928	NM_017570.5	p.Arg604Cys	c.1810C>T	13	het	EXONIC	NON_SYNONYMOUS
PLEC	SNP	8	145004237	145004238	rs376936955	NM_201384.2		c.2613-8C>T	21	het	SPLICE_REGION	
BEST1	SNP	11	61730007	61730008	rs778760005	NM_004183.4	p.Arg461Thr	c.1382G>C	10	het	EXONIC	NON_SYNONYMOUS
IMPG2	SNP	3	100962643	100962644	rs139105788	NM_016247.4	p.Arg844Gln	c.2531G>A	13	het	EXONIC	NON_SYNONYMOUS
EPPK1	SNP	8	144941996	144941997	rs782233446	NM_031308.3	p.Arg1809Gly	c.5425A>G	2	het	EXONIC	NON_SYNONYMOUS
AKNAD1	SNP	1	109373213	109373214	rs200586359	NM_152763.5	p.Pro602Leu	c.1805C>T	10	het	EXONIC	NON_SYNONYMOUS
GLIPR1L2	SNP	12	75785107	75785108	rs781325607	NM_001270396.2	p.Arg71Gln	c.212G>A	1	het	EXONIC	NON_SYNONYMOUS
IPO13	SNP	1	44415291	44415292	rs551718604	NM_014652.4	p.Asp96Glu	c.288C>G	2	het	EXONIC	NON_SYNONYMOUS
COL16A1	SNP	1	32138334	32138335	rs144759774	NM_001856.4	p.Asn989Ser	c.2966A>G	45	het	EXONIC	NON_SYNONYMOUS
HIST1H1E	SNP	6	26157135	26157136	rs545499106	NM_005321.2	p.Pro173Leu	c.518C>T	1	het	EXONIC	NON_SYNONYMOUS
FAM120B	INDEL	6	170627471	170627508	rs746196363	NM_032448.2	p.Ala335_Asp346del	c.1002_1037delCGCCGAATCCAGGGAAGAAGTTCCCATGTGTTCAGA	2	het	EXONIC	NON_FRAMESHIFT
MELK	SNP	9	36651736	36651737	rs775557883	NM_014791.4		c.922-6C>T	11	het	SPLICE_REGION	
PLEC	SNP	8	144995056	144995057	rs782297488	NM_201384.2	p.Arg2978Cys	c.8932C>T	32	het	EXONIC	NON_SYNONYMOUS
TNKS1BP1	SNP	11	57080492	57080493	rs775774343	NM_033396.3	p.Asp557Asn	c.1669G>A	5	het	EXONIC	NON_SYNONYMOUS
SLC5A3	SNP	21	35469012	35469013	rs774586769	NM_006933.7	p.Ile506Val	c.1516A>G	2	het	EXONIC	NON_SYNONYMOUS
ZKSCAN4	INDEL	6	28215928	28215933	rs754363065	NM_019110.5		c.572-6_572-3delTCTT	2	het	SPLICE_REGION	
ZFYVE9	SNP	1	52703474	52703475	rs770838150	NM_004799.3	p.Val129Ala	c.386T>C	4	het	EXONIC	NON_SYNONYMOUS
CDK5RAP2	SNP	9	123280735	123280736	rs141387242	NM_018249.6	p.Arg427Gln	c.1280G>A	12	het	EXONIC	NON_SYNONYMOUS
RABL2B	SNP	22	51215133	51215134	rs781937910	NM_001350012.1	p.His61Tyr	c.181C>T	5	het	EXONIC	NON_SYNONYMOUS
TNFAIP1	SNP	17	26668283	26668284	rs782502673	NM_021137.5	p.Pro133Ala	c.397C>G	4	het	EXONIC	NON_SYNONYMOUS
PRRC2B	SNP	9	134351332	134351333	rs201425705	NM_013318.3	p.Asp1273Tyr	c.3817G>T	15	het	EXONIC	NON_SYNONYMOUS
ATAD3B	SNP	1	1407345	1407346	rs766445790	NM_031921.5	p.Gly28Arg	c.82G>A	1	het	EXONIC	NON_SYNONYMOUS
PLXNA1	SNP	3	126733143	126733144	rs199523740	NM_032242.3	p.Ala844Thr	c.2530G>A	11	het	EXONIC	NON_SYNONYMOUS
AK5	SNP	1	78001725	78001726	rs546493955	NM_174858.3		c.1620+3G>A	13	het	SPLICE_REGION	
RHBG	SNP	1	156354374	156354375	rs548949926	NM_020407.5	p.Asp431Asn	c.1291G>A	10	het	EXONIC	NON_SYNONYMOUS
SELENOO	SNP	22	50647011	50647012	rs200468030	NM_031454.2	p.Arg269His	c.806G>A	3	het	EXONIC	NON_SYNONYMOUS

TNS2	SNP	12	53450827	53450828	rs540051071	NM_170754.2	p.Arg265Trp	c.793C>T	11	het	EXONIC	NON_SYNONYMOUS
DLK2	SNP	6	43418737	43418738	rs200880387	NM_023932.4	p.Val231Ile	c.691G>A	6	het	EXONIC	NON_SYNONYMOUS
KIF7	SNP	15	90191538	90191539	rs570856269	NM_198525.3	p.Ile464Val	c.1390A>G	5	het	EXONIC	NON_SYNONYMOUS
AGBL4	SNP	1	49052813	49052814	rs376232765	NM_032785.4	p.Ala377Thr	c.1129G>A	11	het	EXONIC	NON_SYNONYMOUS
GLOD4	SNP	17	674601	674602	rs141456440	NM_001366247.1	p.Glu184Gly	c.551A>G	6	het	EXONIC	NON_SYNONYMOUS
SLFNL1	SNP	1	41481809	41481810	rs201233485	NM_144990.3	p.Gly398Arg	c.1192G>A	5	het	EXONIC	NON_SYNONYMOUS
NRDE2	SNP	14	90770498	90770499	rs183085971	NM_017970.4	p.Ala262Val	c.785C>T	5	het	EXONIC	NON_SYNONYMOUS
ZNF786	SNP	7	148769172	148769173	rs188243675	NM_152411.3	p.Trp231Arg	c.691T>A	4	het	EXONIC	NON_SYNONYMOUS
OR9Q1	SNP	11	57946998	57946999	rs149361937	NM_001005212.3	p.Phe28Cys	c.83T>G	3	het	EXONIC	NON_SYNONYMOUS
WASHC5	SNP	8	126091073	126091074	rs150026441	NM_014846.4	p.Asn206Ser	c.617A>G	6	het	EXONIC	NON_SYNONYMOUS
ZFP3	SNP	17	4996299	4996300	rs201869640	NM_153018.3	p.Met501Val	c.1501A>G	2	het	EXONIC	NON_SYNONYMOUS
MX1	SNP	21	42813794	42813795	rs199502305	NM_002462.5	p.Ala295Thr	c.883G>A	10	het	EXONIC	NON_SYNONYMOUS
VEZF1	INDEL	17	56056603	56056613	rs57786397	NM_007146.3	p.Gln347_Gln349del	c.1038_1046delGCAGCAGCA	5	het	EXONIC	NON_FRAMESHIFT
CYP4Z1	SNP	1	47564767	47564768	rs531653571	NM_178134.3	p.Ser293Ser	c.879C>T	8	het	SPLICE_REGION	
THSD7A	INDEL	7	11871495	11871496	rs748972424	NM_015204.3	p.Gln24_Leu25dup	c.71_76dupAGCTGC	1	het	EXONIC	NON_FRAMESHIFT
WDR78	SNP	1	67313186	67313187	rs147253554	NM_024763.5	p.Arg424His	c.1271G>A	8	het	EXONIC	NON_SYNONYMOUS
SLAMF6	SNP	1	160460001	160460002	rs149372302	NM_001184714.2	p.Arg261Gln	c.782G>A	5	het	EXONIC	NON_SYNONYMOUS
RIMBP2	SNP	12	130892272	130892273	rs148542555	NM_001351227.1	p.Glu992Lys	c.2974G>A	18	het	EXONIC	NON_SYNONYMOUS
TSHB	SNP	1	115576653	115576654	rs201857310	NM_000549.4	p.Arg75Gly	c.223A>G	3	het	EXONIC	NON_SYNONYMOUS
DMXL2	SNP	15	51828635	51828636	rs201747716	NM_001174116.1	p.Cys681Gly	c.2041T>G	12	het	EXONIC	NON_SYNONYMOUS
SSUH2	SNP	3	8661566	8661567	rs181750881	NM_001256748.1	p.Cys372Ser	c.1115G>C	12	het	EXONIC	NON_SYNONYMOUS
TARSL2	SNP	15	102252162	102252163	rs138576200	NM_152334.3	p.Met244Ile	c.732G>A	5	het	EXONIC	NON_SYNONYMOUS
IGDCC4	SNP	15	65703645	65703646	rs142198652	NM_020962.3	p.Val45Met	c.133G>A	2	het	EXONIC	NON_SYNONYMOUS
LY6E	SNP	8	144103119	144103120	rs111560737	NM_002346.3	p.Asp104Asn	c.310G>A	4	het	EXONIC	NON_SYNONYMOUS
FREM2	SNP	13	39425905	39425906	rs555028352	NM_207361.6	p.Val2276Met	c.6826G>A	11	het	EXONIC	NON_SYNONYMOUS
ZNF710	SNP	15	90610516	90610517	rs144139243	NM_198526.4	p.Glu50Gln	c.148G>C	2	het	EXONIC	NON_SYNONYMOUS
OR1A1	SNP	17	3119647	3119648	rs62090945	NM_014565.2	p.Thr245Met	c.734C>T	1	het	EXONIC	NON_SYNONYMOUS
MAGI1	INDEL	3	65342255	65342280	rs558263084	NM_001033057.1	p.Arg1388_Arg1395del	c.4162_4185delAGAGGGGGCTCGCCCGAGCGCAGG	23	het	EXONIC	NON_FRAMESHIFT
ADAMTSL3	SNP	15	84700166	84700167	rs148548737	NM_207517.3	p.Asn1579Lys	c.4737C>G	28	het	EXONIC	NON_SYNONYMOUS
KRBA1	SNP	7	149422516	149422517	rs113560395	NM_001290187.1	p.Pro447Leu	c.1340C>T	10	het	EXONIC	NON_SYNONYMOUS
CCDC144A	SNP	17	16594014	16594015	rs202033635	NM_014695.2	p.Ala101Thr	c.301G>A	1	het	EXONIC	NON_SYNONYMOUS
ANKS6	SNP	9	101558580	101558581	rs745881769	NM_173551.5	p.Val65Phe	c.193G>T	1	het	EXONIC	NON_SYNONYMOUS
GAA	SNP	17	78092472	78092473	rs377286472	NM_000152.5	p.Val890Leu	c.2668G>C	19	het	EXONIC	NON_SYNONYMOUS
STRIP2	SNP	7	129074434	129074435	rs200680852	NM_020704.3	p.Glu41Gly	c.122A>G	1	het	EXONIC	NON_SYNONYMOUS
TRMT12	SNP	8	125463936	125463937	rs148858836	NM_017956.3	p.Gly257Arg	c.769G>C	1	het	EXONIC	NON_SYNONYMOUS
RIN3	SNP	14	93118789	93118790	rs139248637	NM_024832.5	p.Ile466Leu	c.1396A>C	6	het	EXONIC	NON_SYNONYMOUS
KDM6B	SNP	17	7751530	7751531	rs201403136	NM_001348716.1	p.Pro642Leu	c.1925C>T	11	het	EXONIC	NON_SYNONYMOUS
TP53AIP1	INDEL	11	128807649	128807650	rs141395772	NM_022112.2	p.Gln22fs	c.63dupG	2	het	EXONIC	FRAMESHIFT
FCRLB	INDEL	1	161695749	161695751	rs371919304	NM_001002901.3	p.Ser150fs	c.448delA	6	het	EXONIC	FRAMESHIFT
OMA1	SNP	1	58971747	58971748	rs77244905	NM_145243.5	p.Asp450Val	c.1349A>T	8	het	EXONIC	NON_SYNONYMOUS
CYP4Z1	SNP	1	47533339	47533340	rs548967488	NM_178134.3		c.177+1G>T	1	het	SPLICE_DONOR	
TBC1D3G	SNP	17	34797940	34797941	rs879400104	NM_001291462.1	p.Arg399Trp	c.1195C>T	14	het	EXONIC	NON_SYNONYMOUS
PCDHB12	SNP	5	140590367	140590368	rs369072390	NM_018932.3	p.Leu630Pro	c.1889T>C	1	het	EXONIC	NON_SYNONYMOUS
ZDHHC12	SNP	9	131483667	131483668	rs78428039	NM_032799.5	p.Val199Ala	c.596T>C	5	het	EXONIC	NON_SYNONYMOUS
PKN3	SNP	9	131482719	131482720	rs7874787	NM_001317926.1	p.Arg821Cys	c.2461C>T	22	het	EXONIC	NON_SYNONYMOUS
PKN3	SNP	9	131482516	131482517	rs7874430	NM_001317926.1	p.Ala804Ala	c.2412A>G	21	het	SPLICE_REGION	
PCDHB15	SNP	5	140626116	140626117	rs147373970	NM_018935.4	p.Gly324Ala	c.971G>C	1	het	EXONIC	NON_SYNONYMOUS
PCDHB15	SNP	5	140626617	140626618	rs141098531	NM_018935.4	p.Leu491Pro	c.1472T>C	1	het	EXONIC	NON_SYNONYMOUS
PCDHB15	SNP	5	140625428	140625429	rs142267472	NM_018935.4	p.Leu95Met	c.283C>A	1	het	EXONIC	NON_SYNONYMOUS
PCDHB9	SNP	5	140567241	140567242	rs193259137	NM_019119.4	p.Tyr117Ser	c.350A>C	1	het	EXONIC	NON_SYNONYMOUS
PCDHB10	SNP	5	140572474	140572475	rs143882112	NM_018930.3	p.Tyr117Ser	c.350A>C	1	het	EXONIC	NON_SYNONYMOUS
PCDHB6	SNP	5	140531090	140531091	rs145637479	NM_018939.3	p.Thr418Ile	c.1253C>T	1	het	EXONIC	NON_SYNONYMOUS
PCDHB9	SNP	5	140567949	140567950	rs192033636	NM_019119.4	p.Ser353Phe	c.1058C>T	1	het	EXONIC	NON_SYNONYMOUS
PCDHB6	SNP	5	140529937	140529938	rs141476251	NM_018939.3	p.Val34Ile	c.100G>A	1	het	EXONIC	NON_SYNONYMOUS
DUOX2	SNP	15	45399074	45399075	rs373790251	NM_001363711.1	p.Pro596Ser	c.1786C>T	15	het	EXONIC	NON_SYNONYMOUS
SERINC2	SNP	1	31897586	31897587	rs112561697	NM_178865.5	p.Asp87Asn	c.259G>A	3	het	EXONIC	NON_SYNONYMOUS
F3	SNP	1	94996061	94996062	rs3789683	NM_001993.5	p.Gly281Glu	c.842G>A	6	het	EXONIC	NON_SYNONYMOUS

EBNA1BP2	SNP	1	43637245	43637246	rs11559316	NM_006824.2	p.Pro76Arg	c.227C>G	3	het	EXONIC	NON_SYNONYMOUS
EPHA8	SNP	1	22923858	22923859	rs144329757	NM_020526.5	p.Pro607His	c.1820C>A	10	het	EXONIC	NON_SYNONYMOUS
SAC3D1	SNP	11	64811899	64811900	rs193165731	NM_013299.4	p.Arg260Cys	c.778C>T	2	het	EXONIC	NON_SYNONYMOUS
SYNM	SNP	15	99672969	99672970	rs35238587	NM_145728.2	p.Val1468Ile	c.4402G>A	4	het	EXONIC	NON_SYNONYMOUS
HCRTR1	SNP	1	32089220	32089221	rs7516785	NM_001525.2	p.Arg279Gln	c.836G>A	7	het	EXONIC	NON_SYNONYMOUS
PDCD7	INDEL	15	65425743	65425744	rs566766250	NM_005707.2	p.Ala125dup	c.373_375dupGCG	1	het	EXONIC	NON_FRAMESHIFT
HP1BP3	SNP	1	21071405	21071406	rs143662289	NM_016287.3	p.Ile516Leu	c.1546A>C	13	het	EXONIC	NON_SYNONYMOUS
FER1L6	SNP	8	125022904	125022905	rs151336286	NM_001039112.2	p.Thr591Ile	c.1772C>T	14	het	EXONIC	NON_SYNONYMOUS
HIVEP3	SNP	1	41976374	41976375	rs200138511	NM_024503.5	p.Arg2323Gln	c.6968G>A	9	het	EXONIC	NON_SYNONYMOUS
BOD1L1	SNP	4	13604033	13604034	rs61995956	NM_148894.3	p.Gln1497Arg	c.4490A>G	10	het	EXONIC	NON_SYNONYMOUS
TFPT	SNP	19	54613510	54613511	rs147098569	NM_013342.3		c.283-7C>A	2	het	SPLICE_REGION	
B4GALNT2	SNP	17	47246955	47246956	rs61743617	NM_001159387.1	p.Arg463Trp	c.1387C>T	11	het	EXONIC	NON_SYNONYMOUS
ACAP1	SNP	17	7251712	7251713	rs35019942	NM_014716.4	p.Arg533Trp	c.1597C>T	17	het	EXONIC	NON_SYNONYMOUS
PCDHA9	SNP	5	140230532	140230533	rs148067258	NM_014005.4	p.Arg818Gln	c.2453G>A	1	het	EXONIC	NON_SYNONYMOUS
COL16A1	SNP	1	32149759	32149760	rs34770879	NM_001856.4	p.Gly745Ser	c.2233G>A	32	het	EXONIC	NON_SYNONYMOUS
TNRC6B	SNP	22	40666282	40666283		NM_001162501.2	p.Pro988Pro	c.2964T>G	6	het	SPLICE_REGION	
ARHGEF11	SNP	1	156907260	156907261		NM_198236.2	p.Ser1407Leu	c.4220C>T	39	het	EXONIC	NON_SYNONYMOUS
ITGA7	SNP	12	56092288	56092289		NM_002206.2	p.Val357Ala	c.1070T>C	7	het	EXONIC	NON_SYNONYMOUS
MAST2	SNP	1	46495852	46495853		NM_015112.3	p.Ser832Arg	c.2496T>G	21	het	EXONIC	NON_SYNONYMOUS
SURF6	SNP	9	136199380	136199381		NM_006753.6		c.606+3G>A	4	het	SPLICE_REGION	
DENND2C	SNP	1	115168445	115168446		NM_001256404.2	p.Gln54Glu	c.160C>G	4	het	EXONIC	NON_SYNONYMOUS
